# Water‐Catalytic Deconstructive and Proton Transfer Cyclopropanation of Sulfoxonium Ylide with Olefin

**DOI:** 10.1002/advs.202502430

**Published:** 2025-05-30

**Authors:** Xianglin Yu, Liuting Huang, Haiyue Yang, Lijuan Song, Yi Jin

**Affiliations:** ^1^ Key Laboratory of Medicinal Chemistry for Natural Resource, Ministry of Education Yunnan Key Laboratory of Research and Development for Natural Products School of Pharmacy School of Chemical Science and Technology Yunnan University Kunming 650091 China; ^2^ School of Science Harbin Institute of Technology (Shenzhen) Shenzhen 518055 China

**Keywords:** deconstructive cyclopropanation, sulfoxonium ylide • proton transfer, water‐catalysis

## Abstract

Cyclopropane rings, with their distinct structure and reactivity, have long been a focus in organic chemistry and are significant pharmacophores in medicinal chemistry. Conventional direct cyclopropanation methods for olefins do not modify the functional groups on the *α*‐ or *β*‐carbon of olefins. Herein, a novel deconstructive cyclopropanation reaction is designed for olefins using a close–open–close ring strategy. This enables the migration of functional groups to the *α*‐ or *β*‐carbon of olefins, leading to the formation of regioselective cyclopropane compounds, which is a previously unreported approach. By exploiting the zwitterionic property of sulfoxonium ylides and combining them with Density Functional Theory (DFT) computations, the reaction is proposed to proceed via a [2 + 2] cycloaddition to form a strained cyclobutene intermediate, followed by cyclobutane ring‐opening and nucleophilic substitution through a water‐involved proton‐shuttle process for ring closure. Hydrogen‐bonding interactions play a significant role in controlling the regioselectivity.

## Graphical abstract

Water‐catalytic deconstructive and proton transfer cyclopropanation of sulfoxonium ylide with olefin is developed through a sequential water‐mediated ring‐closing/opening reaction. This atom‐economical, environmentally friendly methodology offers easy access to a range of cyclopropyl ketones with moderate to good yields.

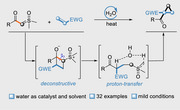

## 1 Introduction

The unique structure and reactivity of cyclopropane rings have long been the focus of attention for organic chemists. This strained three‐membered carbon ring can react with nucleophilic reagents, electrophilic reagents, and radical compounds.^[^ ^1^ ^]^ In addition, the conformational rigidity of cyclopropane and the highly directional spatial arrangement of its substituents make it an important pharmacophore in medicinal chemistry.^[^ ^2^ ^]^

**Scheme 1:**
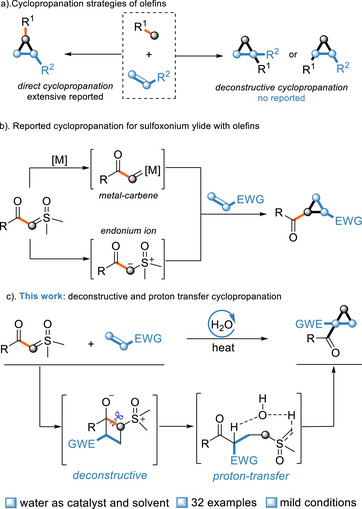
Cyclopropanation Strategies.

Popular methods for the direct cyclopropanation of olefins can be divided into three main groups: 1) halomethylmetal‐mediated cyclopropanation, e.g. Simmons–Smith reaction; 2) transition‐metal catalyzed decomposition of carbene precursors (diazo compounds, ketones, etc); 3) Michael reaction‐initiated ring closure (MIRC).^[^ ^3^ ^]^ Direct cyclopropanation does not alter the functional groups on the *α*‐ or *β*‐carbon of olefins. Recently, the deconstruction of cycloalkane derivatives by ring‐opening constitutes a highly attractive scaffold hopping strategy in organic synthesis.^[^ ^4^ ^]^ Herein, we propose a deconstructive cyclopropanation reaction that employs the close‐open‐close ring strategy for the cyclopropanation of olefins. This enables the migration of functional groups from one carbon skeleton to the *α*‐ or *β*‐carbon of olefins, thereby achieving regioselective synthesis of cyclopropane compounds. To date, such a cyclopropanation strategy has remained unreported (**Scheme** **1**a).

Sulfoxonium ylides have been widely employed as one‐carbon synthons in numerous classical transformation reactions.^[^ ^5^ ^]^ Sulfoxonium ylides can serve either as a carbene precursor for the cyclopropanation of olefins catalyzed by transition metals, or as a nucleophilic 1,1‐dipolar species to undergo cyclopropanation with olefins^[^ ^6^ ^]^ (Scheme 1b). All the obtained products are direct cyclopropanation products where the functional groups remain at their original positions on the olefins, without migration from other carbon skeletons.

Sulfoxonium ylides are a class of zwitterionic compounds characterized by a carbanion adjacent to a positively charged sulfur atom.^[^ ^5^ ^]^ Leveraging this property, we designed a strategy of closing‐opening‐closing the ring for the cyclopropanation of olefins, enabling the migration of functional groups and regioselectively obtaining cyclopropane compounds. The specific process is as follows: 1) Olefins undergo [2 + 2] cycloaddition to form highly strained cyclobutanes,^[^ ^7^ ^]^ which provides the driving force for subsequent reactions; 2) The ring strain prompts the ring‐opening of the cyclobutane; during this process, bond reorganization triggers the migration of functional groups, and the regioselectivity is further regulated to determine the direction of functional‐group migration^[^ ^8^ ^]^; 3) The ring‐opened intermediate undergoes nucleophilic substitution to regenerate the cyclic structure via a water‐mediated proton‐shuttle process, ultimately directing the functional group to the *α*‐ or *β*‐carbon of the olefin with high regioselectivity. Notably, H_2_O serves as a proton‐transfer reagent to achieve proton transfer within the deconstructive intermediate (Scheme 1c).^[^ ^9^ ^]^

## 2 Results and Discussion

Our initial attempt involved the deconstructive and proton transfer cyclopropanation reaction of sulfoxonium ylide **1a** and alkene **2a** by using H_2_O in DMSO. When the reaction was performed at 100 °C for 12 h (**Table** **1**, entry 1), the envisioned 1,1‐disubstituted cyclopropyl ketone **3a** was obtained in 50% isolated yield. Based on these results, we continued to optimize the reaction conditions. Increasing the amount of water significantly improved the product yield, when DMSO/H_2_O (1/1) as solvent, product **3a** was obtained in 78% isolated yield (Table 1, entries 2–4). Solvent screening revealed that DMSO/H_2_O (1/1) was the best solvent; by contrast, using only H_2_O (0.5 mL) as the solvent still afforded a 75% isolated yield. DMSO not only elevated the boiling point of the reaction solvent but also enabled homogeneous reaction conditions. And product **3a** could not be obtained in the absence of H_2_O (entry 9). The optimum temperature was confirmed as 100°C (entries 13–17). Extending sulfoxonium ylide **1a** to 1.2 equivalents did not increase the yield of **3a** (entry 18). Substitution of H_2_O with CH_3_COOH or CH_3_CH_2_OH afforded the target product with reduced yields (entry 19–20).

**Table 1:** Optimized Reaction Conditions.^a)^, ^b)^, ^c)^, ^d)^, ^e)^, ^f)^

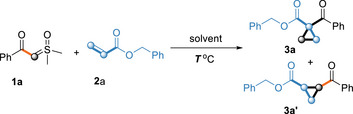				Yield (%)^b)^	
Entry	Solvent	H_2_O (X equiv.)	*T* (°C)	3a	3a’
1	DMSO	10	100	50	40
2	DMSO	100	100	60	37
3	DMSO/ H_2_O (4/1)	—	100	65	26
4	DMSO/ H_2_O (1/1)	—	100	78	15
5	DMF/ H_2_O (1/1)	—	100	63	35
6	PhCH_3_/ H_2_O (1/1)	—	100	35	25
7	DCE/ H_2_O (1/1)	—	100	20	28
8	CH_3_CN/ H_2_O (1/1)	—	100	30	25
9	DMSO	Trace	100	nd ***^c^***	40
10	H_2_O	—	100	64	20
**11**	**H_2_O (0.5 mL)**	**—**	**100**	**75**	**20**
12	H_2_O (0.2 mL)	—	100	70	20
13	H_2_O (0.5 mL)	—	90	70	20
14	H_2_O (0.5 mL)	—	80	64	16
15	H_2_O (0.5 mL)	—	70	50	15
16	H_2_O (0.5 mL)	—	60	42	10
17	H_2_O (0.5 mL)	—	25	nd	nd
18^d)^	H_2_O (0.5 mL)	—	100	73	20
19^e)^	DMSO	Trace	100	30	45
20^f)^	DMSO	Trace	100	15	40

^a)^ Reaction conditions: In a 5 mL reaction tube, sulfoxonium ylide **1a** (0.2 mmol), olefin **2a** (0.2 mmol), solvent 1 mL, under air (1 atm), stirred for 12 h at *T* °C;

^b)^ Isolated yield of **3a** based on **2a**;

^c)^ nd means not detected;

^d)^ Addition of sulfoxonium ylide **1a** (0.24 mmol);

^e)^ Addition of CH_3_COOH (2.0 mmol);

^f)^ Addition of CH_3_CH_2_OH (2.0 mmol).

Under optimized reaction conditions, the scope of sulfoxonium ylides (**1**) was investigated with a variety of olefins (**2**). A range of olefins was first examined (**Table** **2**). The reaction generally tolerated a broad range of substituted benzyl acrylates (**3b**–**3f**: R = *p*‐F, *p*‐Br, *p*‐Cl, *p*‐Me or *p*‐OMe) to afford high to better yields of the corresponding cyclopropane. Moreover, the cascade reaction produced equally satisfactory results by using a range of acrylates (**3g**–**3m**). A study on the electronic influence of the group indicated that although the alkyl substituents with different carbon numbers groups worked well and resulted in good yields (**3i**–**3m**), groups containing benzene achieved a higher yield (**3d**, 90% yield). Notably, both *N*,*N*‐dimethylacrylamide **2n** and 3‐Oxo‐3‐phenylpropene **2o** can be introduced as substrates in this reaction and showed a good yield. Compared to **3a**, the enhanced electron‐withdrawing character of phenoxy and phenyl groups in **3g** and **3o** relative to the benzyloxy group contributes to their improved yields. Afterwards, electron‐deficient alkenes lacking acyl groups (acrylonitrile **2p** and phenyl vinyl sulfone **2q**) were introduced as reaction substrates. Fortunately, both obtained their counterparts in acceptable yields. Noteworthy, olefin **2r** contains an additional alkene group, the target product still was obtained in a high yield, and no extra olefin was reacted to be detected. This indicates that the reaction exhibits good chemical selectivity. The olefin **2s** bearing additional carbonyl group demonstrates a lowered yield of the target product, whereas the olefin **2t** containing an additional reactive CH_2_ group successfully facilitates the formation of the desired product without transformation of the reactive CH_2_ group. Conventional cyclopropanation methods often suffer from regioselectivity issues when multiple CH_2_ groups are present, leading to the formation of cyclopropanes at various positions. In contrast, our approach achieves selective cyclopropanation at specific sites while preserving other CH_2_ moieties intact.^[^ ^10^ ^]^ Olefins containing *DL*‐menthol and cholesterol efficiently delivered the desired products **3u** and **3v** in moderate to moderate yields. To verify the structure of the cyclopropyl ketones, **3d** ^[^ ^11^ ^]^ was selected as the representative compound and was characterized by X‐ray crystallography (Table 2, **3d**).

**Table 2:** Reaction Scope of Olefins.^a)^, ^b)^

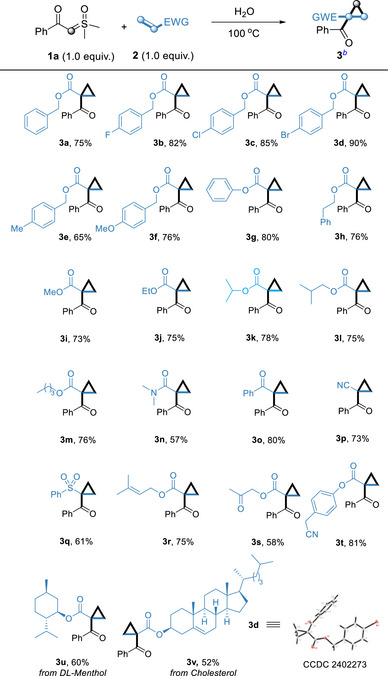

^a)^ Reaction conditions: In a 5 mL reaction tube, sulfoxonium ylide **1a** (0.2 mmol), olefins **2** (0.2 mmol), H_2_O 0.5 mL, under air (1 atm), stirred for 12 h at 100 °C;

^b)^ Isolated yield of **3** based on **1a**.

Then, a wide range of sulfoxonium ylides were employed for reaction under the optimized conditions (**Table** **3**). Single substitution at various positions underwent a smooth reaction under optimal conditions, furnishing the corresponding cyclopropane derivatives **4a–4k** in 63–88% yields. Both electron‐donating and electron‐withdrawing sulfoxonium ylides were well tolerated and efficiently converted into the desired cyclopropanes with moderate to good yields. Compared with the electron‐donating group, the electron‐withdrawing group provided higher yields. Notably, nitro‐ and cyano‐substituted sulfoxonium ylides can also afford cyclopropanes in good yields. The disubstituted aryl sulfoxonium ylide gave the products **4l** in 72% yield. The reaction condition was tolerant to furan, thiophene‐derived and naphthalene‐derived sulfoxonium ylides, furnishing products **4m,** **4n** and **4o** in 75%, 78% and 81% yield, respectively. The yield of alkyl sulfoxonium ylide is unsatisfactory, afforded the corresponding product **4p** in only 59% yield.

**Table 3:** Reaction Scope of Sulfoxonium Ylides **1**.^a)^, ^b)^

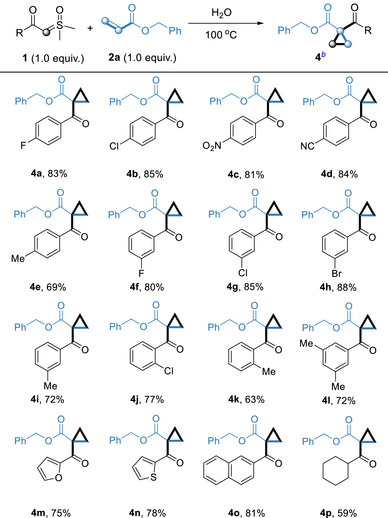

^a)^ Reaction conditions: In a 5 mL reaction tube, sulfoxonium ylide **1** (0.2 mmol), olefin **2a** (0.2 mmol), H_2_O 0.5 mL, under air (1 atm), stirred for 12 h at 100 °C;

^b)^ Isolated yield of **4** based on **1**.

**Scheme 2:**
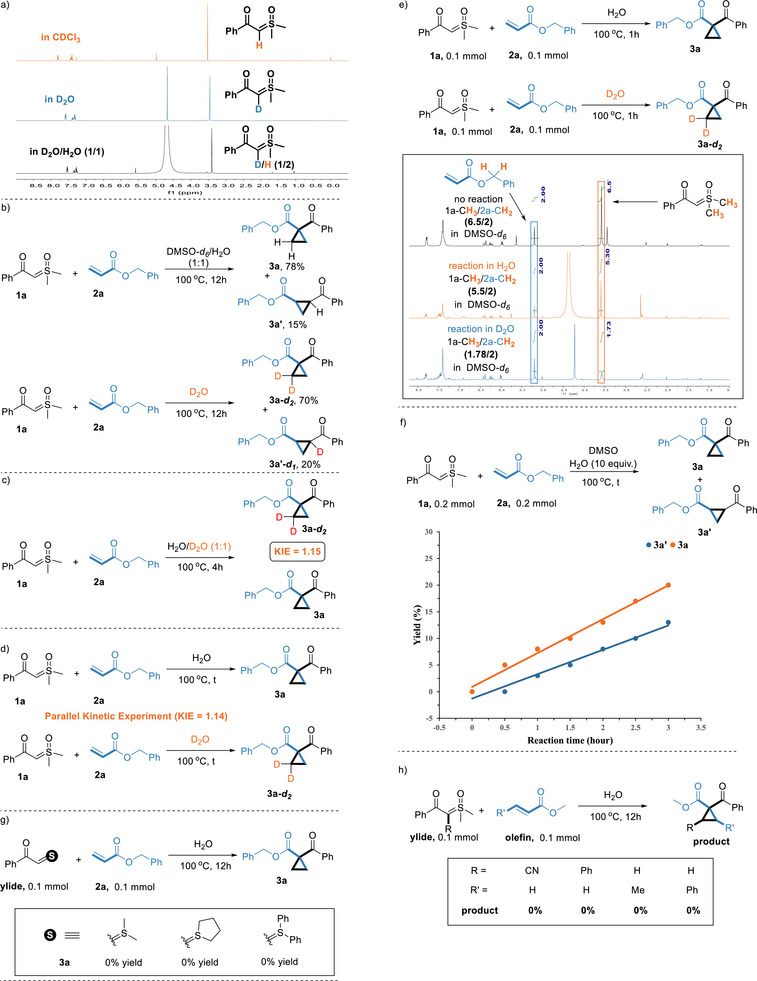
Mechanistic studies.

To gain insights into the reaction mechanism, we first conducted isotopic labeling experiments under the standard conditions (**Scheme** **2**).^[^ ^12^ ^]^ The ^1^H NMR spectrum of **1a** in D_2_O shows a complete D/H exchange of the carbonyl *α* C–H bond that reveals the reversible hydration of aqueous sulfoxonium ylides. The D/H ratio is 1/2 in the D_2_O/H_2_O (1/1) indicating that the reaction is primary kinetic isotope effect (Scheme 2a). When the reaction solvent was replaced with D_2_O, deuterated products **3a*‐d_2_*** and **3a'*‐d_1_*** were obtained at 70% and 20% yields, respectively (Scheme 2b). Competitive kinetic isotope effect experiments demonstrated that H_2_O addition to the intermediate exhibited a normal secondary kinetic isotope effect, accompanied by an irreversible rehybridization from *sp^3^* to *sp^2^* (Scheme 2c). Parallel kinetic isotope effect experiments revealed that the H_2_O addition step was not rate‐determining step in this catalytic cycle (Scheme 2d). When **1a** and **2a** were reacted separately in H_2_O and D_2_O for one hour, the ^1^H NMR spectrum of mixture showed significant D/H exchange at the CH_3_ group of **1a** (Scheme 2e). Monitoring **3a** and the by‐product **3a'** under initial conditions revealed that **3a** formed prior to **3a'**, indicating kinetic preference for **3a** (Scheme 2f). Replaced sulfoxonium ylide with other sulfur ylides, and the reaction cannot occur (Scheme 2g). Neither sulfoxonium ylides with an *α*‐C substituent nor non‐terminal alkenes afforded the target product (Scheme 2h).

**Scheme 3:**
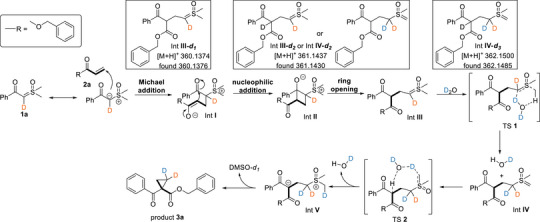
Proposed mechanism.

On the basis of these results and control experiments, we propose a plausible H_2_O catalytic cycle for water‐catalytic deconstructive and proton transfer cyclopropanation of sulfoxonium ylide **1** with alkene **2** (**Scheme** **3**). To more intuitively illustrate proton transfer in the reaction mechanism, we employed D_2_O instead of H_2_O for mechanistic diagram construction. The proposed mechanism initiates with a Michael addition between sulfoxonium ylide (**1a**) and benzyl acrylate (**2a**), yielding intermediate **I**. This intermediate subsequently undergoes intramolecular nucleophilic attack on the benzoyl carbonyl group, generating the zwitterionic cyclobutane intermediate **II**. Compared with cyclopropane, cyclobutane exhibits lower ring strain. Intermediate **I** preferentially undergoes subsequent transformation to form the less strained cyclobutane intermediate **II**. Following this, intermediate **II** participates in a cyclobutane ring‐opening by the alkoxide process to form the intermediate **III**. The reaction cascade continues through a water‐mediated 1,3‐proton transfer from intermediate **III** to intermediate **IV**, which subsequently undergoes a second proton transfer to afford intermediate **V**.^[^ ^13^ ^]^ Eventually, the ring was closed in intramolecular to form the cyclopropyl ketone **3a** and release DMSO. The H_2_O‐mediated proton transfer facilitates the formation of a carbanion intermediate between the two carbonyl groups, which ultimately drives cyclopropane ring closure rather than promoting dihydrofuran derivative formation through carbonyl group reactivity.^[7a]^ Deuterated intermediates are captured for the reaction by HRMS.

**Scheme 4:**
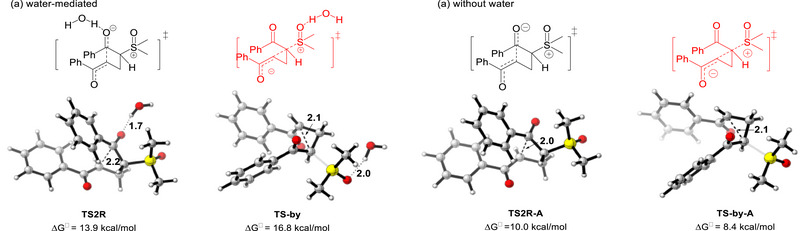
Structures of transition states with water (**TS2R** and **TS‐by**) and without water (**TS2R‐A** and **TS‐by‐A**). Bond lengths are in angstroms.

To further validate the proposed reaction mechanism, density functional theory (DFT) calculations were performed (Figure S3, Supporting Information). The computational results reveals that water‐assisted formation of the cyclobutane intermediate is energetically favored over cyclopropane product formation. The first Michael addition via the transition state **TS1** with an activation barrier of 20.8 kcal mol^−1^ is the rate‐determining step. Subsequently, the intramolecular nucleophilic addition to the carbonyl group of the benzoyl group generates the four‐membered ring cyclic intermediate **int3**, through a low activation barrier of 13.9 kcal mol^−1^. Notably, a competing pathway involving a conventional three‐membered ring transition state **TS2R** produces the by‐product, with a higher activation barrier of 16.8 kcal mol^−1^ (**Scheme** **4**). The preferred cyclobutene pathway can be rationalized by stronger hydrogen‐bonding interactions in the transition state, as supported by the shorter distance between water molecule and carbonyl oxygen (1.7 Å). Intriguingly, Without water, the activation barrier becomes higher for the cyclobutane pathway than for the cyclopropane route (10.0 vs 8.4 kcal mol^−1^). leading to reversed selectivity that aligns with conventional reaction outcomes. These findings demonstrate that hydrogen‐bonding interactions serve as critical determinants in controlling the reaction.

**Scheme 5:**
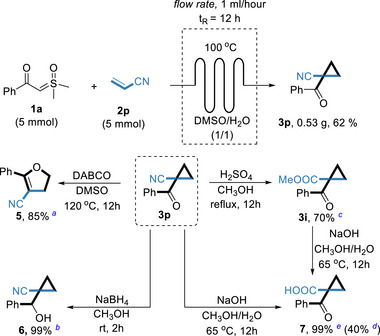
Gram‐Scale Reaction in Continuous‐Flow and Functionalizationa: Reaction conditions: *^a)^* In a 10 mL reaction tube, **3p** (0.2 mmol), DABCO (0.1 mmol), DMSO 2 mL, under N_2_ (1 atm), stirred for 12 h at 120 °C. *^b)^* In a 10 mL reaction tube, **3p** (0.2 mmol), NaBH_4_ (0.6 mmol), MeOH 2 mL, under Air (1 atm), stirred for 2 h at room temperature. *^c)^* In a 10 mL reaction tube, **3p** (0.2 mmol), H_2_SO_4_ (0.5 mL), MeOH 2 mL, under Air (1 atm), refluxed for 12 h. *^d)^* In a 10 mL reaction tube, **3p** (0.2 mmol), NaOH (0.6 mmol), MeOH/H_2_O (3/1) 2 mL, under Air (1 atm), stirred for 12 h at 65 °C. *^e)^* In a 10 mL reaction tube, **3i** (0.2 mmol), NaOH (0.6 mmol), MeOH/H_2_O (3/1) 2 mL, under Air (1 atm), stirred for 12 h at 65 °C.

In order to further demonstrate the synthetic potential of this method, we performed a gram‐scale flow synthesis of 1‐benzoylcyclopropane‐1‐carbonitrile **3p** with the utilization of sulfoxonium ylide (**1a**) and Acrylonitrile (**2p**), as shown in **Scheme** **5**.^[^ ^14^ ^]^ By applying a flow rate of 1 mL h^−1^ and a residence time as short as 12 hours, we were able to scale up the reaction process by 25‐fold. The collected reaction solution, after workup and purification, afforded the corresponding cyclopropyl ketone product **3p** in acceptable yield (62%). The subsequent conversion of 1‐benzoylcyclopropane‐1‐carbonitrile, the organocatalytic Cloke–Wilson rearrangement of cyclopropyl ketones **3p** to 2,3‐dihydrofurans was exploited utilizing the homoconjugate addition process; selective reduction of **3p** with NaBH_4_ afforded *β*‐hydroxynitrile **6**; the acid‐promoted alcoholysis of **3p** in methanol gave cyclopropyl ketones **3i** and the hydrolysis of **3p** gave carboxylic acid **7**, all proceeded smoothly, which further proved the potential synthetic value of this protocol.

## 3 Conclusion

In summary, we demonstrated a water‐catalytic deconstructive and proton transfer cyclopropanation of sulfoxonium ylide with olefin. The reaction is performed with the utilization of sulfoxonium ylides and olefins as starting materials under simple and mild reaction conditions, delivering 1,1‐disubstituted cyclopropyl ketones in 57–90% yields. Control experiments and DFT studies provided a plausible reaction mechanism of response in which H_2_O was involved in proton transfer. Moreover, this method was suitable for continuous‐flow synthesis, which provides an entry point for the industrial application.

## Conflict of Interest

The authors declare no conflict of interest.

## Supporting information



Supporting Information

## Data Availability

The data that support the findings of this study are available in the supplementary material of this article.
