# Supplementary material for: Water‐Catalytic Deconstructive and Proton Transfer Cyclopropanation of Sulfoxonium Ylide with Olefin
Source: Adv Sci (Weinh). 2025 May 30;12(31):e02430. doi: 10.1002/advs.202502430 (PMC12376613; doi:10.1002/advs.202502430)
Supplement: Supplementary file 1 — Supporting Information [file ADVS-12-e02430-s001.docx]

**Support Information**

**Water-Catalytic Deconstructive and Proton Transfer Cyclopropanation of Sulfoxonium Ylides with olefin**

Xianglin Yu,^a†^ Liuting Huang,^b†^ Haiyue Yang,^a†^ Lijuan Song ^b*^ and Yi Jin ^a*^

**^a^** Key Laboratory of Medicinal Chemistry for Natural Resource, Ministry of Education; Yunnan Provincial Center for Research & Development of Natural Products; School of Chemical Science and Technology, Yunnan University, Kunming, 650091, P. R. China.

^b^ School of Science, Harbin Institute of Technology (Shenzhen), Shenzhen 518055, China.

٭ Corresponding author. E-mail: [Lijuan Song@hit.edu.cn](mailto:Lijuan%20Song@hit.edu.cn)(*L*. Song); [jinyi@ynu.edu.cn](mailto:jinyi@ynu.edu.cn) (*Y*. Jin).

^†^ These authors contributed equallyto this work.

Contents

[1. General information S2](#_Toc85136248)

[2. General procedure for preparing **1- 7** S2](#_Toc85136249)

[3. General procedure for the gram-scale flow reaction S5](#_Toc85136250)

[Figure S1. The reaction equipment S6](#_Toc85136252)

[4. Optimization of reaction conditions S7](#_Toc85136250)

[5. KIE experiments S9](#_Toc85136250)

[6. Spectroscopic data of **3-7**  S12](#_Toc85136250)

[7. X-ray structure and data of **3d** S28](#_Toc85136251)

[Figure S2. X-Ray crystal structure of 3d S28](#_Toc85136252)

[Table S1. Crystal data and structure refinement for 3d S29](#_Toc85136253)

[8. References and notes S30](#_Toc85136248)

[9. ^1^H NMR and ^13^C NMR spectra of these compounds S31](#_Toc85136257)

[10. DFT Studies S7](#_Toc85136257)6

[Figure S3. Gibbs free energy profiles for water catalyzed cyclopropanation S7](#_Toc85136252)7

[Figure S4. Gibbs free energy profiles for cyclopropanation without water S7](#_Toc85136252)8

[Energies S7](#_Toc85136257)9

[Cartesian Coordinates S80](#_Toc85136252)

# General information

All chemicals and reagents were used of commercial grade and were used without further purification. The reactions were monitored by thin-layer chromatography (TLC) using silica gel GF254. Column chromatography was performed with 200–300 mesh silica gel. All yields refer to isolated products after purification. The intermediates and the products synthesized were fully characterized by spectroscopic data. The NMR spectra were recorded on Bruker DRX-600 (^1^H: 600 MHz, 500 MHz & 400 MHz ^13^C: 151 MHz, 126 MHz & 101 MHz) using CDCl_3,_ Acetone-*d*_6_ and DMSO-*d*_6_ as solvents. The following abbreviation were used to explain the multiplicities: (s) = singlet, (d) = doublet, (t) = triplet, (q) = quartet, (sept) = septuplet, (dd) = double doublet, (dt) = double triplet, (dq) = double quartet, (ddd) = double-double doublet, (m) = multiplet; Chemical shifts (δ) are expressed in parts per million (ppm) and J values are given in hertz (Hz). IR spectra were recorded on an FT-IR Thermo Nicolet Avatar 360 using a KBr pellet. HRMS was performed on an Agilent LC/MSD TOF instrument. The melting points were measured by the XT-4A melting point apparatus without correction.

# General Procedure for preparing **1- 7**

To a stirred solution of potassium tert-butoxide (1.0 g, 9.1 mmol) in THF (10 mL) was added trimethylsulfoxonium iodide (1.5 g, 6.9 mmol) at room temperature. The resulting mixture was refluxed for 2 h. Then the reaction mixture was cooled to 0 ^o^C, followed by addition of acylchlorides (2.3 mmol) in THF (2 mL). The reaction allowed to warm to room temperature and stirred for 3 h. After the solvent was evaporated, water (20 mL) and ethyl acetate (20 mL) were added to the residual crude product. The aqueous layer was separated and washed with ethyl acetate (3 × 20 mL) and the organic layers were combined. The organic solution was dried over anhydrous Na_2_SO_4_ and evaporated under vacuum. The residue was purified by column chromatography on silica gel to afford products **1**^1^.

Under air atmosphere, sulfoxonium ylides **1** (0.2 mmol), alkene **2** (0.2 mmol), H_2_O (0.5 ml) were added to 5 mL reaction tube. The mixture was stirred at 100 °C in oil bath for 12 h. After cooling to room temperature, the reaction was quenched with saturated NaCl solution and extracted with 10 mL EtOAc for three times. The organic layers were combined, dried over Na_2_SO_4_, filtered and evaporated under reduced pressure. The residues were purified by flash column chromatography on silica gel to provide the products **3a-4n**. The products were further identified by FTIR spectroscopy, NMR spectroscopy, and HRMS.

2,3-dihydrofuran **5** were prepared according to literatures by one the following methods ^2^. Under N_2_ atmosphere, to a solution of cyclopropyl ketones **3p** (0.2 mmol) in DMSO (2.0 mL) was added DABCO (0.1 mmol), and the mixture was stirred at 120 ºC for 15 hours. The reaction mixture was poured into water (10 mL) and then was extracted with ethyl acetate (3 × 10 mL). The combined organic phase was dried over Na_2_SO_4_, filtered and evaporated under reduced pressure. The residue was purified by silica gel chromatography (petroleum ether/ethyl acetate = 20/1 to 5/1) to afford products **5** (85% yield).

Under air atmosphere, to a solution of cyclopropyl ketones **3p** (0.2 mmol) in CH_3_OH (2.0 mL) was added NaBH_4_ (0.6 mmol), and the mixture was stirred at room temperature for 2 hours. The reaction mixture was poured into saturated NH_4_Cl water (10 mL) and then was extracted with ethyl acetate (3 × 10 mL). The combined organic phase was dried over Na_2_SO_4_, filtered and evaporated under reduced pressure to afford products **6** (99% yield).

Under air atmosphere, to a solution of cyclopropyl ketones **3p** (0.2 mmol) in CH_3_OH (2.0 mL) was added H_2_SO_4_ (0.5 mL), and the mixture was refluxed for 12 hours. The reaction mixture was poured into saturated Na_2_CO_3_ water (10 mL) and then was extracted with ethyl acetate (3 × 10 mL). The combined organic phase was dried over Na_2_SO_4_, filtered and evaporated under reduced pressure. The residue was purified by silica gel chromatography (petroleum ether/ethyl acetate = 50/1) to afford products **3i** (70% yield).

Under air atmosphere, to a solution of cyclopropyl ketones **3i** (0.2 mmol) in CH_3_OH /H_2_O (3/1, 2.0 mL) was added NaOH (0.6 mmol), and the mixture was stirred at 65 ºC for 12 hours. Added hydrochloric acid to the mixture to weakly acid. The reaction mixture was poured into water (10 mL) and then was extracted with ethyl acetate (3 × 10 mL). The combined organic phase was dried over Na_2_SO_4_, filtered and evaporated under reduced pressure to afford products **7** (99% yield).

Under air atmosphere, to a solution of cyclopropyl ketones **3p** (0.2 mmol) in CH_3_OH /H_2_O (3/1, 2.0 mL) was added NaOH (0.6 mmol), and the mixture was stirred at 65 ºC for 12 hours. Added hydrochloric acid to the mixture to weakly acid. The reaction mixture was poured into water (10 mL) and then was extracted with ethyl acetate (3 × 10 mL). The combined organic phase was dried over Na_2_SO_4_, filtered and evaporated under reduced pressure. The residue was purified by silica gel chromatography (ethyl acetate/MeOH = 2/1) to afford products **7** (40% yield).

# General procedure for the gram-scale flow reaction

An oven dried 250 mL erlenmeyer flask was charged with a stir bar. Substrate **1a** (1.0 g, 5.0 mmol, 1.0 eq), **2p** (265 mg, 5.0 mmol, 1.0 eq) was added with 12.5 mL DMSO and 12.5 mL H_2_O. The reaction mixture was stirred and heated with commercially available magnetic stirrer and flow pump for 12 hours. After reaction, the solvent was removed by rotary evaporation. Purified by flash column chromatography on silica gel (petroleum ether: ethyl acetate = 20:1) afforded cyclopropyl ketone product **3o** (0.53g, 62% yield) as a colorless oil.


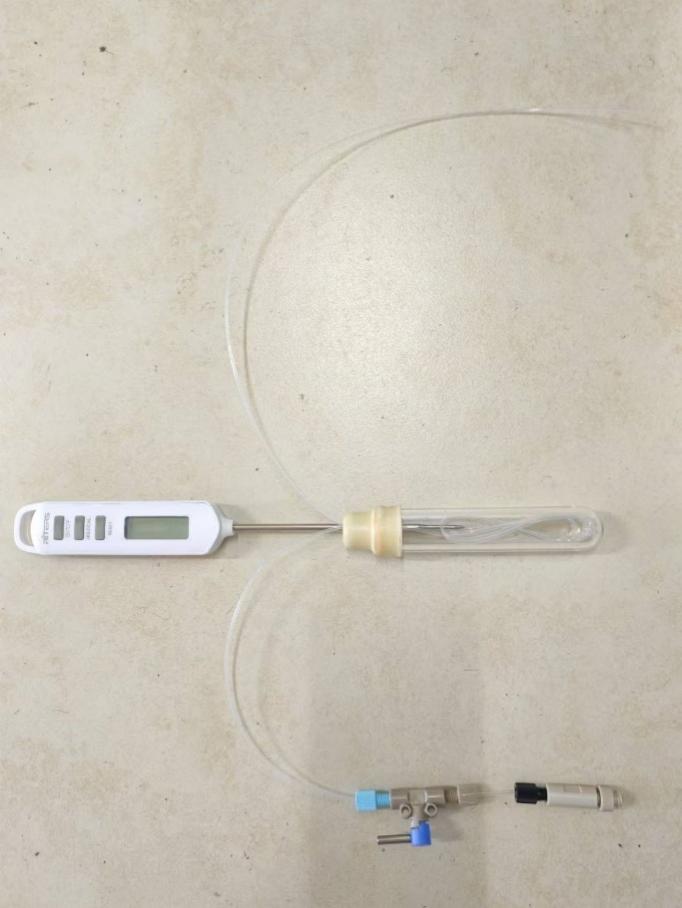


**Throttle Valve**

**Reactor (Silicone oil)**

**thermometer**


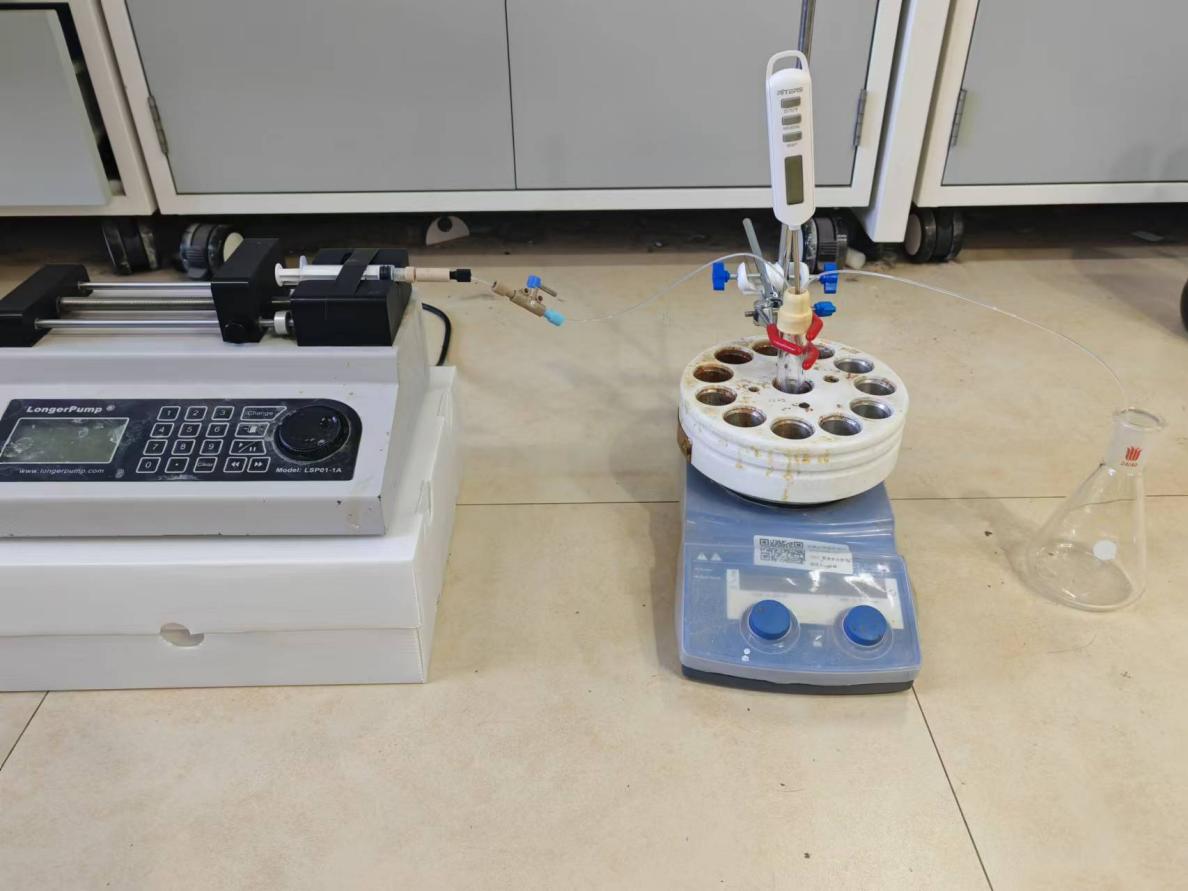


Figure S1. The reaction equipment

# Optimization of reaction conditions

Table S1. Screening of solvents

| Entry*^a^* | Solvent | H_2_O (X equiv.) | Yield (%) | |
| --- | --- | --- | --- | --- |
|  |  |  | **3a** | **3a’** |
| 1 | DMSO | 10 | 50 | 40 |
| 2 | DMSO | 100 | 60 | 37 |
| 3 | DMSO/ H_2_O (4/1) | --- | 65 | 26 |
| 4 | DMSO/ H_2_O (1/1) | --- | 78 | 15 |
| 5 | DMSO/ H_2_O (2/3) | --- | 73 | 15 |
| 6 | DMSO/ H_2_O (1/3) | --- | 65 | 18 |
| 7 | DMSO/ H_2_O (1/4) | --- | 62 | 20 |
| 8 | DMSO/ H_2_O (1/1, 0.5 mL) | --- | 68 | 18 |
| 9 | DMF/ H_2_O (1/1) | --- | 63 | 35 |
| 10 | PhCH_3_/ H_2_O (1/1) | --- | 35 | 25 |
| 11 | DCE/ H_2_O (1/1) | --- | 20 | 28 |
| 12 | CH_3_CN/ H_2_O (1/1) | --- | 30 | 25 |
| 13 | DMSO | Trace | nd | 40 |
| 14 | H_2_O | --- | 64 | 20 |
| **15** | **H_2_O (0.5 mL)** | **---** | **75** | **20** |
| 16 | H_2_O (0.2 mL) | --- | 70 | 20 |
| 17*^c^* | H_2_O (0.5 mL) | --- | 73 | 20 |

[a] Reaction conditions: In a 5 mL reaction tube, sulfoxonium ylide **1a** (0.2 mmol), olefin **2a** (0.2 mmol), solvent 1 mL, under air (1 atm), stirred for 12 h at 100 ^o^C. [b] Yields are of isolated products after chromatographic purification based on **1a**. [c] Addition of sulfoxonium ylide **1a** (0.24 mmol).

Table S2. Screening of temperatures

| Entry*^a^* | *T* ^o^C | Yield (%) | |
| --- | --- | --- | --- |
|  |  | **3a** | **3a’** |
| 1 | 100 | 75 | 20 |
| 2 | 110 | 73 | 20 |
| 3 | 120 | 70 | 16 |
| 4 | 90 | 70 | 20 |
| 5 | 80 | 64 | 16 |
| 6 | 70 | 50 | 15 |
| 7 | 60 | 42 | 10 |
| 8 | 25 | nd | nd |

[a] Reaction conditions: In a 5 mL reaction tube, sulfoxonium ylide **1a** (0.2 mmol), olefin **2a** (0.2 mmol), H_2_O 0.5 mL, under air (1 atm), stirred for 12 h at *T* ^o^C. [b] Yields are of isolated products after chromatographic purification based on **1a**.

Table S3. Screening of additions

| Entry*^a^* | addition | Yield (%) | |
| --- | --- | --- | --- |
|  |  | **3a** | **3a’** |
| 1 | NaH (10.0 equiv) | nd | nd |
| 2 | CH_3_COOH (10.0 equiv) | 30 | 45 |
| 3 | TsOH (10.0 equiv) | nd | nd |
| 4 | CF_3_COOH (10.0 equiv) | nd | nd |
| 5 | EtOH (10.0 equiv) | 15 | 40 |

[a] Reaction conditions: In a 5 mL reaction tube, sulfoxonium ylide **1a** (0.2 mmol), olefin **2a** (0.2 mmol), DMSO 1 mL, under air (1 atm), stirred for 12 h at 100 ^o^C. [b] Yields are of isolated products after chromatographic purification based on **1a**.

# KIE experiments

Competitive KIE experiment

Under air atmosphere, alkene **1a** (0.2 mmol), sulfoxonium ylides **2a** (0.2 mmol), H_2_O/D_2_O (1:1, 0.5 ml) were added to 5 mL reaction tube. The mixture was stirred at 100 °C in oil bath for 4 h. After cooling to room temperature, the reaction was quenched with saturated NaCl solution and extracted with 10 mL EtOAc for three times. The organic layers were combined, dried over Na_2_SO_4_, filtered and evaporated under reduced pressure. After the reaction was completed, it was purified by silica gel, and eluted with PE : EA = 20 : 1 to obtain the mixture·of **3a** and **3a-*d_2_***, 35% yield. It can be seen from the ^1^H NMR of the mixture that **3a**: **3a-*d_2_*** = 1.2 : 0.8 and **1a**: **1a-*d_1_*** = 0.66 : 0.33.

The Competitive KIE = *K*_H/D_ = (1.2 - 0.66)/(0.8 - 0.33) = 1.15

When the ratio of rate constants satisfies 1.0< *K*_H/D_ <1.5, it is classified as a normal secondary isotope effect. This phenomenon typically occurs when the carbon atom bonded to the isotopic substituent undergoes hybridization change from *sp³* to *sp²* during the reaction.


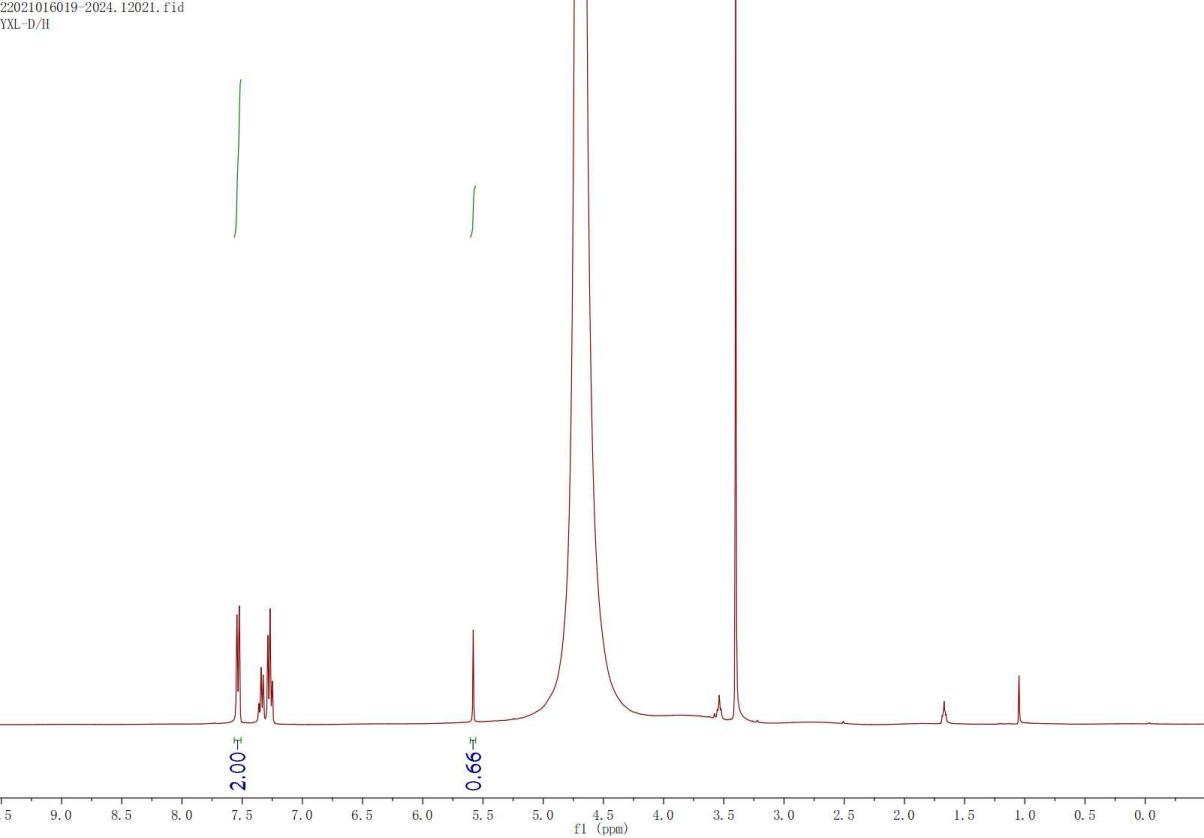

Parallel KIE experiment

Under air atmosphere, alkene **1a** (0.2 mmol), sulfoxonium ylides **2a** (0.2 mmol), H_2_O (0.5 ml) were added to 5 mL reaction tube. The mixture was stirred at 100 °C in oil bath for 3 h, 6 h, 9 h, 12 h. After the reaction was completed, it was purified by silica gel to obtain **3a** in 35.5%, 52.4%, 66.1% and 77.6% yields, respectively.

Under the same condition, **1a**, **2a** and D_2_O (0.5 ml) were added to the other reaction flask, and the products **3a-*d_2_*** was obtained in 32.1%, 53.2%, 61.2% and 70.2% yields, respectively.

The Parallel KIE = *K*_H/D_ = 0.0467/0.0408 = 1.14

The *K*_H/D_ of primary kinetic isotope effect ≈ 6.5. The Parallel KIE = *K*_H/D_ = 1.14, it is classified as a normal secondary isotope effect, and this demonstrates that the step does not serve as the rate-determining step of the reaction.

# Spectroscopic data of **3**-**5** & **3a-*d***

Spectroscopic Data of **3a**

benzyl 1-benzoylcyclopropane-1-carboxylate

75% yield; Colorless liquid

**IR** (KBr): 3669, 3457, 3069, 3039, 2966, 1965, 1734, 1686, 1596, 1454, 1382, 1320, 1216, 1167, 1083, 1011, 893, 792, 704;

^1^H NMR (500 MHz, Chloroform-*d*) δ 7.86 (d, *J* = 7.3 Hz, 2H, PhH), 7.53 (t, *J* = 7.3 Hz, 1H, PhH), 7.39 (t, *J* = 7.5 Hz, 2H, PhH), 7.20 (dt, *J* = 14.3, 7.1 Hz, 3H, PhH), 6.89 (d, *J* = 7.0 Hz, 2H, PhH), 5.01 (s, 2H, CH_2_), 1.64 (s, 2H, CH_2_), 1.56 (s, 2H, CH_2_).

^13^C NMR (126 MHz, Chloroform-*d*) δ 194.78, 171.58, 137.14, 135.08, 132.82, 128.51, 128.43, 128.34, 128.08, 127.87, 67.12, 33.06, 16.12.

**HRMS** (TOF-ESI^+^): *m/z* calcd for C_18_H_16_O_3_ [M+H] ^+^, 281.1172; found, 281.1173.

Spectroscopic Data of **3b**

4-fluorobenzyl 1-benzoylcyclopropane-1-carboxylate

82% yield; Colorless liquid

**IR** (KBr): 3955, 3784, 3567, 3452, 3073, 2963, 1732, 1685, 1607, 1514, 1457, 1379, 1322, 1222, 1156, 1006, 837, 782, 716;

^1^H NMR (400 MHz, Acetone-*d*_6_) δ 7.72 (d, *J* = 7.3 Hz, 2H, PhH), 7.48 (t, *J* = 7.4 Hz, 1H, PhH), 7.34 (t, *J* = 7.8 Hz, 2H, PhH), 6.90 – 6.80 (m, 4H, PhH), 4.87 (s, 2H, CH_2_), 1.47 – 1.41 (m, 2H, CH_2_), 1.40 – 1.35 (m, 2H, CH_2_).

^13^C NMR (101 MHz, Acetone-*d*_6_) δ 193.9, 171.0, 162.4 (*J* = 240.0 Hz), 137.2, 132.8, 131.8, 130.1 (*J* = 8.0 Hz), 128.6, 128.2, 115.0 (*J* = 20.0 Hz), 65.9, 32.6, 15.1.

^19^F NMR (376 MHz, Acetone-*d*_6_) δ -115.55.

**HRMS** (TOF-ESI^+^): *m/z* calcd for C_18_H_15_FO_3_ [M+H] ^+^, 299.1078; found, 299.1080.

Spectroscopic Data of **3c**

4-chlorobenzyl 1-benzoylcyclopropane-1-carboxylate

85% yield; White solid; Mp: 81.5 – 83.2 ^o^C

**IR** (KBr): 3788, 3456, 3066, 2960, 2901, 1736, 1685, 1595, 1494, 1455, 1377, 1319, 1215, 1155, 1089, 1009, 803, 715;

^1^H NMR (400 MHz, Acetone-*d*_6_) δ 7.73 (d, *J* = 7.1 Hz, 2H, PhH), 7.49 (t, *J* = 7.4 Hz, 1H, PhH), 7.35 (t, *J* = 7.7 Hz, 2H, PhH), 7.10 (d, *J* = 8.5 Hz, 2H, PhH), 6.82 (d, *J* = 8.5 Hz, 2H, PhH), 4.89 (s, 2H, CH_2_), 1.48 – 1.42 (m, 2H, CH_2_), 1.42 – 1.37 (m, 2H, CH_2_).

^13^C NMR (101 MHz, Acetone-*d*_6_) δ 193.9, 170.9, 137.2, 134.6, 133.3, 132.9, 129.5, 128.6, 128.3, 128.2, 65.8, 32.6, 15.2.

**HRMS** (TOF-ESI^+^): *m/z* calcd for C_18_H_15_ClO_3_ [M+H] ^+^, 315.0782; found, 315.0782.

Spectroscopic Data of **3d**

4-bromobenzyl 1-benzoylcyclopropane-1-carboxylate

90% yield; White solid; Mp: 77.4 – 79.7 ^o^C

**IR** (KBr): 3884, 3796, 3664, 3364, 3065, 3041, 2963, 1733, 1684, 1599, 1491, 1453, 1376, 1321, 1157, 1073, 1010, 803, 713;

^1^H NMR (400 MHz, Acetone-*d*_6_) δ 7.73 (dd, *J* = 8.3, 1.4 Hz, 2H, PhH), 7.49 (t, *J* = 7.4 Hz, 1H, PhH), 7.35 (t, *J* = 7.8 Hz, 2H, PhH), 7.25 (d, *J* = 8.4 Hz, 2H, PhH), 6.76 (d, *J* = 8.4 Hz, 2H, PhH), 4.87 (s, 2H, CH_2_), 1.47 – 1.42 (m, 2H, CH_2_), 1.42 – 1.37 (m, 2H, CH_2_).

^13^C NMR (101 MHz, Acetone-*d*_6_) δ 193.9, 170.9, 137.2, 135.0, 132.9, 131.3, 129.8, 128.6, 128.2, 121.5, 65.8, 32.6, 15.2.

**HRMS** (TOF-ESI^+^): *m/z* calcd for C_18_H_15_BrO_3_ [M+H] ^+^, 359.0277; found, 359.0275.

Spectroscopic Data of **3e**

4-methylbenzyl 1-benzoylcyclopropane-1-carboxylate

65% yield; Colorless liquid

**IR** (KBr): 3959, 3771, 3665, 3448, 3064, 3022, 2961, 1743, 1684, 1606, 1521, 1452, 1379, 1336, 1215, 1168, 1005, 799, 714;

^1^H NMR (400 MHz, Acetone-*d*_6_) δ 7.76 – 7.70 (m, 2H, PhH), 7.48 (t, *J* = 7.4 Hz, 1H, PhH), 7.34 (t, *J* = 7.8 Hz, 2H, PhH), 6.89 (d, *J* = 7.9 Hz, 2H, PhH), 6.72 (d, *J* = 7.9 Hz, 2H, PhH), 4.84 (s, 2H, CH_2_), 2.13 (s, 3H, CH_3_), 1.43 (td, *J* = 7.0, 6.1, 2.7 Hz, 2H, CH_2_), 1.36 (td, *J* = 6.1, 5.1, 2.7 Hz, 2H, CH_2_).

^13^C NMR (101 MHz, Acetone-*d*_6_) δ 193.9, 171.0, 137.6, 137.1, 132.8, 132.6, 128.9, 128.6, 128.3, 127.9, 66.6, 32.7, 20.2, 15.0.

**HRMS** (TOF-ESI^+^): *m/z* calcd for C_19_H_18_O_3_ [M+H] ^+^, 295.1329; found, 295.1325.

Spectroscopic Data of **3f**

4-methoxybenzyl 1-benzoylcyclopropane-1-carboxylate

76% yield; Colorless liquid

**IR** (KBr): 3631, 3440, 3351, 3013, 2966, 2846, 1740, 1692, 1611, 1517, 1456, 1379, 1301, 1253, 1216, 1182, 1006, 883, 754, 716;

^1^H NMR (400 MHz, Acetone-*d*_6_) δ 7.71 (d, *J* = 7.3 Hz, 2H, PhH), 7.48 (t, *J* = 7.4 Hz, 1H, PhH), 7.34 (t, *J* = 7.7 Hz, 2H, PhH), 6.79 (d, *J* = 8.6 Hz, 2H, PhH), 6.63 (d, *J* = 8.6 Hz, 2H, PhH), 4.81 (s, 2H, CH_2_), 3.63 (s, 3H, CH_3_), 1.42 (td, *J* = 6.2, 5.7, 2.3 Hz, 2H, CH_2_), 1.35 (td, *J* = 5.8, 5.3, 2.4 Hz, 2H, CH_2_).

^13^C NMR (101 MHz, Acetone-*d*_6_) δ 193.9, 171.0, 159.7, 137.1, 132.8, 129.7, 128.6, 128.2, 127.5, 113.6, 66.5, 54.6, 32.7, 15.0.

**HRMS** (TOF-ESI^+^): *m/z* calcd for C_19_H_18_O_4_ [M+H] ^+^, 311.1278; found, 311.1277.

Spectroscopic Data of **3g**

phenyl 1-benzoylcyclopropane-1-carboxylate

80% yield; Colorless liquid

**IR** (KBr): 3842, 3781, 3479, 2971, 1751, 1686, 1600, 1310, 1213, 1132, 914, 780, 724;

^1^H NMR (400 MHz, Acetone-*d*_6_) δ 7.91 (d, *J* = 7.4 Hz, 2H, PhH), 7.55 (t, *J* = 7.3 Hz, 1H, PhH), 7.47 (t, *J* = 7.5 Hz, 2H, PhH), 7.19 (t, *J* = 7.8 Hz, 2H, PhH), 7.07 (t, *J* = 7.4 Hz, 1H, PhH), 6.72 (d, *J* = 8.1 Hz, 2H, PhH), 1.62 (q, *J* = 4.3, 3.3 Hz, 2H, CH_2_), 1.54 (q, *J* = 3.2 Hz, 2H, CH_2_).

^13^C NMR (101 MHz, Acetone-*d*_6_) δ 193.6, 169.9, 150.6, 137.2, 133.0, 129.3, 128.8, 128.3, 126.0, 121.2, 32.7, 15.7.

**HRMS** (TOF-ESI^+^): *m/z* calcd for C_17_H_14_O_3_ [M+Na] ^+^, 289.0835; found, 289.0837.

Spectroscopic Data of **3h**

phenethyl 1-benzoylcyclopropane-1-carboxylate

76% yield; Colorless liquid

**IR** (KBr): 3969, 3803, 3607, 3554, 3464, 3070, 3035, 2965, 1732, 1685, 1595, 1455, 1389, 1323, 1216, 1168, 1003, 924, 783, 748, 715, 662;

^1^H NMR (400 MHz, Acetone-*d*_6_) δ 7.88 (d, *J* = 7.9 Hz, 2H, PhH), 7.62 (t, *J* = 7.3 Hz, 1H, PhH), 7.51 (t, *J* = 7.6 Hz, 2H, PhH), 7.20 (dq, *J* = 14.1, 6.9 Hz, 3H, PhH), 7.04 (d, *J* = 7.2 Hz, 2H, PhH), 4.20 (t, *J* = 6.8 Hz, 2H, CH_2_), 2.67 (t, *J* = 6.8 Hz, 2H, CH_2_), 1.52 (q, *J* = 4.0, 3.1 Hz, 2H, CH_2_), 1.45 (q, *J* = 5.1, 4.1 Hz, 2H, CH_2_).

^13^C NMR (101 MHz, Acetone-*d*_6_) δ 193.9, 171.1, 137.8, 137.0, 132.9, 128.7, 128.5, 128.3, 128.3, 126.3, 65.5, 34.4, 32.6, 14.9.

**HRMS** (TOF-ESI^+^): *m/z* calcd for C_19_H_18_O_3_ [M+H] ^+^, 295.1329; found, 295.1328.

Spectroscopic Data of **3i**

methyl 1-benzoylcyclopropane-1-carboxylate

73% yield; Colorless liquid

**IR** (KBr): 3483, 3362, 2973, 1737, 1687, 1437, 1315, 1227, 1150, 915, 789, 723;

^1^H NMR (400 MHz, Chloroform-*d*) δ 7.91 (d, *J* = 7.1 Hz, 2H, PhH), 7.56 (t, *J* = 7.4 Hz, 1H, PhH), 7.45 (t, *J* = 7.6 Hz, 2H, PhH), 3.58 (s, 3H, CH_3_), 1.64 – 1.60 (m, 2H, CH_2_), 1.54 – 1.50 (m, 2H, CH_2_).

^13^C NMR (101 MHz, Chloroform-*d*) δ 194.6, 172.3, 136.8, 133.0, 128.5, 52.5, 32.8, 16.0.

**HRMS** (TOF-ESI^+^): *m/z* calcd for C_12_H_12_O_3_ [M+H] ^+^, 205.0859; found, 205.0858.

Spectroscopic Data of **3j**

ethyl 1-benzoylcyclopropane-1-carboxylate

75% yield; Colorless liquid

**IR** (KBr): 3770, 3652, 3440, 2985, 1732, 1688, 1318, 1176, 1094, 1010, 753;

^1^H NMR (600 MHz, Chloroform-*d*) δ 7.89 (d, *J* = 7.4 Hz, 2H, PhH), 7.54 (t, *J* = 7.3 Hz, 1H, PhH), 7.44 (t, *J* = 7.5 Hz, 2H, PhH), 4.03 (q, *J* = 7.1 Hz, 2H, CH_2_), 1.60 (s, 2H, CH_2_), 1.52 (s, 2H, CH_2_), 0.94 (t, *J* = 7.1 Hz, 3H, CH_3_).

^13^C NMR (151 MHz, Chloroform-*d*) δ 194.93, 171.64, 137.15, 132.70, 128.31, 61.26, 32.99, 15.78, 13.61.

**HRMS** (TOF-ESI^+^): *m/z* calcd for C_13_H_14_O_3_ [M+H] ^+^, 219.1016; found, 219.1020.

Spectroscopic Data of **3k**

isopropyl 1-benzoylcyclopropane-1-carboxylate

78% yield; Colorless liquid

**IR** (KBr): 3940, 3877, 3798, 3699, 3389, 3080, 2977, 2376, 1728, 1689, 1457, 1324, 1273, 1098, 1030, 840, 797, 711;

^1^H NMR (600 MHz, Chloroform-*d*) δ 7.88 (d, *J* = 7.4 Hz, 2H, PhH), 7.54 (t, *J* = 7.4 Hz, 1H, PhH), 7.44 (t, *J* = 7.7 Hz, 2H, PhH), 4.91 (hept, *J* = 6.3 Hz, 1H, CH), 1.59 (q, *J* = 4.1, 3.5 Hz, 2H, CH_2_), 1.52 (q, *J* = 4.8, 4.1 Hz, 2H, CH_2_), 0.93 (d, *J* = 6.3 Hz, 6H, CH_3_).

^13^C NMR (151 MHz, Chloroform-*d*) δ 194.4, 170.2, 136.5, 131.6, 127.3, 127.3, 68.0, 32.3, 20.2, 14.7.

**HRMS** (TOF-ESI^+^): *m/z* calcd for C_14_H_16_O_3_ [M+H] ^+^, 233.1172; found, 233.1170.

Spectroscopic Data of **3l**

isobutyl 1-benzoylcyclopropane-1-carboxylate

75% yield; Colorless liquid

**IR** (KBr): 3637, 3084, 2966, 2885, 1732, 1686, 1595, 1461, 1379, 1323, 1215, 1165, 1003, 930, 883, 714;

^1^H NMR (400 MHz, Chloroform-*d*) δ 7.91 (d, *J* = 7.1 Hz, 2H, PhH), 7.54 (t, *J* = 7.4 Hz, 1H, PhH), 7.44 (t, *J* = 7.6 Hz, 2H, PhH), 3.75 (d, *J* = 6.4 Hz, 2H, CH_2_), 1.66 – 1.59 (m, 3H, CH&CH_2_), 1.55 – 1.51 (m, 2H, CH_2_), 0.57 (d, *J* = 6.7 Hz, 6H, CH_3_).

^13^C NMR (101 MHz, Chloroform-*d*) δ 195.0, 171.9, 137.2, 132.9, 128.5, 128.4, 71.7, 33.0, 27.4, 18.6, 15.9.

**HRMS** (TOF-ESI^+^): *m/z* calcd for C_15_H_18_O_3_ [M+H] ^+^, 247.1329; found, 247.1330.

Spectroscopic Data of **3m**

butyl 1-benzoylcyclopropane-1-carboxylate

76% yield; Colorless liquid

**IR** (KBr): 3788, 3665, 3578, 3405, 3070, 2966, 2884, 1732, 1685, 1595, 1457, 1391, 1324, 1268, 1216, 1167, 1011, 923, 793, 712;

^1^H NMR (400 MHz, Chloroform-*d*) δ 7.90 (d, *J* = 7.4 Hz, 2H, PhH), 7.54 (t, *J* = 7.4 Hz, 1H, PhH), 7.44 (t, *J* = 7.5 Hz, 2H, PhH), 3.97 (t, *J* = 6.4 Hz, 2H, CH_2_), 1.60 (d, *J* = 3.1 Hz, 2H, CH_2_), 1.56 – 1.50 (m, 2H, CH_2_), 1.29 (dt, *J* = 14.5, 6.8 Hz, 3H, CH_2_), 0.92 (dq, *J* = 15.4, 7.6 Hz, 2H, CH_2_), 0.69 (t, *J* = 7.3 Hz, 3H, CH_3_).

^13^C NMR (101 MHz, Chloroform-*d*) δ 194.0, 170.9, 136.2, 131.8, 127.4, 127.3, 64.2, 32.0, 29.2, 17.6, 14.9, 12.4.

**HRMS** (TOF-ESI^+^): *m/z* calcd for C_15_H_18_O_3_ [M+H] ^+^, 247.1329; found, 247.1329.

Spectroscopic Data of **3n**

1-benzoyl-*N*,*N*-dimethylcyclopropane-1-carboxamide

57% yield; Colorless liquid

**IR** (KBr): 3772, 3674, 3344, 2986, 2728, 1722, 1641, 1294, 1115, 801, 723;

^1^H NMR (400 MHz, Chloroform-*d*) δ 7.98 (d, *J* = 7.8 Hz, 2H, PhH), 7.55 (t, *J* = 7.3 Hz, 1H, PhH), 7.43 (t, *J* = 7.6 Hz, 2H, PhH), 2.78 (d, *J* = 4.4 Hz, 6H, CH_3_), 1.58 (d, *J* = 7.8 Hz, 2H, CH_2_), 1.52 (d, *J* = 7.9 Hz, 2H, CH_2_).

^13^C NMR (101 MHz, Chloroform-*d*) δ 196.6, 169.2, 135.9, 132.1, 127.5, 36.0, 34.9, 34.7, 14.8.

**HRMS** (TOF-ESI^+^): *m/z* calcd for C_13_H_15_NO_2_ [M+H] ^+^, 218.1176; found, 218.1174.

Spectroscopic Data of **3o**

cyclopropane-1,1-diylbis(phenylmethanone)

80% yield; White solid

**IR** (KBr): 3927, 3728, 3471, 3337, 3070, 1669, 1594, 1448, 1319, 1205, 1076, 1012, 934, 790, 716, 689;

^1^H NMR (400 MHz, Chloroform-*d*) δ 7.66 (d, *J* = 7.3 Hz, 4H, PhH), 7.28 (t, *J* = 7.4 Hz, 2H, PhH), 7.18 (t, *J* = 7.6 Hz, 4H, PhH), 1.69 (s, 4H, CH_2_).

^13^C NMR (101 MHz, Chloroform-*d*) δ 196.4, 136.6, 131.8, 127.5, 127.4, 39.7, 15.6.

**HRMS** (TOF-ESI^+^): *m/z* calcd for C_17_H_14_O_2_ [M+H] ^+^, 251.1067; found, 251.1067.

Spectroscopic Data of **3p**

1-benzoylcyclopropane-1-carbonitrile

73% yield; Colorless liquid

**IR** (KBr): 3324, 2939, 2247, 1684, 1438, 1305, 1003, 851, 792;

^1^H NMR (400 MHz, Chloroform-*d*) δ 8.07 – 8.01 (m, 2H, PhH), 7.63 (t, *J* = 7.4 Hz, 1H, PhH), 7.52 (t, *J* = 7.6 Hz, 2H, PhH), 1.92 – 1.87 (m, 2H, CH_2_), 1.79 – 1.75 (m, 2H, CH_2_).

^13^C NMR (101 MHz, Chloroform-*d*) δ 192.5, 135.7, 133.7, 128.8, 128.7, 121.0, 20.3, 18.2.

**HRMS** (TOF-ESI^+^): *m/z* calcd for C_11_H_9_NO [M+H] ^+^, 172.0757; found, 172.0757.

Spectroscopic Data of **3q**

phenyl(1-(phenylsulfonyl)cyclopropyl)methanone

61% yield; Yellow liquid

**IR** (KBr): 3696, 3505, 3397, 3240, 2921, 1675, 1455, 1317, 1150, 1083, 890, 753;

^1^H NMR (400 MHz, Acetone-*d*_6_) δ 7.82 (d, *J* = 7.7 Hz, 2H, PhH), 7.63 – 7.42 (m, 6H, PhH), 7.34 (t, *J* = 7.7 Hz, 2H, PhH), 1.81 – 1.76 (m, 2H, CH_2_), 1.47 – 1.42 (m, 2H, CH_2_).

^13^C NMR (101 MHz, Acetone-*d*_6_) δ 191.3, 139.2, 135.8, 133.9, 133.6, 130.0, 129.1, 128.6, 128.2, 49.0, 12.6.

**HRMS** (TOF-ESI^+^): *m/z* calcd for C_16_H_14_SO_3_ [M+H] ^+^, 287.0736; found, 287.0740.

Spectroscopic Data of **3r**

3-methylbut-2-en-1-yl 1-benzoylcyclopropane-1-carboxylate

75% yield; Yellow liquid

**IR** (KBr): 3877, 3623, 3552, 3322, 3079, 2952, 1734, 1685, 1583, 1457, 1386, 1324, 1268, 1221, 1167, 1011, 923, 783, 712;

^1^H NMR (400 MHz, Chloroform-*d*) δ 7.89 (d, *J* = 7.2 Hz, 2H, PhH), 7.54 (t, *J* = 7.4 Hz, 1H, PhH), 7.43 (t, *J* = 7.6 Hz, 2H, PhH), 4.99 (t, *J* = 7.3 Hz, 1H, CH), 4.46 (d, *J* = 7.3 Hz, 2H, CH_2_), 1.62 (s, 3H, CH_3_), 1.61 – 1.59 (m, 2H, CH_2_), 1.54 – 1.50 (m, 2H, CH_2_), 1.46 (s, 3H, CH_3_).

^13^C NMR (101 MHz, Chloroform-*d*) δ 195.0, 171.7, 139.7, 137.1, 132.7, 128.5, 128.4, 117.7, 62.1, 33.1, 25.6, 17.7, 15.8.

**HRMS** (TOF-ESI^+^): *m/z* calcd for C_16_H_18_O_3_ [M+H] ^+^, 259.1329; found, 259.1332.

Spectroscopic Data of **3s**

2-oxopropyl 1-benzoylcyclopropane-1-carboxylate

58% yield; Colorless liquid

**IR** (KBr): 3812, 3652, 3515, 3411, 3050, 2966, 1715, 1682, 1595, 1457, 1391, 1311, 1279, 1232, 1152, 923, 793, 709;

^1^H NMR (400 MHz, Chloroform-*d*) δ 7.93 (d, *J* = 7.1 Hz, 2H, PhH), 7.49 (t, *J* = 7.4 Hz, 1H, PhH), 7.40 (t, *J* = 7.5 Hz, 2H, PhH), 4.47 (s, 2H, CH_2_), 1.88 (s, 3H, CH_3_), 1.66 – 1.58 (m, 2H, CH_2_), 1.58 – 1.50 (m, 2H, CH_2_).

^13^C NMR (101 MHz, Chloroform-*d*) δ 200.0, 193.2, 170.1, 135.4, 132.2, 127.8, 127.5, 67.9, 31.6, 24.9, 15.5.

**HRMS** (TOF-ESI^+^): *m/z* calcd for C_14_H_14_O_4_ [M+H] ^+^, 247.0965; found, 247.0968.

Spectroscopic Data of **3t**

4-(cyanomethyl)phenyl 1-benzoylcyclopropane-1-carboxylate

81% yield; White solid; Mp: 95.3 – 96.8 ^o^C

**IR** (KBr): 3876, 3836, 3496, 3371, 2987, 2253, 1665, 1589, 1466, 1434, 1310, 1242, 1191, 1075, 1031, 906, 785, 744, 705, 689;

^1^H NMR (400 MHz, Chloroform-*d*) δ 8.00 (d, *J* = 7.1 Hz, 2H, PhH), 7.62 (t, *J* = 7.4 Hz, 1H, PhH), 7.52 (t, *J* = 7.6 Hz, 2H, PhH), 7.23 (d, *J* = 8.6 Hz, 2H, PhH), 6.76 (d, *J* = 8.6 Hz, 2H, PhH), 3.69 (s, 2H, CH_2_), 1.80 – 1.75 (m, 2H, CH_2_), 1.75 – 1.70 (m, 2H, CH_2_).

^13^C NMR (101 MHz, Chloroform-*d*) δ 194.3, 170.3, 150.0, 137.2, 133.2, 129.0, 128.8, 128.4, 127.7, 121.8, 33.1, 23.1, 16.9.

**HRMS** (TOF-ESI^+^): *m/z* calcd for C_19_H_15_NO_3_ [M+H] ^+^, 306.1125; found, 306.1127.

Spectroscopic Data of **3u**

(1*R*,2*S*,5*R*)-2-isopropyl-5-methylcyclohexyl 1-benzoylcyclopropane-1-carboxylate

60% yield; Colorless liquid

^1^H NMR (400 MHz, Chloroform-*d*) δ 7.84 (d, *J* = 7.6 Hz, 2H, PhH), 7.48 (t, *J* = 7.4 Hz, 1H, PhH), 7.37 (t, *J* = 7.6 Hz, 2H, PhH), 4.48 (td, *J* = 10.8, 4.3 Hz, 1H, CH), 1.72 (d, *J* = 11.5 Hz, 1H, CH), 1.64 – 1.58 (m, 1H, CH_2_), 1.54 – 1.37 (m, 5H, CH_2_), 1.00 (td, *J* = 11.6, 11.1, 5.1 Hz, 2H, CH_2_), 0.82 (qd, *J* = 12.6, 2.9 Hz, 2H, CH_2_), 0.74 (d, *J* = 6.5 Hz, 3H, CH_3_), 0.66 (td, *J* = 12.4, 3.0 Hz, 1H, CH), 0.57 (d, *J* = 11.5 Hz, 1H, CH), 0.53 (d, *J* = 6.8 Hz, 3H, CH_3_), 0.44 (d, *J* = 6.8 Hz, 3H, CH_3_).

^13^C NMR (101 MHz, Chloroform-*d*) δ 194.2, 170.2, 136.3, 131.7, 127.5, 127.3, 74.6, 45.5, 39.0, 32.9, 32.4, 30.2, 24.2, 21.8, 20.9, 19.7, 14.8, 14.6, 14.2.

**HRMS** (TOF-ESI^+^): *m/z* calcd for C_21_H_28_O_3_ [M+H] ^+^, 329.2111; found, 329.2110.

Spectroscopic Data of **3v**

(3*S*,8*S*,9*S*,10*R*,13*R*,14*S*,17*R*)-10,13-dimethyl-17-((*R*)-6-methylheptan-2-yl)-2,3,4,7,8,9,10,11,12,13,14,15,16,17-tetradecahydro-1H-cyclopenta[*a*]phenanthren-3-yl 1-benzoylcyclopropane-1-carboxylate

52% yield; White solid; Mp: 131.5 – 132.8 ^o^C

^1^H NMR (400 MHz, Chloroform-*d*) δ 7.82 (d, *J* = 7.3 Hz, 2H, PhH), 7.47 (t, *J* = 7.4 Hz, 1H, PhH), 7.37 (t, *J* = 7.6 Hz, 2H, PhH), 5.20 (s, 1H, CH), 4.44 (tt, *J* = 11.1, 4.8 Hz, 1H, CH), 1.98 – 1.61 (m, 7H, CH_2_), 1.56 – 1.51 (m, 2H, CH_2_), 1.49 – 1.40 (m, 6H, CH_2_), 1.27 (dd, *J* = 14.9, 6.0 Hz, 5H, CH_2_), 1.04 (dd, *J* = 18.1, 9.4 Hz, 5H, CH_2_), 0.96 – 0.87 (m, 4H, CH_2_), 0.85 – 0.76 (m, 15H, CH_3_), 0.57 (s, 3H, CH_3_).

^13^C NMR (101 MHz, Chloroform-*d*) δ 194.4, 170.1, 138.3, 136.4, 131.6, 127.3, 127.3, 121.7, 74.1, 55.6, 55.1, 48.9, 41.2, 38.6, 38.5, 36.4, 35.7, 35.4, 35.1, 34.7, 32.2, 30.8, 30.7, 27.2, 27.0, 26.1, 23.2, 22.8, 21.8, 21.5, 19.9, 18.1, 17.7, 14.9, 14.8, 10.8.

**HRMS** (TOF-ESI^+^): *m/z* calcd for C_38_H_54_O_3_ [M+H] ^+^, 559.4146; found, 559.4150.

Spectroscopic Data of **4a**

benzyl 1-(4-fluorobenzoyl)cyclopropane-1-carboxylate

83% yield; Colorless liquid

**IR** (KBr): 3782, 3445, 3384, 3070, 2971, 1733, 1686, 1602, 1507, 1453, 1381, 1314, 1226, 1156, 1012, 846, 804, 704;

^1^H NMR (400 MHz, Chloroform-*d*) δ 7.77 (dd, *J* = 8.8, 5.4 Hz, 2H, PhH), 7.20 – 7.11 (m, 3H, PhH), 6.94 (t, *J* = 8.6 Hz, 2H, PhH), 6.89 (d, *J* = 7.0 Hz, 2H, PhH), 4.96 (s, 2H, CH_2_), 1.59 – 1.53 (m, 2H, CH_2_), 1.50 – 1.43 (m, 2H, CH_2_).

^13^C NMR (101 MHz, Chloroform-*d*) δ 193.2, 171.4, 166.5 (*J* = 260.0 Hz), 135.0, 133.4 (*J* = 10.0 Hz), 131.0 (*J* = 10.0 Hz), 128.4, 128.3, 128.1, 115.6 (*J* = 20.0 Hz), 67.2, 32.9, 16.1.

^19^F NMR (376 MHz, Chloroform-*d*) δ -105.46.

**HRMS** (TOF-ESI^+^): *m/z* calcd for C_18_H_15_FO_3_ [M+H] ^+^, 299.1078; found, 299.1079.

Spectroscopic Data of **4b**

benzyl 1-(4-chlorobenzoyl)cyclopropane-1-carboxylate

85% yield; White solid; Mp: 88.1 – 89.8 ^o^C

**IR** (KBr): 3780, 3548, 3483, 3377, 3121, 3032, 2965, 1732, 1690, 1589, 1316, 1161, 1098, 1001, 841, 747;

^1^H NMR (400 MHz, Acetone-*d*_6_) δ 7.70 (d, *J* = 8.5 Hz, 2H, PhH), 7.33 (d, *J* = 8.5 Hz, 2H, PhH), 7.11 (q, *J* = 7.3, 6.3 Hz, 3H, PhH), 6.88 (d, *J* = 6.6 Hz, 2H, PhH), 4.91 (s, 2H, CH_2_), 1.48 – 1.42 (m, 2H, CH_2_), 1.42 – 1.36 (m, 2H, CH_2_).

^13^C NMR (101 MHz, Acetone-*d*_6_) δ 193.0, 170.8, 138.4, 135.8, 135.6, 129.9, 128.8, 128.3, 128.1, 128.0, 66.7, 32.6, 15.2.

**HRMS** (TOF-ESI^+^): *m/z* calcd for C_18_H_15_ClO_3_ [M+H] ^+^, 315.0782; found, 315.0780.

Spectroscopic Data of **4c**

benzyl 1-(4-nitrobenzoyl)cyclopropane-1-carboxylate

81% yield; White solid; Mp: 101.2 – 103.3 ^o^C

**IR** (KBr): 3852, 3725, 3296, 3268, 3052, 2964, 1712, 1655, 1537, 1428, 1381, 1334, 1098, 1072, 1001, 890, 731;

^1^H NMR (400 MHz, Chloroform-*d*) δ 8.00 (d, *J* = 8.8 Hz, 2H, PhH), 7.77 (d, *J* = 8.8 Hz, 2H, PhH), 7.20 – 7.15 (m, 1H, PhH), 7.10 (t, *J* = 7.4 Hz, 2H, PhH), 6.87 (d, *J* = 7.2 Hz, 2H, PhH), 4.94 (s, 2H, CH_2_), 1.67 – 1.60 (m, 2H, CH_2_), 1.60 – 1.53 (m, 2H, CH_2_).

^13^C NMR (101 MHz, Chloroform-*d*) δ 192.9, 169.6, 148.8, 141.2, 133.5, 127.8, 127.5, 127.5, 127.4, 122.6, 66.3, 32.4, 15.8.

**HRMS** (TOF-ESI^+^): *m/z* calcd for C_18_H_15_NO_5_ [M+H] ^+^, 326.1023; found, 326.1025.

Spectroscopic Data of **4d**

benzyl 1-(4-cyanobenzoyl)cyclopropane-1-carboxylate

84% yield; White solid; Mp: 96.0 – 97.8 ^o^C

**IR** (KBr): 3892, 3831, 3526, 3351, 3031, 2951, 2223, 1685, 1595, 1472, 1456, 1310, 1213, 1170, 1085, 1023, 896, 795, 751, 690;

^1^H NMR (400 MHz, Chloroform-*d*) δ 7.73 (d, *J* = 8.3 Hz, 2H, PhH), 7.48 (d, *J* = 8.3 Hz, 2H, PhH), 7.23 (t, *J* = 7.4 Hz, 1H, PhH), 7.15 (t, *J* = 7.5 Hz, 2H, PhH), 6.86 (d, *J* = 7.4 Hz, 2H, PhH), 4.93 (s, 2H, CH_2_), 1.61 (q, *J* = 4.2, 3.4 Hz, 2H, CH_2_), 1.52 (q, *J* = 5.0, 4.2 Hz, 2H, CH_2_).

^13^C NMR (101 MHz, Chloroform-*d*) δ 192.9, 169.7, 139.5, 133.6, 131.2, 127.5, 127.4, 127.4, 116.9, 114.8, 66.2, 32.1, 15.6.

**HRMS** (TOF-ESI^+^): *m/z* calcd for C_19_H_15_NO_3_ [M+H] ^+^, 306.1125; found, 306.1121.

Spectroscopic Data of **4e**

benzyl 1-(4-methylbenzoyl)cyclopropane-1-carboxylate

69% yield; Colorless liquid

**IR** (KBr): 3820, 3720, 3627, 3427, 3223, 3024, 2932, 1731, 1679, 1606, 1451, 1320, 1157, 1018, 895, 837, 749, 668;

^1^H NMR (400 MHz, Acetone-*d*_6_) δ 7.76 (d, *J* = 8.2 Hz, 2H, PhH), 7.30 – 7.22 (m, 5H, PhH), 6.99 (d, *J* = 6.4 Hz, 2H, PhH), 5.04 (s, 2H, CH_2_), 2.41 (s, 3H, CH_3_), 1.58 – 1.52 (m, 2H, CH_2_), 1.52 – 1.45 (m, 2H, CH_2_).

^13^C NMR (101 MHz, Acetone-*d*_6_) δ 193.4, 171.1, 143.6, 135.7, 134.6, 129.2, 128.4, 128.2, 127.9, 127.8, 66.5, 32.6, 20.7, 14.9.

**HRMS** (TOF-ESI^+^): *m/z* calcd for C_19_H_18_O_3_ [M+H] ^+^, 295.1329; found, 295.1333.

Spectroscopic Data of **4f**

benzyl 1-(3-fluorobenzoyl)cyclopropane-1-carboxylate

80% yield; Colorless liquid

**IR** (KBr): 3960, 3826, 3564, 3449, 3078, 3037, 2965, 1733, 1689, 1591, 1490, 1449, 1380, 1310, 1248, 1178, 1137, 1086, 1023, 909, 798, 748, 690;

^1^H NMR (400 MHz, Acetone-*d*_6_) δ 7.56 (d, *J* = 7.7 Hz, 1H, PhH), 7.42 – 7.36 (m, 2H) , PhH, 7.25 (t, *J* = 8.5 Hz, 1H, PhH), 7.11 (d, *J* = 6.9 Hz, 3H, PhH), 6.88 (d, *J* = 7.3 Hz, 2H, PhH), 4.91 (s, 2H, CH_2_), 1.50 – 1.45 (m, 2H, CH_2_), 1.44 – 1.38 (m, 2H, CH_2_).

^13^C NMR (101 MHz, Acetone-*d*_6_) δ 193.0, 170.7, 162.7 (*J* = 240.0 Hz), 139.5 (*J* = 10.0 Hz), 135.6, 130.8 (*J* = 10.0 Hz), 128.3, 128.1, 127.9, 124.3 (*J* = 10.0 Hz), 119.7, 119.5, 114.5 (*J* = 20.0 Hz), 66.8, 32.7, 15.3.

^19^F NMR (376 MHz, Acetone-*d*_6_) δ -113.64.

**HRMS** (TOF-ESI^+^): *m/z* calcd for C_18_H_15_FO_3_ [M+H] ^+^, 299.1078; found, 299.1079.

Spectroscopic Data of **4g**

benzyl 1-(3-chlorobenzoyl)cyclopropane-1-carboxylate

85% yield; Colorless liquid

**IR** (KBr): 3828, 3558, 3361, 3076, 3040, 2954, 1733, 1689, 1577, 1423, 1380, 1316, 1210, 1161, 1021, 893, 803, 746, 705;

^1^H NMR (400 MHz, Acetone-*d*_6_) δ 7.80 (dt, *J* = 3.3, 1.9 Hz, 2H, PhH), 7.64 (d, *J* = 8.1 Hz, 1H, PhH), 7.49 (t, *J* = 8.1 Hz, 1H, PhH), 7.28 – 7.22 (m, 3H, PhH), 7.01 (d, *J* = 7.8 Hz, 2H, PhH), 5.05 (s, 2H, CH_2_), 1.65 – 1.60 (m, 2H, CH_2_), 1.59 – 1.54 (m, 2H, CH_2_).

^13^C NMR (101 MHz, Acetone-*d*_6_) δ 193.0, 170.6, 139.1, 135.5, 134.3, 132.6, 130.5, 128.3, 128.1, 127.8, 127.8, 126.7, 66.8, 32.7, 15.3.

**HRMS** (TOF-ESI^+^): *m/z* calcd for C_18_H_15_ClO_3_ [M+H] ^+^, 315.0782; found, 315.0779.

Spectroscopic Data of **4h**

benzyl 1-(3-bromobenzoyl)cyclopropane-1-carboxylate

88% yield; Colorless liquid

**IR** (KBr): 3541, 3454, 3383, 3077, 3037, 2966, 1733, 1688, 1572, 1457, 1419, 1381, 1316, 1208, 1157, 1077, 1016, 888, 798, 747;

^1^H NMR (400 MHz, Acetone-*d*_6_) δ 7.81 (s, 1H, PhH), 7.69 (d, *J* = 7.8 Hz, 1H, PhH), 7.63 (d, *J* = 8.0 Hz, 1H, PhH), 7.27 (t, *J* = 7.9 Hz, 1H, PhH), 7.14 – 7.07 (m, 3H, PhH), 6.90 – 6.83 (m, 2H, PhH), 4.90 (s, 2H, CH_2_), 1.50 – 1.45 (m, 2H, CH_2_), 1.44 – 1.39 (m, 2H, CH_2_).

^13^C NMR (101 MHz, Acetone-*d*_6_) δ 193.0, 170.6, 139.3, 135.6, 135.5, 130.8, 130.7, 128.4, 128.1, 127.8, 127.1, 122.3, 66.8, 32.7, 15.4.

**HRMS** (TOF-ESI^+^): *m/z* calcd for C_18_H_15_BrO_3_ [M+H] ^+^, 359.0277; found, 359.0275.

Spectroscopic Data of **4i**

benzyl 1-(3-methylbenzoyl)cyclopropane-1-carboxylate

72% yield; Colorless liquid

**IR** (KBr): 3771, 3665, 3360, 3041, 2968, 2926, 1732, 1684, 1598, 1381, 1247, 1180, 1146, 1040, 795, 745, 690;

^1^H NMR (400 MHz, Acetone-*d*_6_) δ 7.53 (d, *J* = 8.4 Hz, 2H, PhH), 7.29 (d, *J* = 7.6 Hz, 1H, PhH), 7.22 (t, *J* = 7.5 Hz, 1H, PhH), 7.09 (q, *J* = 6.8, 6.1 Hz, 3H, PhH), 6.83 (d, *J* = 6.2 Hz, 2H, PhH), 4.89 (s, 2H, CH_2_), 2.20 (s, 3H, CH_3_), 1.46 – 1.40 (m, 2H, CH_2_), 1.39 – 1.34 (m, 2H, CH_2_).

^13^C NMR (101 MHz, Acetone-*d*_6_) δ 194.0, 171.0, 138.4, 137.3, 135.7, 133.5, 128.7, 128.5, 128.2, 127.9, 127.8, 125.5, 66.6, 32.7, 20.4, 15.1.

**HRMS** (TOF-ESI^+^): *m/z* calcd for C_19_H_18_O_3_ [M+H] ^+^, 295.1329; found, 295.1329.

Spectroscopic Data of **4j**

benzyl 1-(2-chlorobenzoyl)cyclopropane-1-carboxylate

77% yield; Colorless liquid

**IR** (KBr): 3873, 3769, 3380, 3269, 3076, 3035, 2964, 1733, 1686, 1593, 1439, 1382, 1315, 1160, 1072, 1000, 749;

^1^H NMR (400 MHz, Acetone-*d*_6_) δ 7.42 (d, *J* = 6.1 Hz, 1H, PhH), 7.36 – 7.30 (m, 1H, PhH), 7.29 – 7.21 (m, 2H, PhH), 7.15 – 7.10 (m, 3H, PhH), 6.88 (dd, *J* = 7.0, 2.4 Hz, 2H, PhH), 4.84 (s, 2H, CH_2_), 1.63 – 1.59 (m, 2H, CH_2_), 1.59 – 1.55 (m, 2H, CH_2_).

^13^C NMR (101 MHz, Acetone-*d*_6_) δ 196.3, 170.0, 139.3, 135.4, 131.8, 130.7, 130.2, 129.1, 128.3, 128.0, 127.0, 66.7, 35.0, 19.7.

**HRMS** (TOF-ESI^+^): *m/z* calcd for C_18_H_15_ClO_3_ [M+H] ^+^, 315.0782; found, 315.0785.

Spectroscopic Data of **4k**

benzyl 1-(2-methylbenzoyl)cyclopropane-1-carboxylate

63% yield; Colorless liquid

**IR** (KBr): 3839, 3810, 3751, 3436, 3374, 3200, 2976, 1732, 1684, 1455, 1317, 1158, 1066, 965, 934, 860, 754, 656;

^1^H NMR (400 MHz, Acetone-*d*_6_) δ 7.52 (d, *J* = 7.7 Hz, 1H, PhH), 7.27 (t, *J* = 7.5 Hz, 1H, PhH), 7.17 – 7.07 (m, 5H, PhH), 6.77 (d, *J* = 7.2 Hz, 2H, PhH), 4.79 (s, 2H, CH_2_), 2.21 (s, 3H, CH_3_), 1.51 – 1.47 (m, 2H, CH_2_), 1.47 – 1.43 (m, 2H, CH_2_).

^13^C NMR (101 MHz, Acetone-*d*_6_) δ 197.7, 170.6, 138.7, 137.5, 135.6, 131.6, 130.9, 128.2, 127.9, 127.8, 127.6, 1256, 66.7, 34.4, 19.5, 16.5.

**HRMS** (TOF-ESI^+^): *m/z* calcd for C_19_H_18_O_3_ [M+H] ^+^, 295.1329; found, 295.1327.

Spectroscopic Data of **4l**

benzyl 1-(3,5-dimethylbenzoyl)cyclopropane-1-carboxylate

72% yield; Colorless liquid

**IR** (KBr): 3572, 3042, 2960, 2928, 1734, 1683, 1606, 1453, 1381, 1326, 1182, 1145, 1065, 995, 909, 863, 796, 749;

^1^H NMR (400 MHz, Acetone-*d*_6_) δ 7.32 (s, 2H, PhH), 7.10 (d, *J* = 7.4 Hz, 4H, PhH), 6.83 (d, *J* = 6.3 Hz, 2H, PhH), 4.89 (s, 2H, CH_2_), 2.17 (s, 6H, CH_3_), 1.45 – 1.38 (m, 2H, CH_2_), 1.38 – 1.31 (m, 2H, CH_2_).

^13^C NMR (101 MHz, Acetone-*d*_6_) δ 194.1, 171.1, 138.2, 137.4, 135.8, 134.2, 128.2, 127.9, 127.7, 126.0, 66.5, 32.7, 20.3, 15.0.

**HRMS** (TOF-ESI^+^): *m/z* calcd for C_20_H_20_O_3_ [M+Na] ^+^, 331.1305; found, 331.1304.

Spectroscopic Data of **4m**

benzyl 1-(furan-2-carbonyl)cyclopropane-1-carboxylate

75% yield; Colorless liquid

**IR** (KBr): 3842, 3346, 3123, 3038, 2956, 1735, 1672, 1575, 1463, 1389, 1314, 1168, 1023, 751;

^1^H NMR (400 MHz, Acetone-*d*_6_) δ 7.61 – 7.58 (m, 1H, ArH), 7.17 (dd, *J* = 5.1, 1.9 Hz, 3H, ArH), 7.08 (d, *J* = 3.6 Hz, 1H, ArH), 7.04 (dd, *J* = 6.8, 2.6 Hz, 2H, ArH), 6.50 (dd, *J* = 3.6, 1.7 Hz, 1H, ArH), 4.99 (s, 2H, CH_2_), 1.38 – 1.33 (m, 2H, CH_2_), 1.32 – 1.27 (m, 2H, CH_2_).

^13^C NMR (101 MHz, Acetone-*d*_6_) δ 181.9, 170.6, 152.3, 146.9, 135.9, 128.4, 128.0, 128.0, 117.3, 112.3, 66.7, 32.5, 14.3.

**HRMS** (TOF-ESI^+^): *m/z* calcd for C_16_H_14_O_4_ [M+H] ^+^, 271.0965; found, 271.0970.

Spectroscopic Data of **4n**

benzyl 1-(thiophene-2-carbonyl)cyclopropane-1-carboxylate

78% yield; Colorless liquid

**IR** (KBr): 3635, 3315, 3189, 3101, 3033, 2964, 1745, 1668, 1517, 1456, 1416, 1396, 1216, 1156, 1052, 965, 852, 803, 745, 514;

^1^H NMR (400 MHz, Acetone-*d*_6_) δ 7.75 (d, *J* = 5.0 Hz, 1H, ArH), 7.59 (d, *J* = 3.8 Hz, 1H, ArH), 7.17 – 7.11 (m, 3H, ArH), 7.06 – 7.02 (m, 1H, ArH), 7.00 (dd, *J* = 6.3, 3.2 Hz, 2H, ArH), 4.99 (s, 2H, CH_2_), 1.43 – 1.37 (m, 2H, CH_2_), 1.37 – 1.31 (m, 2H, CH_2_).

^13^C NMR (101 MHz, Acetone-*d*_6_) δ 186.2, 170.8, 143.2, 135.8, 133.8, 133.0, 128.3, 128.3, 128.0, 127.8, 66.7, 33.2, 14.7.

**HRMS** (TOF-ESI^+^): *m/z* calcd for C_16_H_14_SO_3_ [M+H] ^+^, 287.0736; found, 287.0740.

Spectroscopic Data of **4o**

benzyl 1-(2-naphthoyl)cyclopropane-1-carboxylate

81% yield; Colorless liquid

**IR** (KBr): 3839, 3672, 3460, 3265, 3022, 2953, 1736, 1689, 1596, 1478, 1393, 1333, 1220, 1114, 1021, 887, 791, 689;

^1^H NMR (400 MHz, Acetone-*d*_6_) δ 8.46 (s, 1H, PhH), 7.99 (dd, *J* = 13.5, 7.4 Hz, 3H, PhH), 7.92 (dd, *J* = 8.6, 1.6 Hz, 1H, PhH), 7.67 (t, *J* = 7.5 Hz, 1H, PhH), 7.61 (t, *J* = 7.6 Hz, 1H, PhH), 7.10 (t, *J* = 7.4 Hz, 1H, PhH), 6.99 (t, *J* = 7.6 Hz, 2H, PhH), 6.86 (d, *J* = 7.6 Hz, 2H, PhH), 5.02 (s, 2H, CH_2_), 1.68 – 1.63 (m, 2H, CH_2_), 1.62 – 1.57 (m, 2H, CH_2_).

^13^C NMR (101 MHz, Acetone-*d*_6_) δ 194.0, 171.1, 135.6, 135.5, 134.6, 132.6, 129.8, 129.6, 128.4, 128.4, 128.1, 127.8, 127.7, 127.6, 126.8, 124.1, 66.6, 32.8, 15.2.

**HRMS** (TOF-ESI^+^): *m/z* calcd for C_22_H_18_O_3_ [M+H] ^+^, 331.1329; found, 331.1331.

Spectroscopic Data of **4p**

benzyl 1-(cyclohexanecarbonyl)cyclopropane-1-carboxylate

59% yield; Colorless liquid

**IR** (KBr): 3702, 3616, 3434, 3366, 2940, 2862, 1730, 1700, 1453, 1380, 1312, 1165, 1087, 1009, 883, 838, 799, 751;

^1^H NMR (400 MHz, Chloroform-*d*) δ 7.29 (p, *J* = 3.9, 3.4 Hz, 5H, PhH), 5.11 (s, 2H, CH_2_), 2.96 (tt, *J* = 11.3, 3.4 Hz, 1H, CyH), 1.73 (d, *J* = 12.9 Hz, 2H, CyH), 1.66 – 1.59 (m, 2H, CyH), 1.56 – 1.49 (m, 1H, CyH), 1.38 – 1.34 (m, 2H, CH_2_), 1.34 – 1.30 (m, 2H, CH_2_), 1.19 (q, *J* = 15.2, 13.9 Hz, 2H, CyH), 1.07 (d, *J* = 7.2 Hz, 3H, CyH).

^13^C NMR (101 MHz, Chloroform-*d*) δ 207.1, 170.1, 149.3, 134.3, 127.6, 127.5, 66.1, 48.2, 32.9, 28.3, 24.8, 24.6, 16.8.

**HRMS** (TOF-ESI^+^): *m/z* calcd for C_18_H_22_O_3_ [M+H] ^+^, 287.1642; found, 287.1640.

Spectroscopic Data of **5**

2-phenyl-4,5-dihydrofuran-3-carbonitrile

91% yield; Colorless liquid

**IR** (KBr): 3928, 3839, 3783, 3204, 2215, 1615, 1338, 1119, 924, 748;

^1^H NMR (400 MHz, Chloroform-*d*) δ 7.86 (dd, *J* = 8.0, 1.7 Hz, 2H, PhH), 7.41 – 7.35 (m, 3H, PhH), 4.57 (t, *J* = 9.7 Hz, 2H, CH_2_), 3.04 (t, *J* = 9.7 Hz, 2H, CH_2_).

^13^C NMR (101 MHz, Chloroform-*d*) δ 167.6, 131.4, 128.7, 127.9, 127.1, 117.8, 79.6, 71.0, 31.8.

**HRMS** (TOF-ESI^+^): *m/z* calcd for C_11_H_9_NO [M+H] ^+^, 172.0757; found, 172.0760.

Spectroscopic Data of **6**

1-(hydroxy(phenyl)methyl)cyclopropane-1-carbonitrile

99% yield; Colorless liquid

^1^H NMR (400 MHz, Chloroform-*d*) δ 7.49 – 7.32 (m, 5H, PhH), 4.35 (s, 1H, CH), 2.86 (s, 1H, OH), 1.31 – 1.17 (m, 2H, CH_2_), 1.16 – 1.04 (m, 2H, CH_2_).

^13^C NMR (101 MHz, Chloroform-*d*) δ 140.1, 128.7, 128.7, 126.3, 121.9, 75.3, 17.5, 12.7, 11.5.

**HRMS** (TOF-ESI^+^): *m/z* calcd for C_11_H_11_NO [M+H] ^+^, 174.0913; found, 174.0915.

Spectroscopic Data of **7**

1-benzoylcyclopropane-1-carboxylic acid

99% yield; White soild

^1^H NMR (400 MHz, Chloroform-*d*) δ 11.20 (s, 1H, COOH), 7.91 (d, *J* = 7.2 Hz, 2H, PhH), 7.55 (t, *J* = 7.4 Hz, 1H, PhH), 7.44 (t, *J* = 7.6 Hz, 2H, PhH), 1.67 (q, *J* = 4.3, 3.4 Hz, 2H, CH_2_), 1.58 (q, *J* = 5.1, 4.3 Hz, 2H, CH_2_).

^13^C NMR (101 MHz, Chloroform-*d*) δ 194.3, 136.4, 133.2, 128.7, 128.6, 32.5, 16.9.

**HRMS** (TOF-ESI^+^): *m/z* calcd for C_11_H_10_O_3_ [M+H] ^+^, 191.0703; found, 191.0700.

Spectroscopic Data of **3a’**

benzyl 2-benzoylcyclopropane-1-carboxylate

Colorless liquid

All spectroscopic data were in agreement with those reported previously in the literature ^3^.

^1^H NMR (400 MHz, Chloroform-*d*) δ 8.00 (d, *J* = 7.9 Hz, 2H, PhH), 7.58 (t, *J* = 7.3 Hz, 1H, PhH), 7.47 (t, *J* = 7.6 Hz, 2H, PhH), 7.39 – 7.32 (m, 5H, PhH), 5.16 (s, 2H, CH_2_), 3.22 (dt, *J* = 9.1, 4.8 Hz, 1H, CH), 2.45 (dt, *J* = 9.2, 4.9 Hz, 1H, CH), 1.70 – 1.57 (m, 2H, CH_2_).

^13^C NMR (101 MHz, Chloroform-*d*) δ 197.0, 172.2, 137.0, 135.6, 133.5, 128.7, 128.7, 128.4, 128.3, 128.3, 67.0, 26.1, 24.7, 18.1.

Spectroscopic Data of **3a-*d_2_***

benzyl 1-benzoylcyclopropane-1-carboxylate-2,2-*d*_2_

^1^H NMR (400 MHz, DMSO-*d*_6_) δ 7.84 (dd, *J* = 8.3, 1.2 Hz, 2H, PhH), 7.65 (t, *J* = 7.4 Hz, 1H, PhH), 7.51 (t, *J* = 7.7 Hz, 2H, PhH), 7.27 – 7.19 (m, 3H, PhH), 6.92 (d, *J* = 6.5 Hz, 2H, PhH), 5.02 (s, 2H, CH_2_), 1.57 (d, *J* = 4.1 Hz, 1H, CH_2_), 1.51 (d, *J* = 4.2 Hz, 1H, CH_2_).

**HRMS** (TOF-ESI^+^): *m/z* calcd for C_18_H_14_D_2_O_3_ [M+Na] ^+^, 305.1117; found, 305.1116.

Spectroscopic Data of **3a’-*d***

benzyl 2-benzoylcyclopropane-1-carboxylate-2-*d*

^1^H NMR (400 MHz, Chloroform-*d*) δ 7.94 (d, *J* = 7.3 Hz, 2H, PhH), 7.53 (t, *J* = 7.4 Hz, 1H, PhH), 7.42 (t, *J* = 7.7 Hz, 2H, PhH), 7.30 (d, *J* = 3.5 Hz, 5H, PhH), 5.10 (s, 2H, CH_2_), 2.37 (dd, *J* = 8.6, 5.9 Hz, 1H, CH), 1.61 – 1.54 (m, 2H, CH_2_).


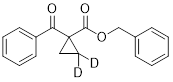


Spectroscopic Data of **int III & int IV**

# X-ray structure and data of **3d**


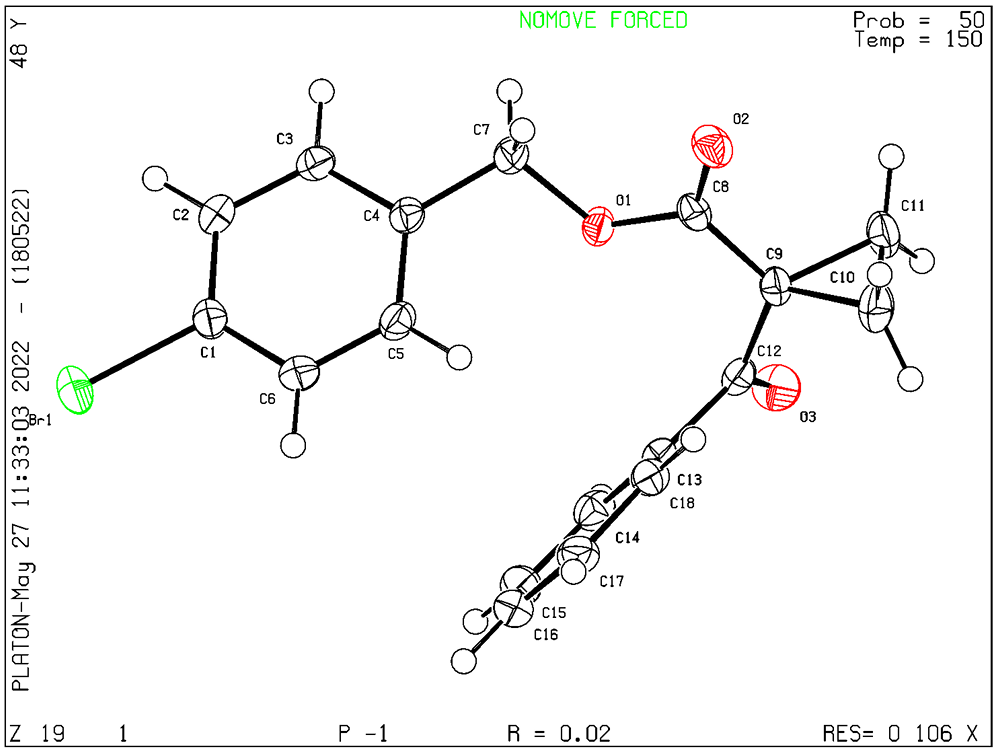


Figure S2. X-Ray crystal structure of 3d

Table S4. Crystal data and structure refinement for 3d


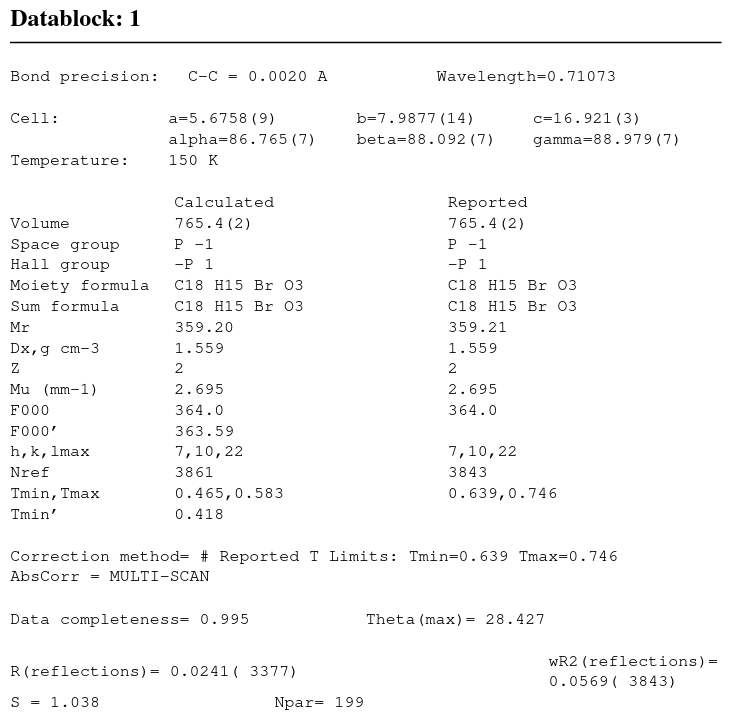


Compound **3d** (50mg) was add to a 10mL sample bottle, following to add DCM (2mL), n-hexane (2.5mL) and toluene (0.1mL), then seal the bottle with a parafilm, and poke 15 small holes on the parafilm, place the sample bottle in a safe place to allow it to volatilize and separate out the single crystal. Take out the single crystal and send it for single crystal diffraction test to obtain relevant data. Instrument model：Intensity data for single crystals of each complex were collected on a BRUKER SMART APEX II CCD detector with graphite-monochromatized Mo Kα radiation (k = 0.071073 nm). The structures were solved by direct method using the program SHELXS-97 and subsequent Fourier difference techniques, and refined anisotropically by full matrix least-squares on F2 using SHELXL-97.

# References and notes

1. Li, J. L.; He, H.; Huang, M. Y.; Chen, Y. C.; Luo, Y.; Yan, K.C.; Wang, Q. T. and Wu, Y. *Org. Lett.* **2019**, *21*, 9005–9008.
2. Zhang, J. F.; Tang, Y. H.; Wei, W.; Wu, Y.; Li, Y.; Zhang, J. J.; Zheng, Y. S.; and Xu, S. L. *Org. Lett.* **2017**, *19*, 3043–3046.
3. Duchemin, C and Cramer, N. *Chem. Sci.* **2019**, *10*, 2773-2777.
4. Frisch, M. J. T., G. W.; Schlegel, H. B.; Scuseria, G. E.; Robb, M. A.; Cheeseman, J. R.; Scalmani, G.; Barone, V.; Petersson, G. A.; Nakatsuji, H.; Li, X.; Caricato, M.; Marenich, A. V.; Bloino, J.; Janesko, B. G.; Gomperts, R.; Mennucci, B.; Hratchian, H. P.; Ortiz, J. V.; Izmaylov, A. F.; Sonnenberg, J. L.; Williams-Young, D.; Ding, F.; Lipparini, F.; Egidi, F.; Goings, J.; Peng, B.; Petrone, A.; Henderson, T.; Ranasinghe, D.; Zakrzewski, V. G.; Gao, J.; Rega, N.; Zheng, G.; Liang, W.; Hada, M.; Ehara, M.; Toyota, K.; Fukuda, R.; Hasegawa, J.; Ishida, M.; Nakajima, T.; Honda, Y.; Kitao, O.; Nakai, H.; Vreven, T.; Throssell, K.; Montgomery, J. A., Jr.; Peralta, J. E.; Ogliaro, F.; Bearpark, M. J.; Heyd, J. J.; Brothers, E. N.; Kudin, K. N.; Staroverov, V. N.; Keith, T. A.; Kobayashi, R.; Normand, J.; Raghavachari, K.; Rendell, A. P.; Burant, J. C.; Iyengar, S. S.; Tomasi,J.; Cossi, M.; Millam, J. M.; Klene, M.; Adamo, C.; Cammi, R.; Ochterski, J. W.; Martin, R. L.; Morokuma, K.; Farkas, O.; Foresman, J. B.; Fox, D. J. , Gaussian, Inc., Wallingford CT, **2016**.
5. J. P. Perdew, K. Burke, M. Ernzerhof, *Phys. Rev. Lett.* **1996**, *77*, 3865.
6. (a) Marenich, A. V.; Cramer, C. J.; Truhlar, D. G. *J. Phys. Chem. B* **2009**, 113, 6378; (b) Marenich, A. V.; Cramer, C. J.; Truhlar, D. G. *J. Phys. Chem. B* **2009**, *113*, 4538.
7. Legault, C. Y. CYLview; Université de Sherbrooke, Sherbrooke, Canada, **2009**.

# ^1^H NMR and ^13^C NMR spectra of these compounds


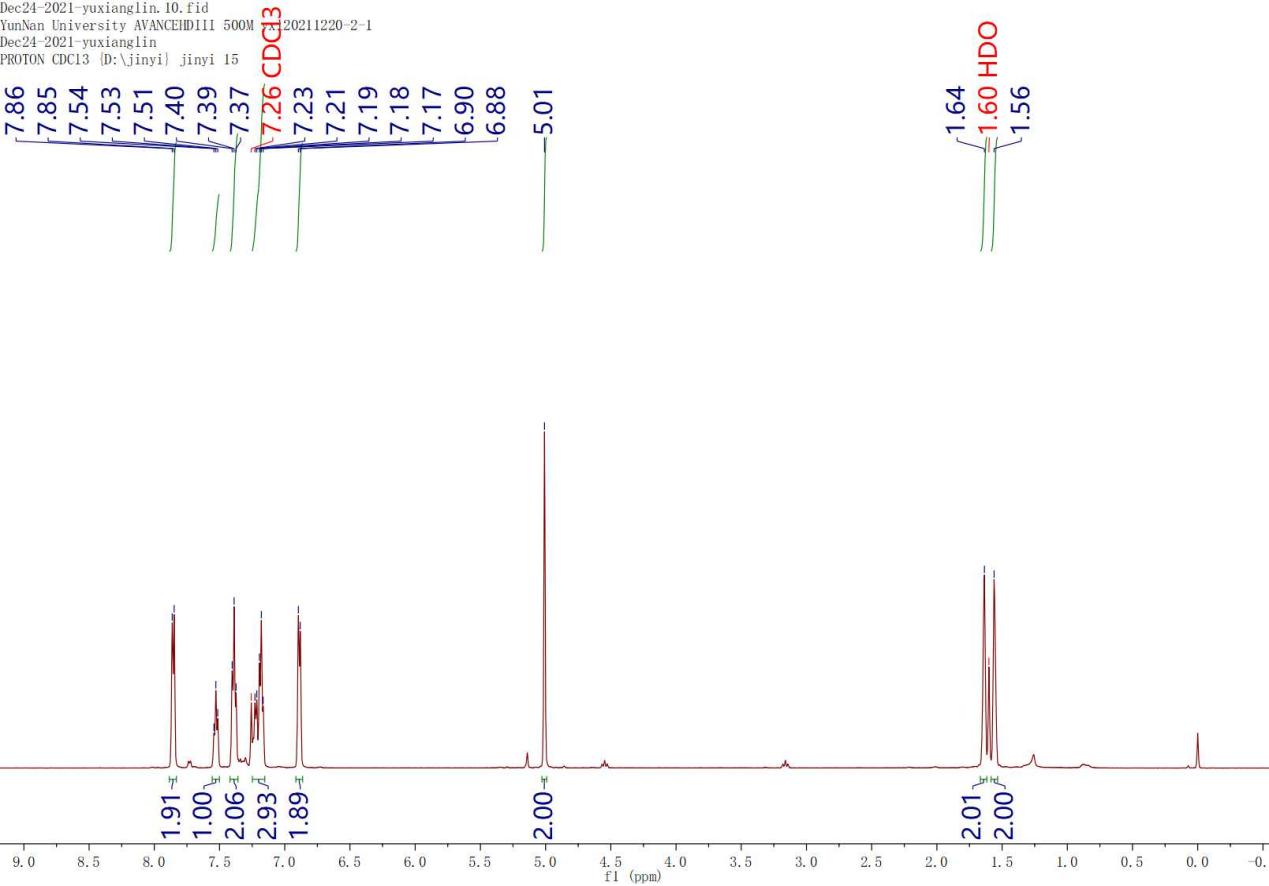


^1^H-NMR spectrum of compound (**3a**)


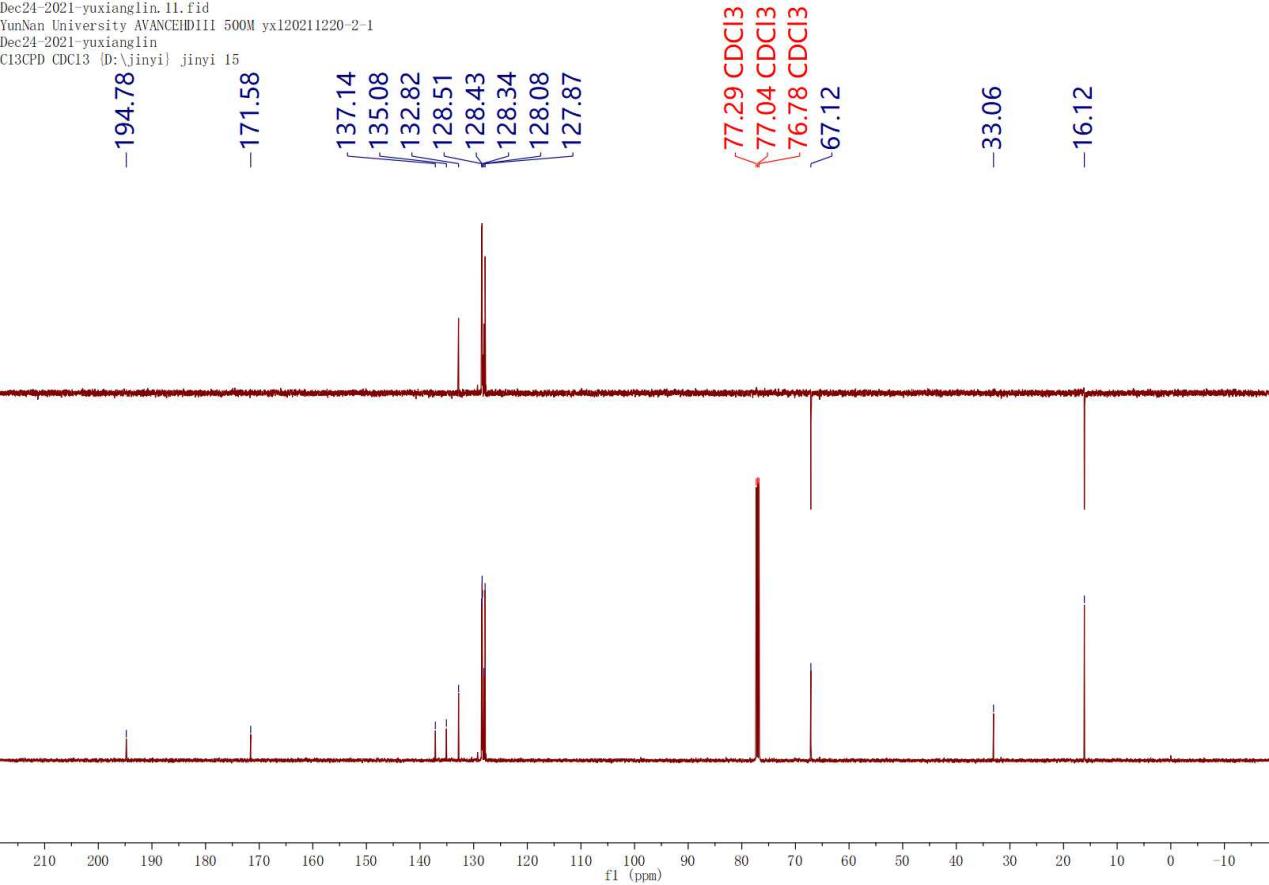


^13^C-NMR spectrum of compound (**3a**)


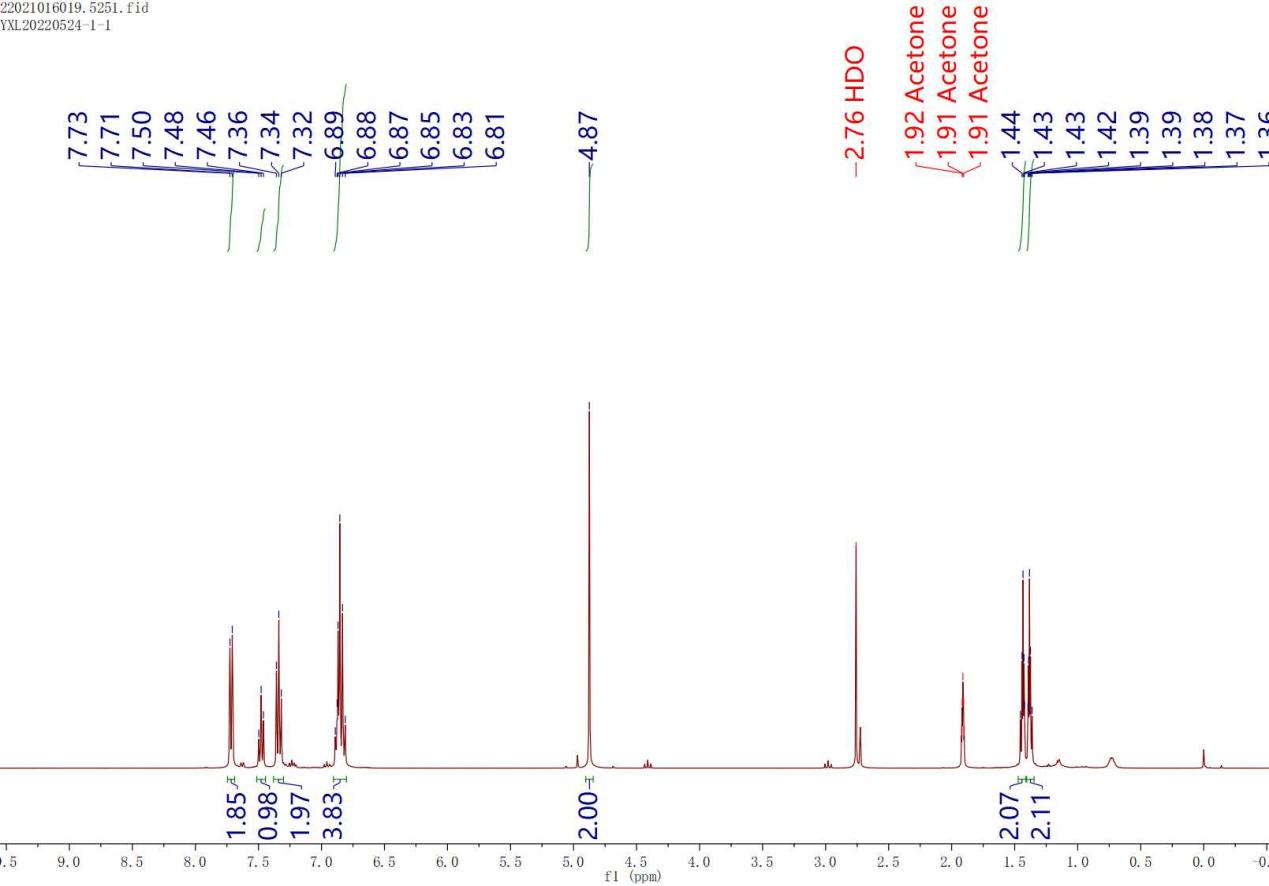


^1^H-NMR spectrum of compound (**3b**)


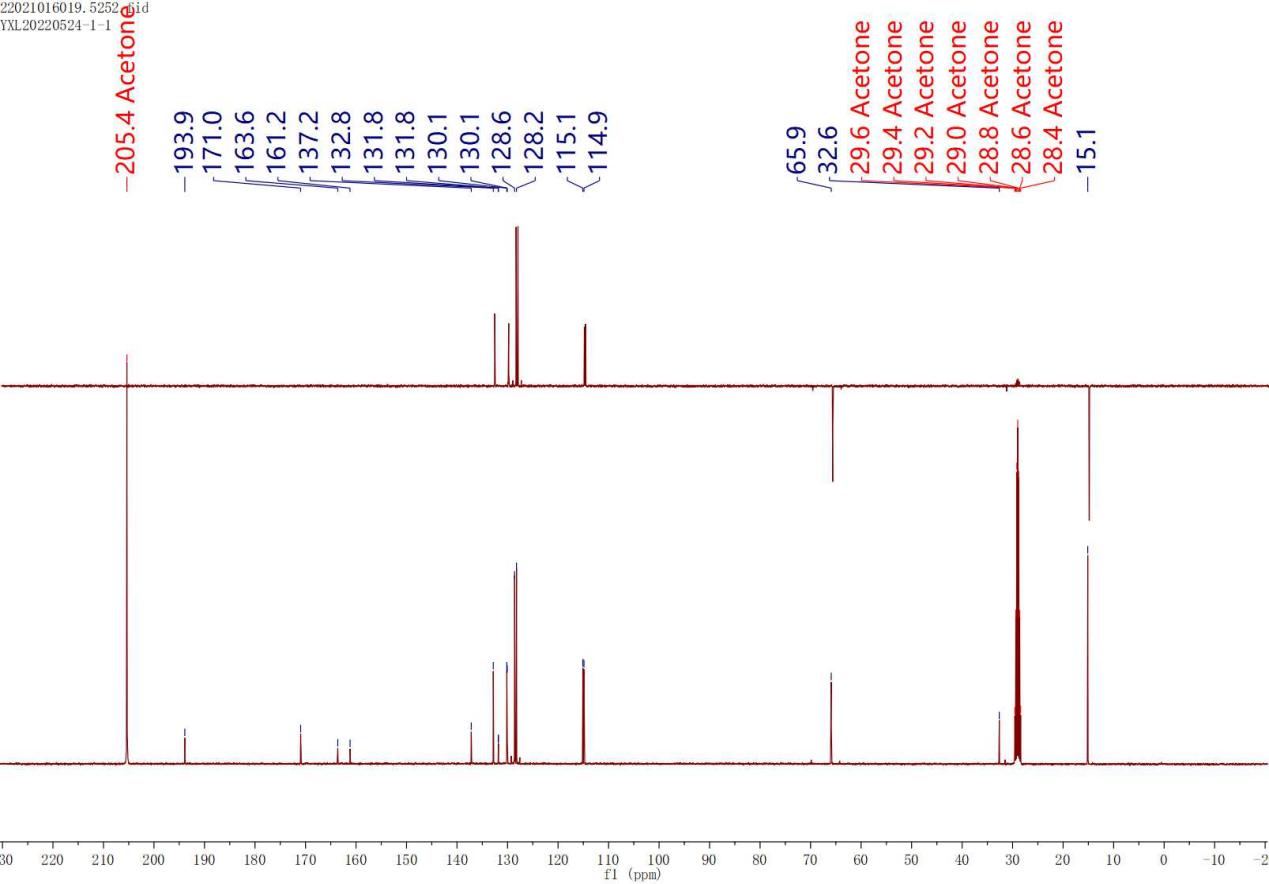


^13^C-NMR spectrum of compound (**3b**)


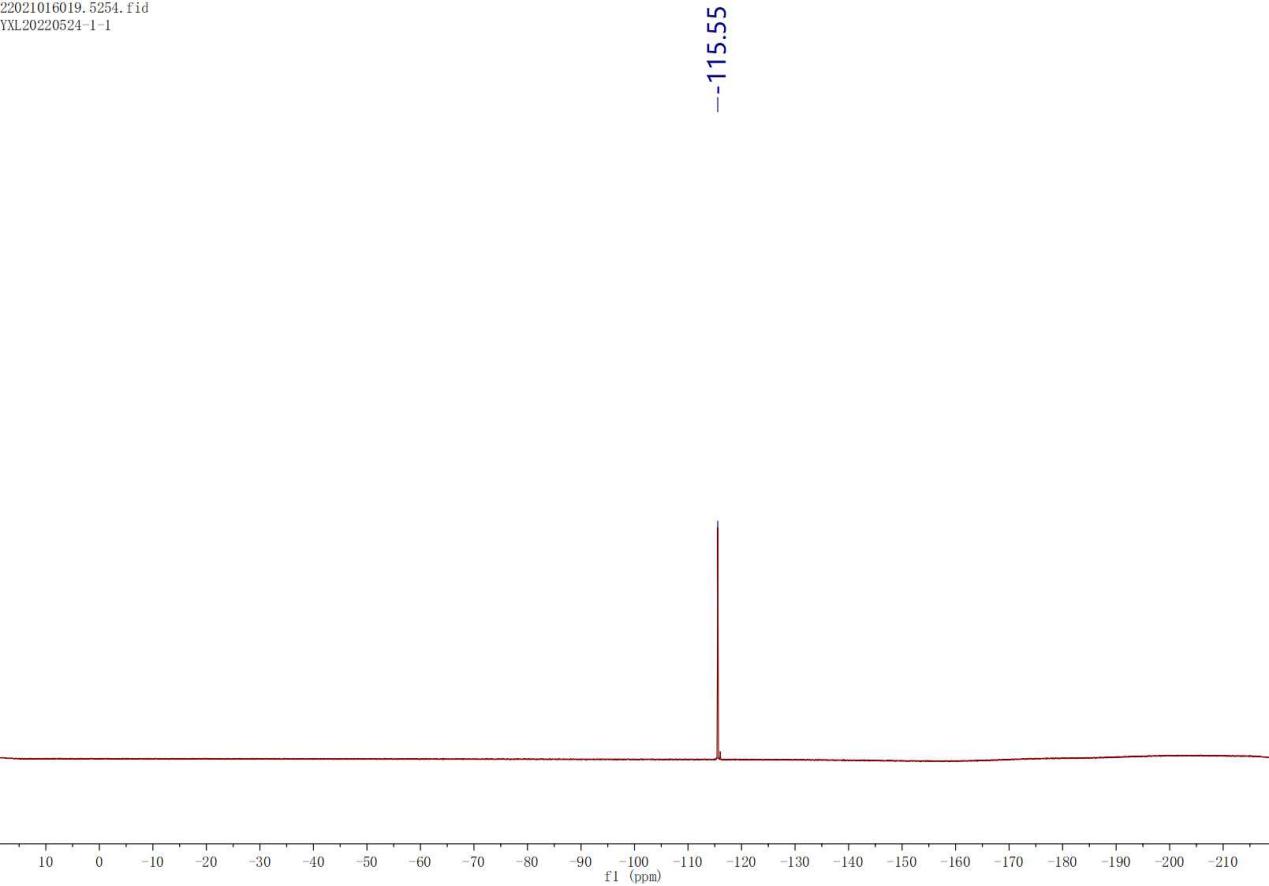


^19^F-NMR spectrum of compound (**3b**)

^^
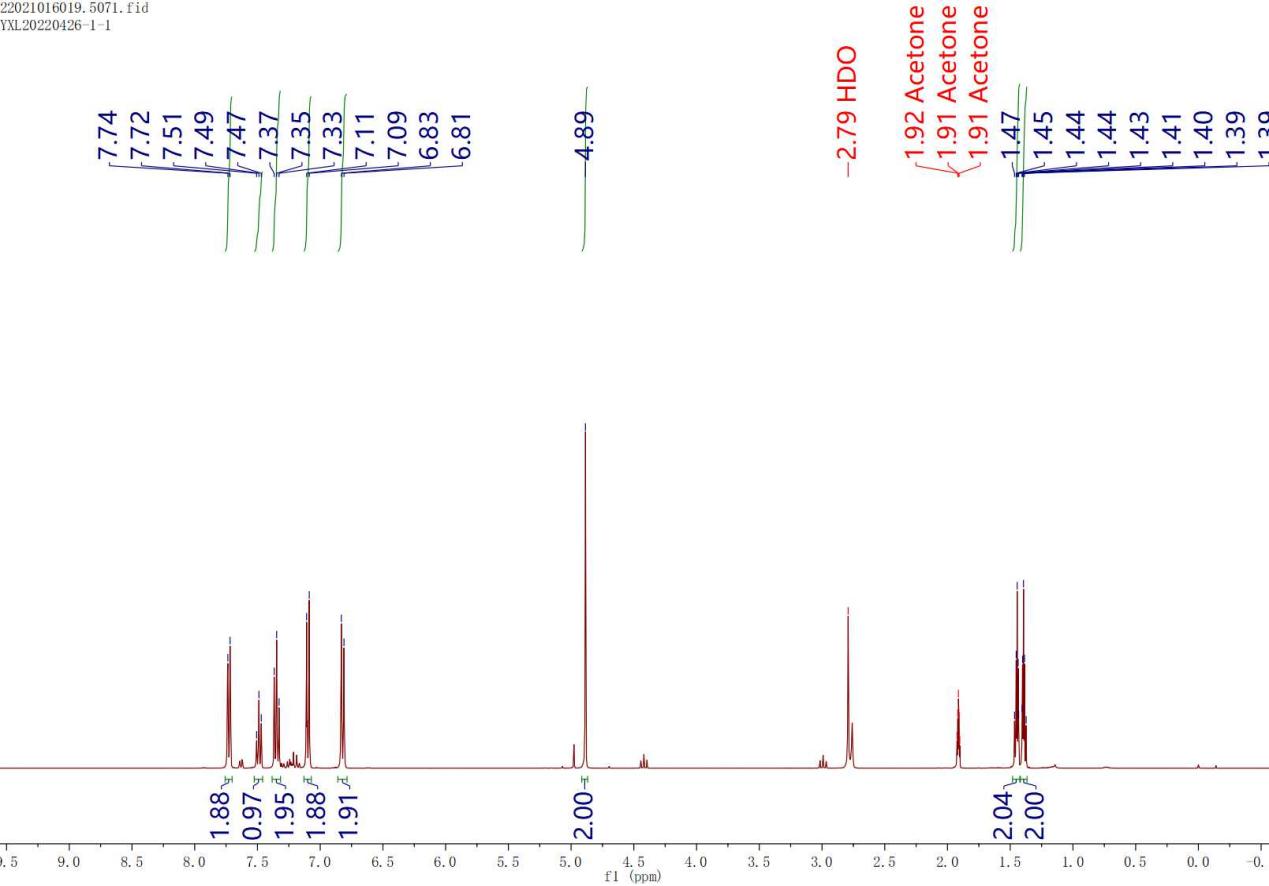


^1^H-NMR spectrum of compound (**3c**)


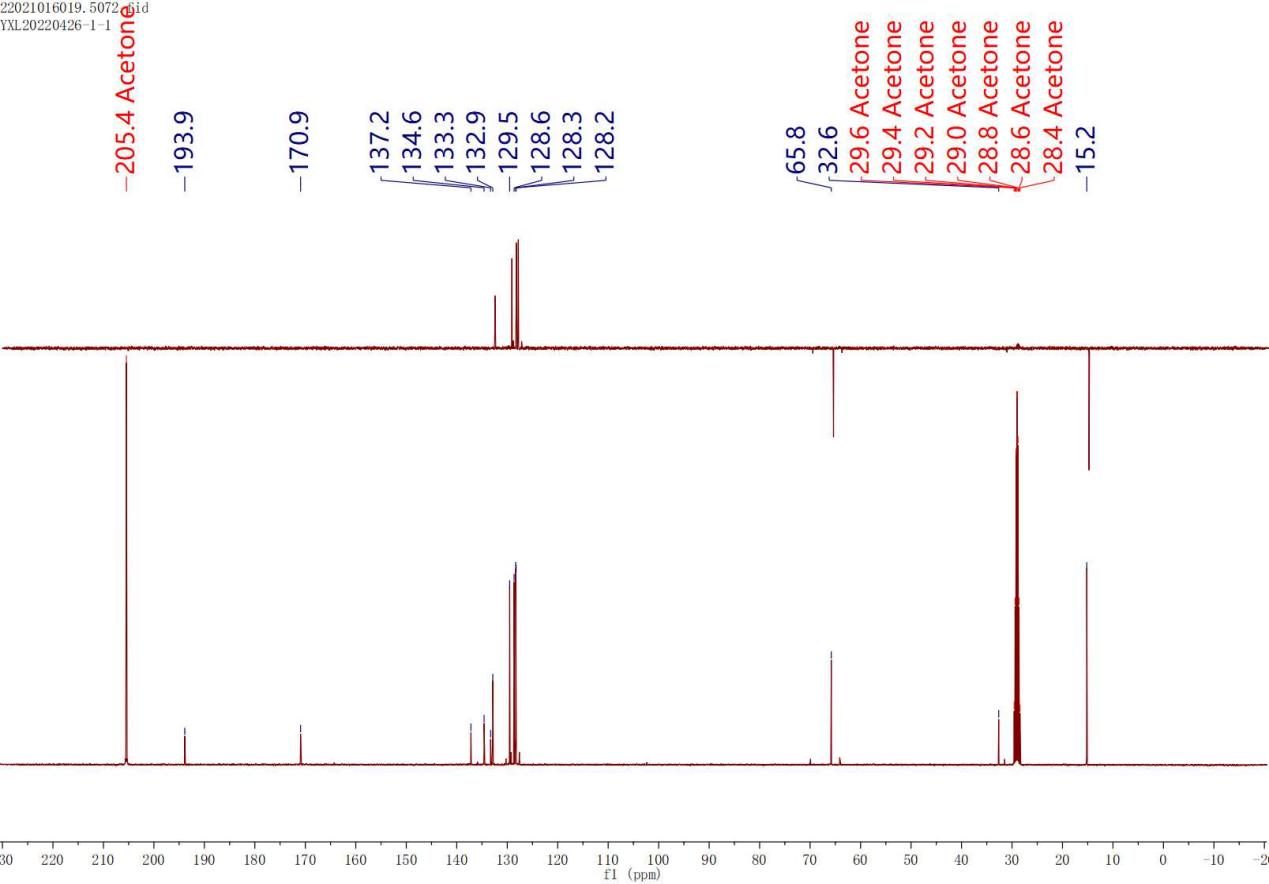


^13^C-NMR spectrum of compound (**3c**)

^
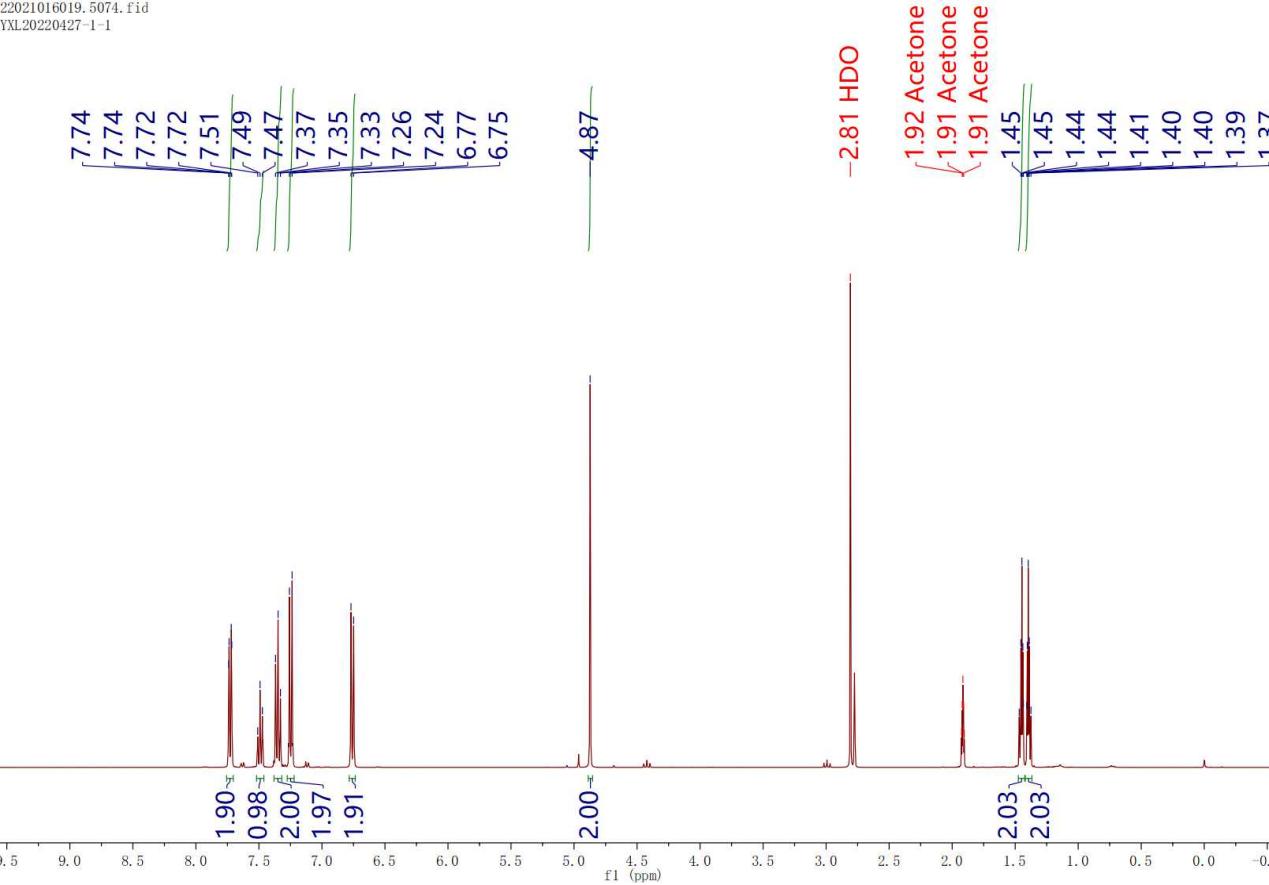
^

^1^H-NMR spectrum of compound (**3d**)


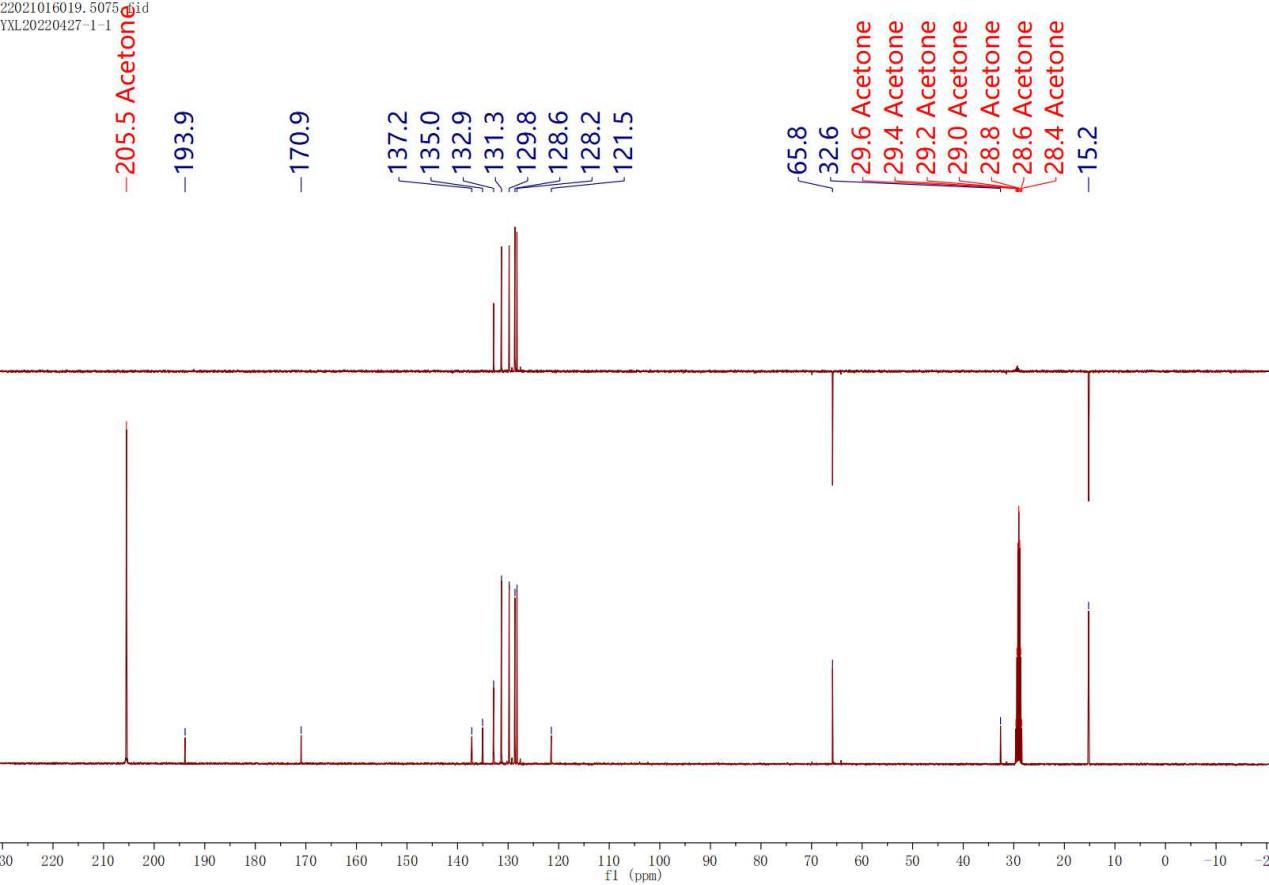


^13^C-NMR spectrum of compound (**3d**)

^^
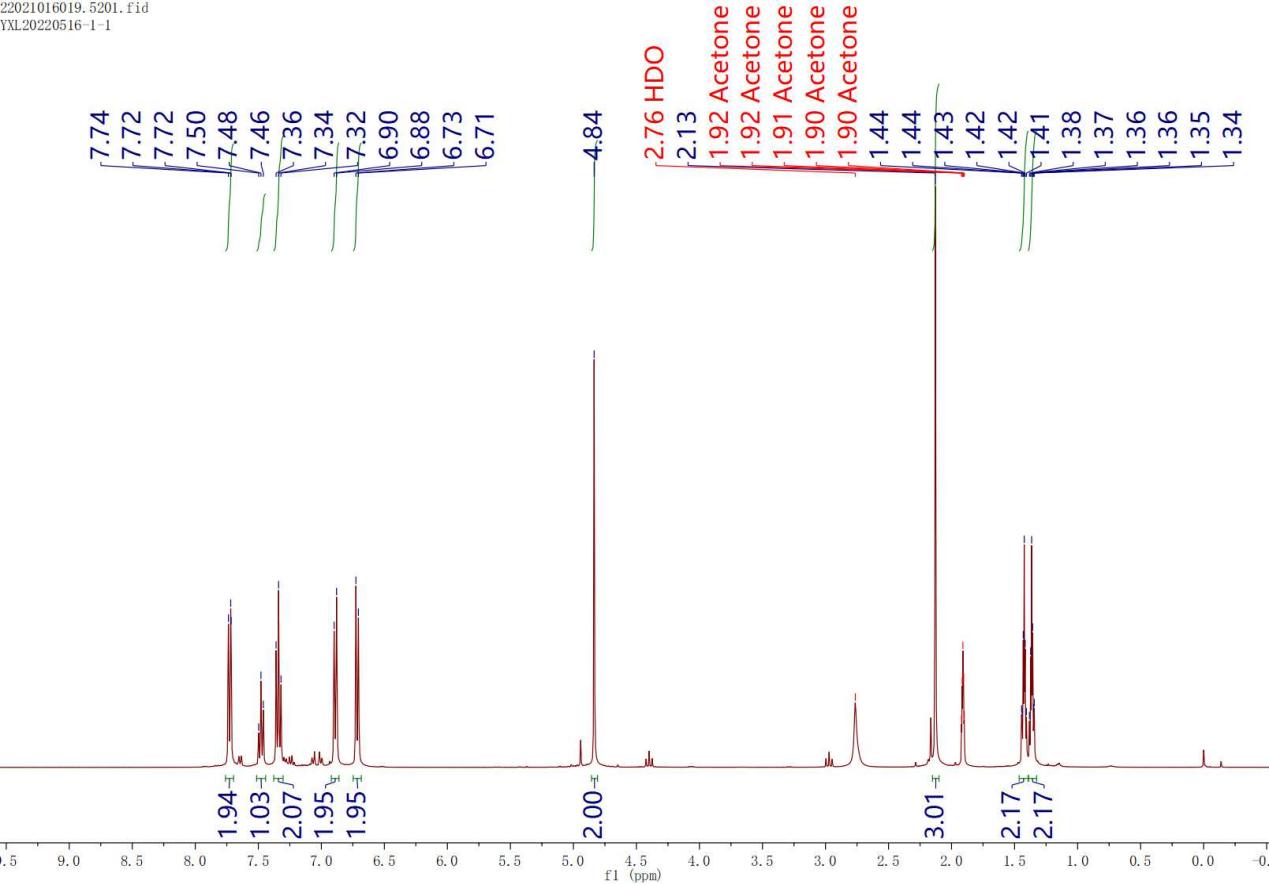


^1^H-NMR spectrum of compound (**3e**)


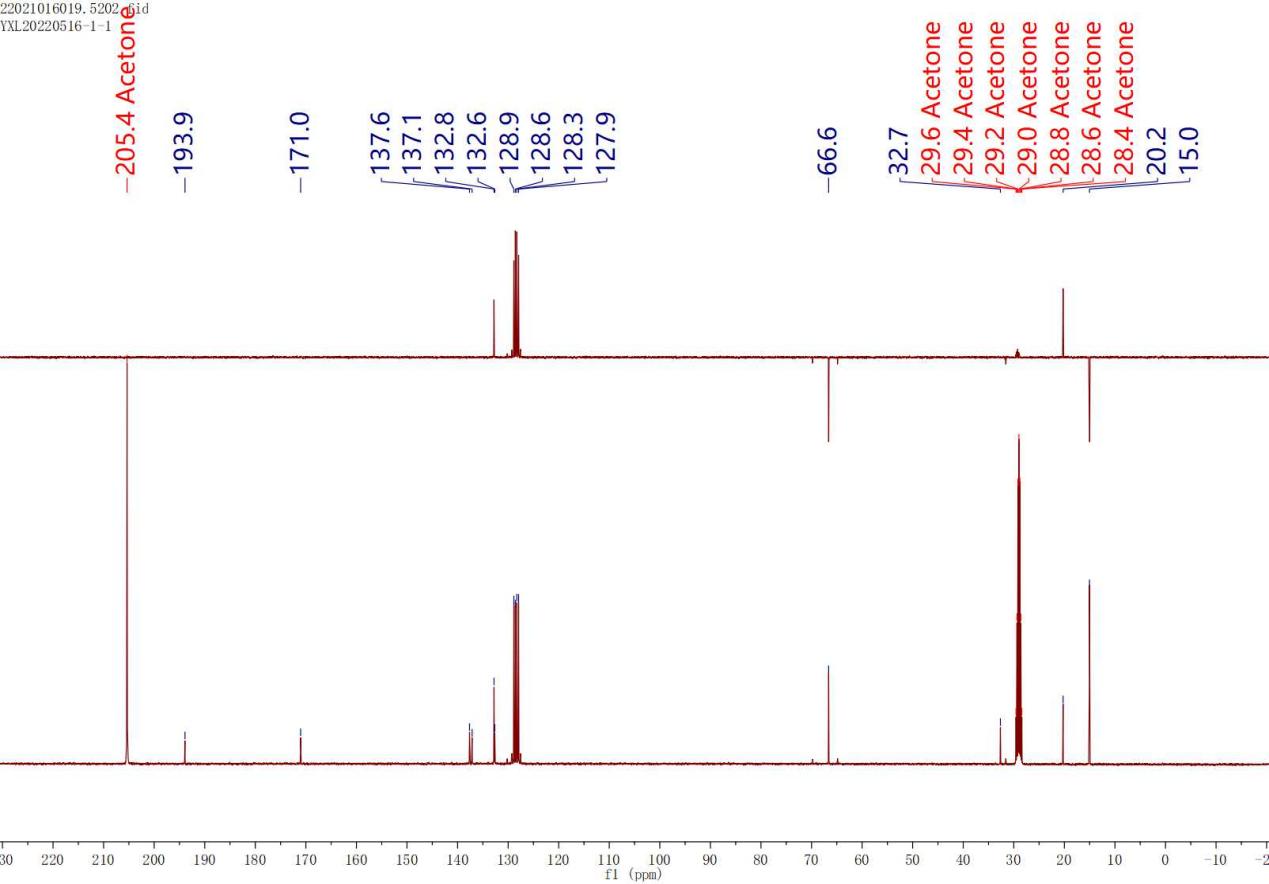


^13^C-NMR spectrum of compound (**3e**)

^^
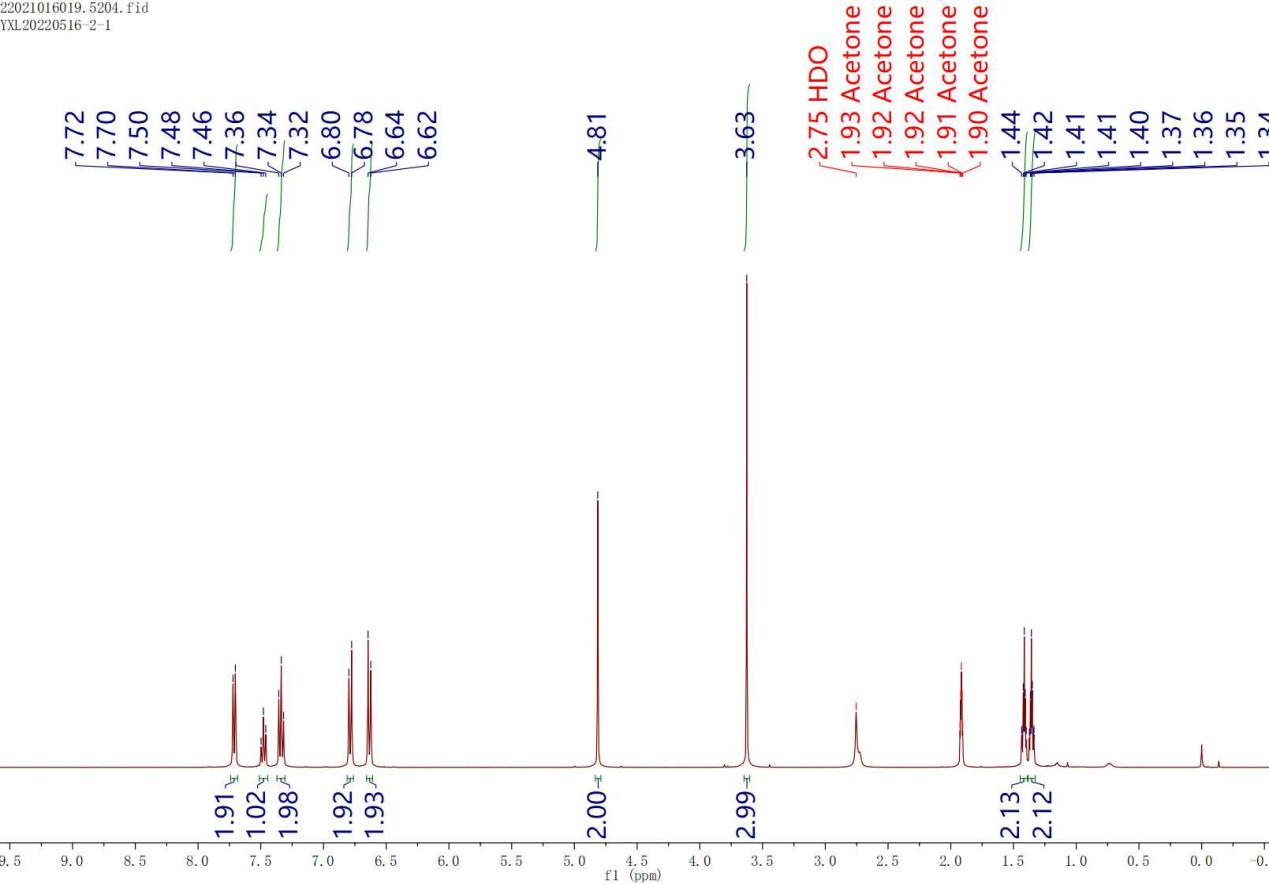


^1^H-NMR spectrum of compound (**3f**)


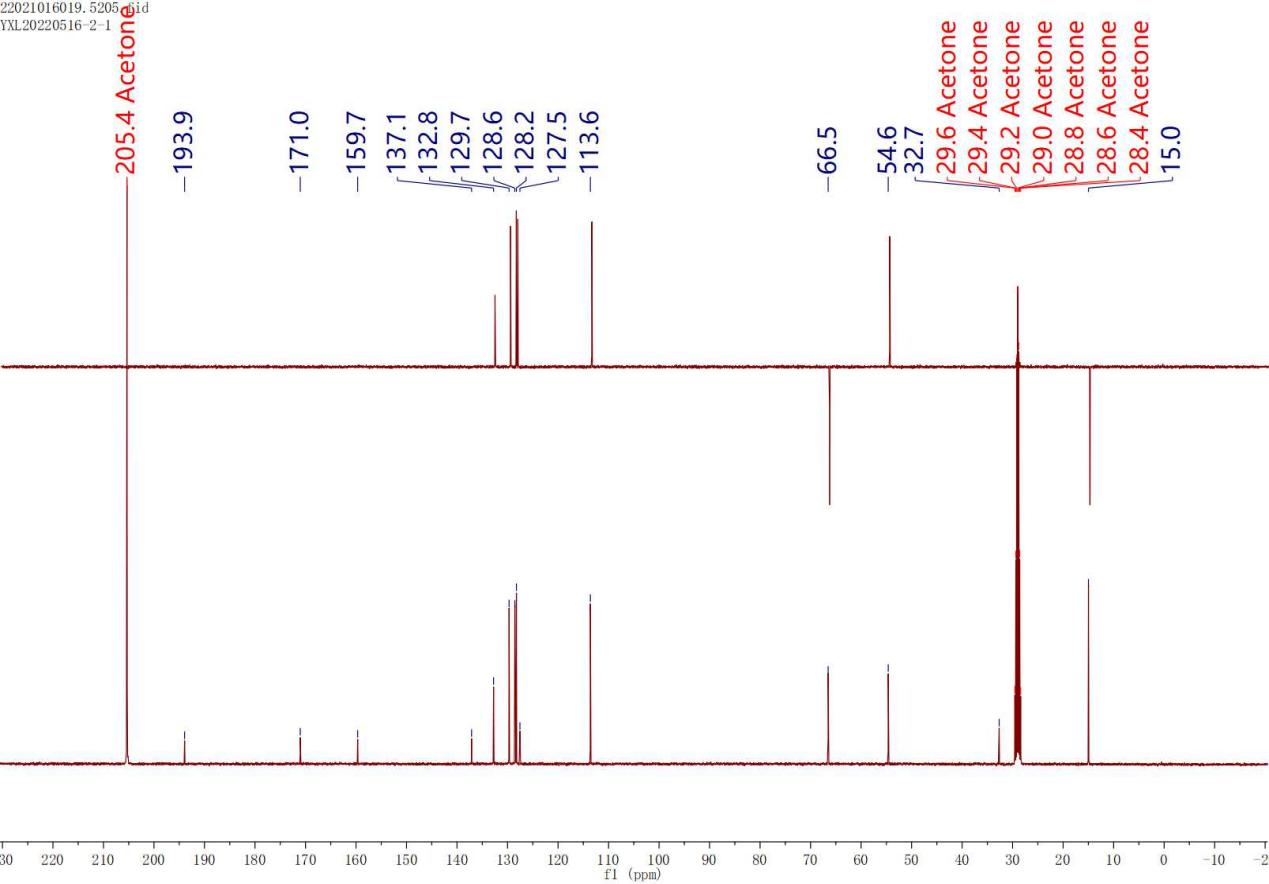


^13^C-NMR spectrum of compound (**3f**)

^^
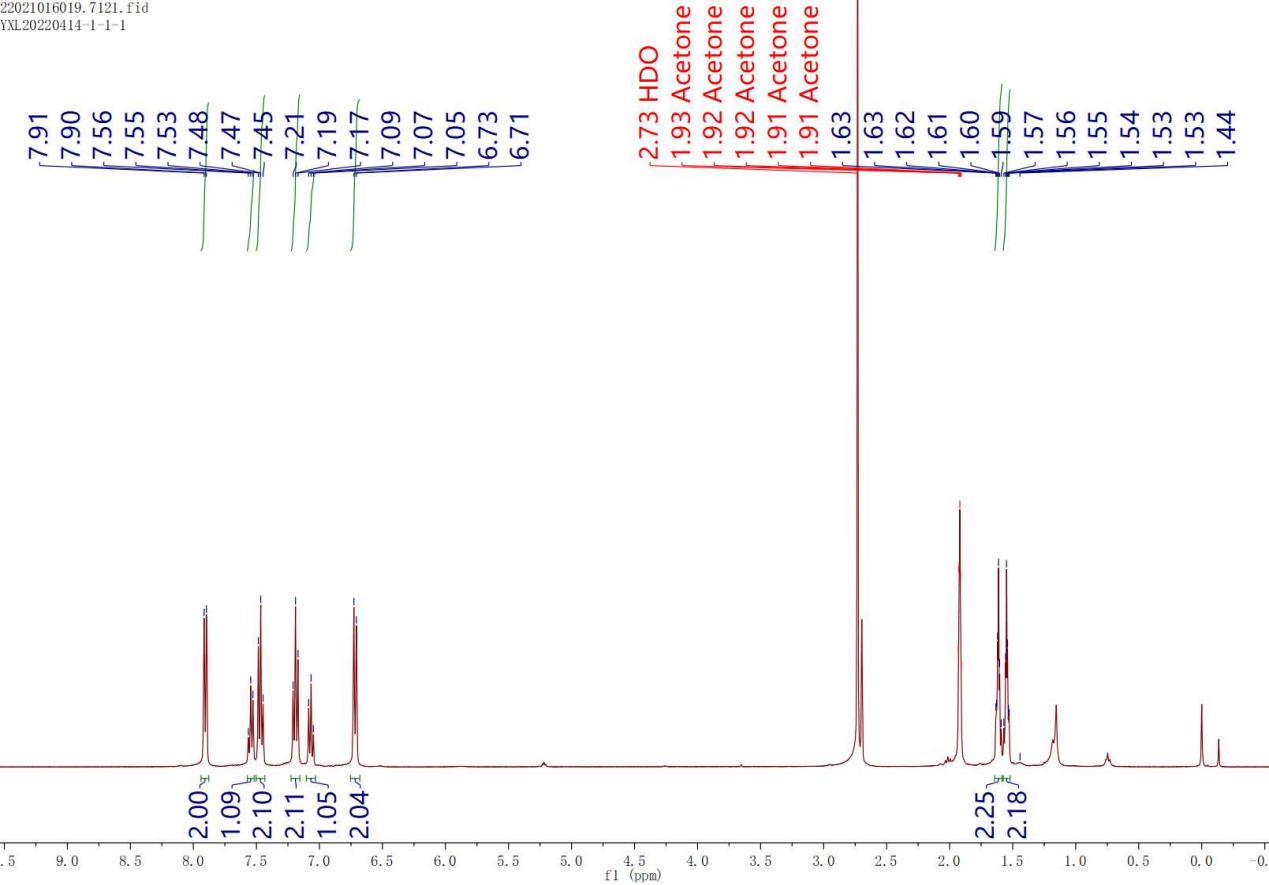


^1^H-NMR spectrum of compound (**3g**)


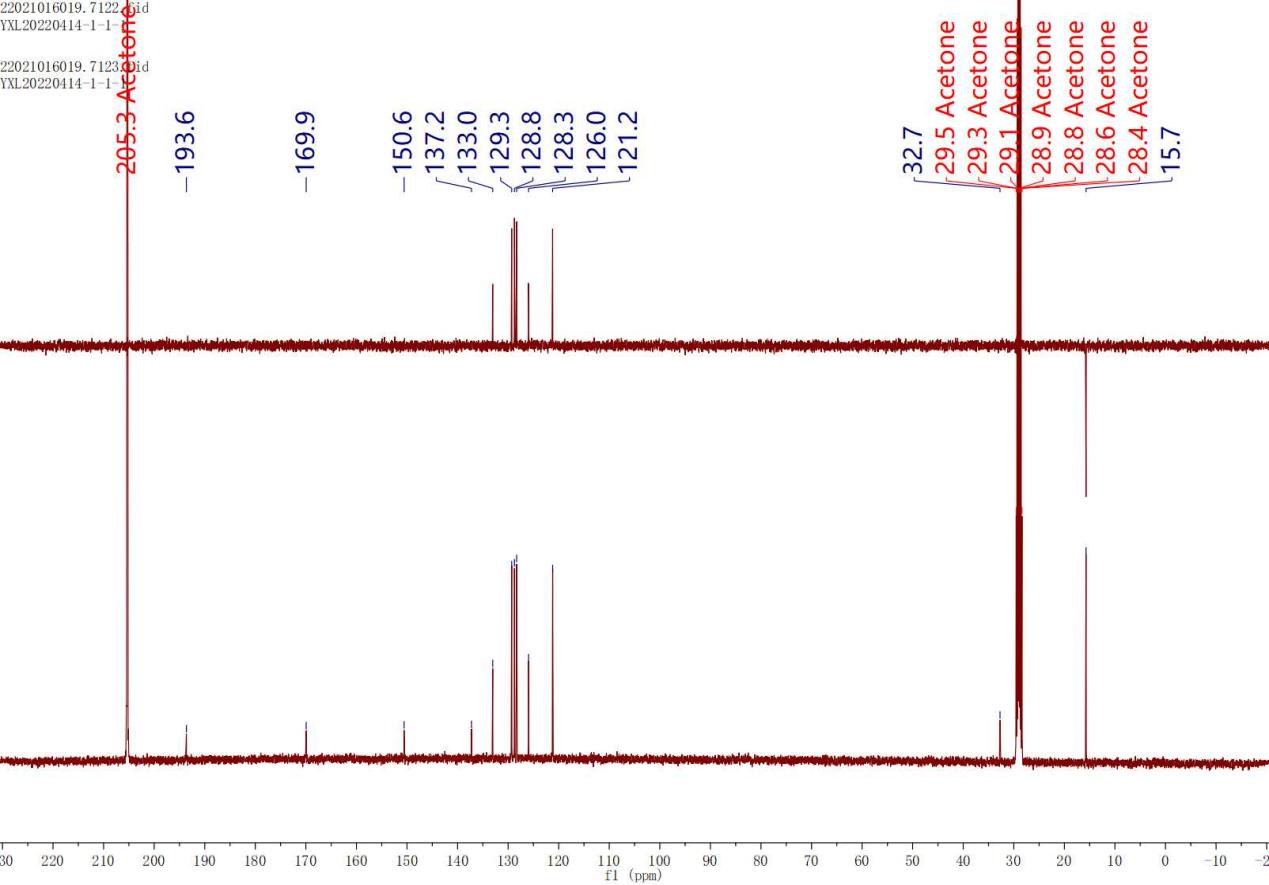


^13^C-NMR spectrum of compound (**3g**)

^^
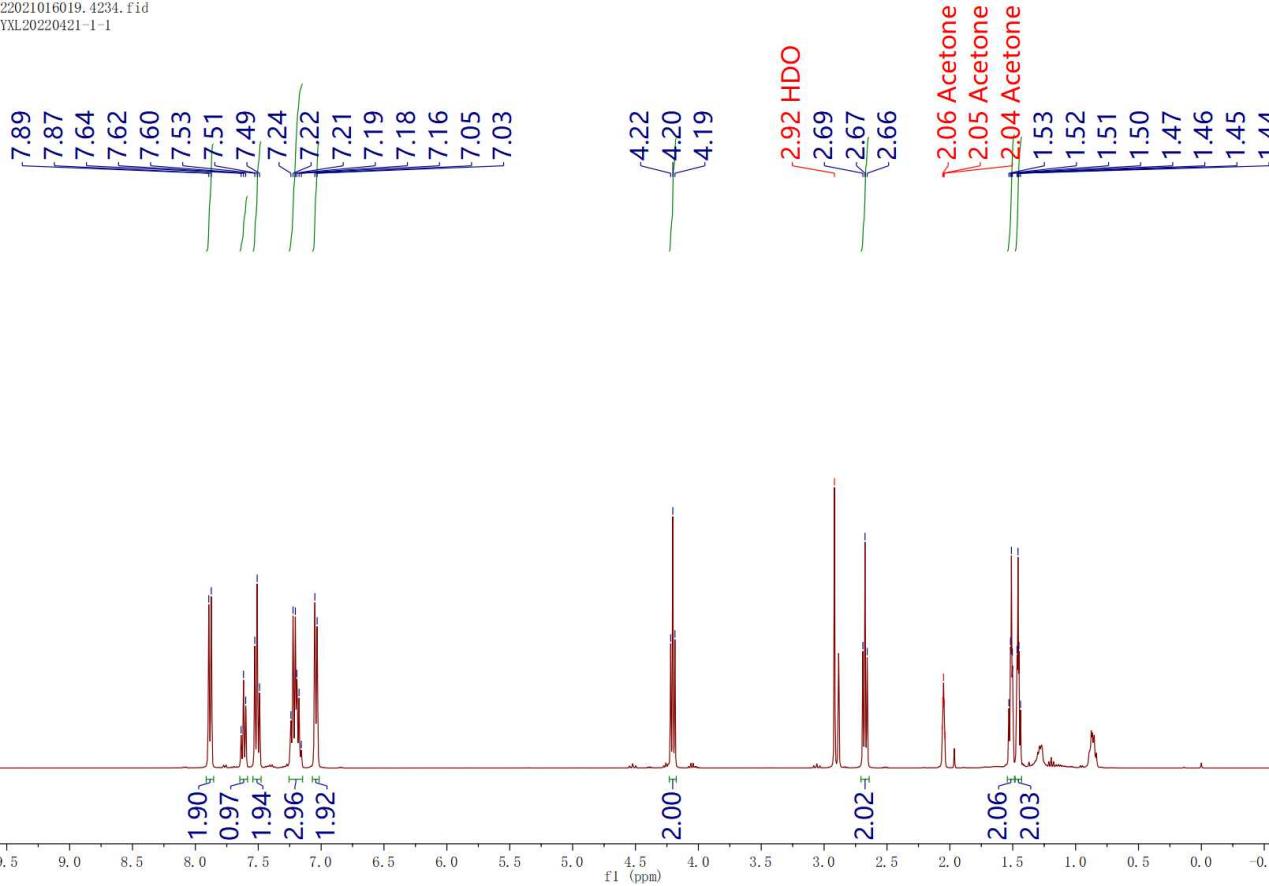


^1^H-NMR spectrum of compound (**3h**)


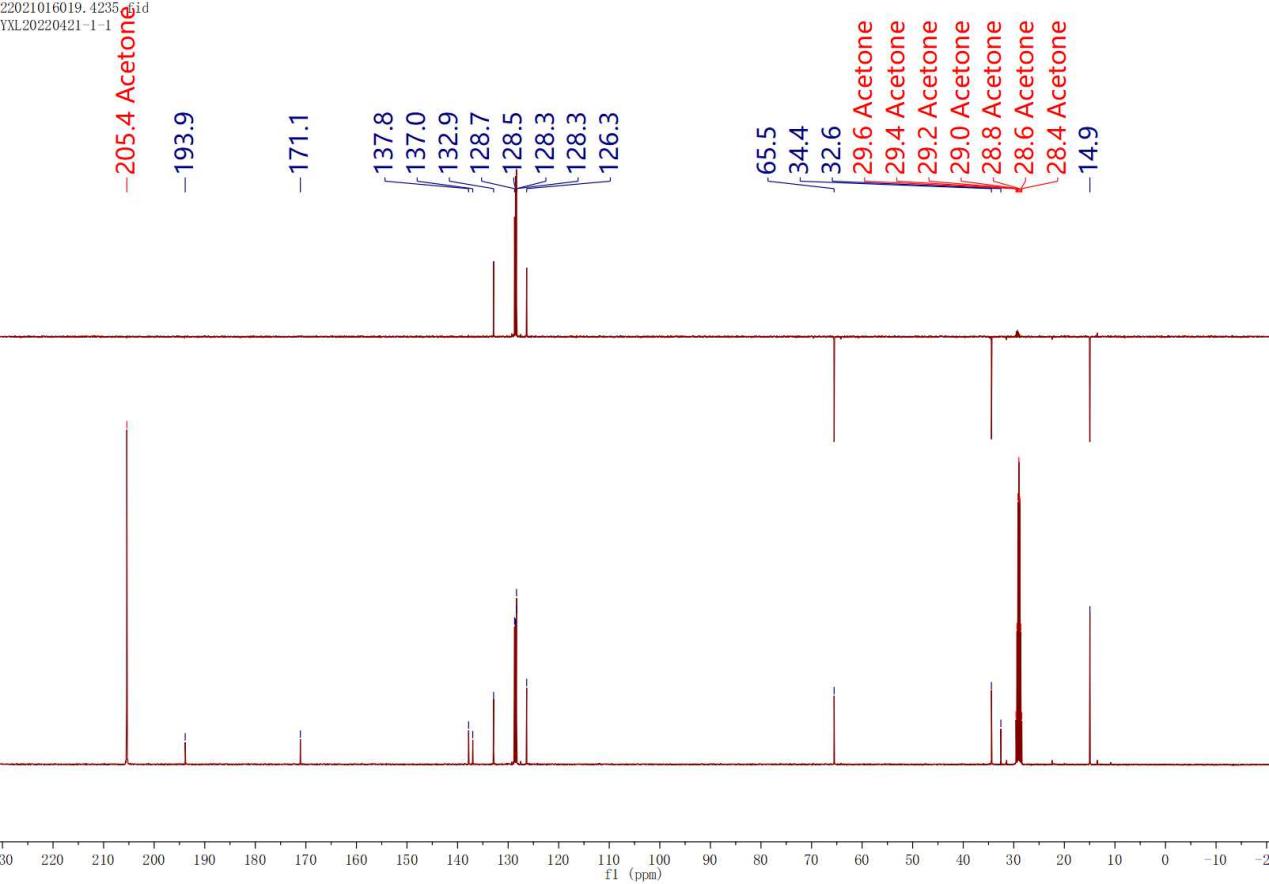


^13^C-NMR spectrum of compound (**3h**)

^^
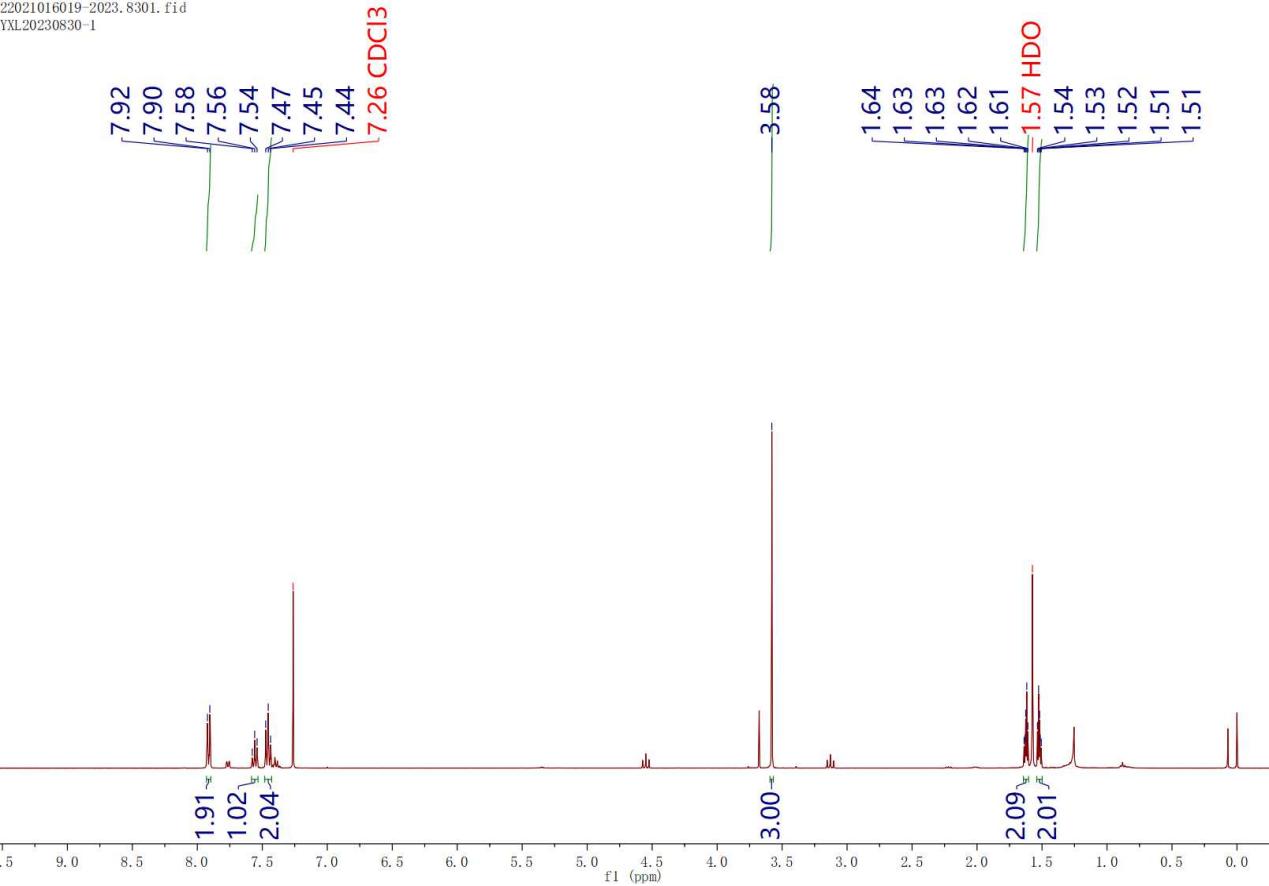


^1^H-NMR spectrum of compound (**3i**)


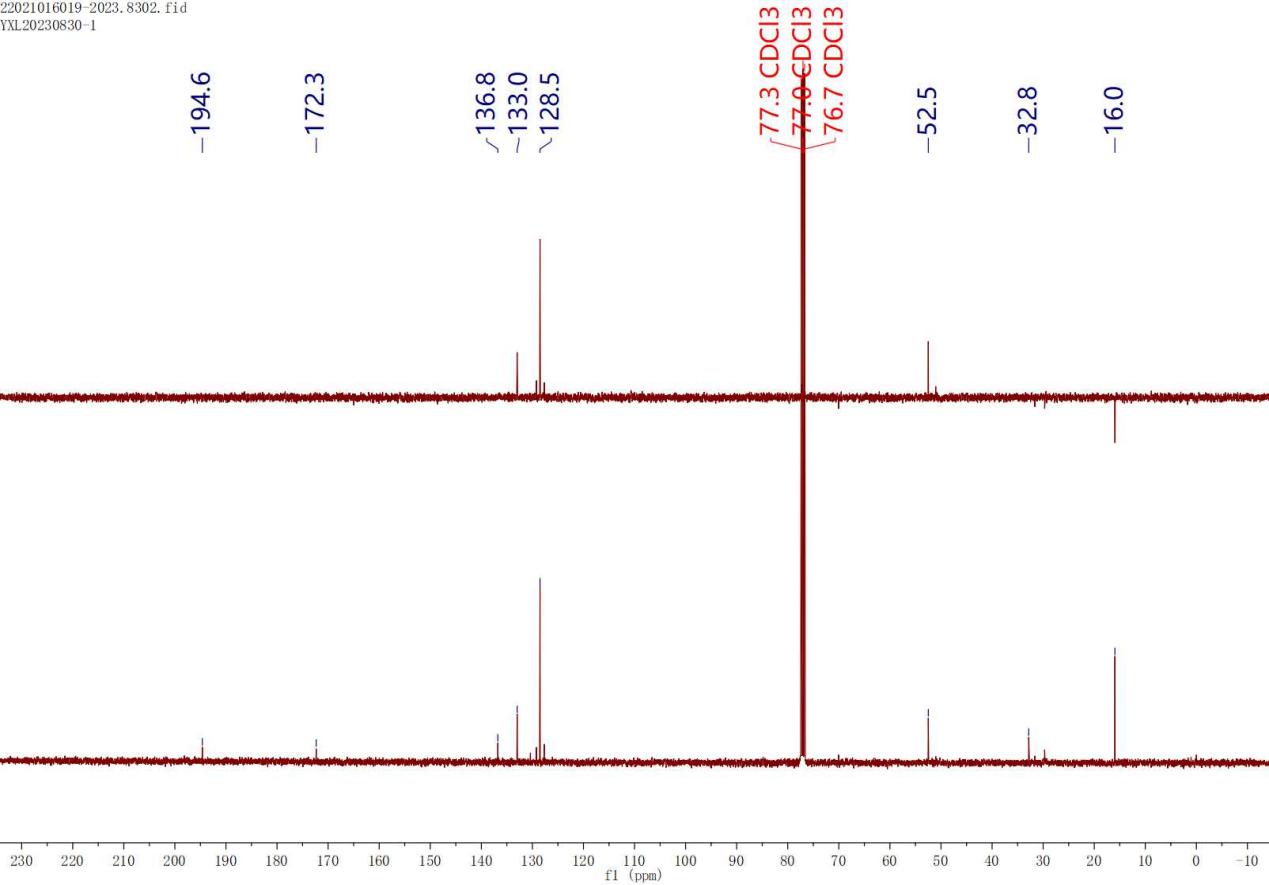


^13^C-NMR spectrum of compound (**3i**)

^^
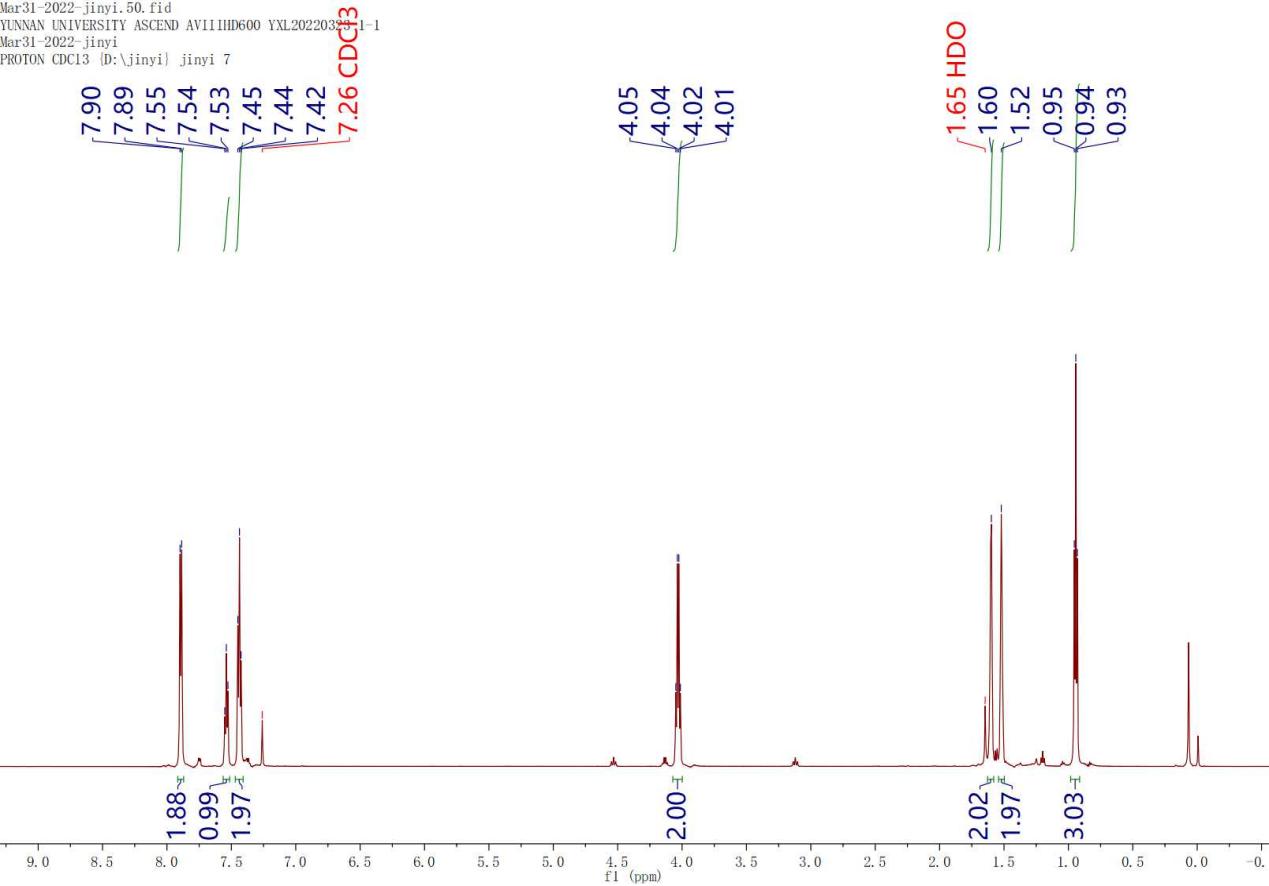


^1^H-NMR spectrum of compound (**3j**)


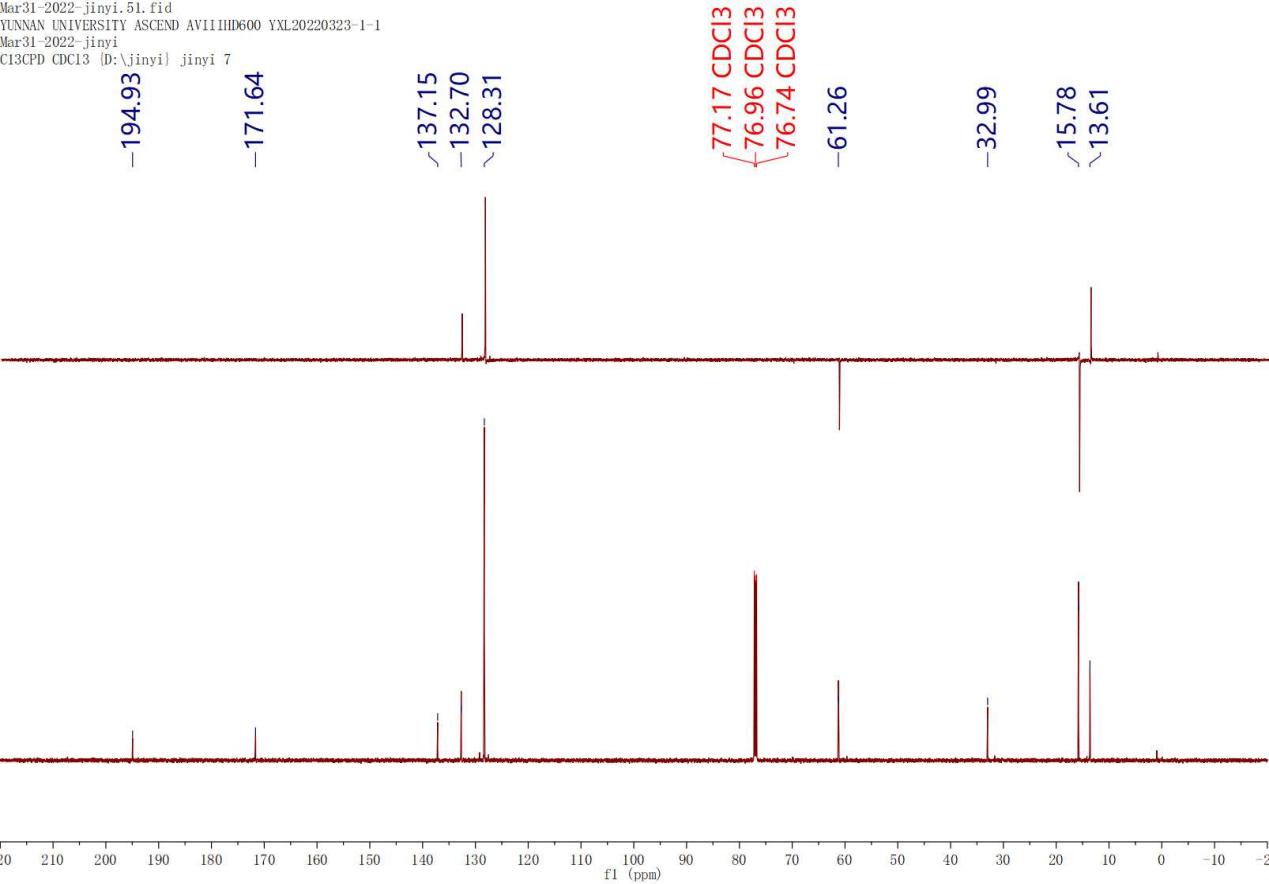


^13^C-NMR spectrum of compound (**3j**)

^^
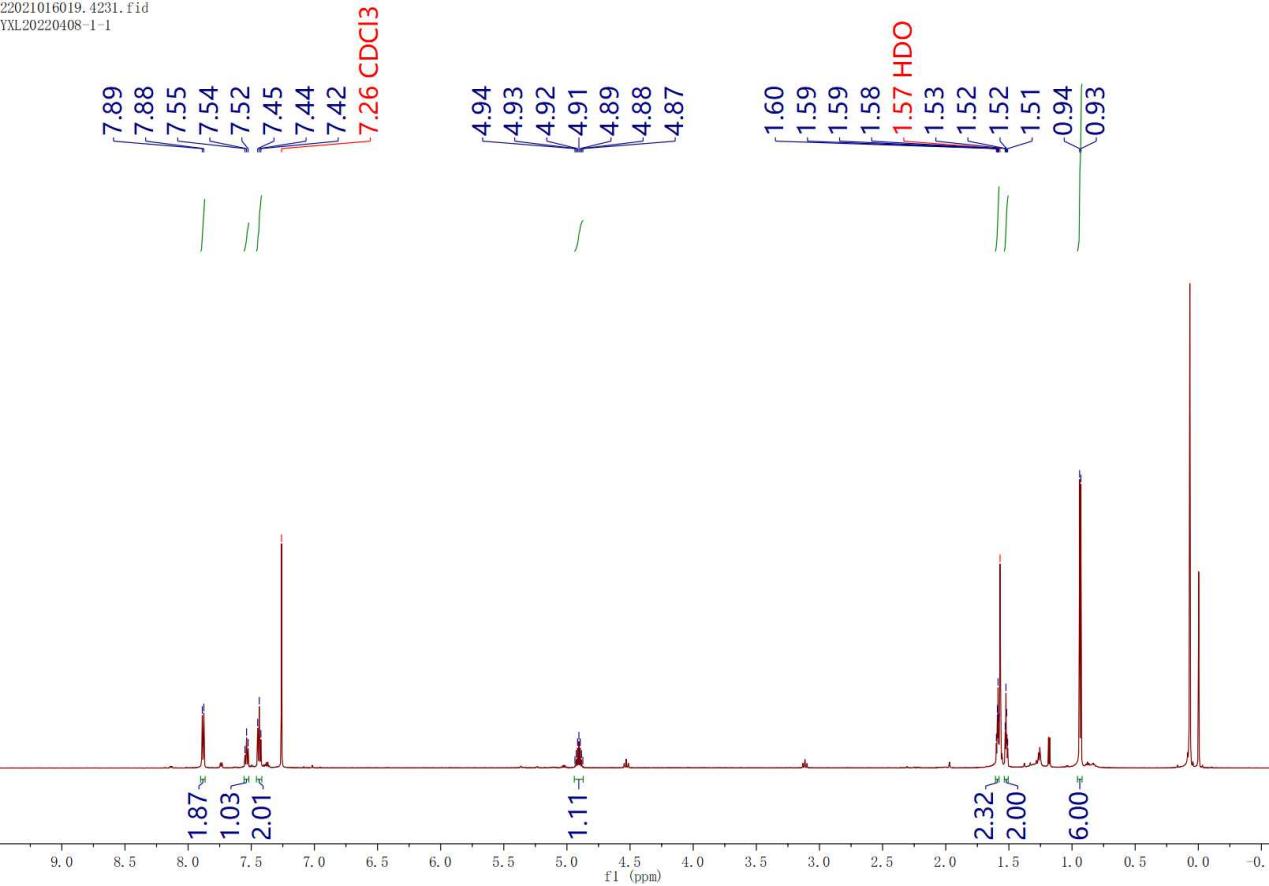


^1^H-NMR spectrum of compound (**3k**)


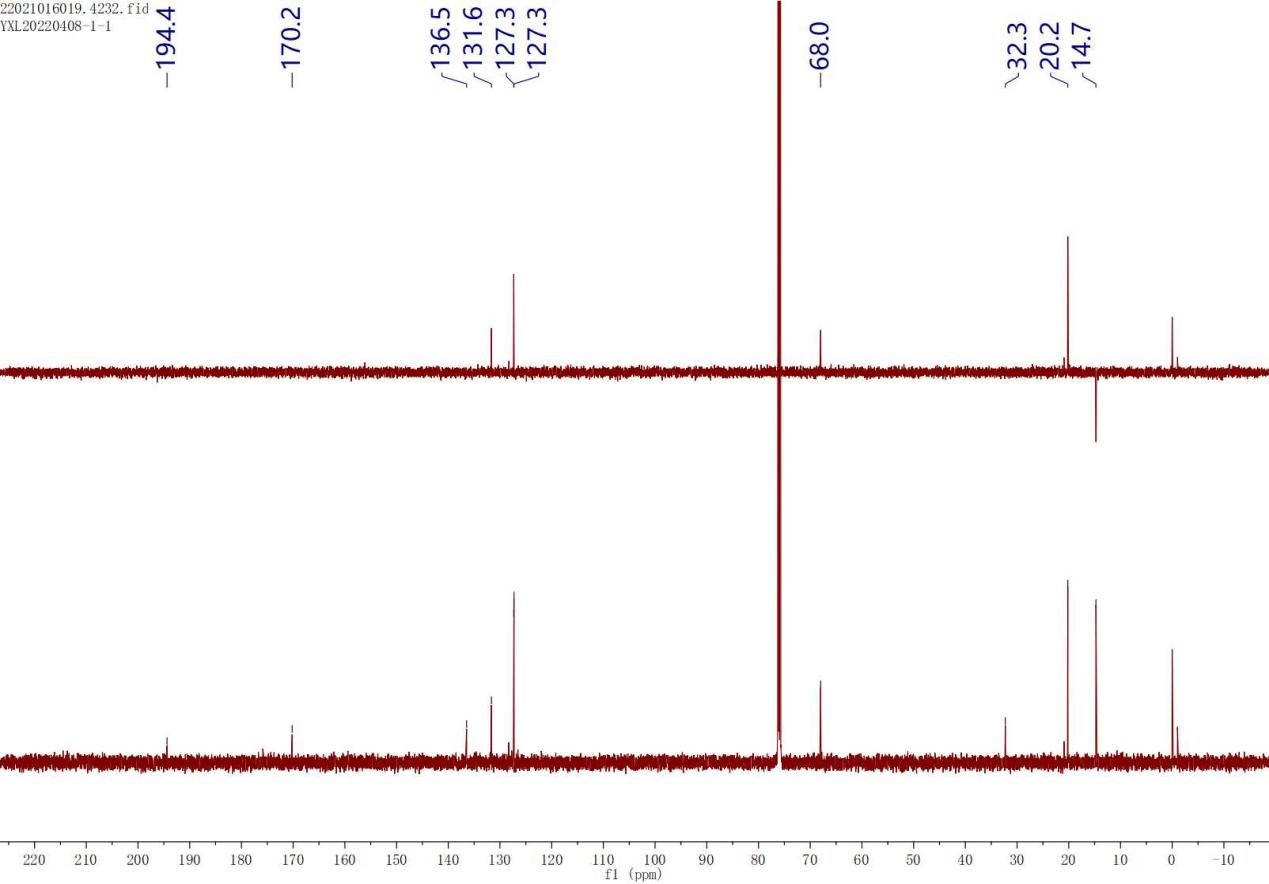


^13^C-NMR spectrum of compound (**3k**)

^^
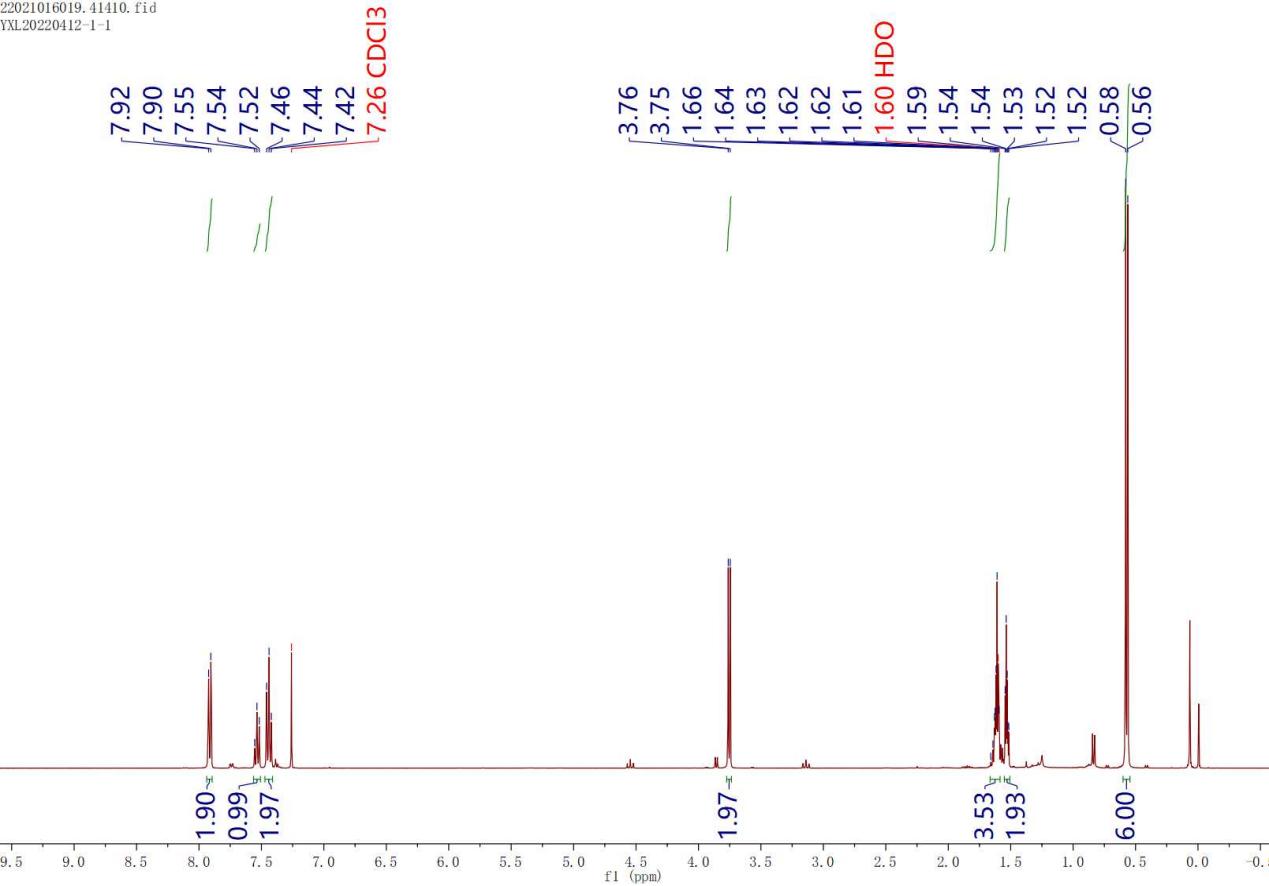


^1^H-NMR spectrum of compound (**3l**)


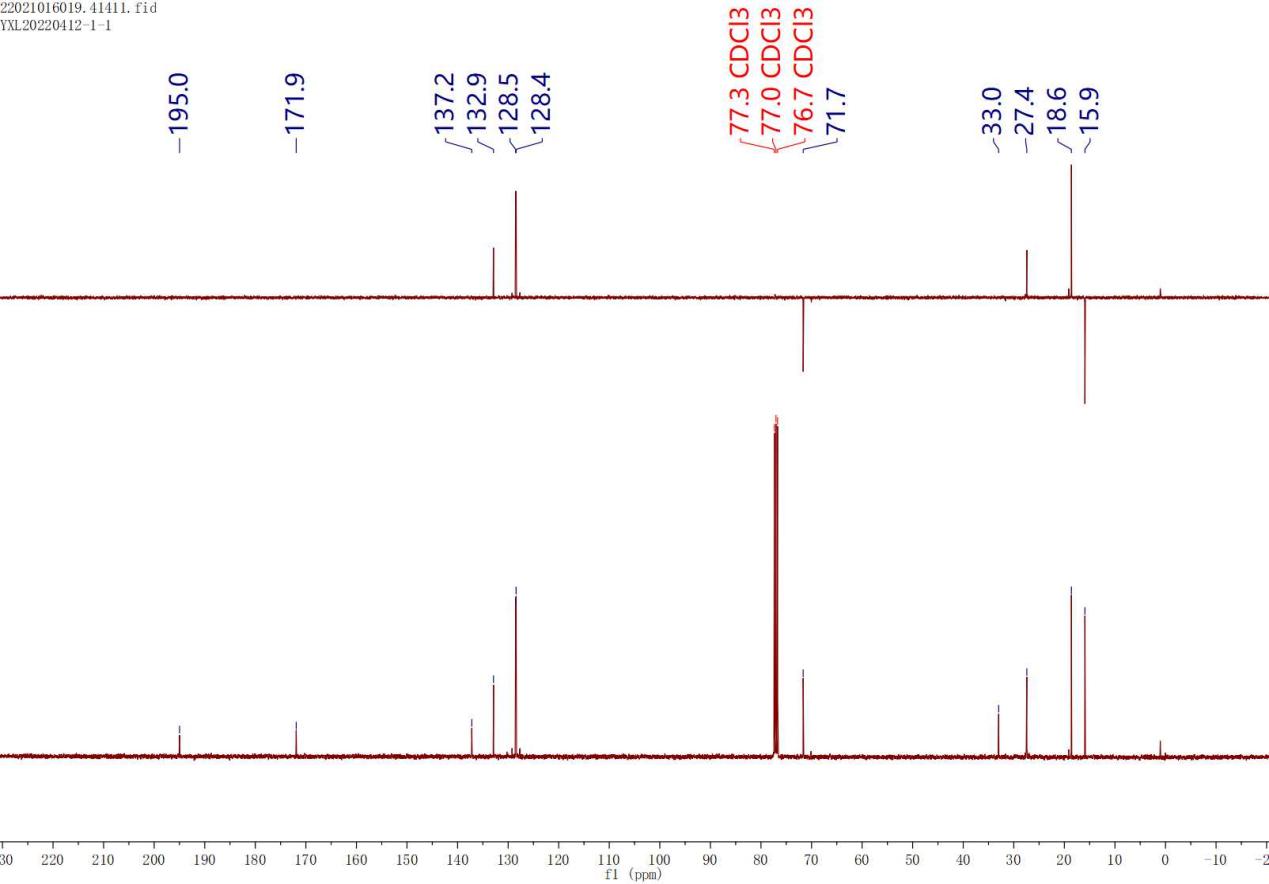


^13^C-NMR spectrum of compound (**3l**)

^^
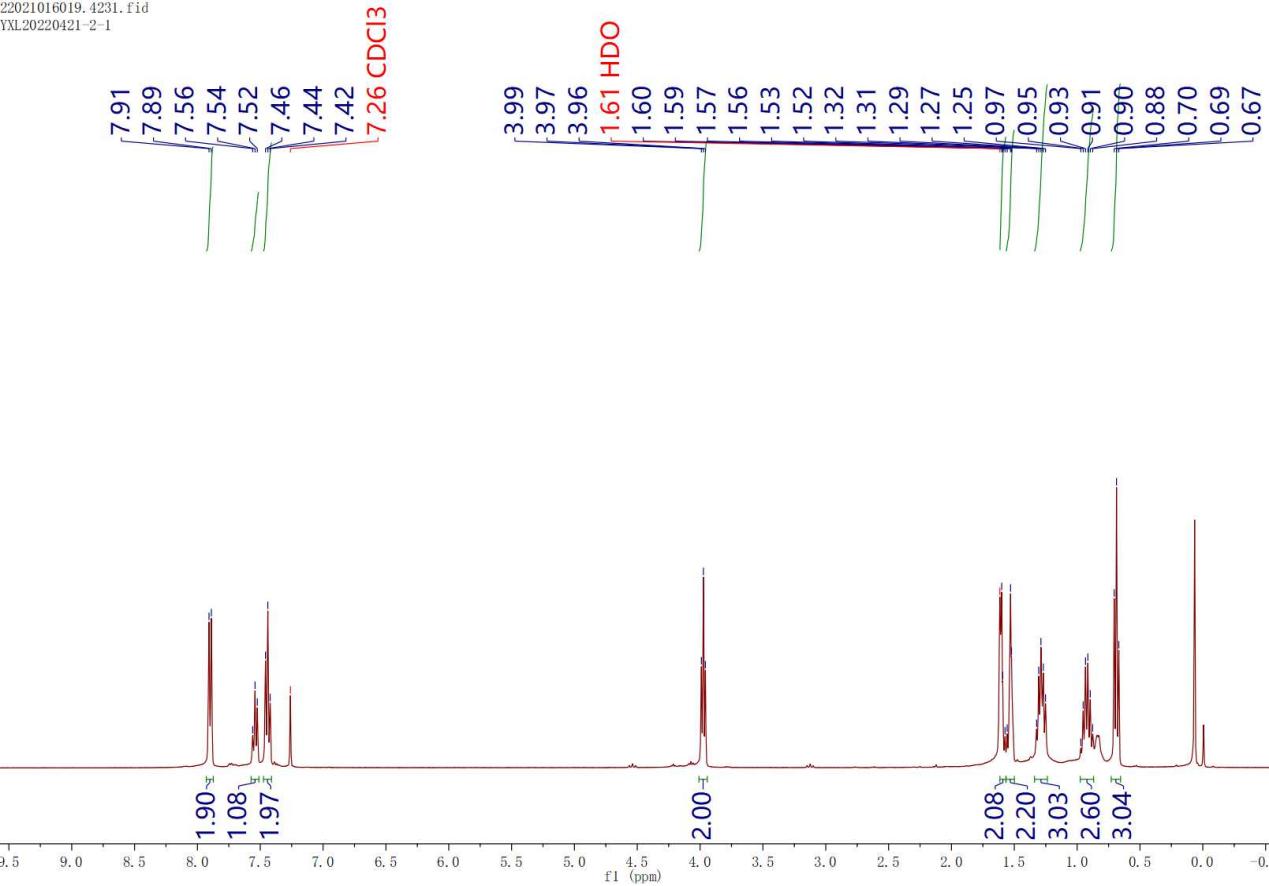


^1^H-NMR spectrum of compound (**3m**)


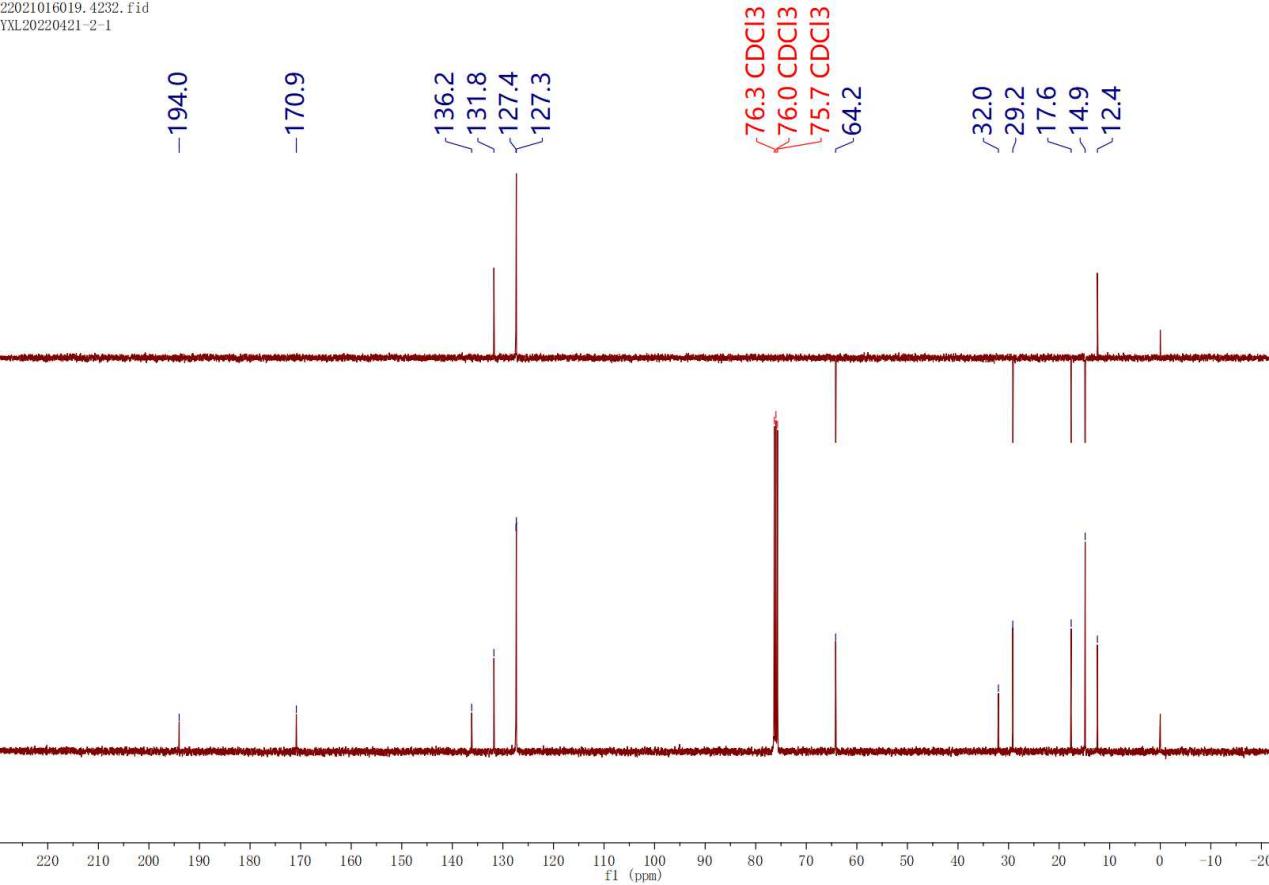


^13^C-NMR spectrum of compound (**3m**)

^^
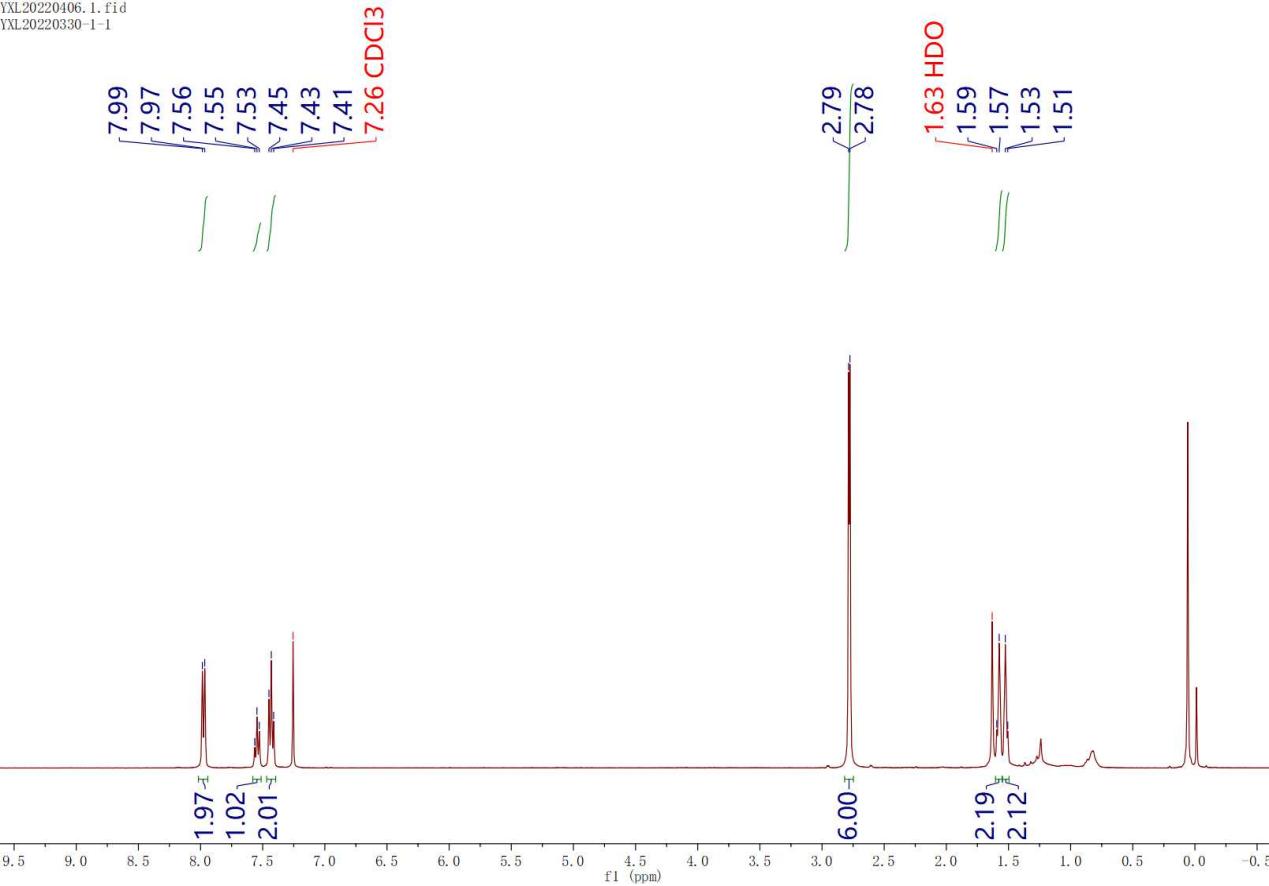


^1^H-NMR spectrum of compound (**3n**)


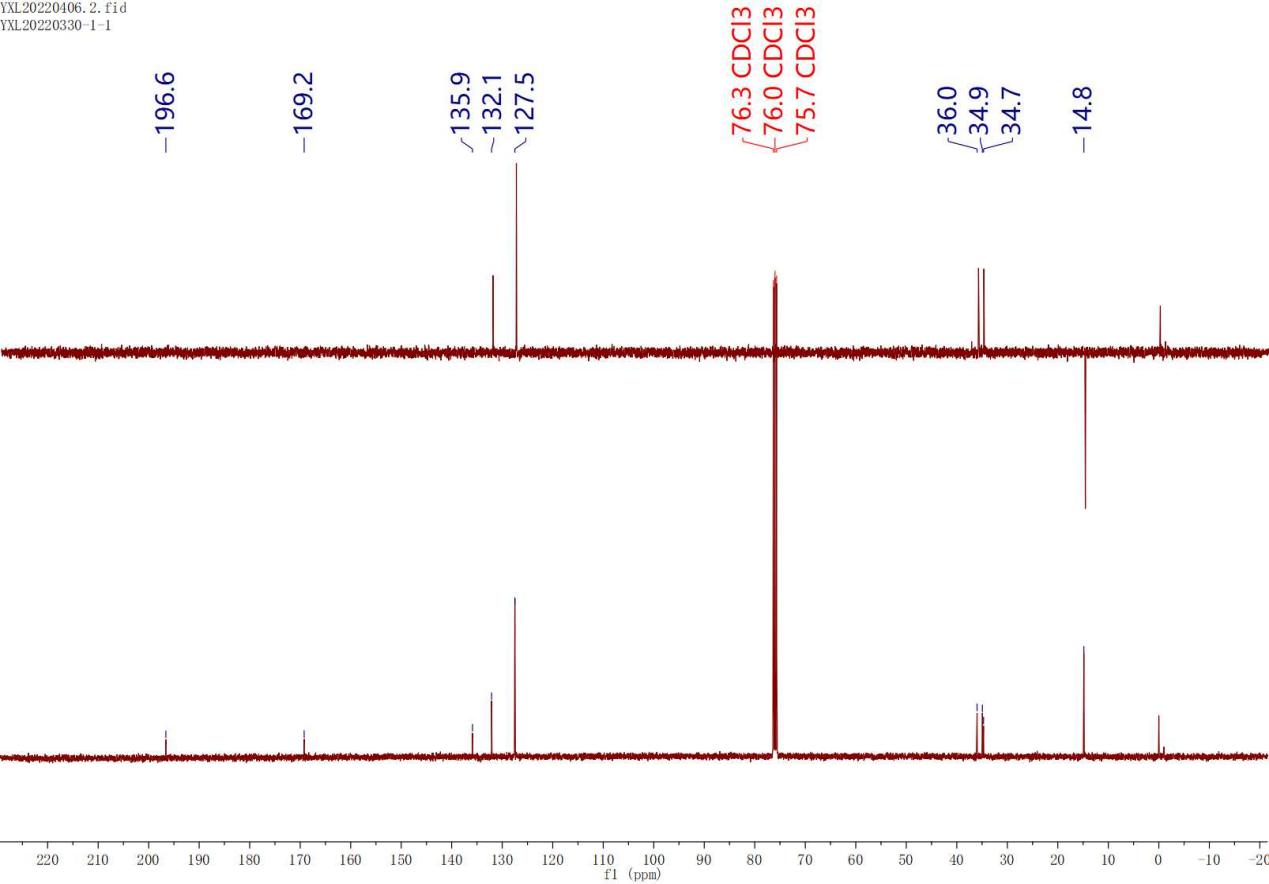


^13^C-NMR spectrum of compound (**3n**)

^^
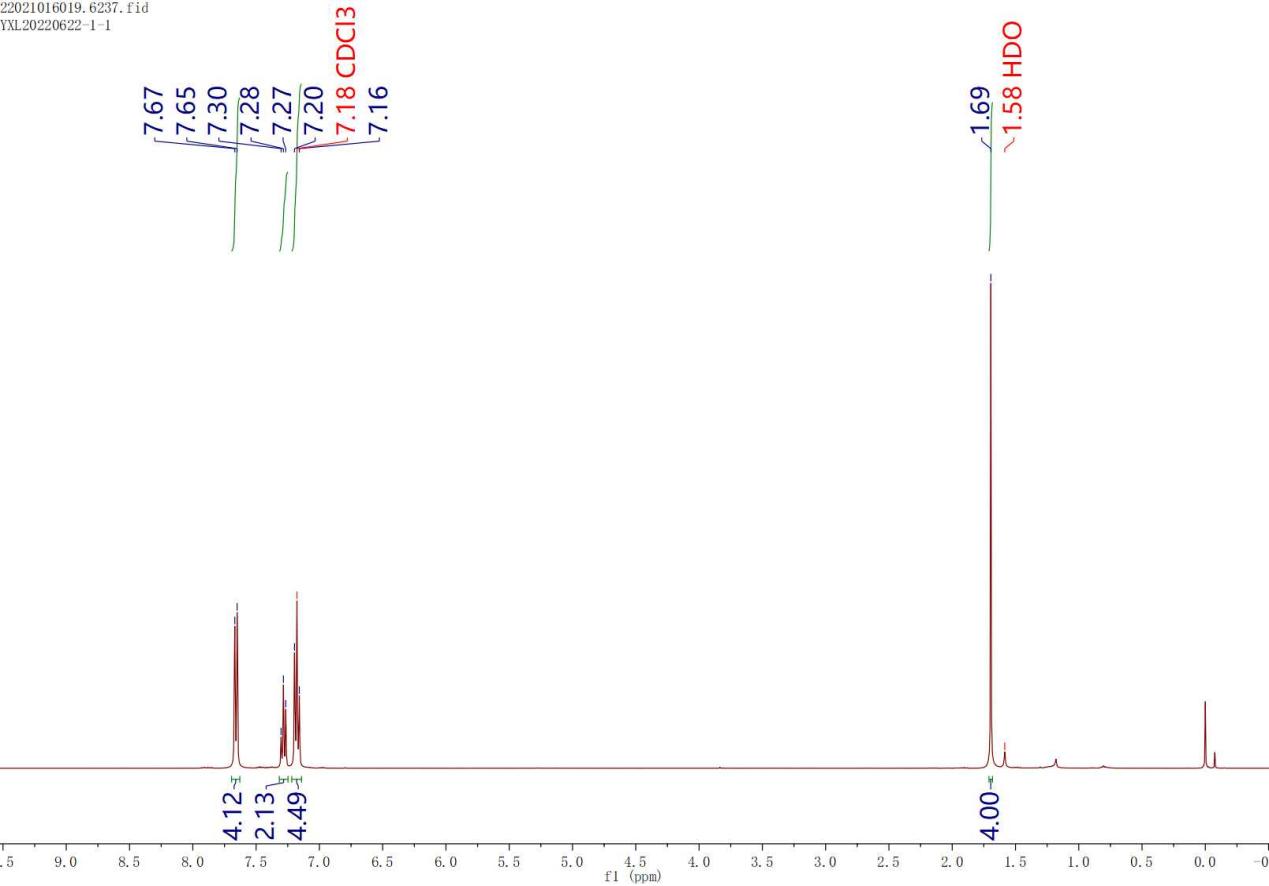


^1^H-NMR spectrum of compound (**3o**)


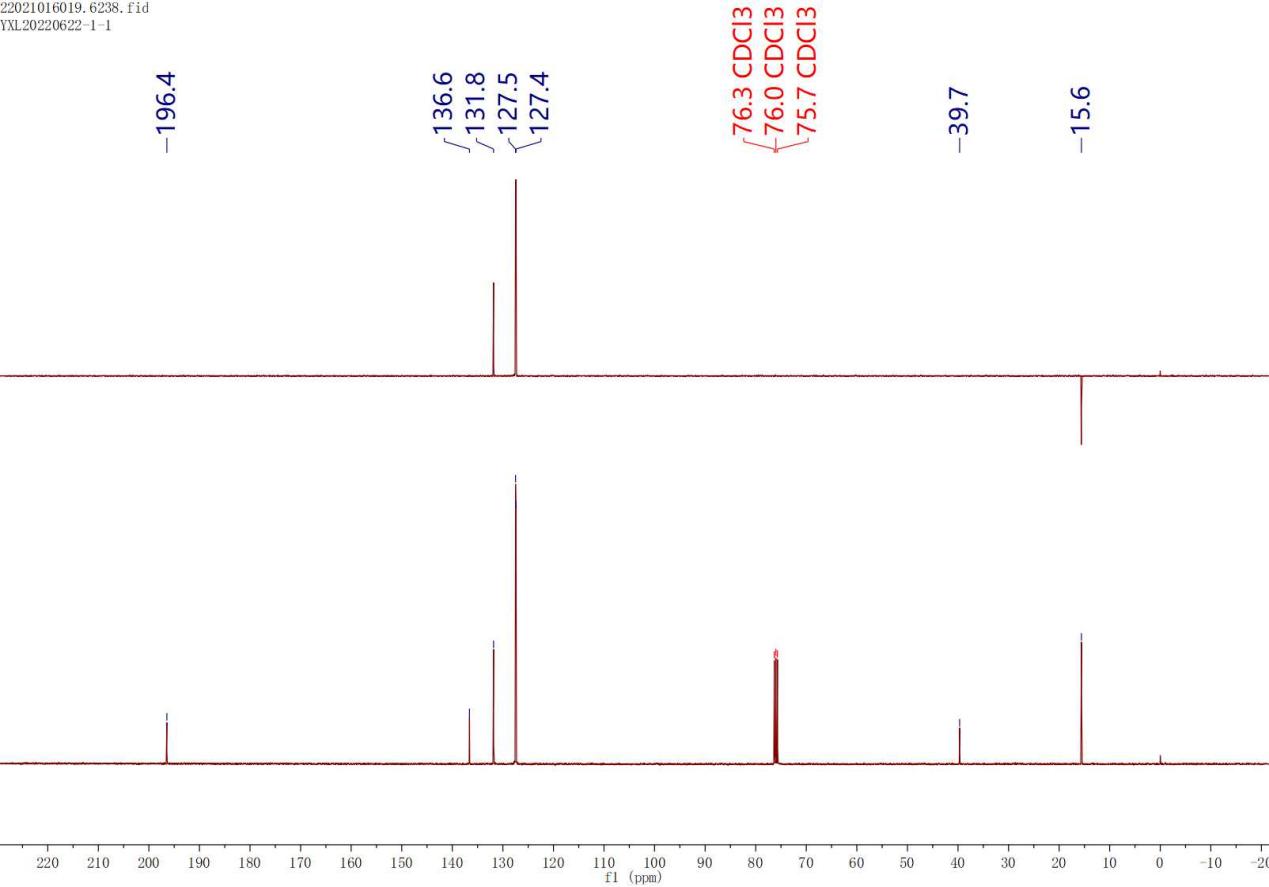


^13^C-NMR spectrum of compound (**3o**)

^^
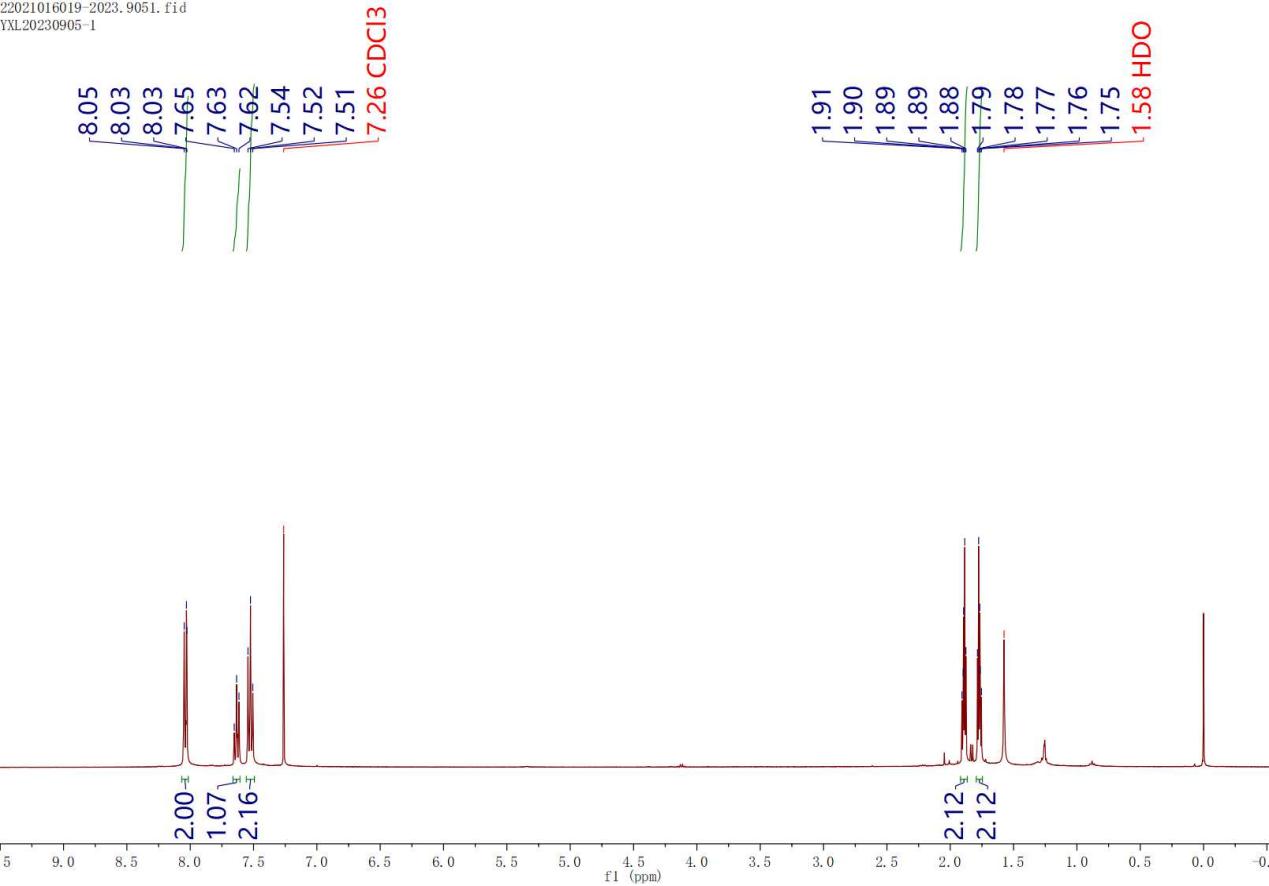


^1^H-NMR spectrum of compound (**3p**)


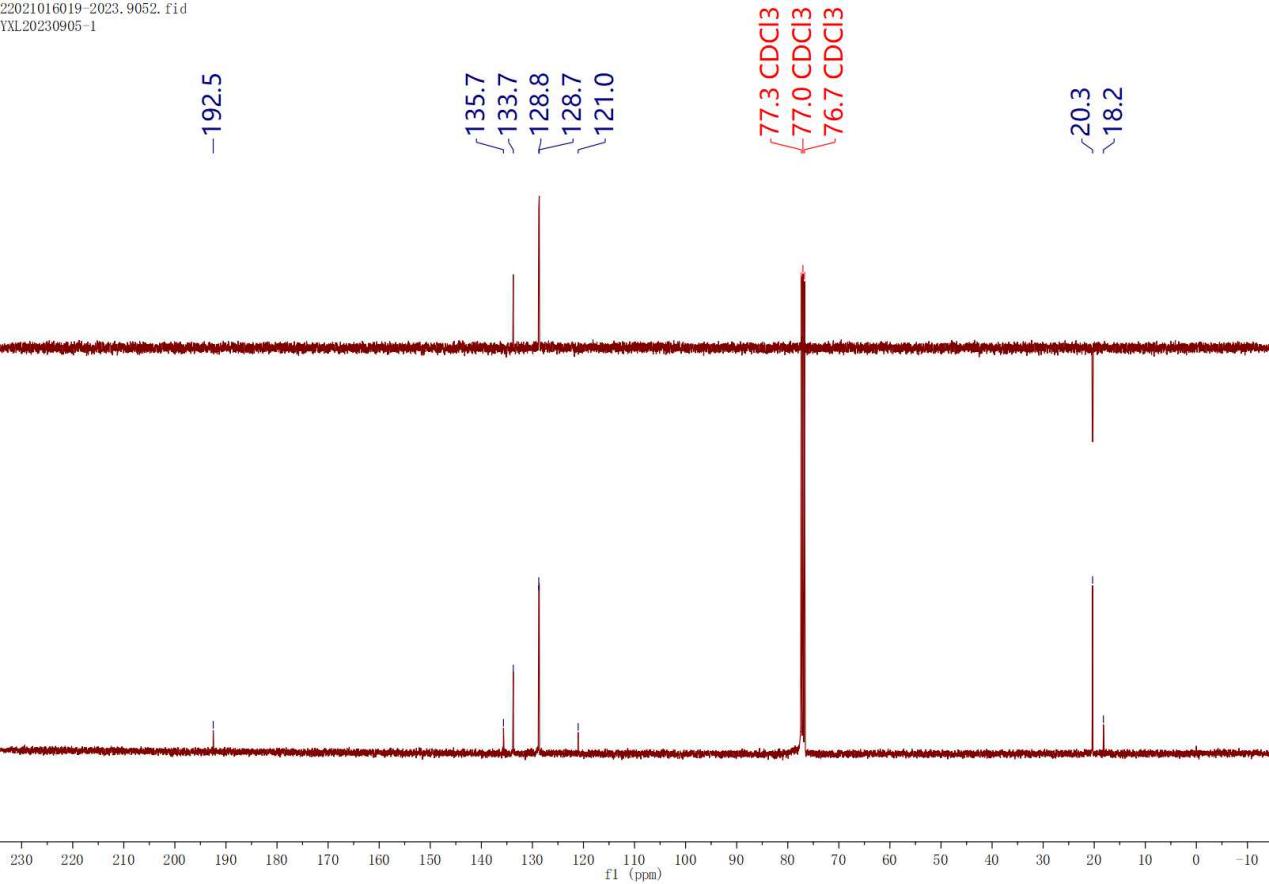


^13^C-NMR spectrum of compound (**3p**)

^^
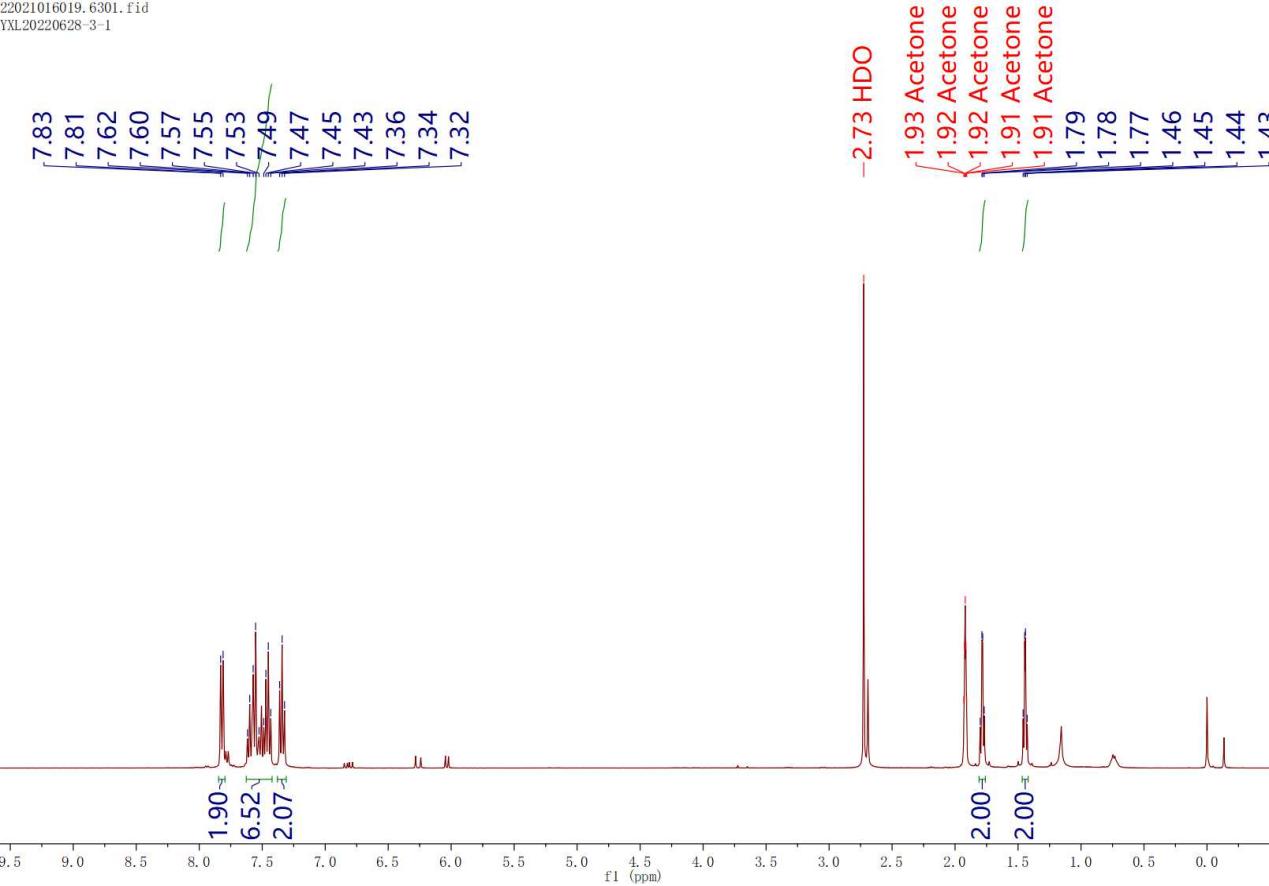


^1^H-NMR spectrum of compound (**3q**)


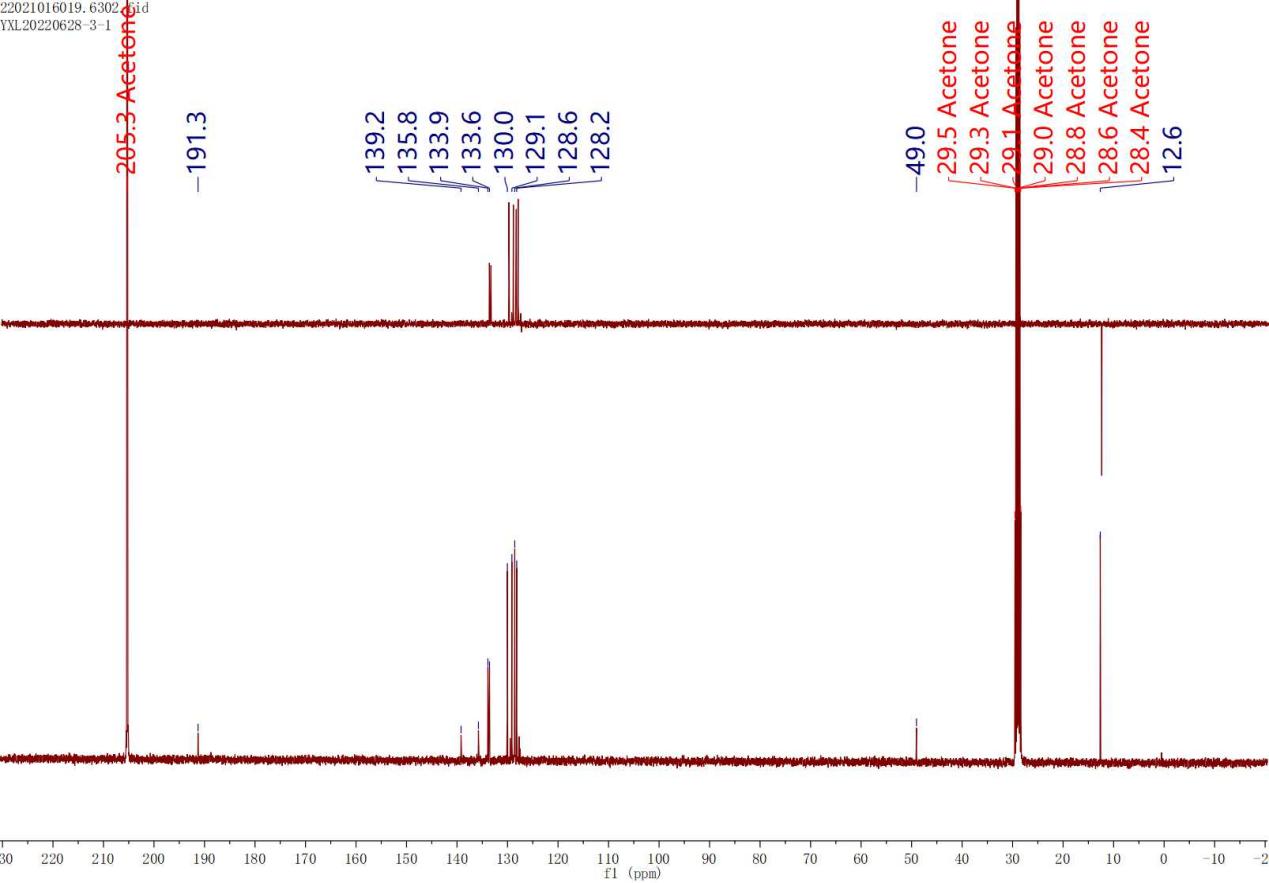


^13^C-NMR spectrum of compound (**3q**)

^^
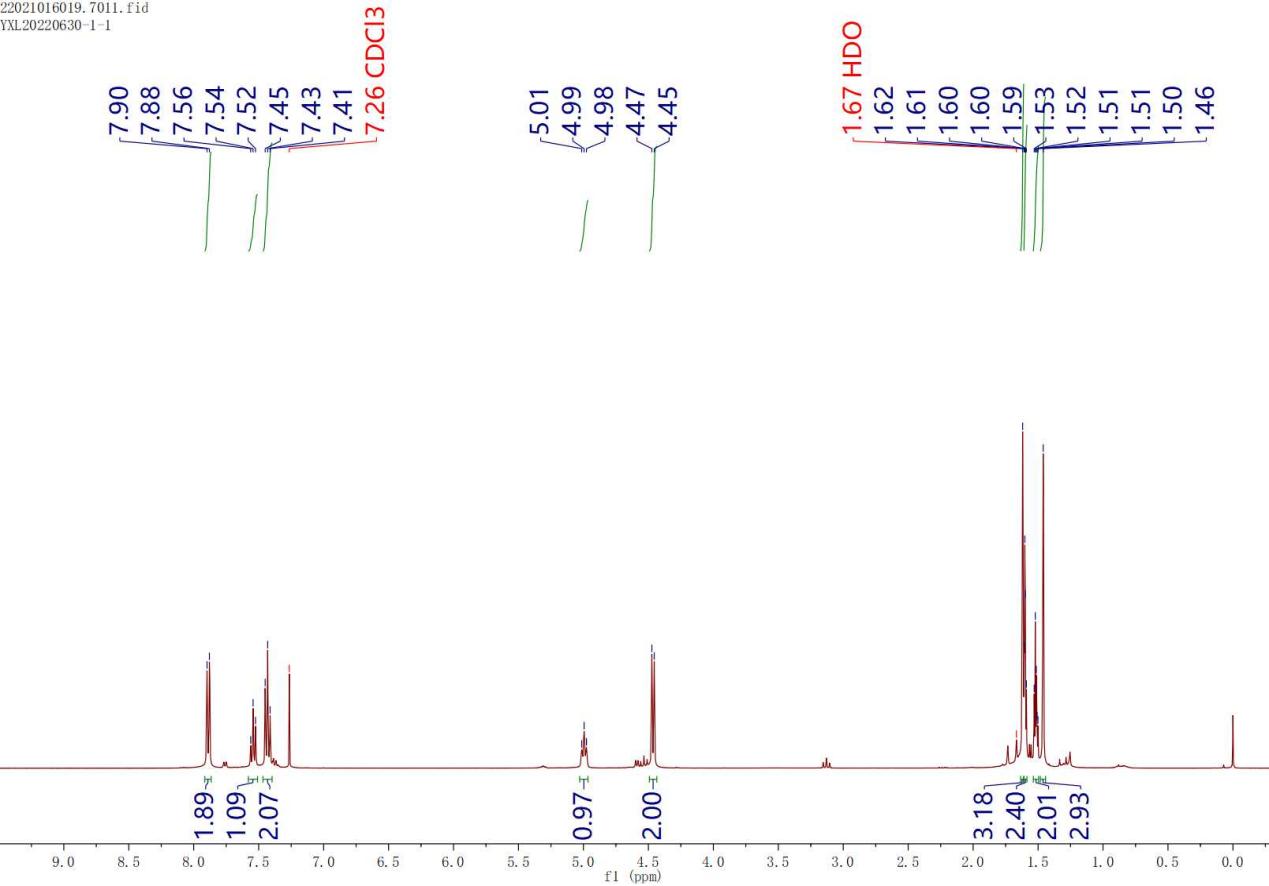


^1^H-NMR spectrum of compound (**3r**)


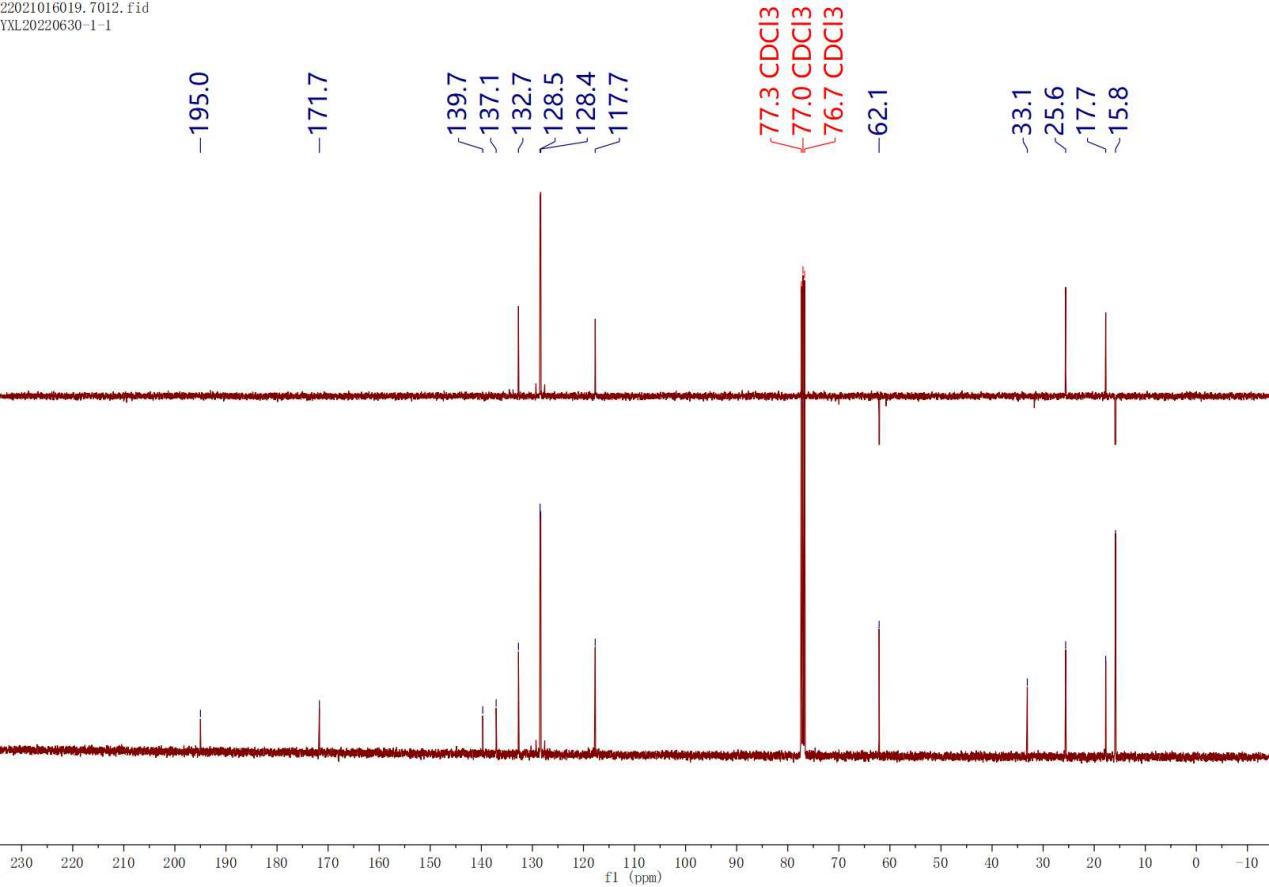


^13^C-NMR spectrum of compound (**3r**)

^^

^1^H-NMR spectrum of compound (**3s**)

^13^C-NMR spectrum of compound (**3s**)

^^

^1^H-NMR spectrum of compound (**3t**)

^13^C-NMR spectrum of compound (**3t**)

^^

^1^H-NMR spectrum of compound (**3u**)

^13^C-NMR spectrum of compound (**3u**)

^1^H-NMR spectrum of compound (**3v**)

^13^C-NMR spectrum of compound (**3v**)

^^
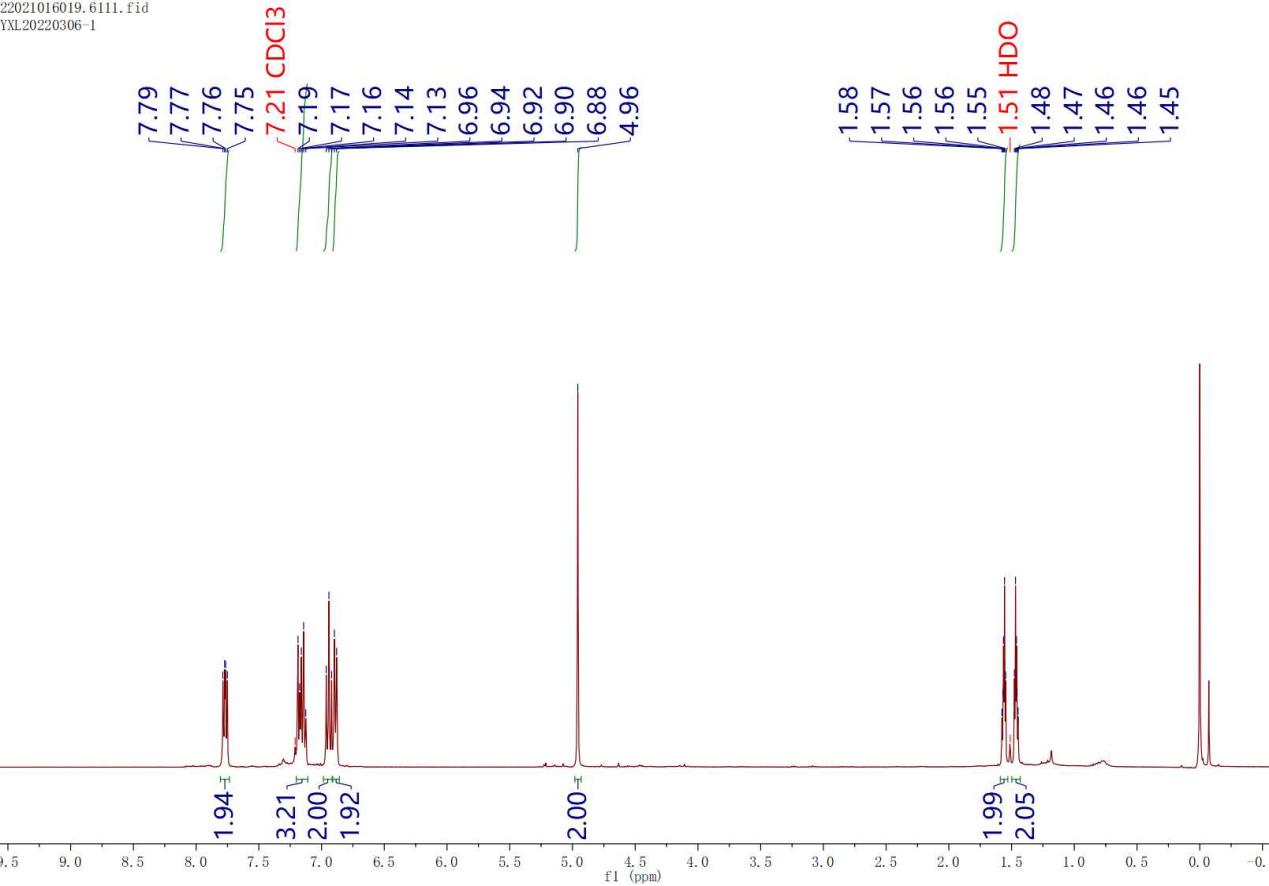


^1^H-NMR spectrum of compound (**4a**)


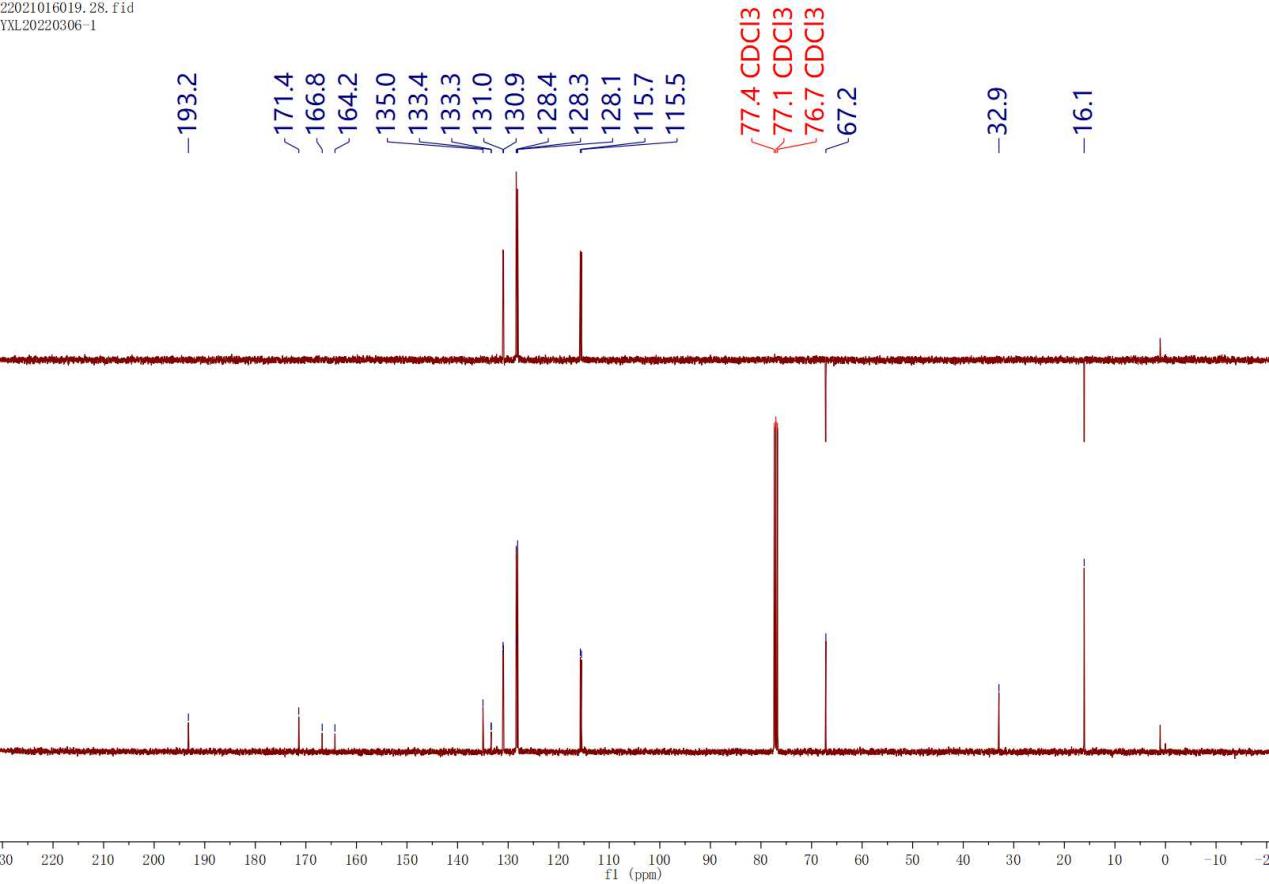


^13^C-NMR spectrum of compound (**4a**)


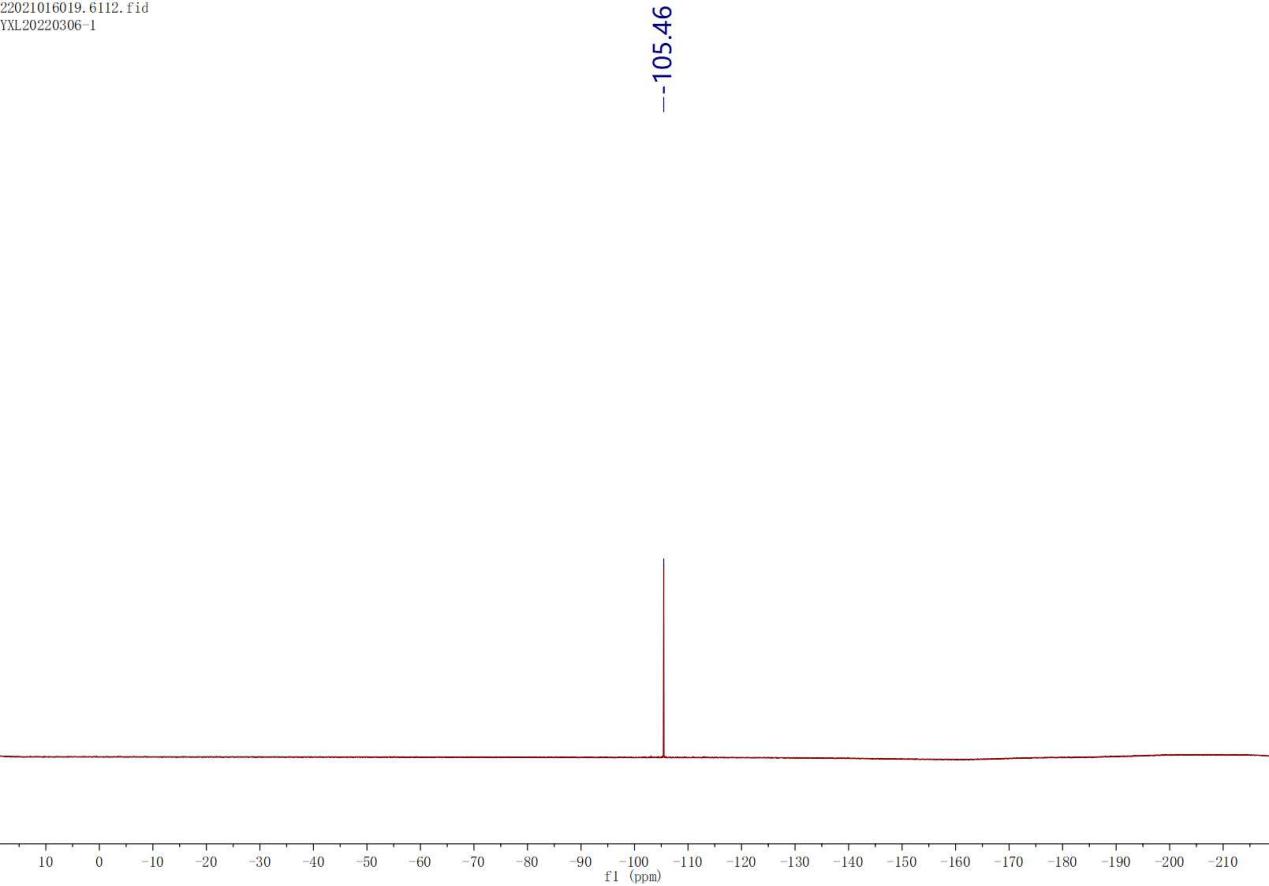


^19^F-NMR spectrum of compound (**4a**)

^^
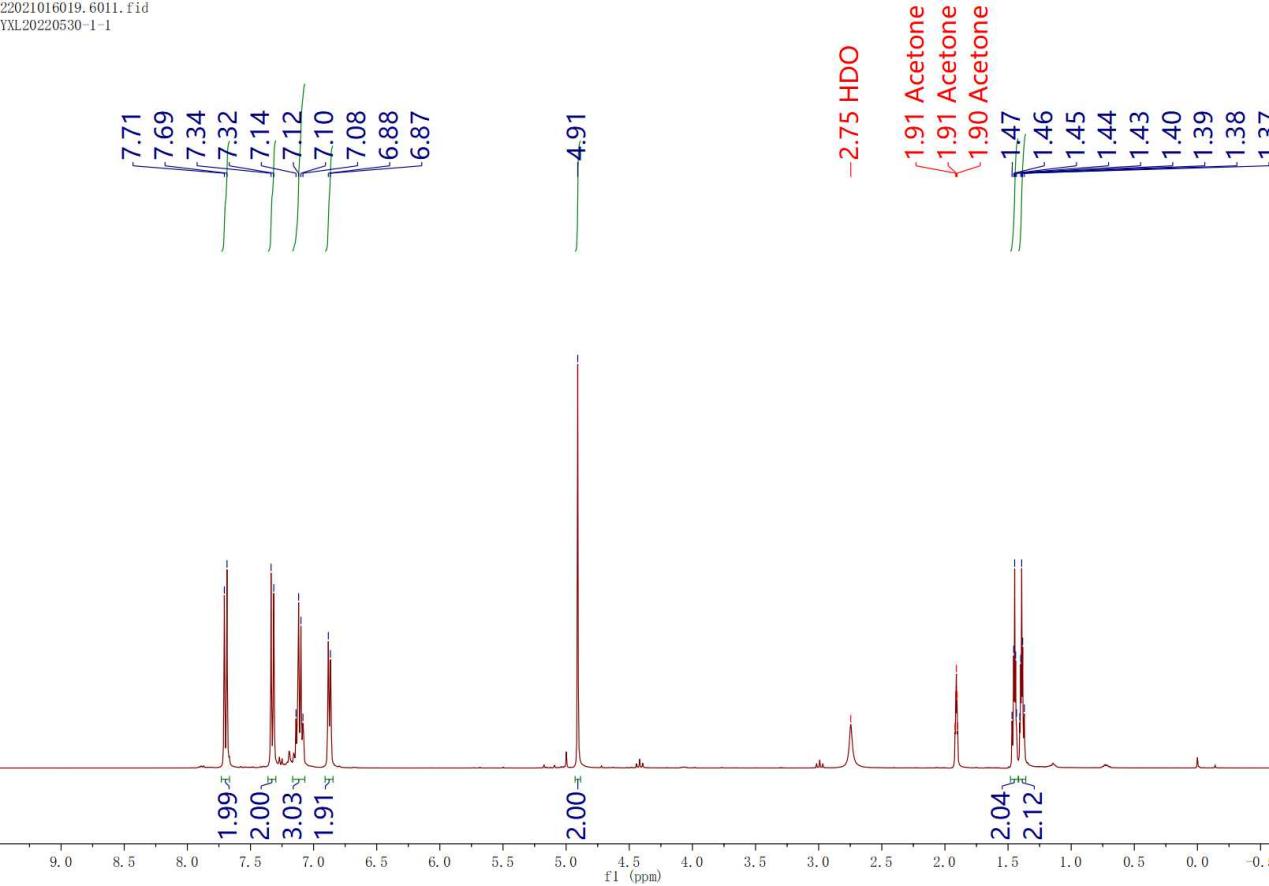


^1^H-NMR spectrum of compound (**4b**)


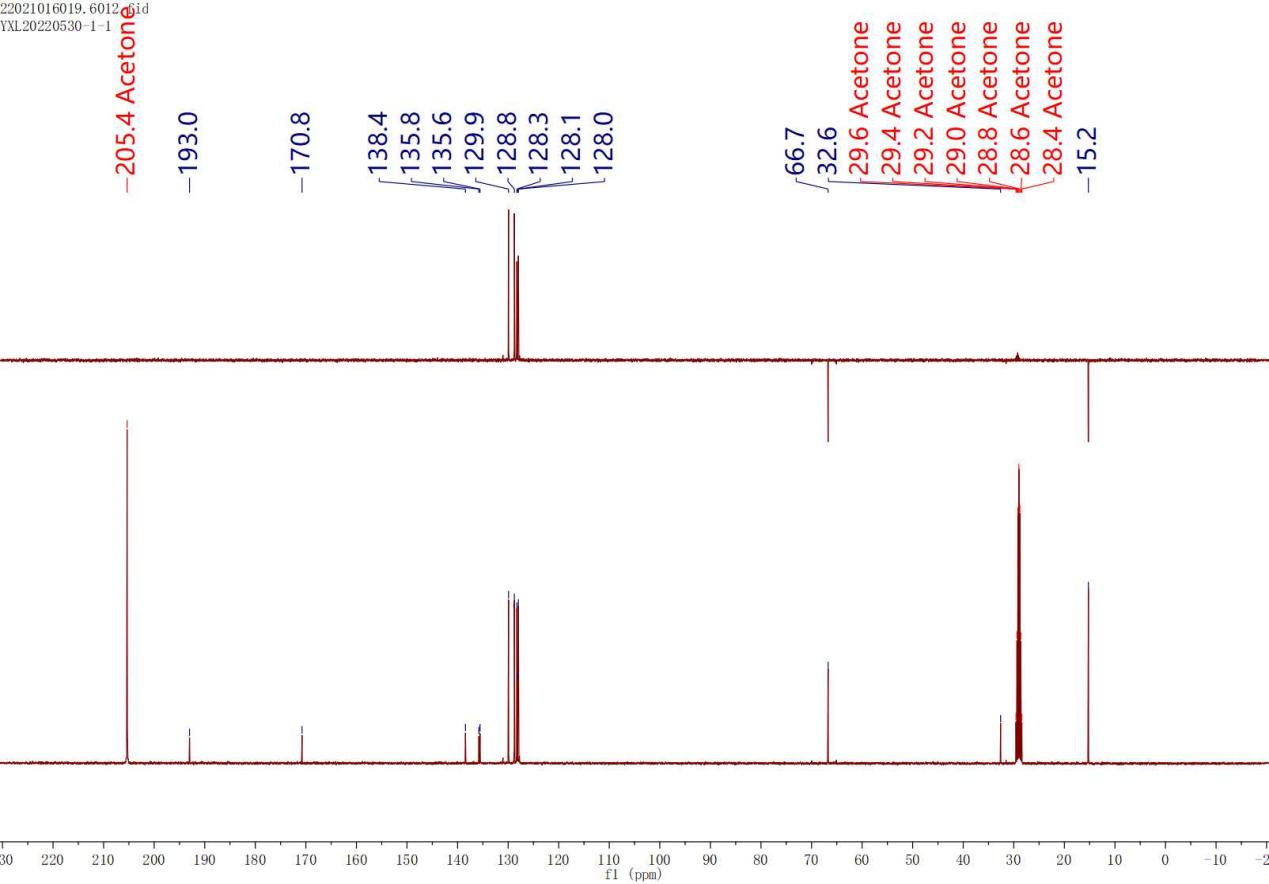


^13^C-NMR spectrum of compound (**4b**)

^^

^1^H-NMR spectrum of compound (**4c**)

^13^C-NMR spectrum of compound (**4c**)

^^

^1^H-NMR spectrum of compound (**4d**)

^13^C-NMR spectrum of compound (**4d**)


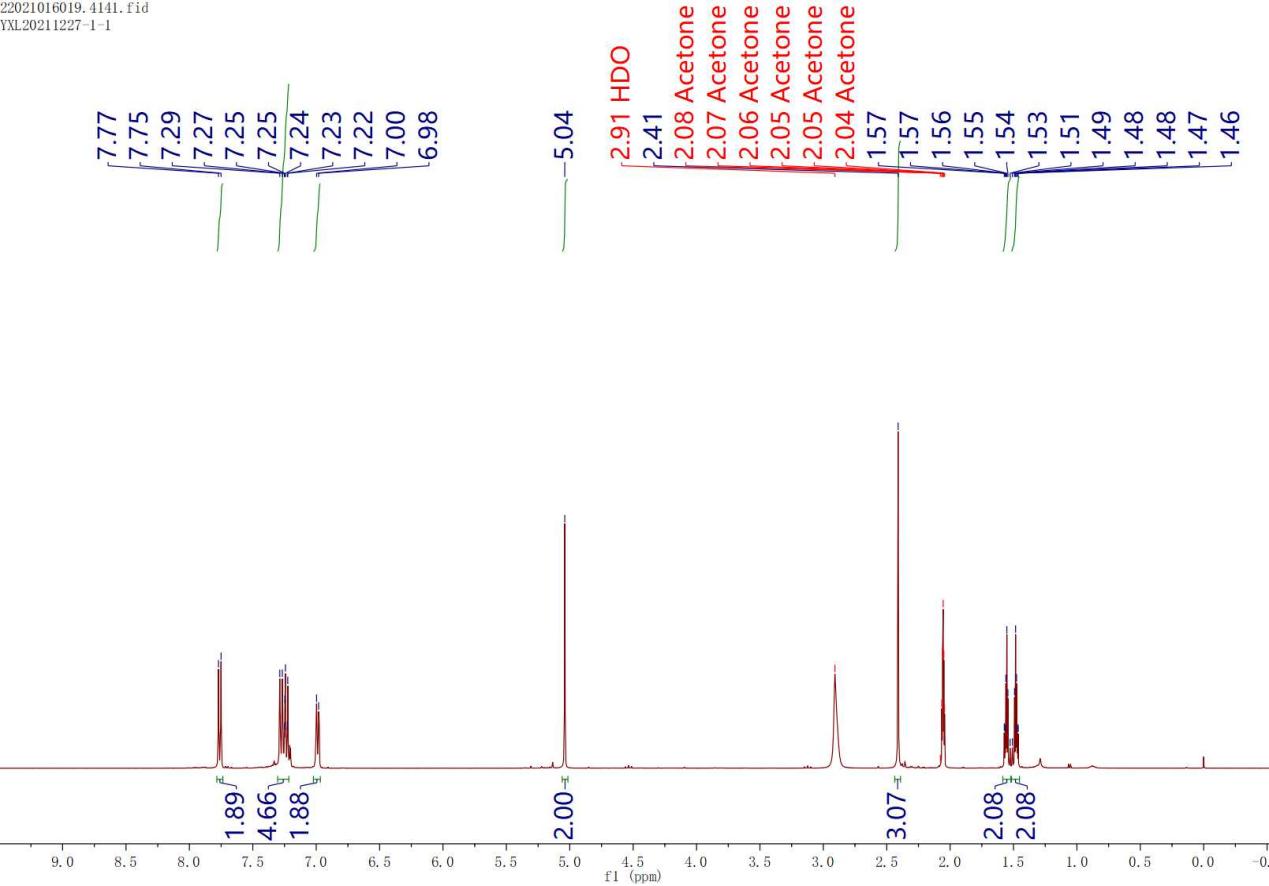


^1^H-NMR spectrum of compound (**4e**)


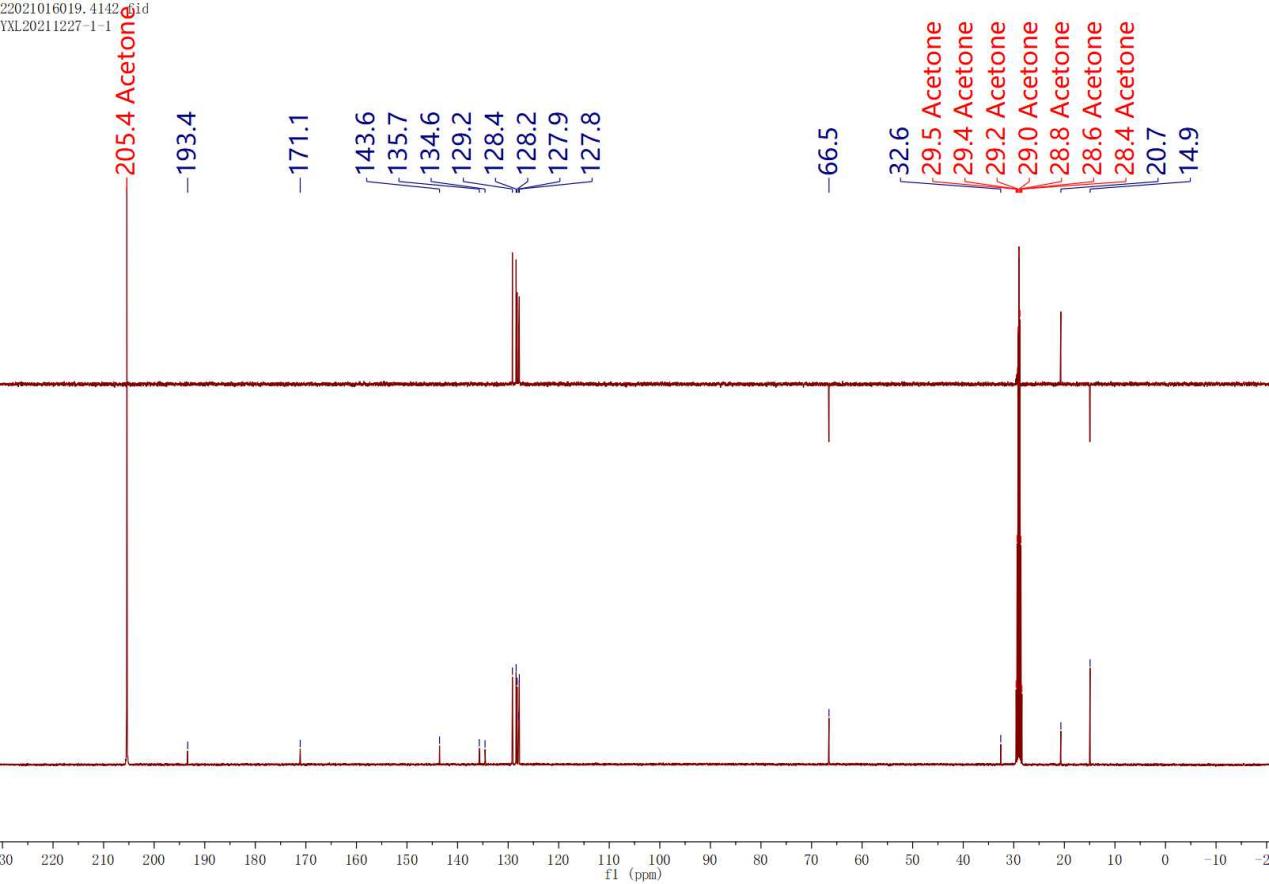


^13^C-NMR spectrum of compound (**4e**)


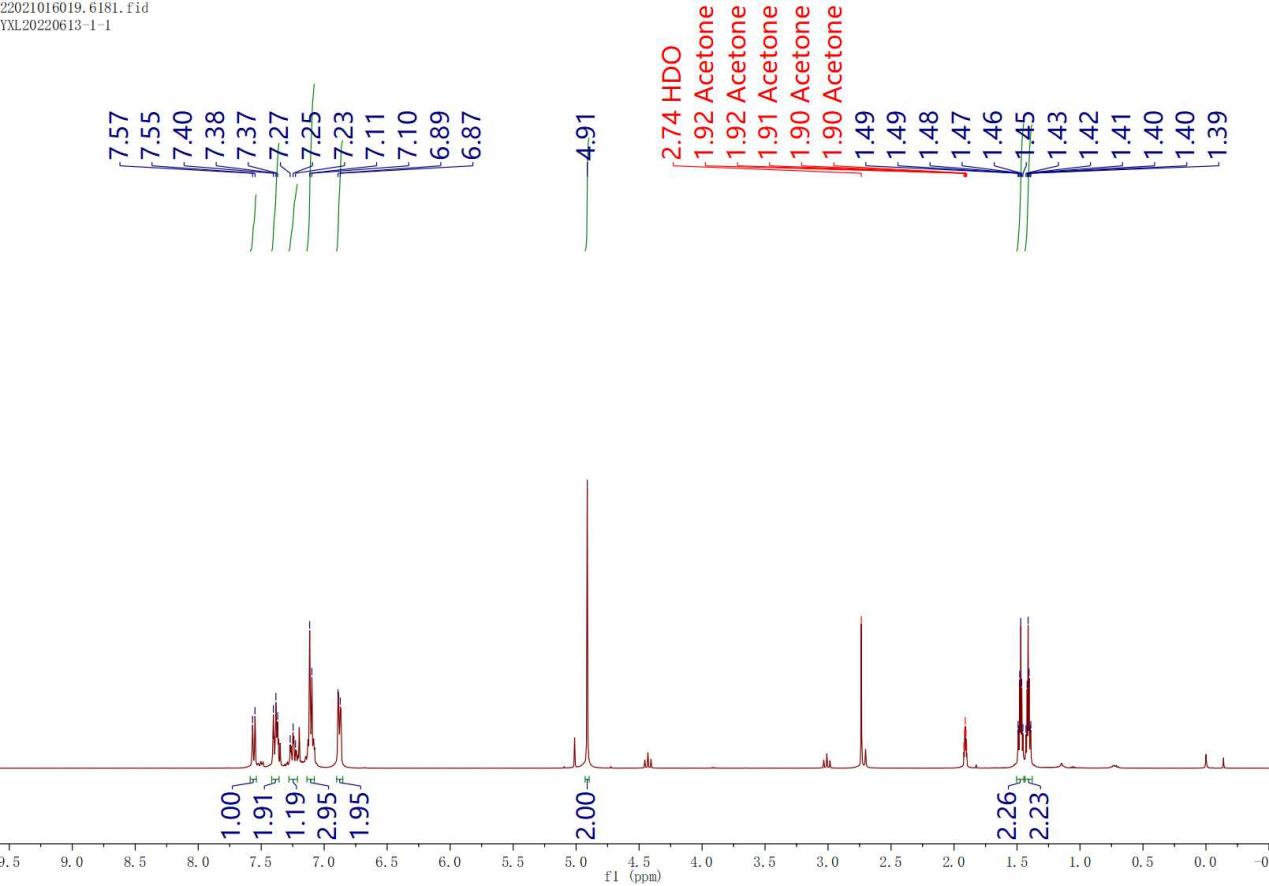


^1^H-NMR spectrum of compound (**4f**)


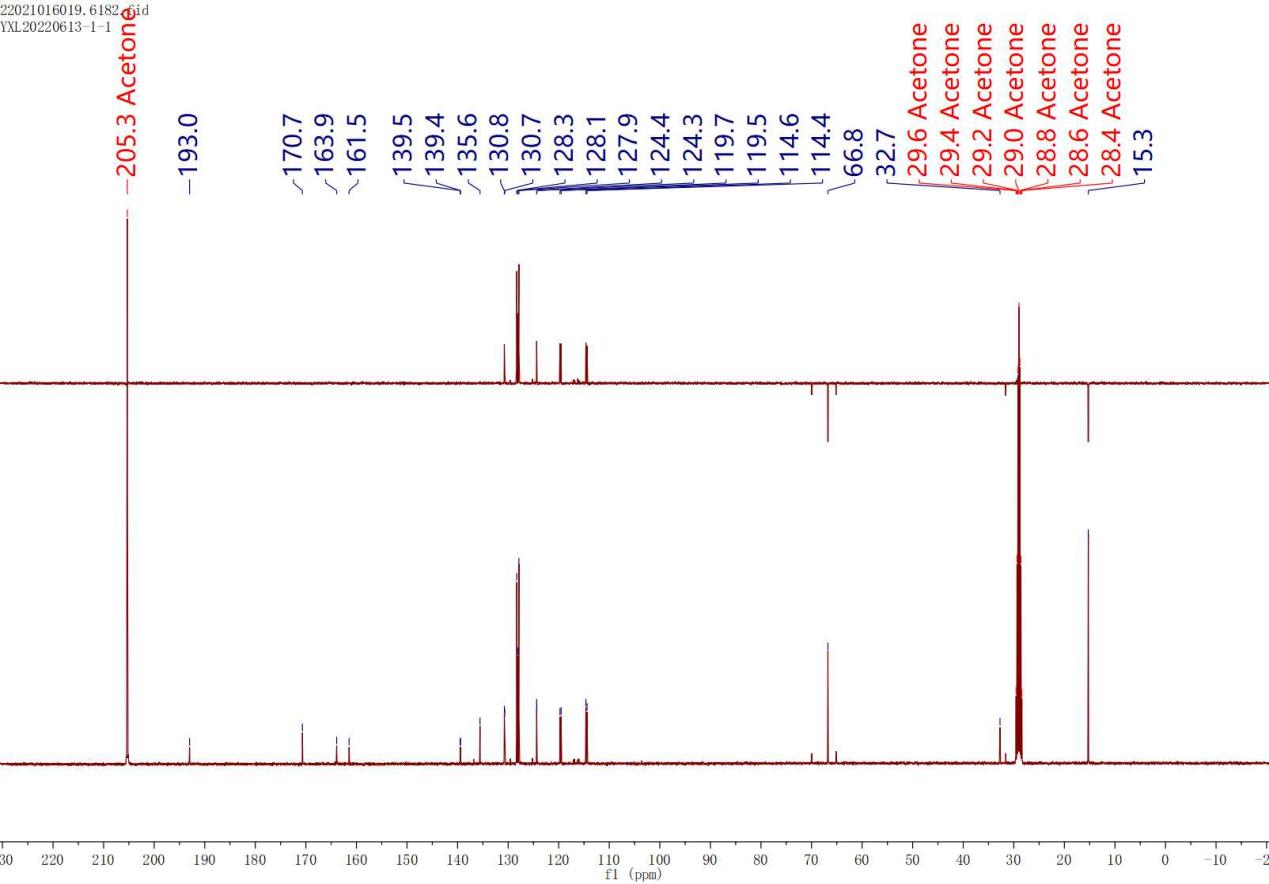


^13^C-NMR spectrum of compound (**4f**)


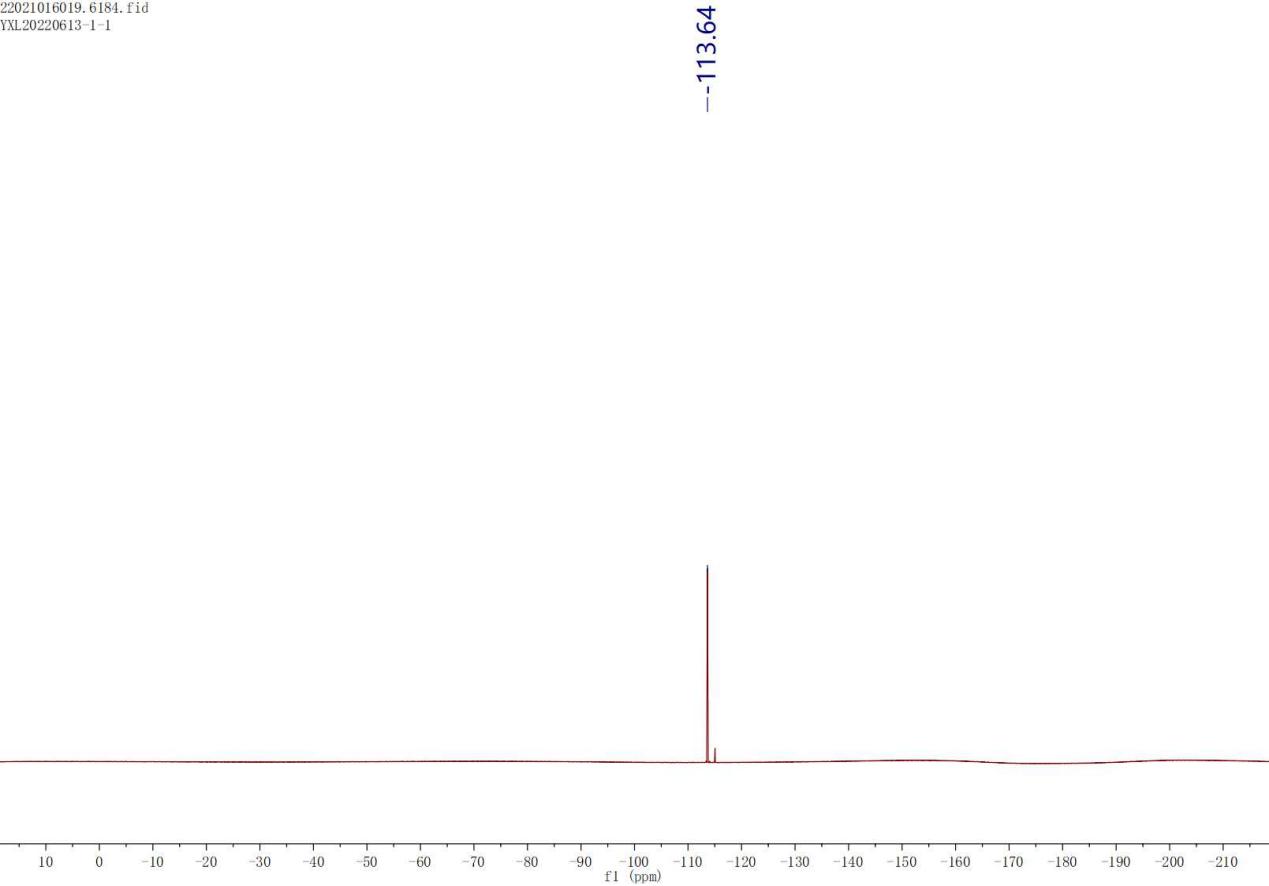


^19^F-NMR spectrum of compound (**4f**)


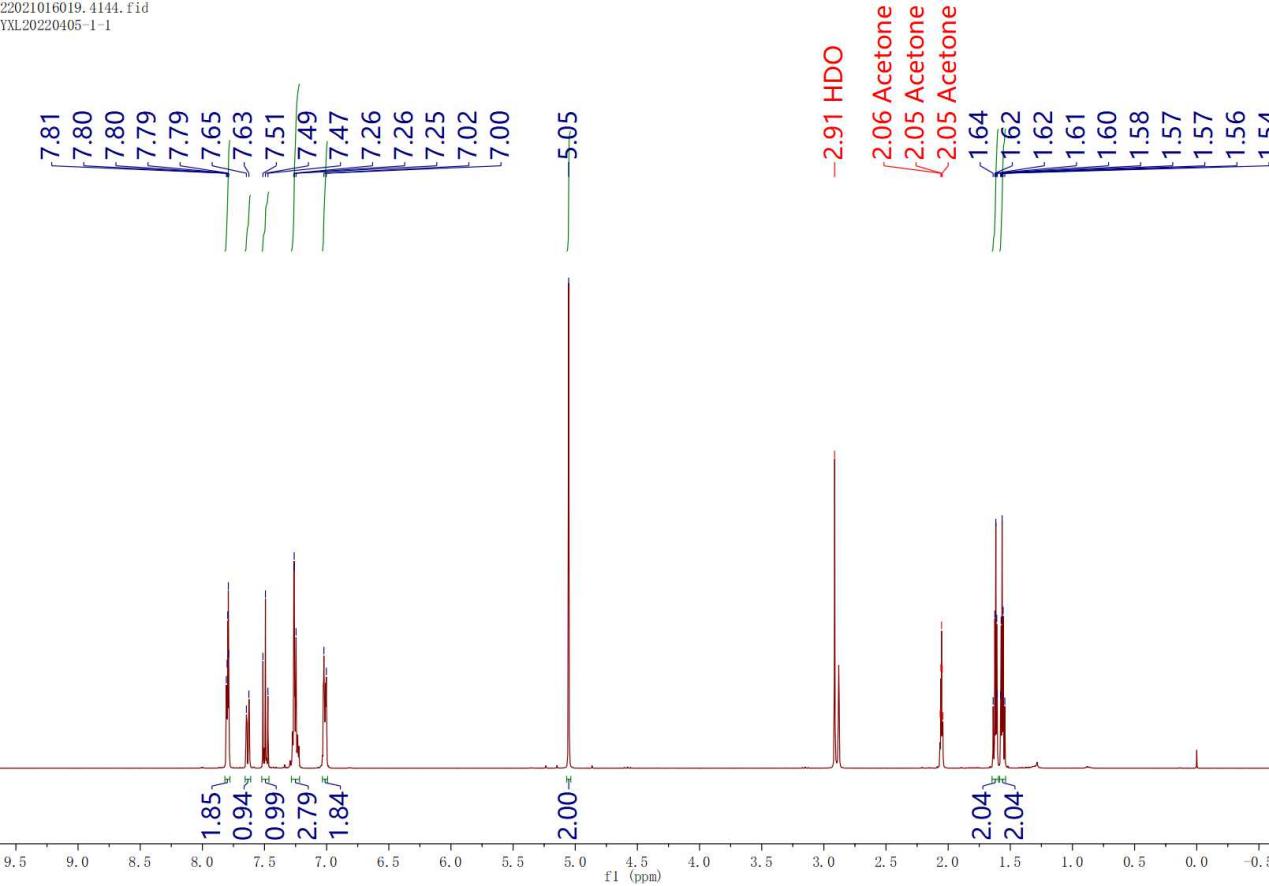


^1^H-NMR spectrum of compound (**4g**)


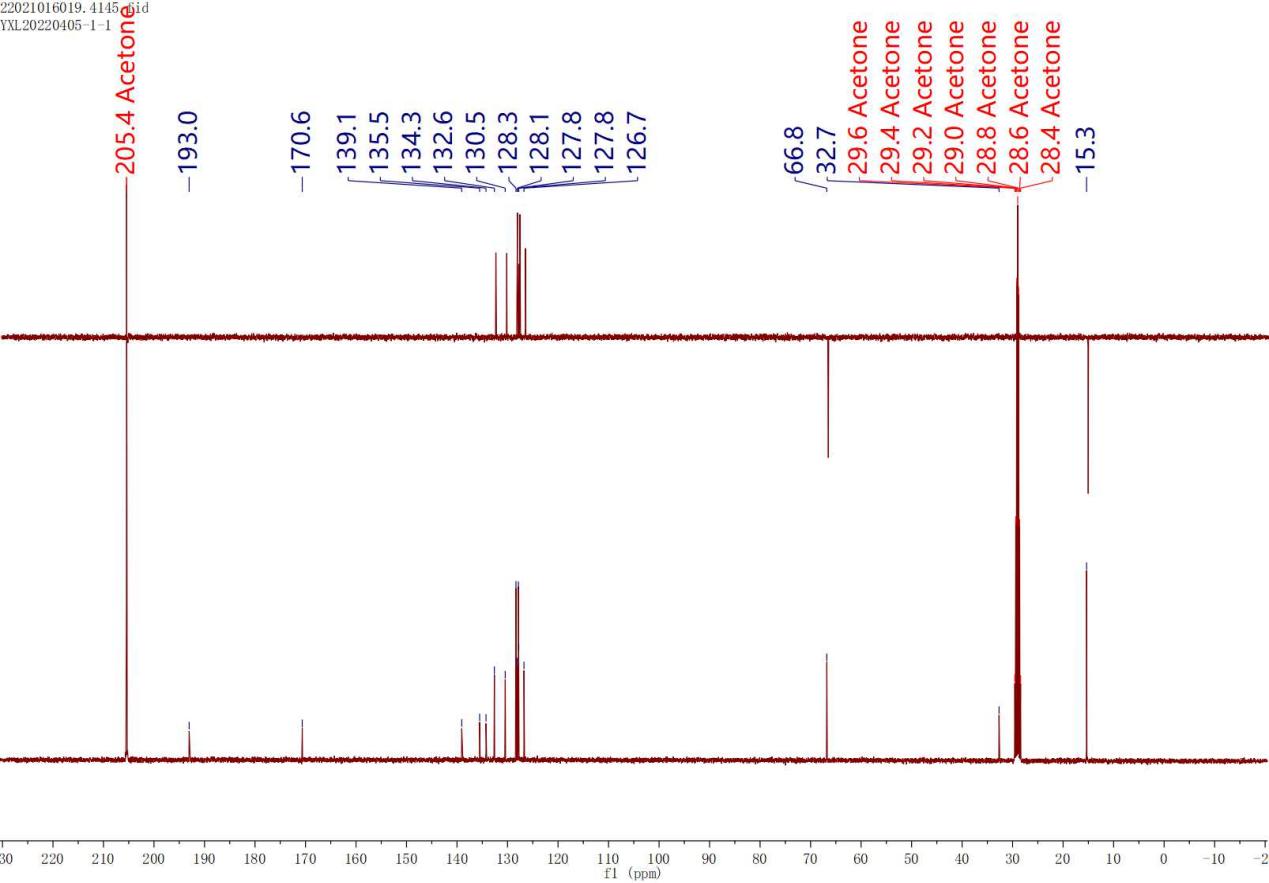


^13^C-NMR spectrum of compound (**4g**)


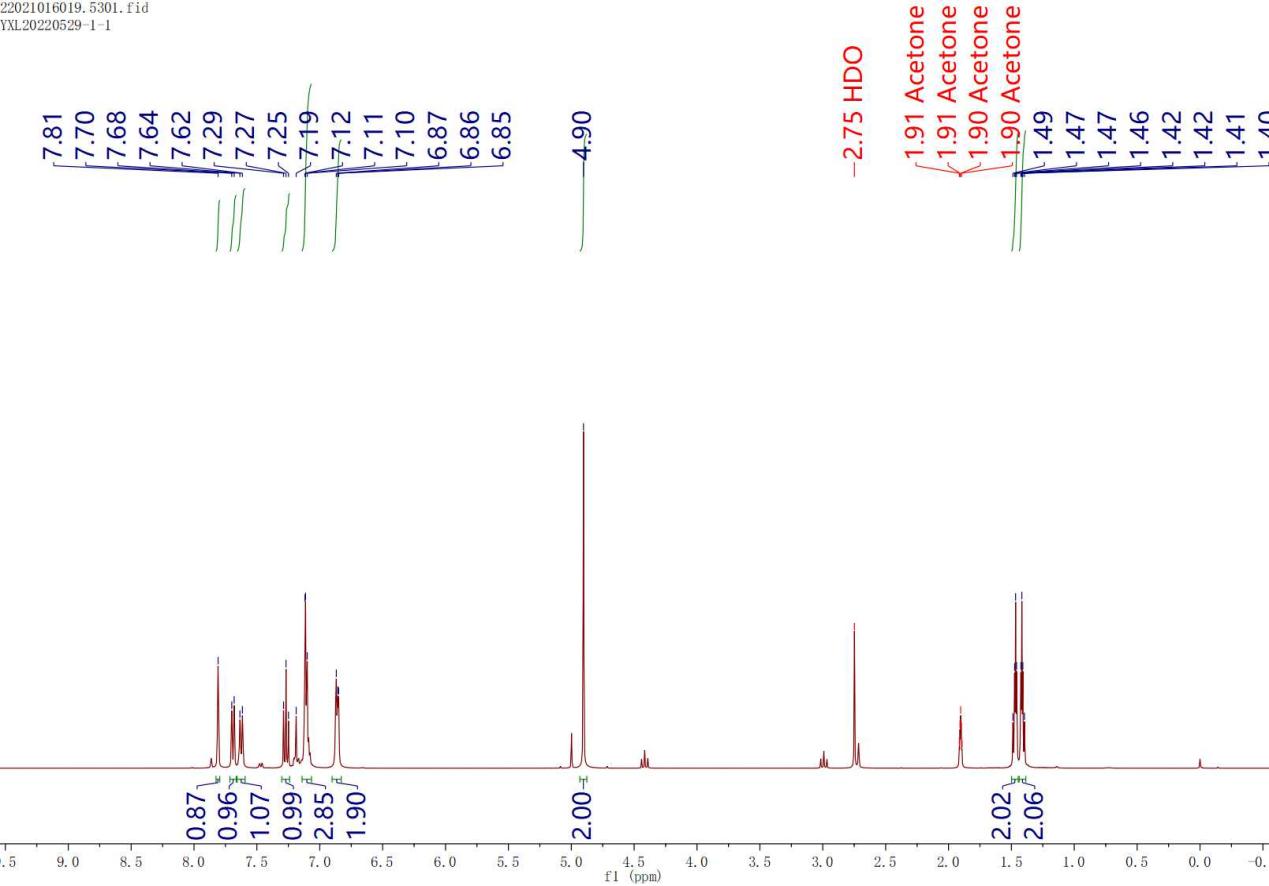


^1^H-NMR spectrum of compound (**4h**)


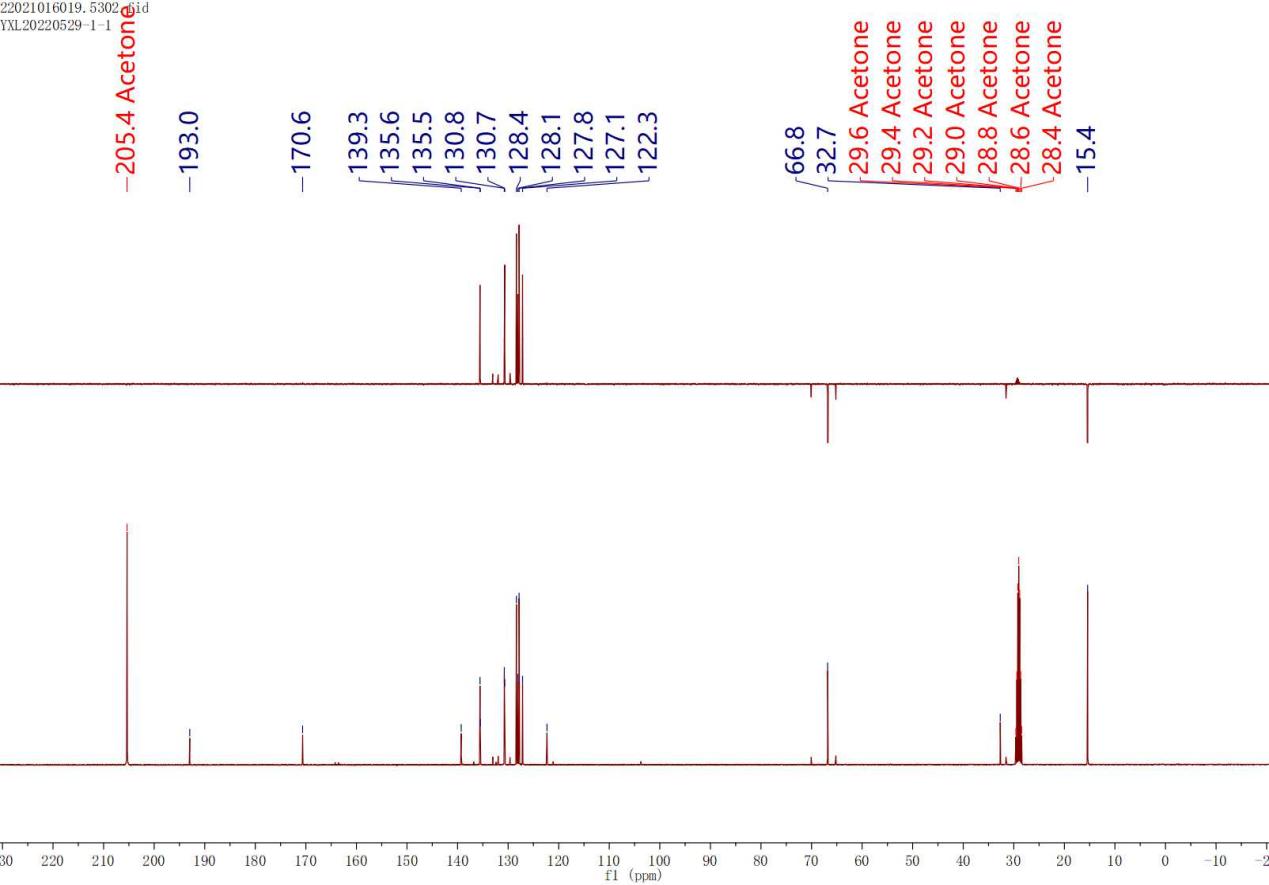


^13^C-NMR spectrum of compound (**4h**)


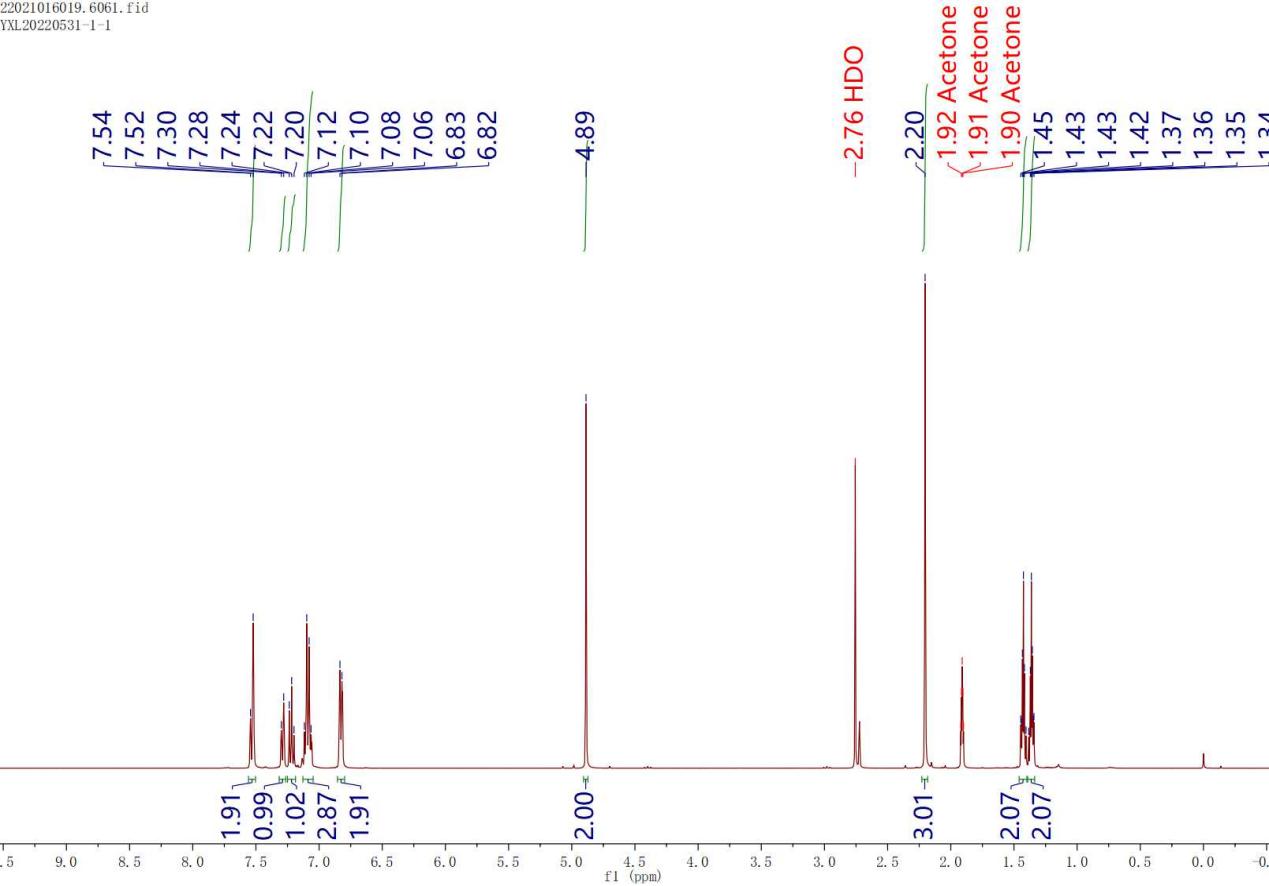


^1^H-NMR spectrum of compound (**4i**)


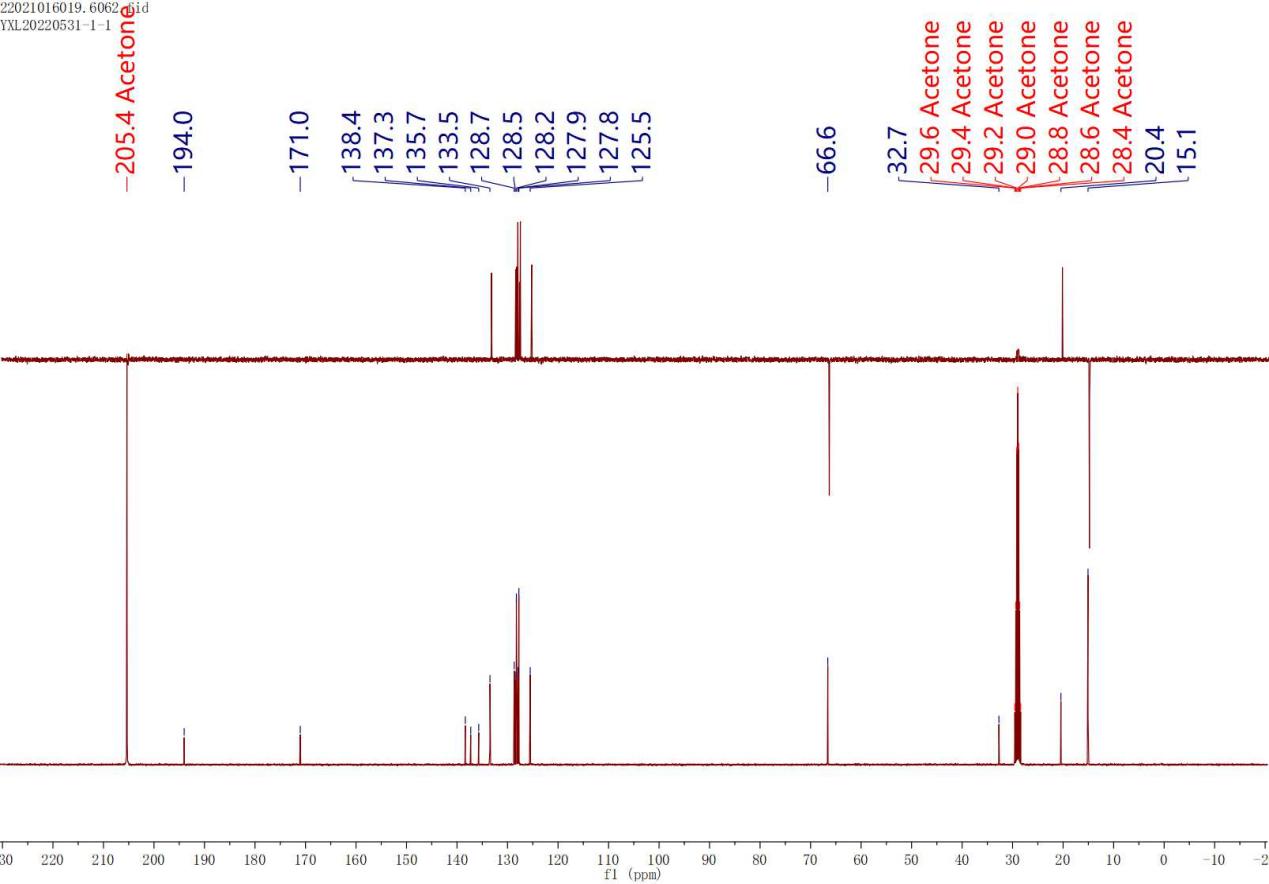


^13^C-NMR spectrum of compound (**4i**)


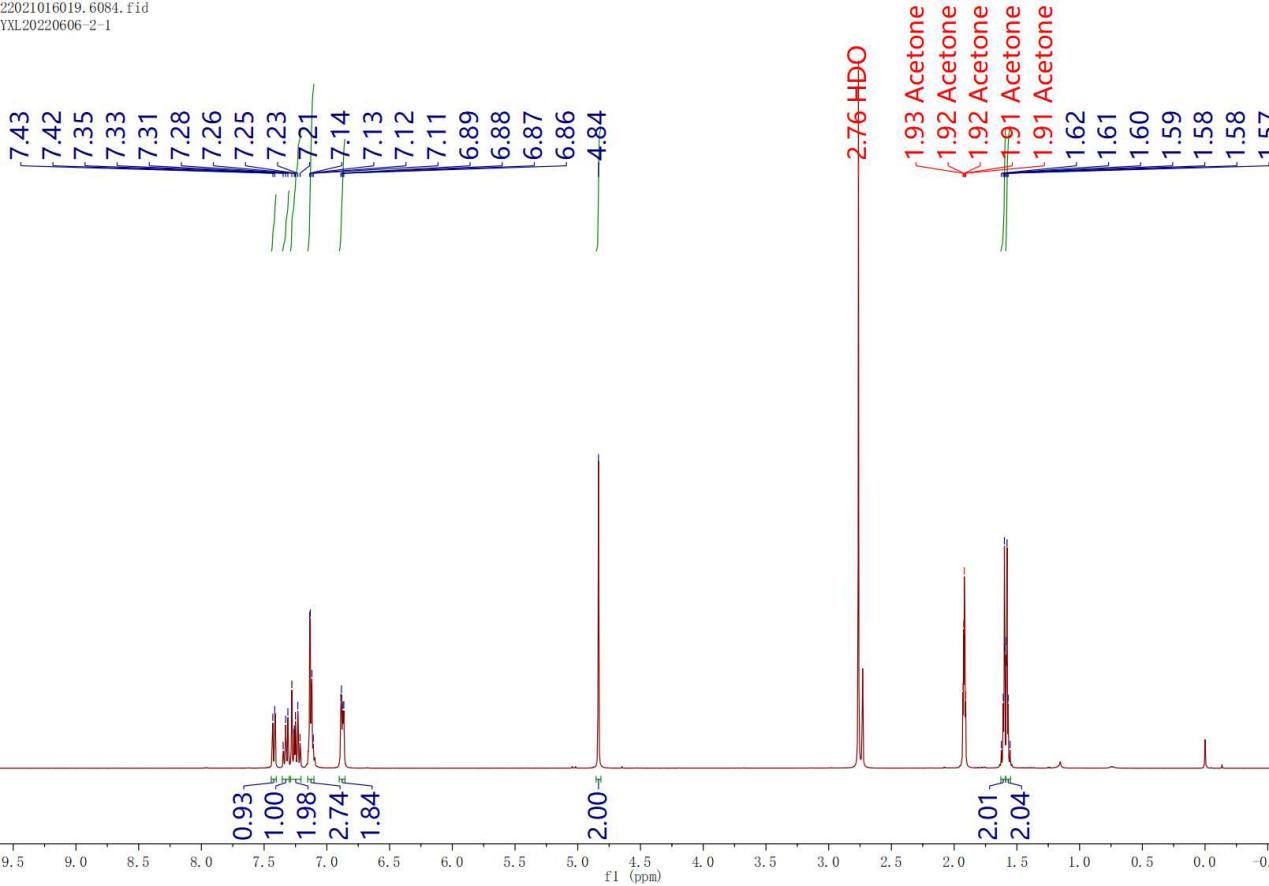


^1^H-NMR spectrum of compound (**4j**)


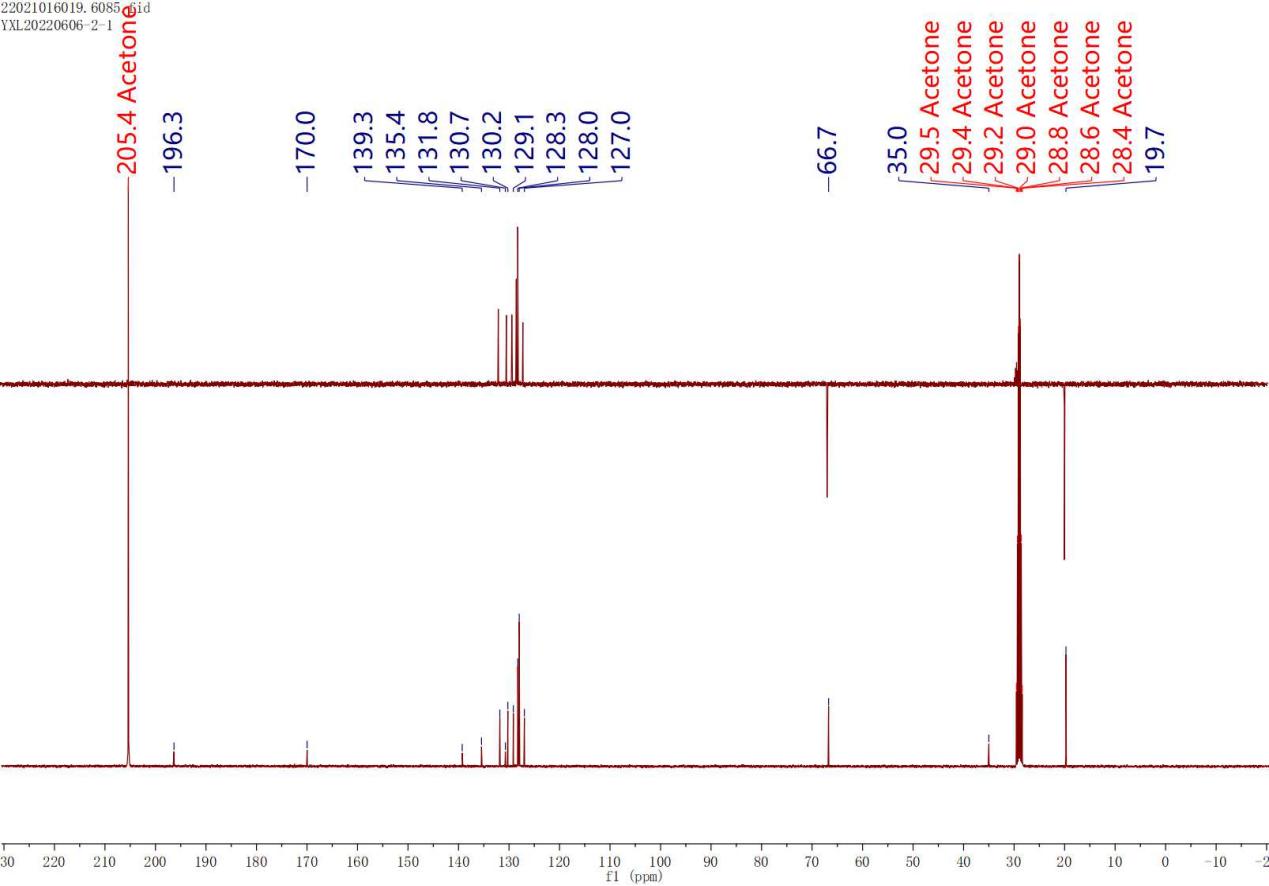


^13^C-NMR spectrum of compound (**4j**)


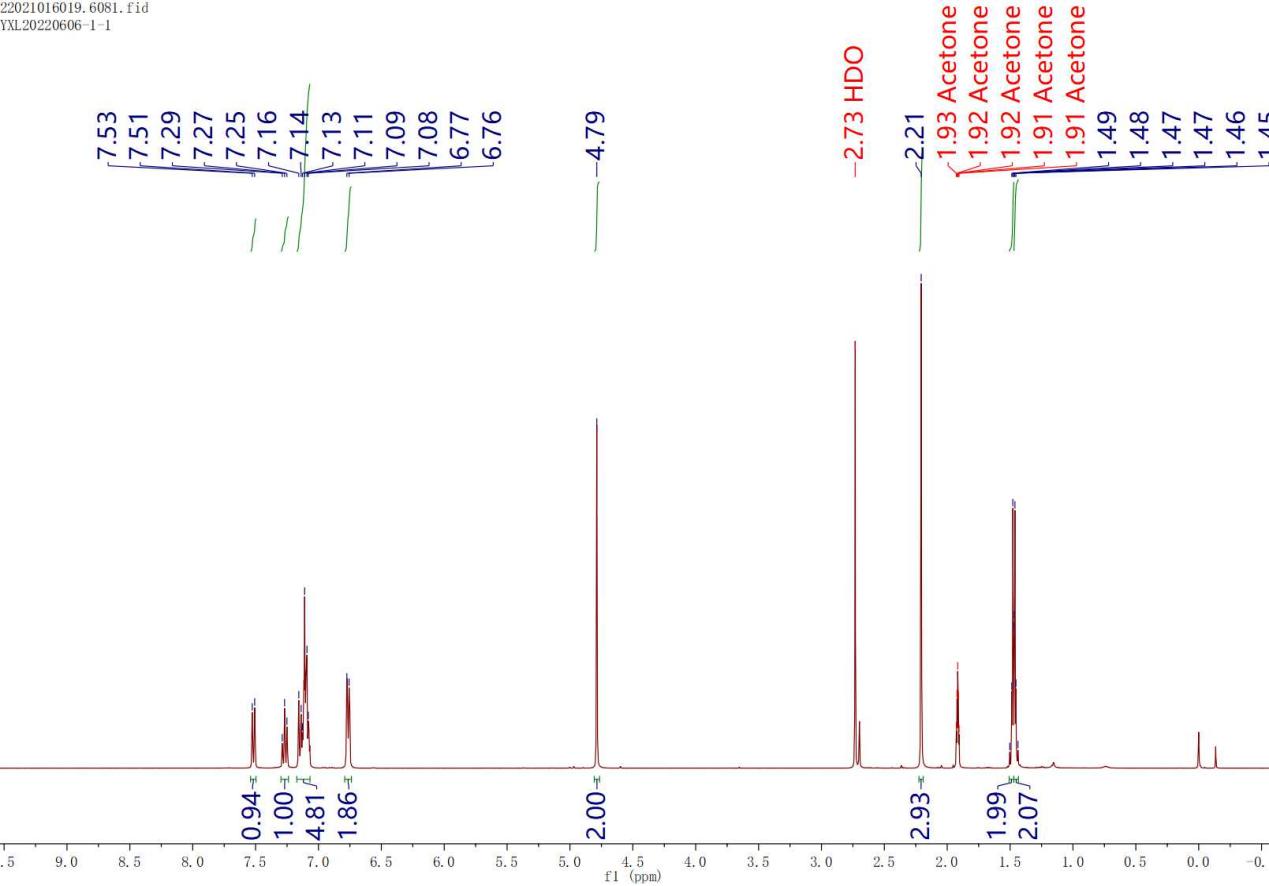


^1^H-NMR spectrum of compound (**4k**)


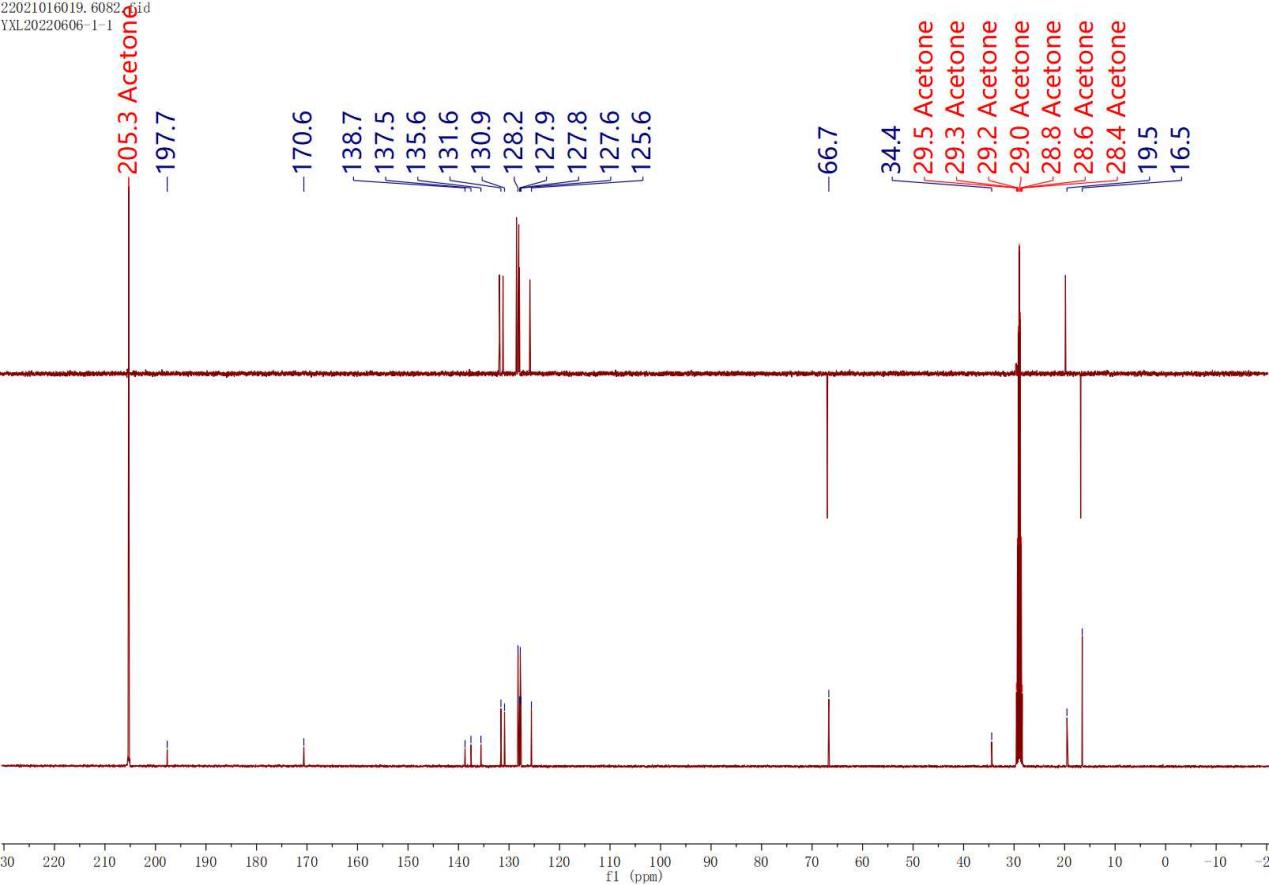


^13^C-NMR spectrum of compound (**4k**)


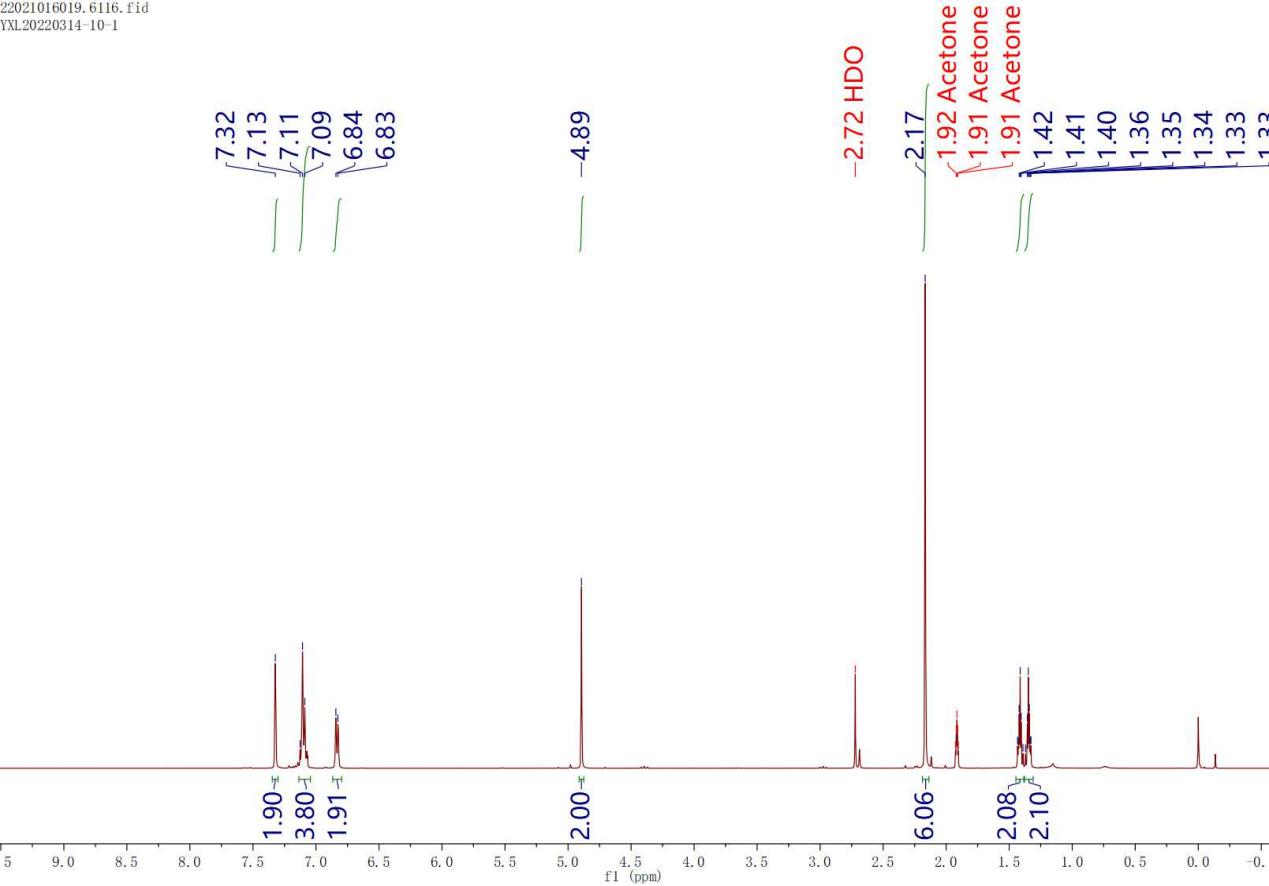


^1^H-NMR spectrum of compound (**4l**)


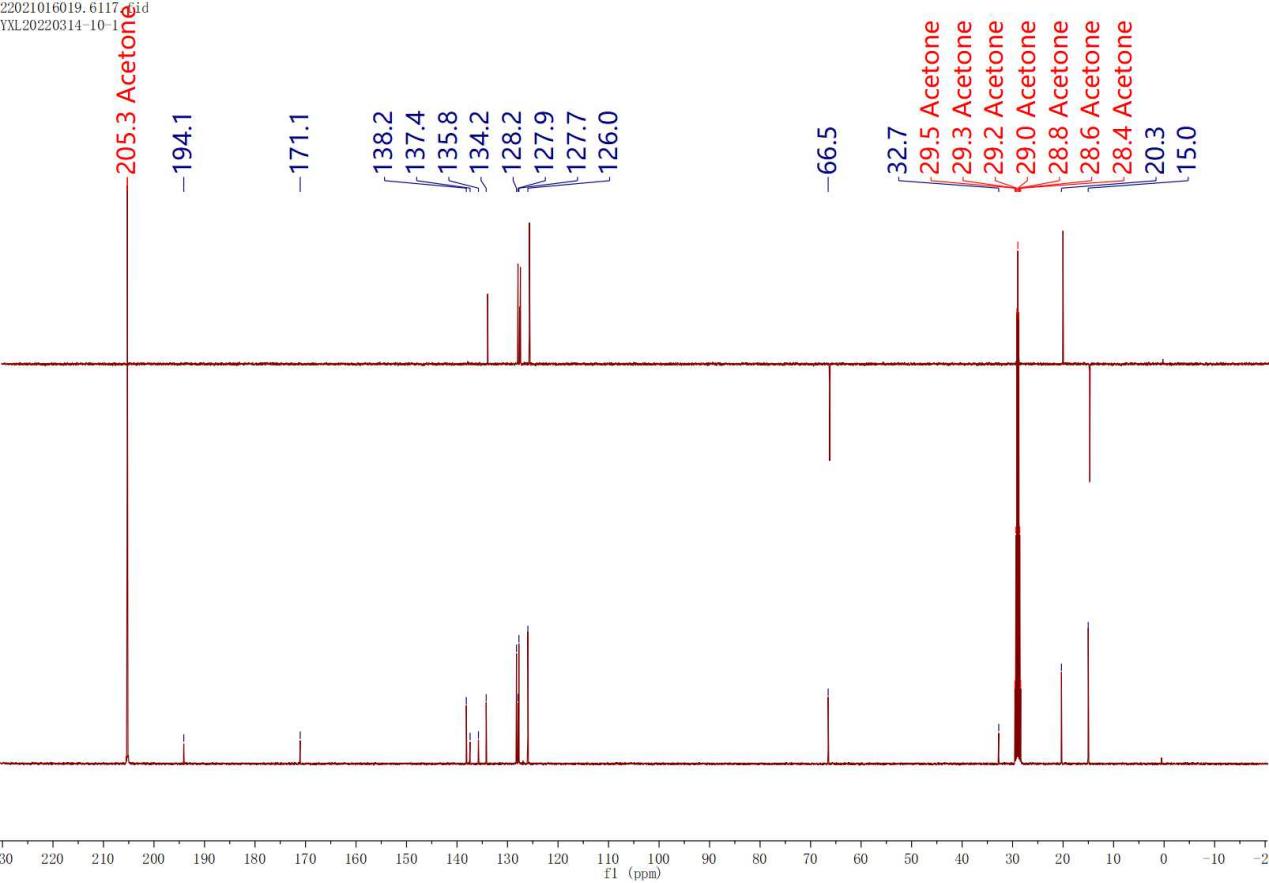


^13^C-NMR spectrum of compound (**4l**)


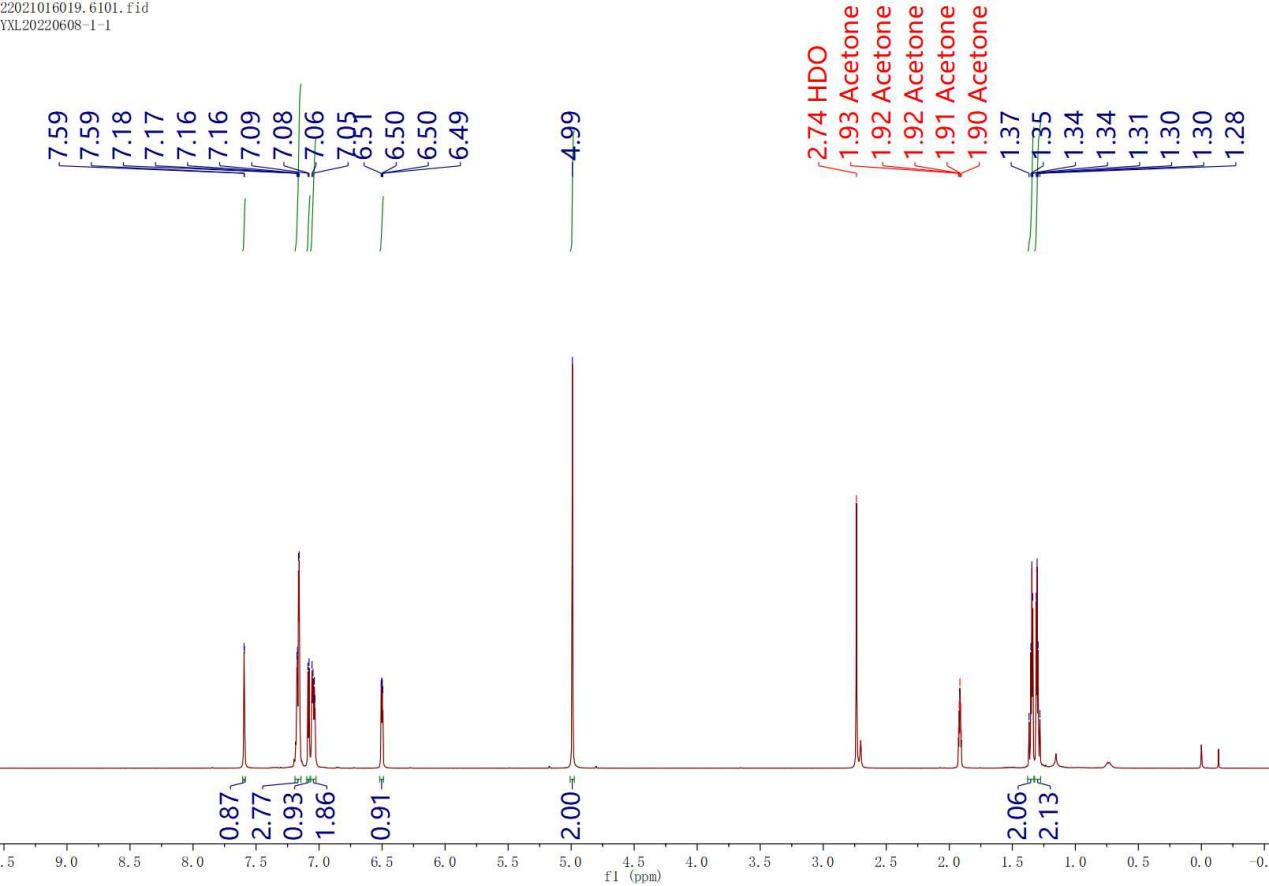


^1^H-NMR spectrum of compound (**4m**)


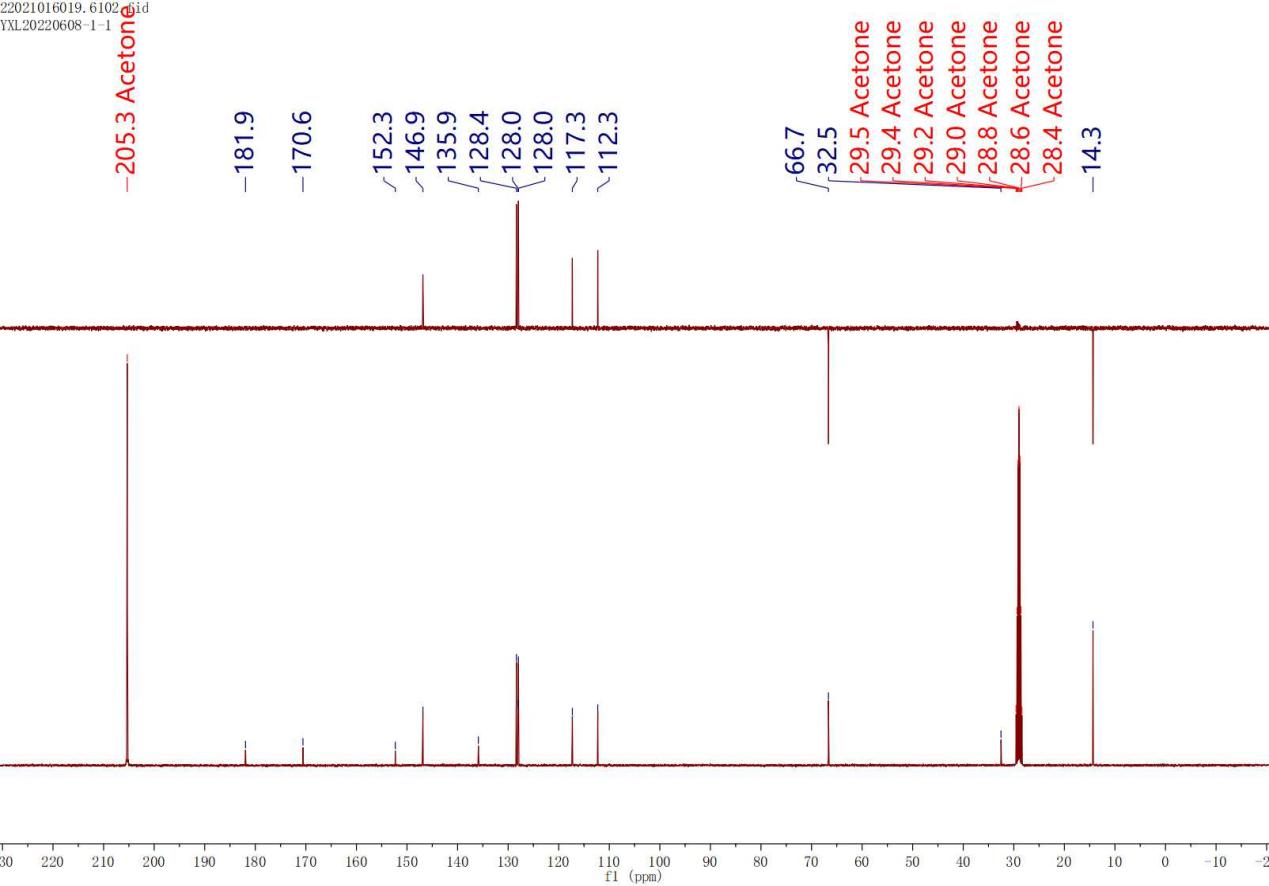


^13^C-NMR spectrum of compound (**4m**)


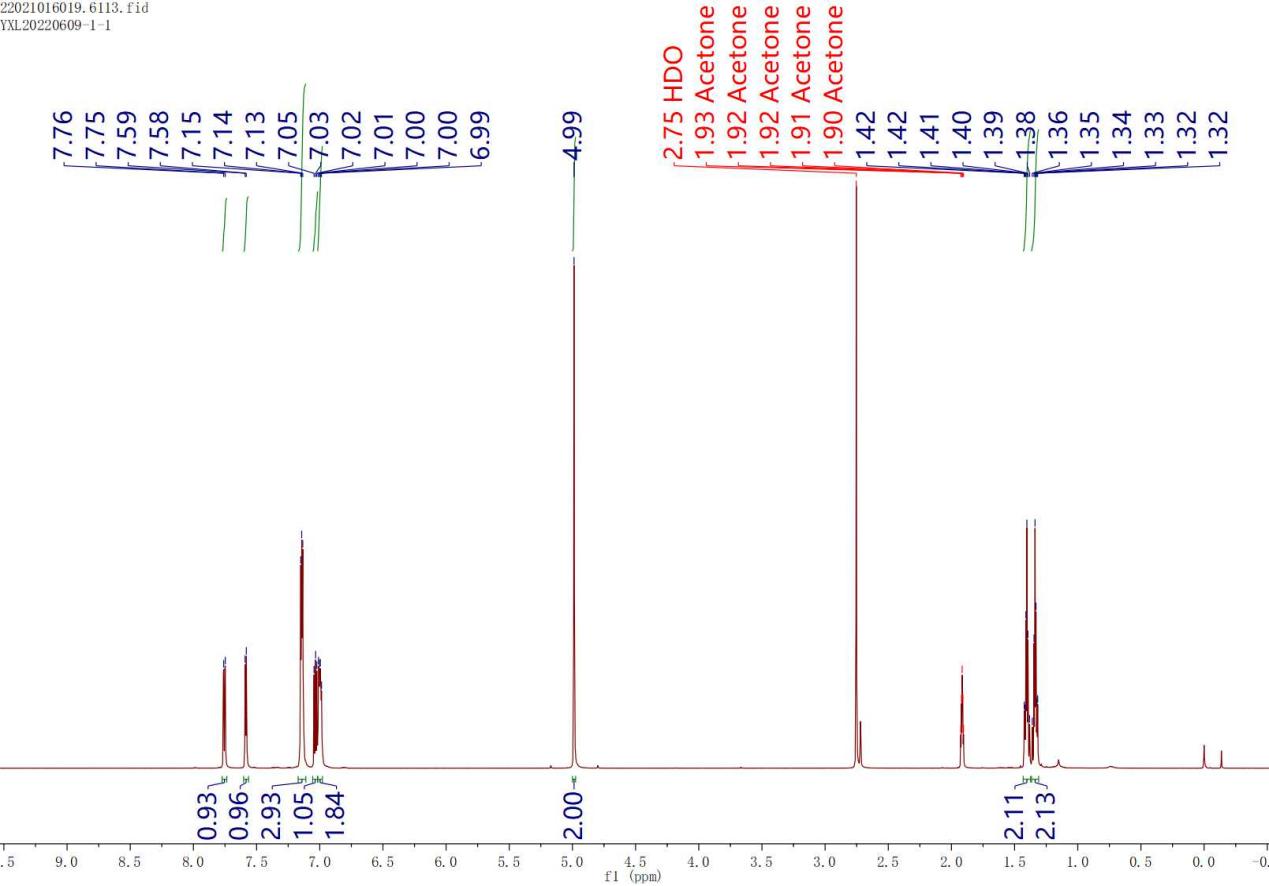


^1^H-NMR spectrum of compound (**4n**)


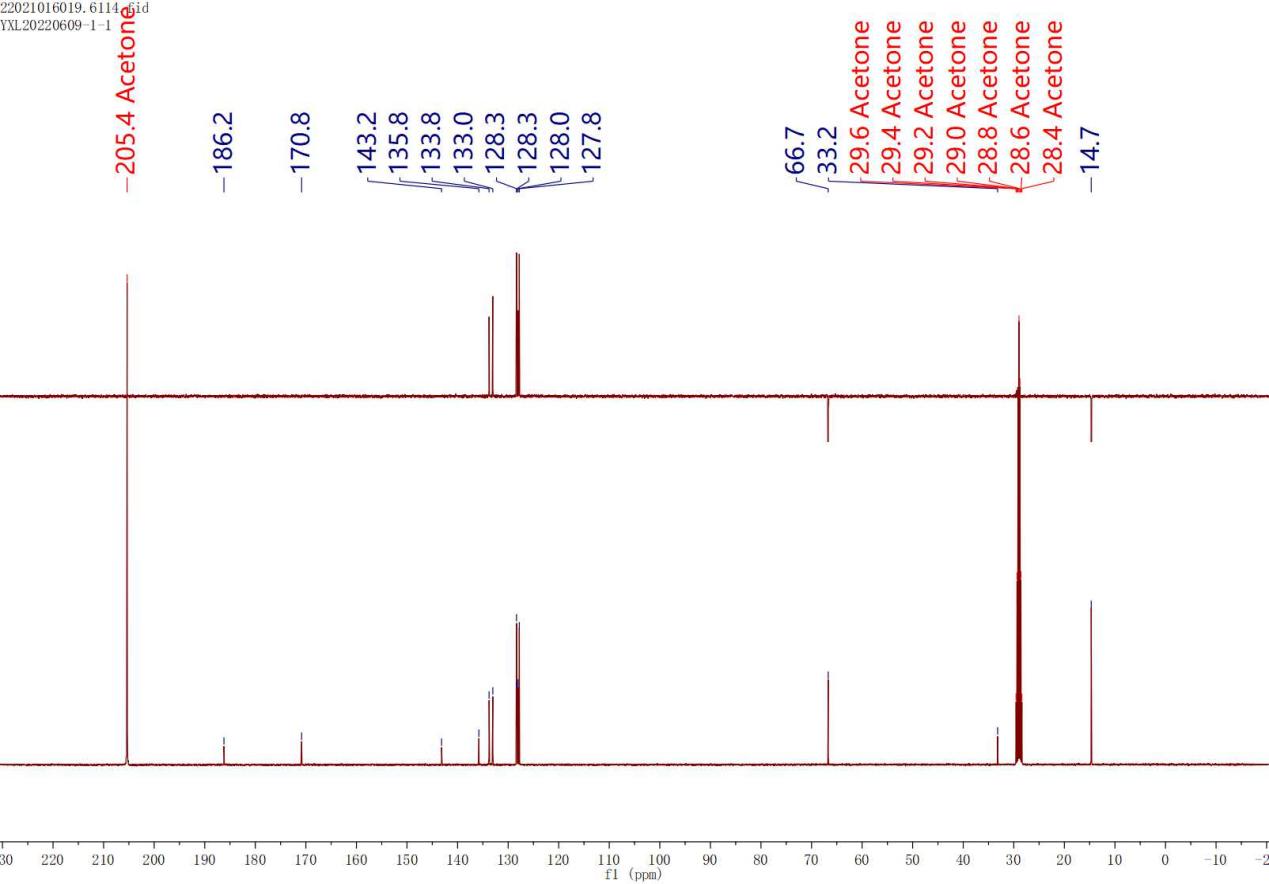


^13^C-NMR spectrum of compound (**4n**)


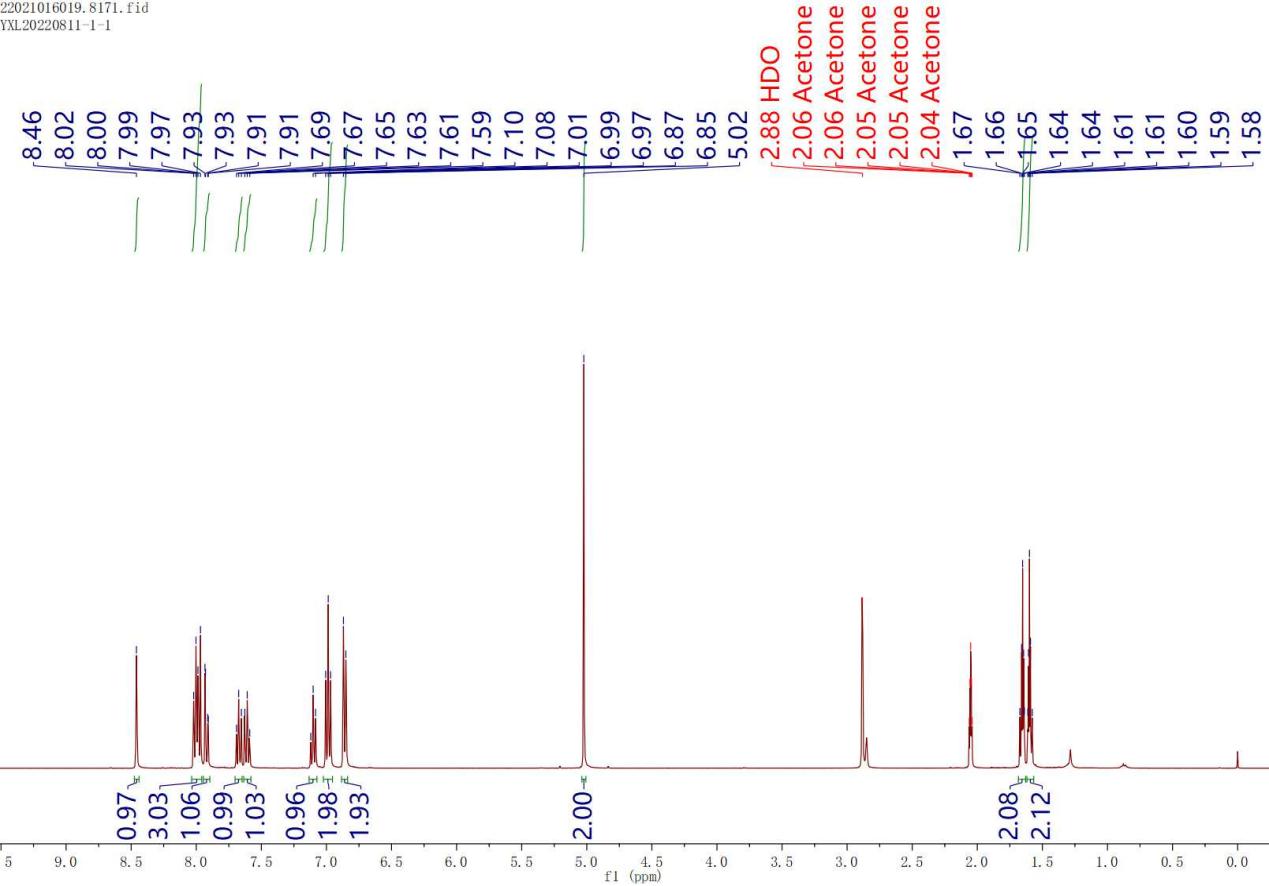


^1^H-NMR spectrum of compound (**4o**)


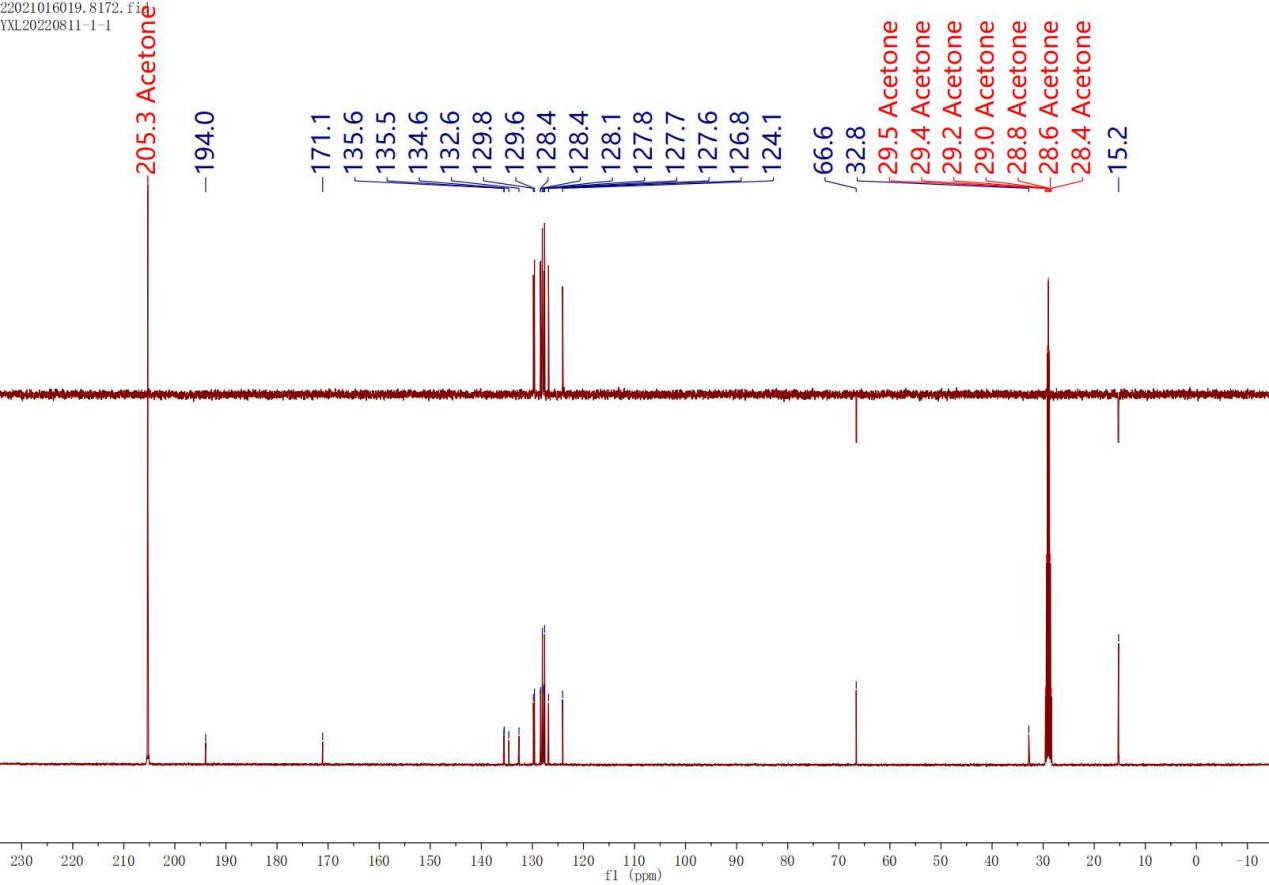


^13^C-NMR spectrum of compound (**4o**)


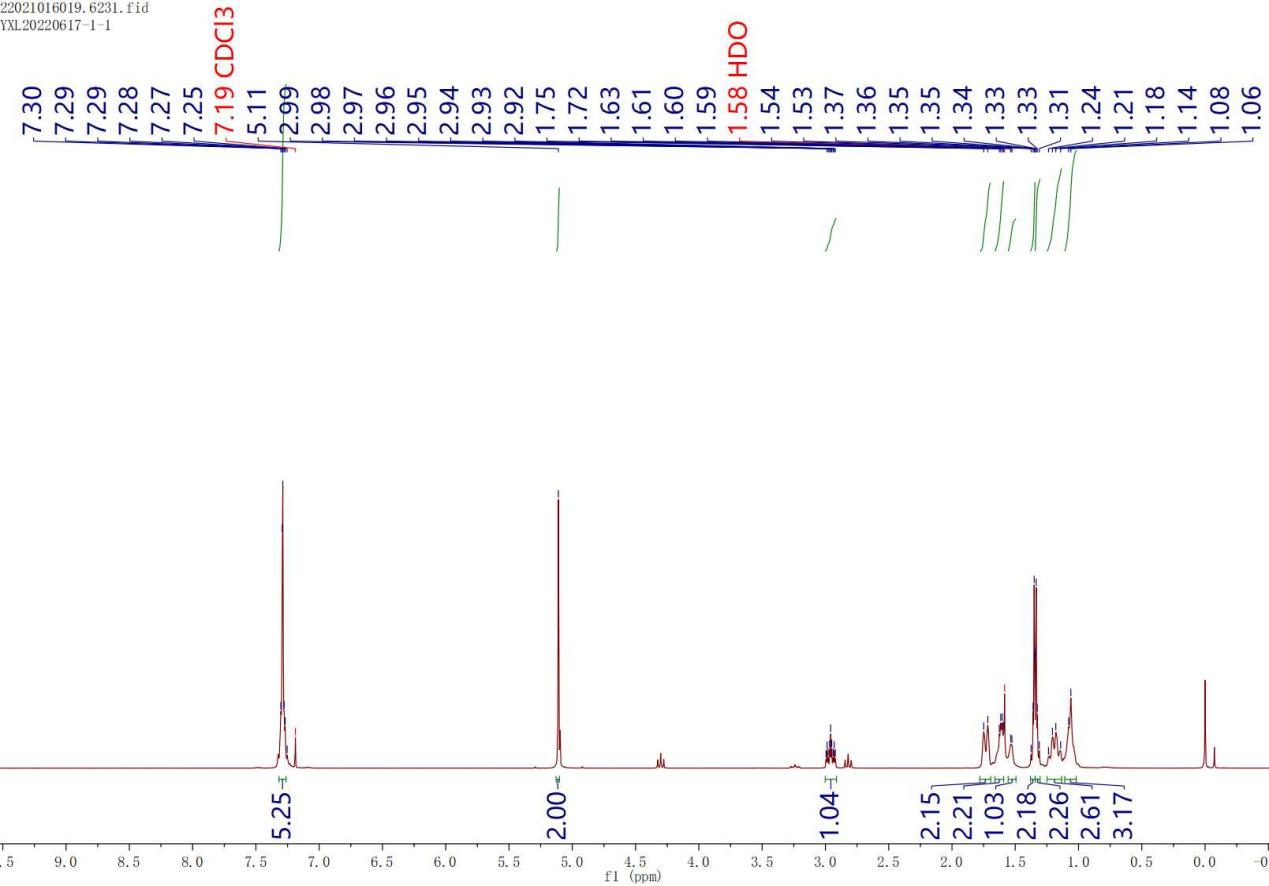


^1^H-NMR spectrum of compound (**4p**)


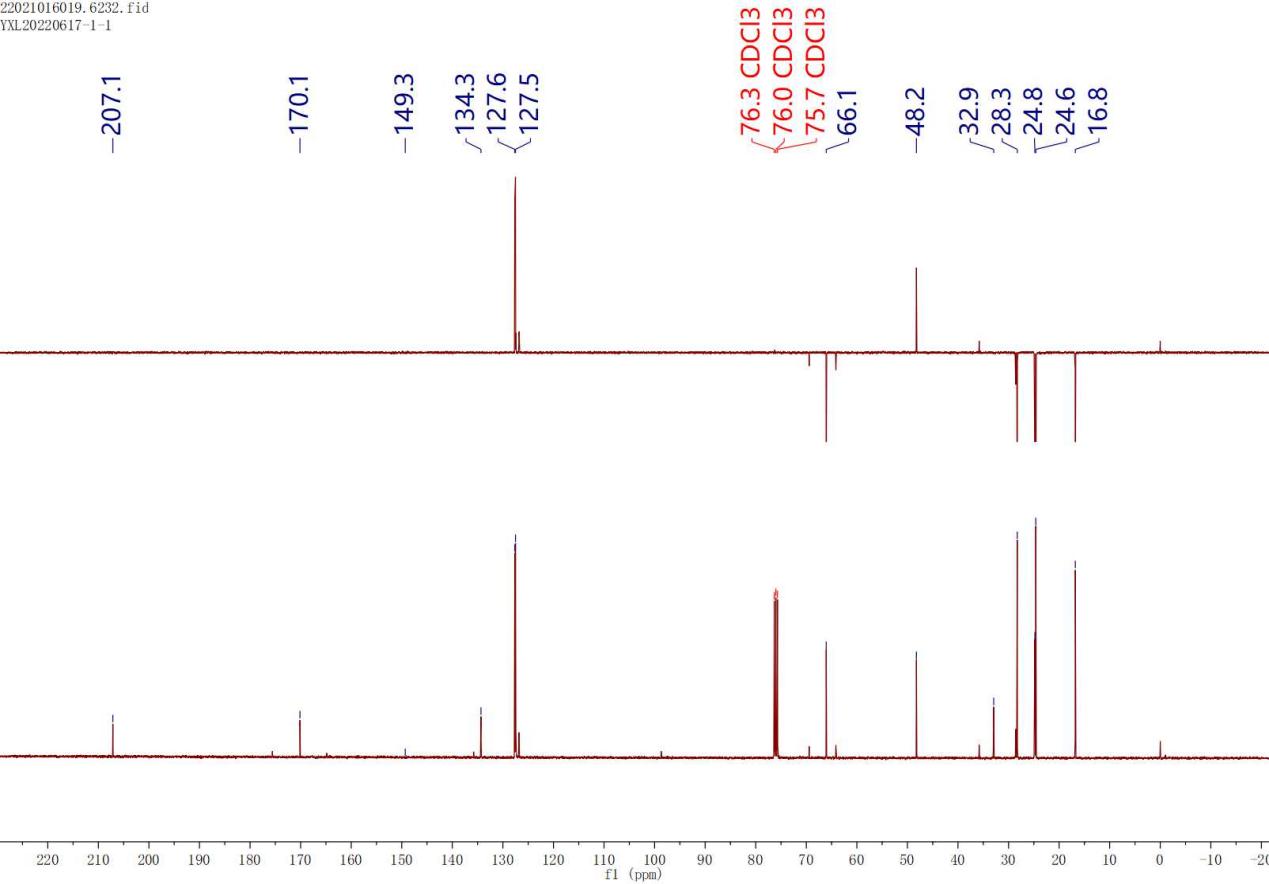


^13^C-NMR spectrum of compound (**4p**)


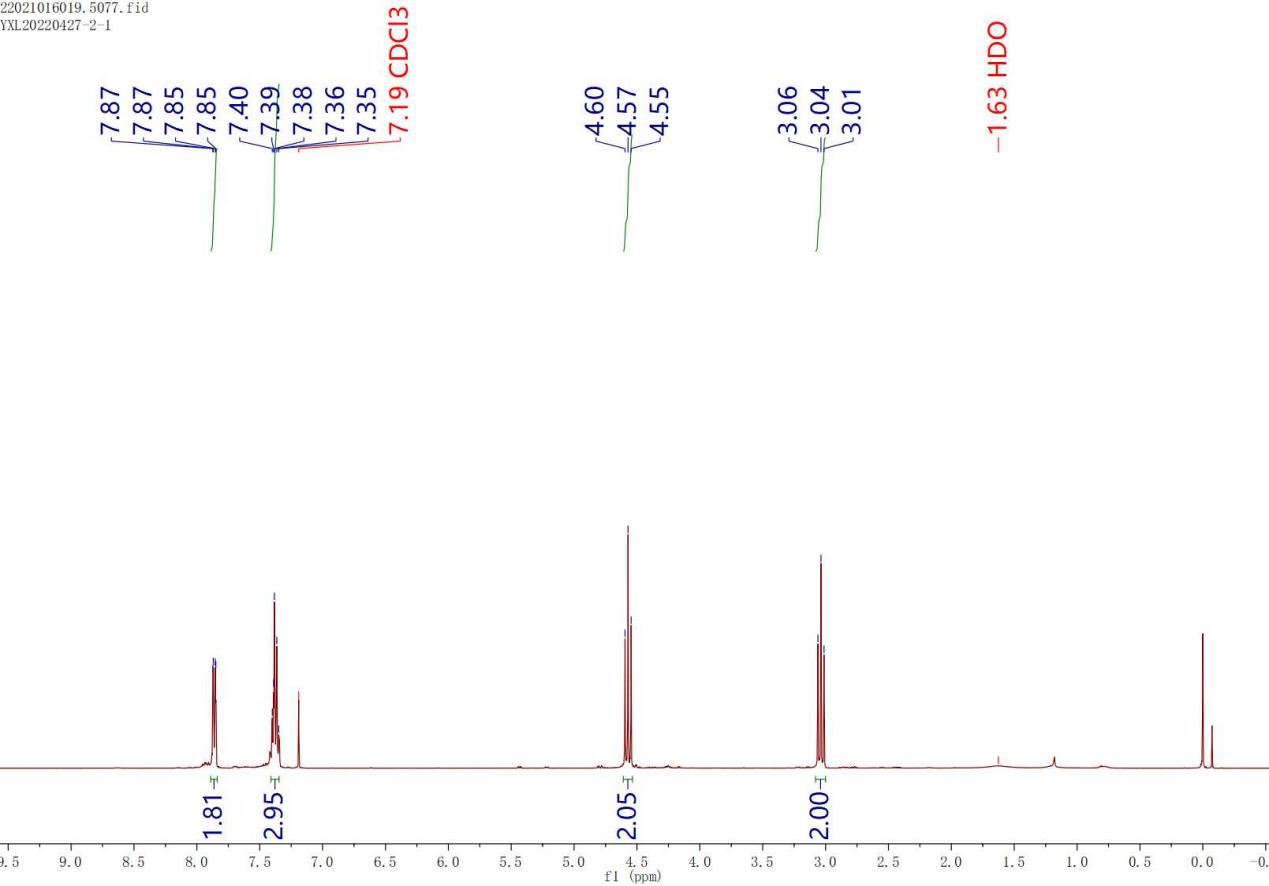


^1^H-NMR spectrum of compound (**5**)


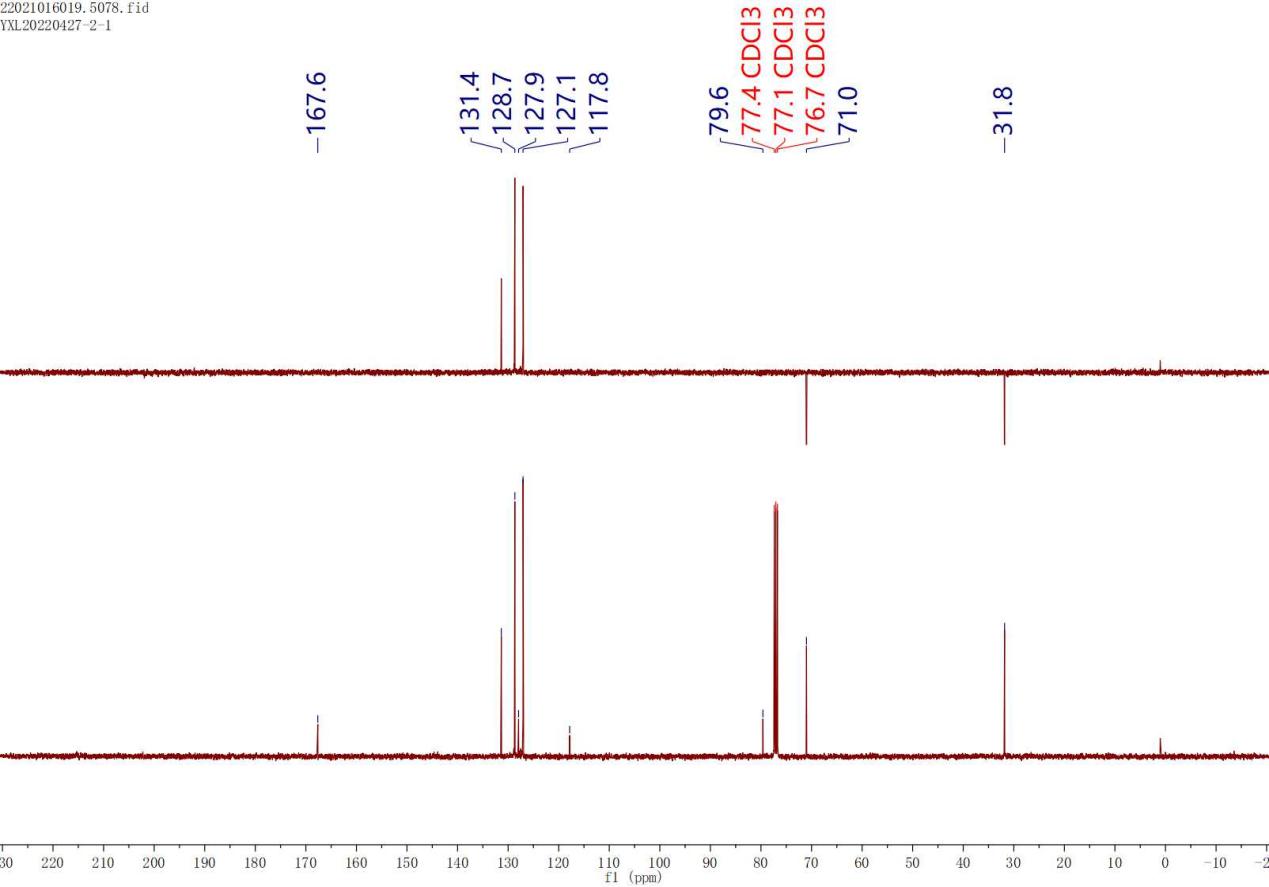


^13^C-NMR spectrum of compound (**5**)

^1^H-NMR spectrum of compound (**6**)

^13^C-NMR spectrum of compound (**6**)

^1^H-NMR spectrum of compound (**7**)

^13^C-NMR spectrum of compound (**7**)


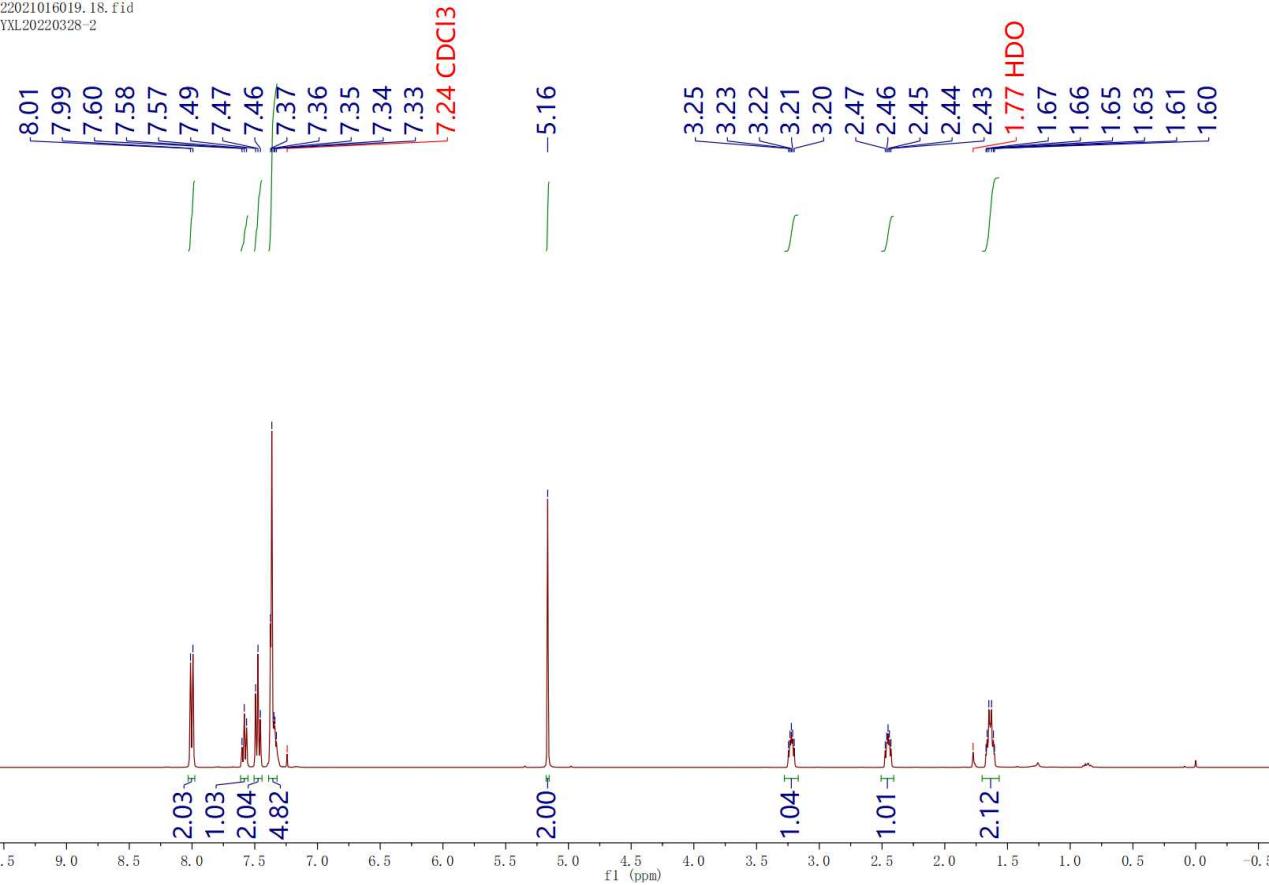


^1^H-NMR spectrum of compound (**3a’**)


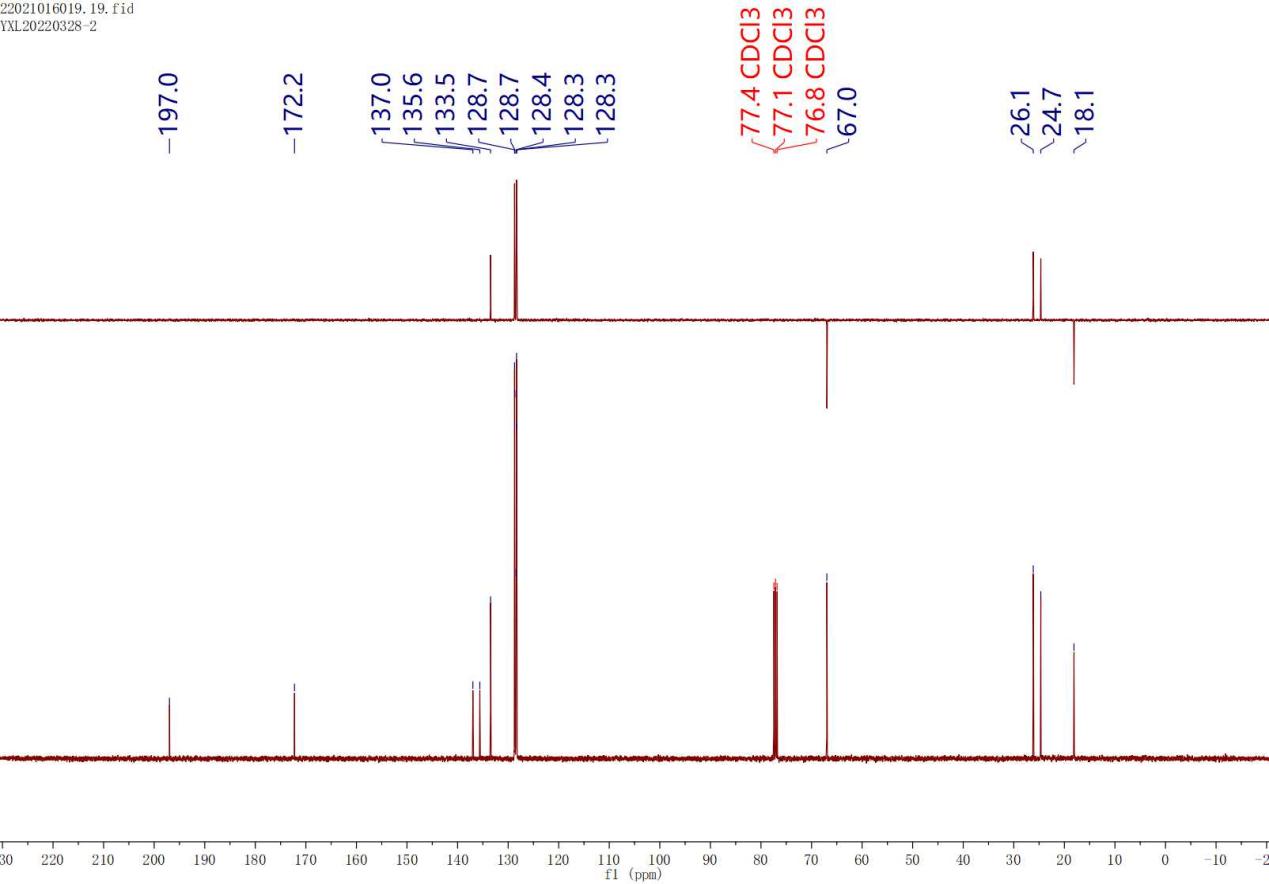


^13^C-NMR spectrum of compound (**3a’**)


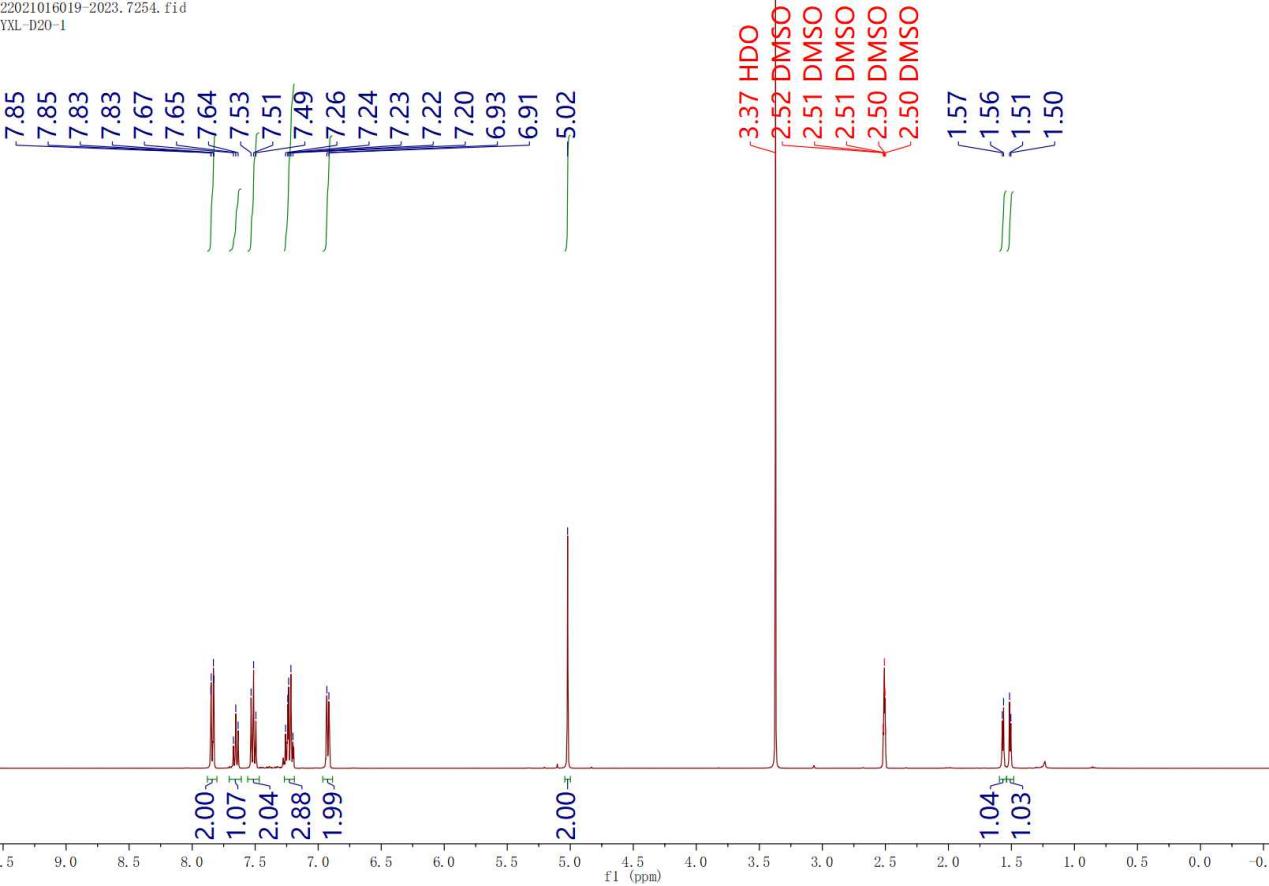


^1^H-NMR spectrum of compound (**3a-*d_2_***)


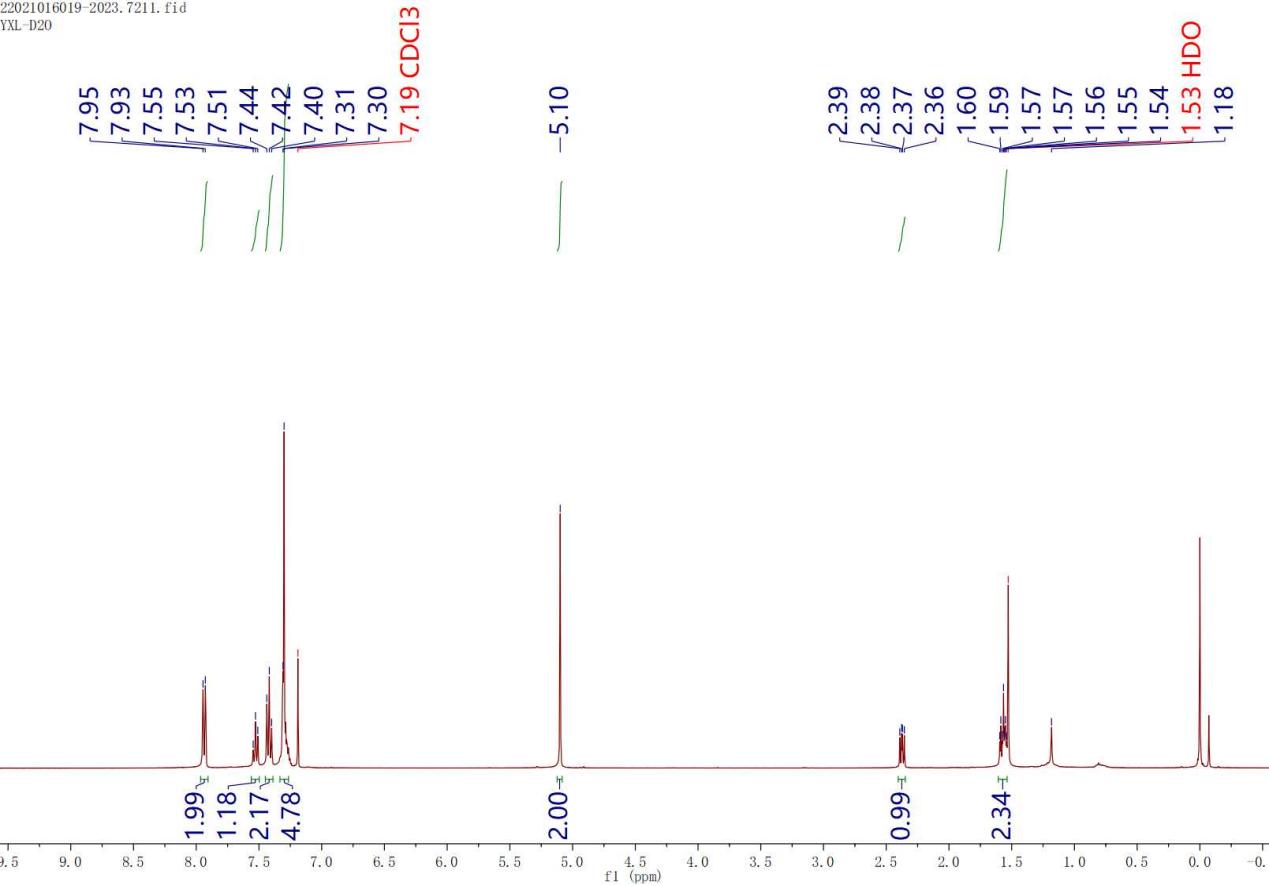


^1^H-NMR spectrum of compound (**3a’-*d***)

# DFT Studies

All DFT calculations were performed with the Gaussian 16 package^4^. The PBE0-D3^5^ functional and the standard 6-31G(d,p) basis set were used for geometry optimization. Harmonic vibrational requency calculations were performed for all stationary points to determine whether they are local minima or transition structures. The 6-311++G(d,p) basis set was used to calculate the single-point energies with the SMD implicit solvation model^6^. Images of the optimized structures were prepared using the CYLview program^7^.

The computational results reveals that water-assisted formation of the cyclobutane intermediate is energetically favored over cyclopropane product formation (Figure S3). In the first step, **1a** and **2o** undergo a Michael addition reaction to generate **int2** via the transition state **TS1**, which has an energy of 20.8 kcal/mol and is the rate-determining step of the whole reaction pathway. During this process, **1a** could attack both sides of **2o**, while the energy barrier via **TS1-S** is higher. Then, the generated **int2** undergoes an intramolecular nucleophilic addition with the carbonyl group of the benzoyl group through **TS2R** (energy barrier of 13.9 kcal/mol) to form the four-membered ring cyclic intermediate **int3**. Notably, a competing pathway proceeds by the traditional three-membered ring transition state **TS-by** to obtain the by-product, with an energy barrier of 16.8 kcal/mol. The energy difference between cyclobutene mechanism and cyclopropane mechanism could be attributed to the stronger hydrogen bonding in the cyclobutene transition state (in MS, Scheme 4). Following the preferred four-membered ring pathway, **int3** undergoes a ring opening to produce **int4**. Subsequently, the methyl hydrogen on the sulfoxide of **1a** undergoes proton transfer via H_2_O to form **int5**, which has an energy of 16.3 kcal/mol. **int5** undergoes a second proton transfer mediated by another water, which has an energy of 19.9 kcal/mol. Alternatively, the energy barrier for the one-step proton transfer via 1,3-proton transfer is 19.7 kcal/mol. This energy barrier is close to the two-step proton transfer, suggesting that the reaction could proceed through both processes. Finally, the ring closure yields the final cyclopropyl ketone **3o** and release DMSO via **TS6**, with an energy barrier of 14.3 kcal/mol. Notably, both the one-step and two-step proton transfer pathways exhibit relatively high energy barriers. This observation can be rationalized by two factors: (1) the proton transfer process proves inherently complex to characterize definitively, as the precise number of participating water molecules remains unresolved; (2) the reaction is likely governed by thermodynamic control under the high-temperature experimental conditions.

Meanwhile, the computational results reveal that the formation of cyclopropane products is more favorable than the formation of cyclobutane intermediate without water catalyst(Figure S4). In the first step, **1a** and **2o** undergo a Michael addition reaction to generate **int2-A** via the transition state **TS1-A**, which has an energy of 15.4 kcal/mol and is the rate-determining step of the whole reaction pathway. During this process, **1a** could attack both sides of **2o**, while **TS1S-A** has a higher activation barrier. Then, the generated **int2-A** undergoes an intramolecular nucleophilic addition with the carbonyl group of the benzoyl group through **TS2R-A** (energy barrier of 10.0 kcal/mol) to form the four-membered ring cyclic intermediate **int3-A**. Notably, the traditional three-membered ring transition state **TS-by-A** to obtain the by-product **TS-by-A**, with an energy barrier of 8.4 kcal/mol (Scheme 4). This suggests that the by-products are preferentially generated without water catalysis.The subsequent transition states were carried out under the condition of water catalysis. Finally, the ring closure yields the final cyclopropyl ketone **3o-A** and release DMSO via **TS6-A**, with an energy barrier of 14.8 kcal/mol.

**Figure S3**. Gibbs free energy profiles for water catalyzed cyclopropanation.

**Figure S4**. Gibbs free energy profiles for cyclopropanation without water.

**Energies (in Hartree)**

| **Complex** | **SCF-Done  Energy** | **Single Point Energy in Solvent** | **Free Energy with Corrections in Solvent** | **EZPE Energy** | **Imaginary  Frequency** |
| --- | --- | --- | --- | --- | --- |
| **1a** | -936.114873 | -936.2672434 | -936.1071594 | -935.915037 |  |
| **2o** | -422.510451 | -422.6030733 | -422.4930393 | -422.365895 |  |
| **H_2_O** | -76.344445 | -76.38599089 | -76.38199189 | -76.322796 |  |
| **DMSO** | -552.877434 | -552.9502447 | -552.8983487 | -552.797239 |  |
| **3o** | -882.160824 | -882.3702222 | -882.1283332 | -881.870255 |  |
| **by** | -882.156748 | -882.3683416 | -882.1256626 | -881.865653 |  |
| **3o-A** | -805.794013 | -805.9696331 | -805.7485411 | -805.528171 |  |
| **by-A** | -805.802959 | -805.979958 | -805.759789 | -805.536781 |  |
| **TS1R** | -1434.977513 | -1435.265313 | -1434.949072 | -1434.606035 | -421.014 |
| **TS1S** | -1434.98404 | -1435.263033 | -1434.945984 | -1434.611751 | -320.413 |
| **TS2R** | -1434.989777 | -1435.276758 | -1434.960067 | -1434.617638 | -137.526 |
| **TS2S** | -1434.971657 | -1435.265214 | -1434.949856 | -1434.600125 | -259.724 |
| **TS3** | -1434.997852 | -1435.274563 | -1434.954895 | -1434.624274 | -98.408 |
| **TS4** | -1434.982912 | -1435.271768 | -1434.956215 | -1434.611677 | -140.146 |
| **TS5** | -1434.983559 | -1435.265931 | -1434.950505 | -1434.614276 | -387.653 |
| **TS6** | -1435.005866 | -1435.287234 | -1434.972522 | -1434.634313 | -468.698 |
| **TS7** | -1434.98332 | -1435.270548 | -1434.950861 | -1434.610902 | -68.67 |
| **TS-by** | -1434.984625 | -1435.269885 | -1434.955371 | -1434.612964 | -354.701 |
| **int1** | -1435.002506 | -1435.283276 | -1434.970768 | -1434.631268 |  |
| **int2** | -1434.989501 | -1435.284245 | -1434.967914 | -1434.616537 |  |
| **int1'** | -1435.007164 | -1435.285176 | -1434.969883 | -1434.635497 |  |
| **int2'** | -1435.001275 | -1435.286062 | -1434.966717 | -1434.6269 |  |
| **int3** | -1435.00696 | -1435.285457 | -1434.965517 | -1434.633121 |  |
| **int3'** | -1434.97782 | -1435.271554 | -1434.954551 | -1434.605121 |  |
| **int4** | -1435.007105 | -1435.286303 | -1434.969402 | -1434.63289 |  |
| **int5** | -1435.00655 | -1435.285962 | -1434.968691 | -1434.632202 |  |
| **int6** | -1435.029504 | -1435.314224 | -1434.995245 | -1434.655043 |  |
| **TS1R-A** | -1358.612583 | -1358.870405 | -1358.575586 | -1358.266044 | -445.111 |
| **TS1S-A** | -1358.620433 | -1358.868505 | -1358.575267 | -1358.273799 | -379.068 |
| **TS2R-A** | -1358.626794 | -1358.880609 | -1358.584216 | -1358.2792 | -283.599 |
| **TS2S-A** | -1358.618959 | -1358.875059 | -1358.577543 | -1358.270952 | -256.714 |
| **TS3-A** | -1358.636482 | -1358.879291 | -1358.5807 | -1358.28784 | -241.725 |
| **TS6-A** | -1358.633506 | -1358.888589 | -1358.596616 | -1358.287169 | -445.393 |
| **TS-by-A** | -1358.628074 | -1358.88004 | -1358.586826 | -1358.281352 | -358.371 |
| **int1-A** | -1358.638195 | -1358.886635 | -1358.596038 | -1358.292386 |  |
| **int1'-A** | -1358.646349 | -1358.891124 | -1358.598998 | -1358.299974 |  |
| **int2'-A** | -1358.637093 | -1358.886832 | -1358.591007 | -1358.28867 |  |
| **int2-A** | -1358.637379 | -1358.885811 | -1358.590029 | -1358.288954 |  |
| **int3-A** | -1358.635158 | -1358.885101 | -1358.586768 | -1358.286359 |  |
| **int3'-A** | -1358.62539 | -1358.881881 | -1358.58386 | -1358.27665 |  |
| **int4-A** | -1358.641106 | -1358.885122 | -1358.589834 | -1358.292187 |  |
| **int6-A** | -1358.65466 | -1358.916236 | -1358.620221 | -1358.305565 |  |

**Cartesian Coordinates**

1a

C -0.795752 0.652894 -0.128624

S -2.447546 0.330174 -0.079861

O -3.272852 1.527612 -0.341102

C -2.847023 -0.974544 -1.226769

C -2.855136 -0.399799 1.492517

C 0.108036 -0.402708 0.097872

O -0.255703 -1.580790 0.334027

C 1.575357 -0.095694 0.039557

C 2.458803 -1.170673 -0.092557

C 3.830806 -0.956064 -0.155511

C 4.337370 0.340077 -0.075774

C 3.464839 1.417132 0.069351

C 2.091559 1.201301 0.125258

H -0.573324 1.664513 -0.435836

H -3.885061 -1.276277 -1.079175

H -2.148552 -1.789301 -1.024838

H -2.694306 -0.561715 -2.224750

H -2.160107 -1.228202 1.642378

H -2.707319 0.380572 2.239903

H -3.893497 -0.733840 1.470364

H 2.041975 -2.171321 -0.143867

H 4.507065 -1.798811 -0.265712

H 5.408982 0.510640 -0.121249

H 3.855402 2.427626 0.144696

H 1.424760 2.047225 0.260541

2o

C 3.557890 -0.558927 0.027385

C 2.244705 -0.747913 0.171252

C 1.295971 0.379467 -0.021328

C -0.168725 0.103305 -0.002968

O 1.705571 1.524334 -0.181489

C -1.037287 1.198618 0.089642

C -2.411674 1.010195 0.102411

C -2.937432 -0.278990 0.009295

C -2.083550 -1.373949 -0.096184

C -0.704858 -1.186584 -0.097394

H 4.270456 -1.364006 0.172926

H 3.941092 0.421090 -0.242964

H 1.858265 -1.723349 0.446470

H -0.606458 2.192433 0.154327

H -3.076578 1.864600 0.183756

H -4.012918 -0.428875 0.017107

H -2.491189 -2.376669 -0.177920

H -0.057482 -2.051226 -0.194456

3o-A

C 0.323914 1.879820 -1.434176

C -0.043544 1.419158 -0.034608

C 0.538136 2.795261 -0.284129

C 0.715944 0.407298 0.761921

O 0.170745 -0.038676 1.764521

C -1.532148 1.379616 0.243264

O -2.131451 2.375357 0.611435

C 2.055304 -0.122288 0.365144

C 2.330697 -1.437776 0.764162

C 3.543385 -2.035623 0.450532

C 4.512416 -1.317525 -0.248181

C 4.258663 -0.003558 -0.631655

C 3.035566 0.590681 -0.336870

C -2.253339 0.106718 -0.030067

C -1.668601 -0.940898 -0.749708

C -2.392355 -2.099068 -1.008978

C -3.701085 -2.221592 -0.546022

C -4.290549 -1.179710 0.169831

C -3.571616 -0.018634 0.420465

H 1.169508 1.399974 -1.914619

H -0.513362 2.085798 -2.094590

H 1.543489 2.990310 0.071158

H -0.165921 3.607271 -0.134498

H 1.570672 -1.979378 1.317351

H 3.735442 -3.060420 0.752887

H 5.464424 -1.780052 -0.490253

H 5.015116 0.565336 -1.162904

H 2.877385 1.619644 -0.635214

H -0.653249 -0.849513 -1.123215

H -1.936044 -2.906151 -1.573494

H -4.263314 -3.129029 -0.745203

H -5.310382 -1.275350 0.529626

H -4.012847 0.806834 0.969758

3o

C -1.526364 -2.336835 -1.586374

C -1.156315 -1.137933 -0.728548

C -2.564245 -1.703138 -0.739956

C -0.985009 0.169415 -1.453110

O -1.122151 0.241441 -2.664764

C -0.276453 -1.449137 0.442472

O -0.720690 -1.959477 1.467126

C -0.673653 1.373295 -0.640641

C -1.082479 1.486722 0.693167

C -0.771845 2.636665 1.412310

C -0.043602 3.662699 0.814331

C 0.358487 3.553137 -0.517239

C 0.033535 2.417382 -1.246219

C 1.173266 -1.169039 0.313184

C 1.788052 -1.007330 -0.934116

C 3.151153 -0.745444 -1.007479

C 3.903318 -0.634180 0.160259

C 3.295922 -0.798315 1.405522

C 1.937766 -1.072845 1.481605

H -1.529731 -2.139147 -2.653347

H -1.124285 -3.295298 -1.274827

H -3.316803 -1.088723 -1.222669

H -2.880683 -2.209754 0.164713

H -1.679111 0.707480 1.162734

H -1.100923 2.732023 2.442524

H 0.207957 4.552106 1.384325

H 0.924404 4.354064 -0.982851

H 0.333854 2.312745 -2.284022

H 1.209852 -1.104663 -1.847429

H 3.627228 -0.629139 -1.975811

H 4.966253 -0.420666 0.100479

H 3.883936 -0.710019 2.313634

H 1.444532 -1.204329 2.439156

O -3.064485 -0.743858 2.405055

H -2.307854 -1.289426 2.130276

H -2.769215 -0.342819 3.228775

DMSO

S 0.258092 0.416583 -0.000000

O -1.098095 1.094980 -0.000000

C 0.258092 -0.782184 1.349690

C 0.258092 -0.782184 -1.349690

H 1.185380 -1.359180 1.322144

H 0.203878 -0.220706 2.283963

H -0.610169 -1.439595 1.257163

H 1.185380 -1.359180 -1.322144

H 0.203878 -0.220706 -2.283963

H -0.610169 -1.439595 -1.257163

H2O

O -0.000000 0.000000 0.119514

H 0.000000 0.754241 -0.478056

H -0.000000 -0.754241 -0.478056

TS-by-A

C -1.079605 -2.132025 0.707012

C 0.365283 -1.752281 0.860775

C 1.229428 -1.762883 -0.250732

C 2.642621 -1.291758 -0.055223

O 0.857985 -2.068144 -1.416694

C 3.345583 -0.859021 -1.184770

C 4.646241 -0.380076 -1.076702

C 5.273063 -0.340163 0.167730

C 4.590011 -0.785880 1.298113

C 3.285291 -1.256910 1.187840

H -1.584444 -2.259399 1.665013

H -1.255472 -2.986912 0.050768

H 0.694912 -1.403298 1.831015

H 2.840246 -0.908177 -2.144175

H 5.173798 -0.037485 -1.962562

H 6.290114 0.030726 0.256208

H 5.077304 -0.770721 2.268859

H 2.773873 -1.618530 2.074719

C -1.361631 -0.828257 0.047289

S -3.380312 -0.636892 -0.484292

O -3.824760 -1.413844 -1.672843

C -4.274308 -1.107473 0.983168

C -3.797294 1.093700 -0.653038

C -1.112038 0.379408 0.912522

O -1.614855 0.427862 2.034579

C -0.371155 1.533212 0.345001

C -0.421327 2.744544 1.046948

C 0.251905 3.860742 0.570700

C 0.990494 3.775332 -0.609395

C 1.053826 2.571206 -1.308073

C 0.374979 1.452795 -0.838708

H -0.960745 -0.807159 -0.965216

H -5.329065 -0.859133 0.854857

H -3.823986 -0.573665 1.823329

H -4.144816 -2.183862 1.104716

H -3.540835 1.609944 0.273739

H -3.217683 1.491132 -1.487281

H -4.867382 1.166283 -0.856728

H -0.998411 2.786044 1.964986

H 0.203109 4.797996 1.116331

H 1.519476 4.647085 -0.982715

H 1.639805 2.500373 -2.219077

H 0.460024 0.516773 -1.381203

TS-by

C -1.175545 -1.699651 0.835216

C 0.315866 -1.565722 0.954938

C 1.141890 -1.754984 -0.167589

C 2.619211 -1.529054 -0.010944

O 0.700292 -2.024842 -1.318997

C 3.363142 -1.274449 -1.168144

C 4.729159 -1.025095 -1.099484

C 5.379310 -1.040767 0.133435

C 4.652922 -1.311798 1.291941

C 3.284376 -1.553261 1.220568

H -1.675057 -1.691818 1.804483

H -1.517876 -2.541513 0.231291

H 0.720050 -1.248224 1.907759

H 2.836359 -1.277572 -2.117216

H 5.289517 -0.818617 -2.007156

H 6.446865 -0.849029 0.191457

H 5.155575 -1.339696 2.254564

H 2.737091 -1.781852 2.130020

C -1.257388 -0.397000 0.115996

S -3.204064 0.098651 -0.402521

O -3.765190 -0.650706 -1.568606

C -4.158912 -0.184618 1.071832

C -3.342108 1.858342 -0.669690

C -0.791750 0.786318 0.927302

O -1.272065 0.967284 2.045653

C 0.133848 1.768965 0.312161

C 0.311991 2.992463 0.971084

C 1.166771 3.953175 0.449296

C 1.859117 3.697740 -0.734298

C 1.694268 2.479632 -1.390954

C 0.834078 1.517125 -0.875672

H -0.869889 -0.489705 -0.898502

H -5.147539 0.260656 0.950119

H -3.606030 0.255943 1.904790

H -4.226998 -1.271593 1.166542

H -3.016266 2.373387 0.235777

H -2.697655 2.113749 -1.511980

H -4.385477 2.085902 -0.896724

H -0.233739 3.167643 1.892482

H 1.295603 4.901307 0.962130

H 2.529733 4.447553 -1.143195

H 2.243166 2.274891 -2.304799

H 0.742068 0.563112 -1.384886

O -4.030911 -3.185352 -0.261397

H -4.969976 -3.369469 -0.156612

H -4.003001 -2.498720 -0.945192

TS1R-A

C 0.971951 -1.220336 1.322040

C -0.078548 -0.916117 2.213256

C -1.465478 -0.903036 1.913976

C -1.941762 -1.404823 0.574040

O -2.339824 -0.506798 2.715346

C -2.841916 -0.617532 -0.149781

C -3.333505 -1.046384 -1.377191

C -2.956731 -2.289382 -1.885867

C -2.086924 -3.097199 -1.157679

C -1.579822 -2.654984 0.062680

H 0.745949 -1.778912 0.418327

H 1.896384 -1.558965 1.788425

H 0.178548 -0.565537 3.211330

H -3.132760 0.347002 0.254985

H -4.012361 -0.411259 -1.939610

H -3.344561 -2.628504 -2.842173

H -1.803410 -4.074488 -1.538190

H -0.912102 -3.293427 0.634604

C 1.737321 0.318054 0.419558

S 3.279876 -0.227655 -0.202112

O 3.303056 -1.624140 -0.671047

C 4.392710 0.011366 1.162869

C 3.907538 0.869812 -1.460155

C 0.844222 0.719464 -0.670557

O 1.010099 0.262247 -1.809748

C -0.286153 1.621454 -0.361629

C -1.098330 2.034573 -1.426553

C -2.192096 2.853819 -1.197333

C -2.496771 3.258498 0.104571

C -1.697528 2.849995 1.168455

C -0.588239 2.041401 0.938306

H 1.914302 1.018596 1.232310

H 4.032922 -0.596483 1.993894

H 4.419311 1.066426 1.437690

H 5.374932 -0.334661 0.837569

H 3.207143 0.816355 -2.292113

H 3.963699 1.879818 -1.051901

H 4.894503 0.496966 -1.739956

H -0.853374 1.688184 -2.425001

H -2.815958 3.173078 -2.026509

H -3.357768 3.894807 0.286833

H -1.936088 3.161075 2.180518

H 0.016388 1.720089 1.778318

TS1R

C 0.971343 0.600488 -1.640038

C -0.120331 -0.076107 -2.208973

C -1.480181 0.073424 -1.847816

C -1.906208 1.201858 -0.955766

O -2.387935 -0.699778 -2.253421

C -1.509514 2.524986 -1.173741

C -1.958698 3.544193 -0.337191

C -2.802307 3.249371 0.731054

C -3.211991 1.933756 0.947245

C -2.779214 0.921115 0.099639

H 0.800494 1.509473 -1.071906

H 1.876133 0.646477 -2.242142

H 0.088818 -0.868616 -2.924744

H -0.864674 2.758285 -2.016135

H -1.651281 4.569377 -0.522228

H -3.144900 4.042431 1.389416

H -3.870210 1.698108 1.778701

H -3.092521 -0.105706 0.258828

C 1.813973 -0.396940 -0.117920

S 3.416994 0.273117 0.023611

O 3.521656 1.713647 -0.269621

C 4.373148 -0.689450 -1.125476

C 4.146286 -0.093810 1.609937

C 1.010309 -0.150051 1.078446

O 1.295882 0.782865 1.841412

C -0.179014 -0.999585 1.314576

C -0.907272 -0.789393 2.493701

C -2.047613 -1.532816 2.754590

C -2.487070 -2.483054 1.828901

C -1.776217 -2.691300 0.650714

C -0.617772 -1.962491 0.400433

H 1.875911 -1.407237 -0.513506

H 3.932712 -0.554391 -2.114318

H 4.355153 -1.740258 -0.833766

H 5.389458 -0.294159 -1.106848

H 5.159577 0.311847 1.595503

H 3.525356 0.404195 2.353147

H 4.151074 -1.174673 1.756867

H -0.560462 -0.029144 3.185600

H -2.604655 -1.368676 3.672014

H -3.387634 -3.056552 2.028750

H -2.111969 -3.401731 -0.097405

H -0.094288 -2.132928 -0.532489

O -1.572704 -3.264934 -2.585139

H -1.855696 -2.322367 -2.519235

H -0.633419 -3.213187 -2.781630

TS1S-A

C 0.736139 -0.750347 1.598763

C -0.627864 -0.516927 1.416385

C -1.339208 -1.398517 0.561159

C -2.792532 -1.151094 0.292833

O -0.773305 -2.344986 -0.042432

C -3.381726 -1.795718 -0.799917

C -4.720248 -1.588027 -1.111877

C -5.493987 -0.734149 -0.327279

C -4.920767 -0.094921 0.770849

C -3.579803 -0.301077 1.078715

H 1.303101 -0.119227 2.278616

H 1.042380 -1.793985 1.591309

H -1.065949 0.419756 1.737418

H -2.760309 -2.457948 -1.394344

H -5.162532 -2.090189 -1.967622

H -6.540284 -0.569721 -0.567918

H -5.521666 0.563393 1.391511

H -3.150931 0.189514 1.946768

C 1.714574 -0.297327 -0.137498

S 3.080422 -1.336125 -0.375902

O 4.043376 -1.019533 -1.451735

C 2.320222 -2.921967 -0.651424

C 3.934704 -1.445918 1.178522

C 2.063041 1.111247 -0.017716

O 3.197802 1.466016 0.330117

C 0.977608 2.107356 -0.215976

C 1.230251 3.427710 0.178696

C 0.247724 4.399433 0.058097

C -1.004335 4.063187 -0.459820

C -1.264189 2.755817 -0.860658

C -0.278606 1.780285 -0.741933

H 0.920384 -0.627976 -0.802166

H 2.178686 -2.984526 -1.732457

H 1.345714 -2.953546 -0.150169

H 3.005152 -3.700412 -0.314663

H 4.241483 -0.426298 1.413121

H 3.266403 -1.844048 1.942617

H 4.793995 -2.100238 1.026901

H 2.207783 3.662987 0.586874

H 0.451092 5.419105 0.370756

H -1.775268 4.822447 -0.552237

H -2.235784 2.490812 -1.266109

H -0.499190 0.771840 -1.070896

TS1S

C 0.357478 -0.858759 -0.950966

C -0.905468 -1.263101 -0.520944

C -2.074452 -0.550623 -0.879506

C -3.394521 -1.005204 -0.341958

O -2.072231 0.447576 -1.653703

C -4.554393 -0.640440 -1.035304

C -5.807891 -1.023445 -0.574374

C -5.921515 -1.765008 0.600911

C -4.774884 -2.118824 1.309172

C -3.519570 -1.744593 0.840020

H 1.174667 -1.572453 -0.902725

H 0.416009 -0.169641 -1.789923

H -0.985669 -2.122001 0.135401

H -4.447751 -0.054347 -1.942537

H -6.698695 -0.742491 -1.128680

H -6.900377 -2.061033 0.966619

H -4.858681 -2.682971 2.233377

H -2.636205 -2.005771 1.413923

C 1.355148 0.367004 0.406369

S 0.385478 1.785560 0.666737

O -0.833939 1.458354 1.435935

C 0.019152 2.531485 -0.895594

C 1.352738 3.009164 1.527307

C 2.672300 0.613784 -0.150947

O 2.933793 1.659429 -0.766821

C 3.691239 -0.467531 -0.044264

C 3.471408 -1.671718 0.636281

C 4.469801 -2.638300 0.690162

C 5.695096 -2.413175 0.066960

C 5.921712 -1.216841 -0.613076

C 4.925616 -0.251926 -0.668910

H 1.247656 -0.254829 1.290268

H -0.591841 1.800190 -1.439665

H 0.973455 2.741341 -1.379389

H -0.568733 3.426997 -0.688977

H 1.579834 2.593788 2.509949

H 2.260087 3.184610 0.948169

H 0.746412 3.912311 1.614913

H 2.525890 -1.868958 1.131149

H 4.290163 -3.568335 1.220152

H 6.473009 -3.169426 0.111058

H 6.875836 -1.039787 -1.099915

H 5.078519 0.685017 -1.193969

O -3.092917 2.551850 -0.131191

H -2.566314 2.259449 0.623446

H -2.906542 1.850015 -0.782218

TS2R-A

C 1.418512 -2.025254 -0.362858

C 0.005737 -1.602345 -0.715539

C -0.970193 -1.639999 0.325129

C -2.384035 -1.269515 -0.004512

O -0.673622 -1.881946 1.514668

C -3.373573 -1.521225 0.950893

C -4.697816 -1.173320 0.712070

C -5.048939 -0.550437 -0.485033

C -4.068326 -0.280307 -1.437777

C -2.745293 -0.640014 -1.201300

H 1.998098 -2.251716 -1.259241

H 1.499740 -2.844845 0.354807

H -0.321116 -1.712953 -1.743670

H -3.073231 -1.989597 1.882882

H -5.457814 -1.381477 1.459902

H -6.081790 -0.271204 -0.672385

H -4.334180 0.218354 -2.365424

H -1.986486 -0.400994 -1.939100

C 1.811794 -0.669164 0.202432

S 3.559452 -0.237585 0.004289

O 4.231978 -0.945466 -1.096896

C 3.625832 1.528534 -0.166776

C 4.342348 -0.585004 1.563037

C 0.918306 0.213813 -0.717108

O 1.381587 0.437723 -1.870740

C 0.003693 1.224514 -0.088829

C -0.555967 2.184144 -0.939727

C -1.437104 3.142018 -0.453498

C -1.765438 3.164584 0.901173

C -1.204425 2.221936 1.759217

C -0.326147 1.258113 1.271502

H 1.598956 -0.597002 1.271099

H 4.675275 1.825705 -0.167982

H 3.083774 1.980566 0.666313

H 3.125773 1.742538 -1.112637

H 3.854472 -0.008628 2.350609

H 4.237294 -1.656033 1.742974

H 5.395675 -0.313498 1.471234

H -0.277613 2.154043 -1.987979

H -1.865879 3.876132 -1.129920

H -2.451274 3.913534 1.286216

H -1.452016 2.233988 2.816782

H 0.067768 0.515539 1.955647

TS2R

C 1.175103 -2.082933 -0.854737

C -0.230265 -1.568405 -1.084404

C -1.186796 -1.738893 -0.050722

C -2.580556 -1.231676 -0.268987

O -0.893309 -2.217805 1.070466

C -3.569454 -1.608965 0.644964

C -4.873648 -1.146446 0.514668

C -5.205134 -0.282383 -0.528266

C -4.224407 0.112897 -1.436060

C -2.921802 -0.359451 -1.309081

H 1.756410 -2.059780 -1.777923

H 1.241984 -3.071573 -0.392265

H -0.554972 -1.386422 -2.101931

H -3.283423 -2.267228 1.459195

H -5.632967 -1.453591 1.228376

H -6.221821 0.086275 -0.629771

H -4.473275 0.797856 -2.241561

H -2.162327 -0.022574 -2.006827

C 1.585456 -0.947873 0.067497

S 3.366623 -0.644511 0.195252

O 4.093419 -0.767267 -1.078045

C 3.558198 0.948162 0.949292

C 3.968771 -1.819782 1.386318

C 0.841326 0.227415 -0.606585

O 1.336855 0.659024 -1.682758

C -0.059478 1.100017 0.198933

C -0.636486 2.195907 -0.456626

C -1.509733 3.044552 0.209532

C -1.816280 2.817562 1.550837

C -1.242569 1.736244 2.214218

C -0.371325 0.880344 1.546718

H 1.244003 -1.160090 1.083271

H 4.608911 1.055127 1.224890

H 2.916449 0.998282 1.831662

H 3.269067 1.700981 0.198917

H 3.484955 -1.650756 2.348970

H 3.736564 -2.813917 1.001166

H 5.048965 -1.680262 1.458024

H -0.385941 2.359853 -1.499495

H -1.952027 3.885922 -0.315924

H -2.497145 3.480847 2.075892

H -1.473880 1.554701 3.259596

H 0.038028 0.032366 2.082622

O 2.673249 2.974326 -1.320215

H 3.439174 2.868832 -1.893973

H 2.100060 2.213327 -1.557580

TS2S-A

C 0.959774 -1.937419 0.704218

C -0.409098 -1.658171 0.133740

C -1.456957 -1.165924 0.976575

C -2.774863 -0.876012 0.326789

O -1.305822 -0.905734 2.185463

C -3.912559 -0.770686 1.132535

C -5.152259 -0.487723 0.571118

C -5.263172 -0.285689 -0.804388

C -4.129179 -0.364474 -1.610995

C -2.889209 -0.662269 -1.052225

H 1.243952 -2.986817 0.823887

H 1.054683 -1.442128 1.673396

H -0.704802 -2.264872 -0.716808

H -3.800579 -0.913504 2.203074

H -6.032960 -0.419708 1.203506

H -6.230103 -0.060258 -1.245082

H -4.210956 -0.187318 -2.679731

H -2.001111 -0.689516 -1.679980

C 1.684817 -1.227871 -0.428813

S 3.338334 -0.526583 -0.184671

O 3.668519 0.495332 -1.192193

C 3.490281 0.105210 1.467332

C 4.464721 -1.901922 -0.233992

C 0.573269 -0.261469 -0.986246

O 0.342785 -0.348810 -2.214656

C 0.376525 1.080548 -0.317375

C 0.148174 2.161229 -1.176169

C -0.014588 3.450401 -0.681624

C 0.046003 3.685256 0.690022

C 0.257025 2.616024 1.557409

C 0.417835 1.324566 1.061500

H 1.867212 -1.910839 -1.266318

H 4.530905 0.409878 1.591310

H 3.222939 -0.669264 2.188048

H 2.823325 0.964718 1.543681

H 4.166095 -2.636176 0.516431

H 4.403526 -2.325829 -1.237462

H 5.469187 -1.522409 -0.037319

H 0.105745 1.955604 -2.240336

H -0.185289 4.274597 -1.368602

H -0.078329 4.690691 1.081233

H 0.286070 2.782722 2.630380

H 0.514036 0.507827 1.766059

TS2S

C -0.927075 1.657482 1.099764

C 0.426182 1.328257 0.517769

C 1.379522 0.571352 1.271573

C 2.682119 0.272796 0.593096

O 1.166884 0.109991 2.408221

C 3.802658 -0.016442 1.375632

C 5.028754 -0.290523 0.779363

C 5.142246 -0.298623 -0.610092

C 4.021800 -0.041632 -1.399265

C 2.794224 0.244863 -0.804439

H -1.092963 2.693598 1.408287

H -1.129811 1.007017 1.953862

H 0.830488 2.047766 -0.189298

H 3.691672 -0.018141 2.455706

H 5.898104 -0.498922 1.396370

H 6.098864 -0.513475 -1.077187

H 4.102001 -0.072789 -2.482177

H 1.912599 0.411605 -1.421826

C -1.668219 1.266486 -0.169629

S -3.405752 0.757735 -0.117835

O -3.821762 0.057752 -1.344780

C -3.714689 -0.221436 1.329910

C -4.336942 2.247335 0.155753

C -0.649955 0.287221 -0.864072

O -0.349695 0.560714 -2.049945

C -0.646418 -1.167778 -0.448502

C -0.522350 -2.095735 -1.488398

C -0.539444 -3.463211 -1.237719

C -0.679999 -3.932447 0.066121

C -0.789788 -3.019679 1.112572

C -0.770317 -1.650016 0.861114

H -1.721577 2.110760 -0.866633

H -4.790798 -0.402053 1.357968

H -3.387617 0.316344 2.220921

H -3.166177 -1.156249 1.209157

H -3.957976 2.747878 1.048651

H -4.198917 2.873694 -0.726704

H -5.386451 1.972716 0.273753

H -0.414640 -1.708049 -2.495643

H -0.446863 -4.164481 -2.062324

H -0.695840 -4.999541 0.267266

H -0.880463 -3.372635 2.135845

H -0.795898 -0.969380 1.702898

O 2.755371 3.438945 -1.008829

H 3.101936 2.537962 -1.039655

H 2.722110 3.623767 -0.064520

TS3-A

C 1.274104 -1.103926 1.562182

C 0.101052 -1.190684 0.587839

C -1.278395 -1.125980 1.203753

C -2.468721 -1.120565 0.309398

O -1.414115 -1.078322 2.419567

C -3.731960 -0.987386 0.899095

C -4.876417 -0.977693 0.114809

C -4.769140 -1.102770 -1.270939

C -3.516270 -1.234295 -1.865200

C -2.366511 -1.244481 -1.081460

H 1.746723 -2.067491 1.773469

H 0.967426 -0.693331 2.527035

H 0.150277 -2.104810 -0.013770

H -3.788986 -0.890428 1.978521

H -5.852771 -0.872348 0.577997

H -5.663662 -1.096306 -1.886852

H -3.433788 -1.328620 -2.943704

H -1.392231 -1.325643 -1.554293

C 2.124734 -0.104003 0.792513

S 3.508302 -0.615967 -0.053349

O 4.807701 -0.840640 0.629986

C 3.649657 0.620551 -1.319713

C 3.013142 -2.066805 -0.944288

C 0.504826 -0.036081 -0.412247

O 0.676304 -0.334372 -1.627917

C 0.012025 1.361946 -0.105631

C -0.149017 2.233945 -1.184387

C -0.607285 3.533199 -0.988974

C -0.918360 3.978188 0.293444

C -0.766388 3.113480 1.376341

C -0.303211 1.816688 1.180283

H 2.346066 0.858908 1.252080

H 3.804677 1.579548 -0.822956

H 2.702198 0.607466 -1.871118

H 4.502726 0.372621 -1.952391

H 3.837240 -2.337527 -1.605560

H 2.836964 -2.861802 -0.218245

H 2.100509 -1.800897 -1.494858

H 0.086825 1.860802 -2.175402

H -0.726522 4.198042 -1.839930

H -1.279364 4.990463 0.449821

H -1.009032 3.450511 2.379982

H -0.194421 1.163019 2.039745

TS3

C 1.329263 -0.544292 1.818438

C 0.155966 -0.933998 0.917215

C -1.217007 -0.801617 1.548088

C -2.418969 -0.959038 0.685508

O -1.334672 -0.528267 2.734177

C -2.398355 -1.679065 -0.514269

C -3.556887 -1.781530 -1.278625

C -4.730862 -1.158454 -0.862393

C -4.755421 -0.440957 0.334087

C -3.607077 -0.349858 1.107682

H 1.871905 -1.411052 2.206112

H 0.984859 0.012378 2.693880

H 0.255808 -1.964986 0.557985

H -1.496098 -2.186872 -0.842257

H -3.542198 -2.350656 -2.203196

H -5.629058 -1.233242 -1.468269

H -5.670041 0.045590 0.658984

H -3.602679 0.202250 2.042113

C 2.123427 0.352157 0.884409

S 3.561805 -0.150696 0.170981

O 4.881175 -0.015114 0.845912

C 3.581182 0.728721 -1.374707

C 3.324233 -1.844036 -0.306041

C 0.399450 -0.031941 -0.339899

O 0.629590 -0.572220 -1.450813

C -0.161510 1.363661 -0.351243

C -0.384656 1.959513 -1.595256

C -0.924252 3.238640 -1.683756

C -1.254739 3.938641 -0.526199

C -1.039438 3.350549 0.719573

C -0.495764 2.074583 0.808220

H 2.187813 1.416587 1.101365

H 4.480027 0.443285 -1.923157

H 3.600404 1.794157 -1.139494

H 2.665685 0.464553 -1.912803

H 4.213793 -2.139942 -0.863609

H 3.245192 -2.437993 0.605353

H 2.422511 -1.930804 -0.914591

H -0.133679 1.393705 -2.486030

H -1.092189 3.687559 -2.658458

H -1.679283 4.935959 -0.592065

H -1.294988 3.888866 1.627385

H -0.334072 1.639992 1.789144

O 0.465364 -3.248866 -1.505986

H 0.110947 -3.561831 -2.344053

H 0.503296 -2.270692 -1.601351

TS4

C 3.875812 -0.330909 0.827740

H 4.741760 0.281859 0.569738

H 3.237863 0.167423 1.559786

C 1.743202 -1.811607 -0.163266

S 2.920642 -0.557668 -0.627288

H 4.118029 -1.449556 1.065319

H 2.479708 -2.567346 0.272561

O 4.090243 -3.063438 0.803014

C 0.680028 -1.371658 0.827804

H 0.069074 -2.263540 1.001417

H 1.126188 -1.106949 1.790184

C -0.217111 -0.206737 0.372832

H -0.288070 -0.200010 -0.718395

C 0.321614 1.147971 0.824598

C -1.603548 -0.384510 0.988452

O -1.867072 0.161453 2.047925

O 1.181597 1.222786 1.686618

C -2.589995 -1.259952 0.303395

C -3.845449 -1.419056 0.905189

C -2.315749 -1.923395 -0.898608

C -4.809332 -2.222902 0.315292

H -4.040617 -0.898916 1.837127

C -3.281992 -2.732142 -1.486792

H -1.351390 -1.819675 -1.384545

C -4.527855 -2.881157 -0.882507

H -5.780912 -2.339468 0.785064

H -3.062029 -3.244805 -2.417629

H -5.281765 -3.510865 -1.345131

C -0.244284 2.369931 0.199144

C -1.286689 2.329786 -0.734091

C 0.294363 3.606573 0.574275

C -1.781538 3.508958 -1.280004

H -1.726451 1.386158 -1.041490

C -0.195859 4.782248 0.024446

H 1.101190 3.619091 1.299644

C -1.236201 4.734318 -0.903799

H -2.592151 3.470855 -2.000645

H 0.229087 5.737336 0.317101

H -1.621823 5.653481 -1.334485

H 1.314705 -2.160141 -1.108663

H 3.912011 -3.502968 1.640668

C 4.015002 -1.467128 -1.688717

H 4.843882 -0.808825 -1.953098

H 4.336016 -2.329740 -1.087601

H 3.453774 -1.757820 -2.577794

O 2.355874 0.648614 -1.273160

TS5

C -3.682169 1.277337 -0.938972

H -3.543374 2.130155 -0.269463

C -2.754742 -0.295556 1.286104

S -4.031284 -0.127252 0.024806

H -2.675979 1.020444 -1.558973

H -3.276686 -0.790672 2.109652

O -1.384498 0.706152 -2.178292

C -1.560280 -1.132554 0.837890

H -1.173470 -1.591084 1.754885

H -1.908507 -1.947800 0.196763

C -0.440294 -0.377137 0.124626

C 0.192917 0.668822 0.971374

C 0.460162 -1.358798 -0.590342

O 0.002897 -2.015749 -1.518252

O -0.179767 0.883373 2.126478

C 1.879292 -1.565176 -0.182324

C 2.801572 -1.965834 -1.154539

C 2.292645 -1.436254 1.147103

C 4.126556 -2.198842 -0.810004

H 2.462506 -2.077293 -2.179590

C 3.614146 -1.694712 1.495941

H 1.581895 -1.147715 1.915258

C 4.534027 -2.064782 0.517431

H 4.843462 -2.487602 -1.572621

H 3.926809 -1.603512 2.531540

H 5.568826 -2.251268 0.788884

C 1.261873 1.514968 0.365159

C 1.379336 1.680628 -1.020256

C 2.166454 2.151942 1.221527

C 2.405949 2.469663 -1.533013

H 0.632309 1.241599 -1.684322

C 3.196914 2.924559 0.703379

H 2.051605 2.016263 2.292523

C 3.319606 3.081644 -0.677926

H 2.492112 2.609699 -2.606755

H 3.906558 3.404122 1.371055

H 4.124831 3.686215 -1.085373

H -2.474915 0.709844 1.608070

H -0.888889 0.180383 -0.843052

H -4.547662 1.430112 -1.588324

H -1.479191 -0.168221 -2.576525

O -4.172801 -1.352328 -0.784229

C -5.506358 0.183469 0.970410

H -5.343850 1.053627 1.608754

H -6.310886 0.373038 0.258399

H -5.719243 -0.706204 1.565259

TS6-A

C 0.744990 -0.596864 0.947595

C -0.553953 -0.155892 0.312633

C 1.159132 -1.059461 -0.385051

C -0.653953 1.096332 -0.408948

O -1.525725 1.348672 -1.250271

C -1.593884 -1.167397 0.314229

O -1.334002 -2.359705 0.545954

C 0.397683 2.145317 -0.163577

C 0.727713 2.574374 1.125547

C 1.654419 3.596157 1.311076

C 2.273619 4.184470 0.209875

C 1.945692 3.765177 -1.078450

C 0.999798 2.762103 -1.263450

C -3.032325 -0.794970 0.127205

C -3.572709 0.380992 0.655351

C -4.936142 0.633407 0.555698

C -5.771700 -0.279403 -0.087462

C -5.240663 -1.455915 -0.612536

C -3.880186 -1.718635 -0.488959

H 1.353206 0.202090 1.365259

H 0.577130 -1.404853 1.658879

H 1.475271 -0.316076 -1.111502

H 0.240507 2.117636 1.983218

H 1.894327 3.932764 2.315299

H 3.006430 4.972594 0.355394

H 2.422868 4.225802 -1.938444

H 0.718617 2.442979 -2.262879

H -2.921976 1.090715 1.156450

H -5.349838 1.543524 0.980188

H -6.835251 -0.076217 -0.173140

H -5.888644 -2.171720 -1.110000

H -3.455086 -2.642367 -0.869361

S 3.225841 -2.006635 -0.283243

O 3.815445 -2.770126 -1.428563

C 4.327722 -0.677404 0.193489

H 4.389375 0.009696 -0.652012

H 3.914316 -0.162987 1.063108

H 5.311146 -1.094254 0.419674

C 3.249475 -3.017227 1.195039

H 4.266182 -3.383465 1.350525

H 2.918323 -2.420995 2.047161

H 2.564323 -3.850056 1.029693

H 0.671198 -1.950352 -0.768423

TS6

C -1.597218 0.014897 -1.266930

C -0.169672 -0.005515 -0.774705

C -1.943163 -0.419078 0.089232

C 0.344205 1.243414 -0.276205

O -0.408144 2.175981 0.083269

C 0.524180 -1.276605 -0.920365

O 0.074079 -2.168608 -1.653169

C 1.811358 1.522108 -0.247978

C 2.668339 1.085558 -1.261481

C 4.021946 1.402976 -1.219507

C 4.530370 2.152901 -0.161154

C 3.676564 2.607156 0.843686

C 2.321085 2.306941 0.790482

C 1.739568 -1.589615 -0.104367

C 2.733033 -2.391943 -0.671824

C 3.866739 -2.729371 0.057360

C 4.001563 -2.294326 1.375560

C 2.996526 -1.526515 1.960013

C 1.871441 -1.172524 1.222408

H -1.945553 1.012191 -1.526637

H -1.770637 -0.726649 -2.044097

H -1.885096 0.311028 0.889831

H 2.272052 0.504462 -2.088443

H 4.680837 1.063592 -2.013020

H 5.589522 2.389850 -0.122763

H 4.069647 3.197093 1.666320

H 1.642056 2.660859 1.559808

H 2.607804 -2.731618 -1.695399

H 4.645446 -3.333710 -0.398528

H 4.885210 -2.560003 1.948352

H 3.089510 -1.202030 2.992152

H 1.086959 -0.576950 1.678881

S -4.239290 -0.632213 0.302295

O -4.855337 -1.998762 0.372986

C -4.785044 0.372885 1.679701

H -4.399604 -0.084412 2.592296

H -4.362821 1.371601 1.531786

H -5.876930 0.393040 1.694238

C -4.929107 0.294433 -1.061624

H -6.015707 0.325070 -0.956796

H -4.485790 1.293308 -1.013891

H -4.648697 -0.215536 -1.984506

H -1.787960 -1.461558 0.348767

O -3.096911 2.611633 0.181840

H -2.127825 2.466878 0.138234

H -3.239563 3.470274 -0.226547

TS7

C -0.189306 -1.514558 -0.976739

C -0.244875 -0.069092 -0.466309

C 0.496037 -2.445449 0.022526

C -1.521086 0.628049 -0.860239

O -1.521529 1.612352 -1.591565

C 0.925990 0.761544 -0.916556

O 1.653365 0.420765 -1.843881

C -2.808567 0.077477 -0.345570

C -2.873099 -0.679658 0.830994

C -4.107127 -1.142929 1.280516

C -5.268393 -0.871857 0.560078

C -5.204289 -0.116588 -0.611432

C -3.979831 0.363005 -1.056893

C 1.184451 2.031579 -0.186103

C 1.933819 3.027046 -0.822793

C 2.201128 4.225579 -0.175481

C 1.736242 4.430883 1.124172

C 1.007889 3.434711 1.770371

C 0.722734 2.236970 1.120357

H -1.204284 -1.910916 -1.070536

H 0.255751 -1.555581 -1.976101

H 0.302178 -3.504187 -0.173728

H -1.966885 -0.860256 1.410906

H -4.163181 -1.718223 2.200182

H -6.225747 -1.245354 0.912098

H -6.109270 0.096951 -1.172363

H -3.907396 0.960597 -1.960297

H 2.291515 2.843137 -1.831112

H 2.771690 5.000647 -0.678399

H 1.947579 5.366387 1.634019

H 0.662529 3.590195 2.788253

H 0.188716 1.444049 1.644103

S 2.260622 -2.290637 0.095210

O 2.725314 -1.051746 0.742285

C 2.796261 -3.726536 0.990898

H 2.298941 -3.697079 1.961935

H 2.526891 -4.628333 0.439731

H 3.877704 -3.647469 1.110250

C 2.934632 -2.458217 -1.537978

H 4.021078 -2.493888 -1.441186

H 2.546459 -3.367146 -2.000026

H 2.617142 -1.561351 -2.076500

H 0.195101 -2.093151 1.069330

O -0.124479 -0.820966 2.214215

H -0.219021 -0.188043 0.688129

H 0.805532 -0.615777 2.366614

by-A

C -0.998135 3.173336 0.603346

C 0.041701 2.104261 0.808153

C -1.450916 1.821955 1.001238

C 0.688737 1.504223 -0.391962

O 0.238713 1.716793 -1.511476

C -2.177901 0.955504 0.007689

O -2.902789 1.446388 -0.841667

C 1.856770 0.598798 -0.208710

C 2.502580 0.131027 -1.360237

C 3.576174 -0.741918 -1.261032

C 4.012880 -1.166839 -0.005862

C 3.373784 -0.713213 1.145282

C 2.302797 0.168989 1.046794

C -1.990612 -0.516654 0.107515

C -1.150984 -1.095188 1.065840

C -0.981554 -2.474895 1.105811

C -1.649425 -3.284735 0.190198

C -2.490424 -2.715037 -0.766932

C -2.659244 -1.338406 -0.807726

H -1.035779 3.973948 1.333314

H -1.181561 3.451345 -0.429646

H 0.624106 2.145009 1.720671

H -1.741418 1.650134 2.035508

H 2.139016 0.466970 -2.325731

H 4.073278 -1.095955 -2.158896

H 4.850758 -1.852936 0.073998

H 3.708885 -1.046799 2.122305

H 1.814959 0.504861 1.955269

H -0.624139 -0.470364 1.778663

H -0.325261 -2.917216 1.848708

H -1.514179 -4.361794 0.220390

H -3.009573 -3.347929 -1.480091

H -3.303866 -0.871963 -1.545744

by

C -1.119823 -2.780417 -0.268450

C -0.708632 -2.017152 0.950112

C 0.294217 -2.323455 -0.172621

C -0.971091 -0.564757 1.209374

O -0.221199 0.016853 1.988340

C 0.723430 -1.165503 -1.021113

O 0.023906 -0.758105 -1.936701

C -2.063707 0.155582 0.516175

C -1.892988 1.514219 0.218878

C -2.913872 2.218938 -0.404626

C -4.118833 1.584235 -0.706946

C -4.301064 0.239319 -0.391826

C -3.271890 -0.479517 0.205783

C 1.994125 -0.479024 -0.666371

C 2.690063 -0.769049 0.512501

C 3.839973 -0.056615 0.836884

C 4.301796 0.946585 -0.011914

C 3.609982 1.244367 -1.187997

C 2.459870 0.536296 -1.511830

H -1.327286 -3.837662 -0.146207

H -1.716735 -2.266057 -1.014199

H -0.516849 -2.572315 1.864083

H 1.070429 -3.034501 0.091808

H -0.951430 1.998575 0.464042

H -2.774671 3.266551 -0.653217

H -4.918094 2.140205 -1.187865

H -5.243416 -0.250532 -0.615772

H -3.413965 -1.525469 0.456692

H 2.319208 -1.527219 1.193520

H 4.370895 -0.278067 1.757115

H 5.197665 1.503655 0.244621

H 3.967772 2.030109 -1.845835

H 1.898399 0.757057 -2.413763

O 1.230821 2.383785 1.314228

H 0.769512 1.617860 1.689380

H 1.691009 2.013250 0.552582

int1'-A

C 0.747416 0.324390 3.354041

C -0.073433 -0.158023 2.418177

C 0.318788 -1.328255 1.599049

C -0.679094 -1.954128 0.691098

O 1.455671 -1.797232 1.681975

C -0.328178 -3.162726 0.072357

C -1.204847 -3.791248 -0.799029

C -2.448538 -3.217308 -1.067273

C -2.807386 -2.018277 -0.458294

C -1.931715 -1.389284 0.420605

H 0.472477 1.178056 3.964473

H 1.714313 -0.137830 3.531635

H -1.042734 0.299171 2.259723

H 0.640678 -3.596272 0.297494

H -0.923833 -4.727958 -1.270327

H -3.135805 -3.705460 -1.751789

H -3.768383 -1.561730 -0.672415

H -2.230817 -0.446488 0.863524

C 1.049447 0.623889 -0.955438

S 2.711951 0.397296 -0.951134

O 3.557532 1.163975 -1.901847

C 2.897752 -1.351750 -1.217151

C 3.337571 0.649508 0.698060

C 0.501252 1.798078 -0.388350

O 1.168138 2.696974 0.163430

C -0.994239 1.908818 -0.418824

C -1.624413 2.569869 0.639700

C -3.011205 2.654008 0.692216

C -3.781954 2.106078 -0.333579

C -3.159020 1.477525 -1.409397

C -1.771678 1.370379 -1.447279

H 0.478595 -0.175118 -1.406969

H 2.422265 -1.592457 -2.169049

H 2.423763 -1.880793 -0.386785

H 3.967436 -1.559671 -1.265228

H 3.003138 1.645705 0.988412

H 2.903342 -0.119320 1.341734

H 4.425005 0.580174 0.651266

H -1.005504 2.999016 1.421588

H -3.493399 3.149339 1.529822

H -4.865051 2.175753 -0.297018

H -3.754558 1.063203 -2.217367

H -1.292006 0.876950 -2.287183

int1'

C -0.721570 -0.243541 -3.534559

C 0.144337 -0.343842 -2.523552

C -0.005914 -1.399787 -1.495707

C 1.132743 -1.671309 -0.586785

O -1.062691 -2.030108 -1.403571

C 0.893918 -2.339293 0.622606

C 1.944658 -2.604225 1.489714

C 3.244788 -2.229733 1.149483

C 3.491243 -1.582369 -0.058050

C 2.439252 -1.292836 -0.918432

H -0.630488 0.533852 -4.285815

H -1.541063 -0.950265 -3.631105

H 0.965787 0.357643 -2.424448

H -0.119570 -2.639104 0.875427

H 1.753510 -3.107248 2.432734

H 4.065572 -2.441468 1.828305

H 4.501756 -1.290464 -0.324793

H 2.644625 -0.786598 -1.855324

C -1.162782 0.699212 0.920263

S -2.823450 0.517597 0.733088

O -3.361384 -0.619070 1.522731

C -3.250341 0.337742 -0.984710

C -3.640964 2.029950 1.191516

C -0.514470 1.728378 0.208813

O -1.118185 2.536573 -0.536463

C 0.973563 1.829037 0.345732

C 1.675101 1.298415 1.431701

C 3.058611 1.418849 1.498956

C 3.756198 2.060261 0.477695

C 3.062942 2.594647 -0.607099

C 1.678031 2.488144 -0.665806

H -0.705001 -0.108388 1.473126

H -2.799822 -0.602757 -1.312119

H -2.796936 1.184477 -1.503866

H -4.337058 0.327701 -1.078689

H -3.495410 2.144574 2.266186

H -3.144083 2.828004 0.636194

H -4.699356 1.952370 0.938817

H 1.140549 0.802845 2.235900

H 3.594641 1.004842 2.347449

H 4.837761 2.145197 0.528207

H 3.602691 3.097443 -1.404316

H 1.114841 2.908833 -1.493079

O -2.370623 -3.250706 0.809291

H -2.733066 -2.412665 1.133734

H -1.937663 -2.990090 -0.018489

int1-A

C 0.514143 -2.125106 1.922260

C -0.781447 -2.204050 2.239818

C -1.908724 -1.769861 1.383654

C -1.762868 -1.687099 -0.096762

O -2.969075 -1.459429 1.917648

C -2.549775 -0.749896 -0.775861

C -2.478476 -0.643179 -2.157532

C -1.632141 -1.485554 -2.878610

C -0.860866 -2.435185 -2.212904

C -0.922330 -2.536057 -0.826340

H 0.861593 -1.771048 0.957271

H 1.278299 -2.390713 2.647446

H -1.087940 -2.510705 3.237633

H -3.191848 -0.093218 -0.198153

H -3.074567 0.103037 -2.673750

H -1.575618 -1.402444 -3.959972

H -0.212808 -3.101612 -2.773700

H -0.338275 -3.293066 -0.314122

C 1.812207 0.882423 0.560216

S 3.190356 0.079031 0.054123

O 3.072740 -1.305837 -0.471633

C 4.254565 0.100510 1.480812

C 4.055725 1.057714 -1.161533

C 0.858189 1.258334 -0.416959

O 1.056012 1.155587 -1.645559

C -0.448601 1.811721 0.066015

C -1.224620 2.538803 -0.840397

C -2.468526 3.033434 -0.468379

C -2.958647 2.792159 0.814963

C -2.195948 2.059030 1.721653

C -0.945079 1.575366 1.350931

H 1.696691 0.964038 1.631297

H 3.736119 -0.434259 2.277987

H 4.458671 1.130625 1.773380

H 5.170772 -0.424358 1.208490

H 3.332920 1.221654 -1.961055

H 4.353608 2.002215 -0.705188

H 4.918786 0.484483 -1.502155

H -0.834078 2.692129 -1.841140

H -3.061697 3.601458 -1.179167

H -3.934854 3.169338 1.105322

H -2.581069 1.850720 2.715182

H -0.378503 0.973612 2.054496

int1

C 0.463643 -1.460236 2.021493

C -0.664064 -1.129391 2.658914

C -1.967042 -0.814336 2.034013

C -2.284419 -1.232281 0.638793

O -2.785909 -0.166381 2.680455

C -3.161648 -0.421613 -0.090988

C -3.507151 -0.752574 -1.393188

C -2.998093 -1.914262 -1.974029

C -2.144366 -2.739307 -1.246767

C -1.784674 -2.400766 0.054244

H 0.524275 -1.612829 0.948904

H 1.389647 -1.589352 2.573908

H -0.658550 -0.963067 3.734281

H -3.538179 0.483491 0.372751

H -4.167108 -0.102782 -1.959888

H -3.267834 -2.175643 -2.993166

H -1.751770 -3.647662 -1.692726

H -1.126176 -3.053973 0.614345

C 2.036467 1.023090 0.226172

S 3.436712 0.116699 0.048921

O 3.442896 -1.327665 0.417891

C 4.641692 0.970290 1.038331

C 4.026628 0.251631 -1.628557

C 0.961178 0.831547 -0.666286

O 0.989158 0.035649 -1.636993

C -0.260053 1.667336 -0.456091

C -1.056913 1.955285 -1.567385

C -2.201280 2.730941 -1.430465

C -2.575573 3.204647 -0.173640

C -1.799577 2.899993 0.942965

C -0.641797 2.141737 0.802204

H 2.038309 1.701053 1.067684

H 4.290314 0.932991 2.070521

H 4.737606 2.000467 0.695430

H 5.581576 0.426560 0.938626

H 3.207699 -0.104728 -2.253882

H 4.250866 1.297094 -1.841077

H 4.911105 -0.379667 -1.719297

H -0.760979 1.558056 -2.532864

H -2.808220 2.960245 -2.301318

H -3.475163 3.802990 -0.063165

H -2.099822 3.247542 1.926731

H -0.057212 1.885919 1.680401

O 1.361772 -2.683668 -1.069661

H 1.087015 -1.802971 -1.376988

H 2.144116 -2.464796 -0.543888

int2'-A

C 1.035194 -0.825073 1.461676

C -0.454043 -0.832819 1.404296

C -1.090306 -1.393700 0.322440

C -2.578840 -1.259896 0.203270

O -0.470247 -1.933596 -0.677019

C -3.149144 -1.326180 -1.072863

C -4.519457 -1.165109 -1.255395

C -5.350975 -0.953288 -0.157423

C -4.798122 -0.909204 1.122407

C -3.426977 -1.062265 1.300424

H 1.415747 -0.221486 2.290887

H 1.427765 -1.840783 1.616909

H -0.999381 -0.270259 2.151548

H -2.484709 -1.507429 -1.911937

H -4.941377 -1.208580 -2.256082

H -6.421735 -0.832940 -0.295089

H -5.440430 -0.761845 1.986468

H -3.011641 -1.045902 2.304003

C 1.626719 -0.278759 0.118648

S 3.119881 -1.134964 -0.366694

O 3.681401 -0.647326 -1.636356

C 2.569024 -2.828368 -0.485842

C 4.296272 -1.069159 0.961168

C 1.873766 1.208182 0.136665

O 2.993769 1.647218 0.375933

C 0.707025 2.085209 -0.074968

C 0.811219 3.424591 0.327050

C -0.263615 4.285556 0.169373

C -1.449622 3.818573 -0.402503

C -1.554647 2.494739 -0.819730

C -0.482422 1.623458 -0.655095

H 0.923788 -0.644780 -0.658541

H 3.115960 -3.251431 -1.330160

H 1.481709 -2.792167 -0.658043

H 2.809126 -3.366120 0.431451

H 4.608913 -0.027948 1.031997

H 3.811678 -1.396861 1.882490

H 5.122519 -1.730283 0.694911

H 1.741519 3.763052 0.771711

H -0.184862 5.318831 0.492332

H -2.291999 4.492974 -0.524919

H -2.473145 2.133058 -1.271404

H -0.573215 0.601006 -1.003476

int2'

C 0.446848 -0.599537 -0.805118

C -0.904015 -1.038965 -0.373834

C -2.051273 -0.436151 -0.821002

C -3.370257 -0.950665 -0.323391

O -2.105072 0.549433 -1.664554

C -4.502202 -0.773510 -1.126890

C -5.750339 -1.232473 -0.719051

C -5.894183 -1.864917 0.514835

C -4.778871 -2.028325 1.335004

C -3.530268 -1.574711 0.920097

H 1.104967 -1.453088 -1.012579

H 0.387897 -0.014570 -1.729270

H -0.960508 -1.879494 0.309609

H -4.375566 -0.263899 -2.076885

H -6.615130 -1.093773 -1.362475

H -6.868776 -2.217673 0.839958

H -4.884247 -2.501317 2.307622

H -2.673893 -1.680977 1.579496

C 1.246527 0.232872 0.250498

S 0.355116 1.758106 0.684539

O -0.781664 1.488988 1.582796

C -0.111057 2.534950 -0.824316

C 1.514767 2.832894 1.497603

C 2.638211 0.537337 -0.278793

O 2.842163 1.579736 -0.888210

C 3.682173 -0.494404 -0.118613

C 3.475801 -1.690670 0.581725

C 4.504140 -2.618960 0.688847

C 5.741011 -2.360603 0.102317

C 5.953405 -1.171714 -0.596732

C 4.929416 -0.244182 -0.708677

H 1.257232 -0.294501 1.208990

H -0.801937 1.819628 -1.323456

H 0.805763 2.719793 -1.385951

H -0.641466 3.449280 -0.554686

H 1.888714 2.304672 2.376458

H 2.318275 3.094188 0.811374

H 0.939424 3.710483 1.797870

H 2.520776 -1.913287 1.045860

H 4.339922 -3.544026 1.231449

H 6.542445 -3.087790 0.189543

H 6.917556 -0.973270 -1.053621

H 5.070570 0.685385 -1.249661

O -3.178167 2.468437 -0.040470

H -2.685252 2.163859 0.730102

H -2.925717 1.798340 -0.715934

int2-A

C 0.942701 -0.748608 -1.535089

C -0.541717 -0.786962 -1.397712

C -1.156077 -1.285121 -0.275009

C -2.645460 -1.166748 -0.152820

O -0.527780 -1.749167 0.762081

C -3.204579 -1.148472 1.129981

C -4.576331 -1.001238 1.312792

C -5.420786 -0.888377 0.210175

C -4.878939 -0.929499 -1.074414

C -3.506555 -1.069283 -1.253558

H 1.257011 -0.060446 -2.325380

H 1.356849 -1.726141 -1.828353

H -1.124661 -0.301908 -2.171048

H -2.530628 -1.253900 1.974212

H -4.989100 -0.978148 2.317944

H -6.492636 -0.779218 0.348550

H -5.530718 -0.860125 -1.941130

H -3.100684 -1.121753 -2.259720

C 1.623162 -0.318749 -0.193648

S 3.067349 -1.336947 0.094094

O 4.072086 -1.388348 -0.979377

C 3.749753 -0.811069 1.647906

C 2.338192 -2.931781 0.419994

C 1.974892 1.141641 -0.129758

O 3.138091 1.515003 -0.246587

C 0.854958 2.088363 0.036568

C 1.051313 3.418827 -0.359428

C 0.028691 4.344421 -0.220450

C -1.193081 3.952376 0.332403

C -1.386576 2.638275 0.748137

C -0.369476 1.700345 0.596836

H 0.913943 -0.683277 0.591487

H 4.556236 -1.503603 1.893045

H 2.963976 -0.840046 2.404958

H 4.126263 0.200434 1.501678

H 2.928993 -3.430686 1.188886

H 1.288602 -2.766445 0.711357

H 2.393950 -3.486364 -0.517923

H 2.009979 3.700581 -0.783065

H 0.176936 5.370922 -0.540692

H -1.993200 4.678346 0.442998

H -2.331388 2.336391 1.189525

H -0.520989 0.686596 0.950808

int2

C 1.117874 -0.787970 1.365335

C -0.010368 -0.190876 2.127027

C -1.357299 -0.326175 1.863179

C -1.828769 -1.342035 0.859436

O -2.284693 0.381231 2.403845

C -1.444078 -2.686215 0.897534

C -1.920489 -3.592735 -0.048009

C -2.789219 -3.165352 -1.049178

C -3.192899 -1.830328 -1.086810

C -2.727683 -0.933244 -0.132220

H 0.848272 -1.693602 0.818839

H 1.945248 -1.058597 2.034512

H 0.265706 0.585541 2.839165

H -0.778686 -3.025621 1.687180

H -1.616044 -4.634667 0.000614

H -3.156002 -3.868725 -1.791358

H -3.872394 -1.489656 -1.863515

H -3.040323 0.106281 -0.150597

C 1.675330 0.226158 0.305923

S 3.375299 -0.276981 -0.124190

O 3.570988 -1.730733 -0.196106

C 4.355568 0.452015 1.165989

C 3.897107 0.562937 -1.602170

C 0.858772 0.221268 -0.978856

O 1.161711 -0.597745 -1.839760

C -0.259843 1.160826 -1.141251

C -0.958127 1.120638 -2.358678

C -2.044913 1.952937 -2.566751

C -2.452833 2.827506 -1.555683

C -1.766033 2.870735 -0.345802

C -0.664569 2.048142 -0.137381

H 1.786536 1.218880 0.751393

H 4.006472 0.044756 2.115856

H 4.245833 1.537137 1.141846

H 5.390308 0.156585 0.984930

H 4.967128 0.375228 -1.707818

H 3.334203 0.129791 -2.427543

H 3.693241 1.630320 -1.497777

H -0.633303 0.420788 -3.121175

H -2.582788 1.919836 -3.508963

H -3.311720 3.473026 -1.715175

H -2.082229 3.528455 0.456549

H -0.183244 2.077159 0.833077

O -1.408773 2.809549 2.917059

H -1.742792 1.879488 2.752426

H -0.727230 2.691489 3.584462

int3'-A

C 0.799254 -1.904329 0.748391

C -0.496945 -1.488687 0.056840

C -1.678822 -1.176929 0.921151

C -2.925705 -0.695752 0.268064

O -1.622299 -1.317362 2.140182

C -4.068297 -0.525773 1.059330

C -5.251610 -0.074239 0.492377

C -5.298966 0.219446 -0.871511

C -4.161498 0.062632 -1.660498

C -2.973836 -0.399701 -1.099807

H 0.945169 -2.964719 0.961409

H 0.937904 -1.335622 1.671428

H -0.795563 -2.238637 -0.683215

H -4.001924 -0.753474 2.118640

H -6.137499 0.052223 1.107555

H -6.224257 0.573341 -1.317208

H -4.200381 0.301913 -2.719306

H -2.072516 -0.493576 -1.709508

C 1.578305 -1.335918 -0.429122

S 3.219777 -0.722901 -0.242036

O 3.736102 -0.129145 -1.488514

C 3.308749 0.425931 1.110115

C 4.247020 -2.077070 0.293466

C 0.257751 -0.385061 -0.876070

O -0.019857 -0.367012 -2.129412

C 0.420591 0.994654 -0.221808

C 0.922464 2.000060 -1.055442

C 1.199278 3.271178 -0.564611

C 0.951351 3.571881 0.775099

C 0.418118 2.591222 1.606401

C 0.157600 1.312163 1.114354

H 1.686256 -2.047304 -1.254996

H 4.370269 0.580175 1.310420

H 2.803594 0.015277 1.984639

H 2.833892 1.350717 0.782071

H 3.855235 -2.478350 1.229704

H 4.214506 -2.830995 -0.494387

H 5.262445 -1.697391 0.423456

H 1.091028 1.745682 -2.096655

H 1.604918 4.031347 -1.226629

H 1.163956 4.563546 1.163475

H 0.207846 2.816741 2.648102

H -0.241689 0.569427 1.795226

int3'

C 0.767752 -1.568160 1.171538

C -0.500104 -1.137632 0.439161

C -1.593201 -0.504695 1.235947

C -2.824056 -0.077921 0.513226

O -1.498276 -0.356433 2.451460

C -3.938926 0.310471 1.263708

C -5.111962 0.694206 0.627507

C -5.176338 0.700699 -0.766156

C -4.062857 0.331637 -1.519037

C -2.884591 -0.061441 -0.887828

H 0.790852 -2.572378 1.598565

H 1.028494 -0.845259 1.948754

H -0.930956 -1.975444 -0.121182

H -3.862850 0.299712 2.346479

H -5.978199 0.986779 1.213278

H -6.094548 0.997161 -1.264778

H -4.111695 0.352357 -2.603877

H -1.997212 -0.318685 -1.474818

C 1.544162 -1.364298 -0.122162

S 3.262349 -0.963136 -0.121143

O 3.774709 -0.717283 -1.481151

C 3.587646 0.413312 0.953520

C 4.126555 -2.312542 0.658143

C 0.343026 -0.352096 -0.718415

O 0.002318 -0.539227 -1.942890

C 0.715135 1.094343 -0.359095

C 1.291985 1.840614 -1.392716

C 1.755545 3.133500 -1.175741

C 1.625351 3.720018 0.083499

C 1.020444 3.001637 1.110692

C 0.571713 1.698689 0.893858

H 1.511490 -2.238641 -0.781315

H 4.672181 0.463947 1.063956

H 3.106013 0.255673 1.918758

H 3.202303 1.307582 0.463597

H 3.744775 -2.445897 1.671925

H 3.939425 -3.201378 0.054158

H 5.190856 -2.069550 0.668517

H 1.366305 1.367786 -2.366485

H 2.215499 3.688015 -1.989144

H 1.984097 4.730066 0.258301

H 0.900701 3.452158 2.091994

H 0.119788 1.164218 1.721236

O -2.820930 -3.312931 -0.956672

H -3.176271 -2.422108 -1.064511

H -3.024865 -3.520989 -0.038984

int3-A

C 1.403796 -0.800791 1.637020

C 0.212007 -1.070805 0.723753

C -1.149397 -0.970708 1.321868

C -2.315084 -1.178432 0.417824

O -1.314814 -0.697330 2.507051

C -3.597414 -1.219322 0.976132

C -4.709461 -1.404489 0.165971

C -4.547645 -1.539335 -1.213648

C -3.274082 -1.485743 -1.776406

C -2.155006 -1.311761 -0.966967

H 1.989548 -1.676958 1.926196

H 1.124791 -0.260242 2.543396

H 0.322700 -2.000400 0.161160

H -3.696783 -1.101929 2.050831

H -5.702613 -1.442094 0.603684

H -5.416549 -1.683238 -1.849495

H -3.152789 -1.578910 -2.851767

H -1.161648 -1.235365 -1.410175

C 2.034477 0.136494 0.599853

S 3.385181 -0.622588 -0.265364

O 3.223899 -2.063997 -0.516064

C 3.619097 0.342328 -1.733737

C 4.825849 -0.339468 0.739682

C 0.698889 0.089928 -0.321053

O 0.840530 -0.290335 -1.566659

C -0.081193 1.397872 -0.128549

C -0.691926 1.940214 -1.258735

C -1.438755 3.112065 -1.177744

C -1.580535 3.766490 0.043869

C -0.968733 3.238077 1.178980

C -0.225836 2.063217 1.093632

H 2.405152 1.111202 0.934089

H 4.393184 -0.147728 -2.326106

H 3.919630 1.351090 -1.445204

H 2.621291 0.321093 -2.194285

H 4.956127 0.730755 0.903581

H 4.665994 -0.861825 1.684301

H 5.679636 -0.763364 0.208053

H -0.552193 1.407087 -2.193614

H -1.909176 3.517831 -2.069605

H -2.160848 4.682223 0.112316

H -1.069913 3.741889 2.136323

H 0.230347 1.668586 1.997058

int3

C 1.451349 -0.213939 1.882635

C 0.295739 -0.793908 1.064961

C -1.075988 -0.591915 1.635497

C -2.248651 -0.967533 0.798228

O -1.235672 -0.068696 2.732066

C -2.140039 -1.802314 -0.319800

C -3.271011 -2.091765 -1.078058

C -4.506183 -1.545790 -0.737237

C -4.617674 -0.712178 0.376186

C -3.495133 -0.431091 1.142189

H 2.133218 -0.937429 2.335566

H 1.104956 0.455904 2.672256

H 0.446119 -1.839892 0.784420

H -1.184399 -2.236938 -0.601037

H -3.186302 -2.744710 -1.941753

H -5.383044 -1.768596 -1.338288

H -5.579239 -0.282948 0.641408

H -3.555277 0.217337 2.010448

C 1.968017 0.565161 0.668092

S 3.545686 0.062802 0.031241

O 4.679675 0.550048 0.840144

C 3.541359 0.667817 -1.628620

C 3.578467 -1.701624 -0.103956

C 0.688665 0.106668 -0.220499

O 0.930756 -0.545025 -1.351203

C -0.251223 1.301416 -0.399833

C -0.935858 1.406083 -1.610087

C -1.845950 2.436048 -1.828908

C -2.081768 3.383837 -0.835579

C -1.397726 3.292919 0.374715

C -0.489210 2.260109 0.590446

H 2.107303 1.643849 0.797452

H 4.428247 0.285947 -2.135726

H 3.561332 1.757506 -1.570377

H 2.591486 0.293437 -2.044488

H 4.521438 -1.952319 -0.593507

H 3.570027 -2.100626 0.911431

H 2.711375 -2.046760 -0.676735

H -0.730884 0.653625 -2.363823

H -2.372689 2.499978 -2.777264

H -2.791664 4.188794 -1.002486

H -1.571548 4.027708 1.155707

H 0.016569 2.204021 1.550621

O 0.861275 -3.084996 -1.169266

H 0.706745 -3.458745 -2.042220

H 0.833881 -2.082625 -1.307620

int4-A

C 1.032704 -0.770543 1.471077

C -0.148644 -0.787389 0.480908

C -1.509246 -0.671569 1.164139

C -2.741862 -0.885964 0.359524

O -1.583630 -0.418826 2.357679

C -3.975011 -0.644071 0.979674

C -5.160209 -0.825939 0.282637

C -5.126278 -1.257003 -1.044137

C -3.905519 -1.502232 -1.668103

C -2.715084 -1.318121 -0.972488

H 1.108295 -1.764313 1.929480

H 0.791848 -0.100438 2.299245

H -0.157291 -1.744545 -0.050336

H -3.976397 -0.310255 2.012090

H -6.111699 -0.633079 0.768464

H -6.053155 -1.401510 -1.591201

H -3.879570 -1.838994 -2.699684

H -1.771874 -1.504316 -1.474412

C 2.308682 -0.292034 0.824646

S 3.383244 -1.309753 0.124747

O 4.373541 -2.161803 0.862111

C 4.308454 -0.292221 -1.014667

C 2.463298 -2.432171 -0.919145

C 0.091696 0.236440 -0.639795

O 0.214891 -0.157711 -1.792175

C 0.155236 1.690649 -0.335518

C 0.540786 2.551640 -1.371204

C 0.613707 3.920469 -1.158981

C 0.292751 4.448240 0.092228

C -0.100588 3.600808 1.124860

C -0.167729 2.226924 0.916436

H 2.765962 0.639195 1.141267

H 4.749110 0.518759 -0.431985

H 3.636428 0.106619 -1.773967

H 5.096408 -0.905320 -1.453721

H 3.184756 -3.020189 -1.487758

H 1.904361 -3.095166 -0.255517

H 1.786908 -1.871439 -1.566549

H 0.782304 2.119952 -2.336805

H 0.919171 4.579499 -1.965920

H 0.348062 5.519625 0.260411

H -0.356025 4.009876 2.097416

H -0.480778 1.583336 1.730055

int4

C 3.855884 -0.295022 0.887894

H 4.668701 0.394964 0.658186

H 3.166155 0.156025 1.603273

C 1.766038 -1.711210 -0.268397

S 2.928086 -0.554716 -0.613399

H 4.220564 -1.264886 1.228812

H 2.961259 -3.052132 0.456024

O 3.711222 -3.613143 0.787822

C 0.688165 -1.367574 0.730045

H 0.080904 -2.270737 0.849348

H 1.102508 -1.148488 1.719267

C -0.243115 -0.184678 0.351174

H -0.337608 -0.151318 -0.737260

C 0.304390 1.153721 0.827016

C -1.607538 -0.393529 0.989339

O -1.858460 0.099216 2.079288

O 1.154875 1.212766 1.700492

C -2.601330 -1.250747 0.287008

C -3.860316 -1.409332 0.880483

C -2.326655 -1.899271 -0.923123

C -4.828804 -2.195664 0.273904

H -4.054867 -0.901952 1.819666

C -3.296538 -2.692276 -1.526996

H -1.356317 -1.799465 -1.398510

C -4.547189 -2.838928 -0.931856

H -5.803901 -2.310284 0.737043

H -3.075656 -3.195075 -2.463125

H -5.304182 -3.455230 -1.407452

C -0.239389 2.393439 0.212695

C -1.304099 2.382955 -0.695687

C 0.340259 3.614956 0.575956

C -1.780383 3.575842 -1.228811

H -1.775929 1.450855 -0.990550

C -0.129977 4.804125 0.037533

H 1.163227 3.604621 1.283071

C -1.192909 4.785592 -0.865964

H -2.609388 3.560595 -1.929224

H 0.327963 5.747090 0.319832

H -1.563222 5.715343 -1.287377

H 1.407828 -2.118341 -1.217507

H 3.574402 -3.641889 1.739946

C 4.077902 -1.407842 -1.676992

H 4.859537 -0.697841 -1.949903

H 4.478803 -2.269149 -1.141801

H 3.526774 -1.720024 -2.565589

O 2.623201 0.783092 -1.207991

int5

C -3.843070 -0.292806 -0.714014

H -4.729936 0.284756 -0.450083

H -3.256780 0.138881 -1.524343

C -1.683881 -1.787196 0.200539

S -2.885821 -0.516756 0.633392

H -4.323729 -2.135904 -1.017425

H -2.280377 -2.629199 -0.161494

O -4.468386 -3.119585 -0.990749

C -0.656548 -1.351167 -0.832880

H -0.045157 -2.237093 -1.032248

H -1.140214 -1.081957 -1.776059

C 0.240917 -0.182241 -0.392297

H 0.298592 -0.158117 0.699070

C -0.289174 1.167517 -0.867532

C 1.635044 -0.371302 -0.986028

O 1.936297 0.197873 -2.022948

O -1.112106 1.236195 -1.764320

C 2.588860 -1.284909 -0.302930

C 3.844497 -1.475041 -0.894875

C 2.283937 -1.953095 0.888887

C 4.777955 -2.315060 -0.306196

H 4.064029 -0.950141 -1.818723

C 3.219742 -2.797169 1.476635

H 1.319426 -1.823523 1.368309

C 4.465780 -2.977919 0.881359

H 5.749610 -2.456237 -0.769055

H 2.976062 -3.312578 2.400036

H 5.195791 -3.635855 1.343043

C 0.248922 2.395593 -0.226609

C 1.254848 2.365378 0.745825

C -0.277044 3.628337 -0.630707

C 1.725944 3.549726 1.301943

H 1.684319 1.425164 1.076929

C 0.188688 4.809388 -0.070831

H -1.055188 3.633577 -1.386903

C 1.192758 4.771139 0.896887

H 2.508549 3.518576 2.053332

H -0.227193 5.761209 -0.386483

H 1.559437 5.694315 1.335534

H -1.207128 -2.054449 1.148981

H -4.010599 -3.442001 -1.773430

C -3.931237 -1.368900 1.801040

H -4.765049 -0.703269 2.030925

H -4.285610 -2.293172 1.343198

H -3.342689 -1.557372 2.699641

O -2.160136 0.573122 1.359728

int6-A

C -0.576664 -0.849278 -0.808245

C 0.696030 -0.160097 -0.387021

C -1.263691 -1.428364 0.434899

C 0.636780 1.153721 0.164644

O 1.538856 1.759193 0.777003

C 1.812463 -1.044613 -0.267634

O 1.678288 -2.287457 -0.353421

C -0.693043 1.877496 0.074464

C -1.258331 2.257511 -1.147183

C -2.462760 2.956872 -1.185244

C -3.125007 3.274380 -0.000025

C -2.564286 2.909585 1.222710

C -1.349546 2.228125 1.257721

C 3.213481 -0.532809 -0.094157

C 3.686301 0.590260 -0.778455

C 5.023059 0.961647 -0.686547

C 5.901819 0.225313 0.108470

C 5.439352 -0.895619 0.794661

C 4.106597 -1.280234 0.676898

H -1.243260 -0.163155 -1.333341

H -0.330577 -1.675852 -1.479264

H -1.294372 -0.710614 1.260817

H -0.746927 2.005161 -2.073089

H -2.885913 3.252499 -2.140948

H -4.069704 3.808865 -0.029588

H -3.070933 3.160471 2.150250

H -0.900168 1.959065 2.209923

H 2.996970 1.164547 -1.388595

H 5.383060 1.828147 -1.234344

H 6.943985 0.521845 0.187306

H 6.119615 -1.475467 1.412292

H 3.738985 -2.169186 1.180536

S -2.982446 -1.870410 0.215157

O -3.558462 -2.615129 1.345273

C -3.820618 -0.332306 -0.067044

H -3.728752 0.249407 0.852129

H -3.355527 0.202639 -0.896917

H -4.865611 -0.559761 -0.280736

C -3.090841 -2.768398 -1.309515

H -4.143976 -3.003339 -1.472056

H -2.693984 -2.157502 -2.121566

H -2.506748 -3.681789 -1.186327

H -0.763034 -2.341422 0.769800

int6

C -1.684725 -0.044249 -1.017832

C -0.222901 -0.033969 -0.660003

C -2.472247 -0.518275 0.199130

C 0.335312 1.229356 -0.347913

O -0.373299 2.250668 -0.099249

C 0.387986 -1.328758 -0.624012

O -0.196679 -2.335192 -1.082301

C 1.810952 1.479472 -0.395455

C 2.627514 0.926334 -1.384850

C 3.988645 1.213197 -1.418203

C 4.547987 2.058124 -0.461824

C 3.735122 2.634224 0.514326

C 2.373090 2.358078 0.535162

C 1.700334 -1.579134 0.062482

C 2.599612 -2.476166 -0.520172

C 3.810496 -2.766518 0.097994

C 4.120104 -2.189843 1.329588

C 3.209406 -1.329130 1.938571

C 2.006967 -1.024903 1.307306

H -1.986951 0.968280 -1.293324

H -1.859040 -0.726619 -1.854833

H -2.277575 0.114427 1.068335

H 2.188397 0.269044 -2.128670

H 4.614002 0.777493 -2.192103

H 5.612115 2.275063 -0.482139

H 4.165632 3.300081 1.256870

H 1.726078 2.807070 1.282476

H 2.339674 -2.930853 -1.471492

H 4.513276 -3.445555 -0.376542

H 5.063714 -2.418273 1.816694

H 3.436624 -0.892738 2.907051

H 1.297951 -0.351706 1.779126

S -4.256358 -0.476789 -0.000261

O -4.813259 -1.670376 -0.659808

C -4.878806 -0.225164 1.642138

H -4.569004 -1.081119 2.243818

H -4.436777 0.703675 2.011834

H -5.966533 -0.164453 1.584180

C -4.649696 1.026312 -0.849395

H -5.733812 1.143482 -0.812385

H -4.122016 1.830920 -0.319964

H -4.310199 0.918736 -1.880274

H -2.260601 -1.568044 0.425528

O -2.715597 2.283167 1.153214

H -1.844822 2.281493 0.673587

H -2.768636 3.138140 1.589309
